# Organ systems of a Cambrian euarthropod larva

**DOI:** 10.1038/s41586-024-07756-8

**Published:** 2024-07-31

**Authors:** Martin R. Smith, Emma J. Long, Alavya Dhungana, Katherine J. Dobson, Jie Yang, Xiguang Zhang

**Affiliations:** 1 Department of Earth Sciences, Durham University, Durham, UK; 2 Science Group, Natural History Museum, London, UK; 3 Centre for Ecology and Conservation, University of Exeter, Cornwall, UK; 4 Department of Civil and Environmental Engineering, University of Strathclyde, Glasgow, UK; 5 Department of Chemical and Process Engineering, University of Strathclyde, Glasgow, UK; 6 Institute of Palaeontology, Yunnan University, Chenggong, Kunming, China

**Keywords:** Zoology, Palaeontology, Evolution

## Abstract

The Cambrian radiation of euarthropods can be attributed to an adaptable body plan. Sophisticated brains and specialized feeding appendages, which are elaborations of serially repeated organ systems and jointed appendages, underpin the dominance of Euarthropoda in a broad suite of ecological settings. The origin of the euarthropod body plan from a grade of vermiform taxa with hydrostatic lobopodous appendages (‘lobopodian worms’)^1,2^ is founded on data from Burgess Shale-type fossils. However, the compaction associated with such preservation obscures internal anatomy^3–6^. Phosphatized microfossils provide a complementary three-dimensional perspective on early crown group euarthropods^7^, but few lobopodians^8,9^. Here we describe the internal and external anatomy of a three-dimensionally preserved euarthropod larva with lobopods, midgut glands and a sophisticated head. The architecture of the nervous system informs the early configuration of the euarthropod brain and its associated appendages and sensory organs, clarifying homologies across Panarthropoda. The deep evolutionary position of *Youti yuanshi* gen. et sp. nov. informs the sequence of character acquisition during arthropod evolution, demonstrating a deep origin of sophisticated haemolymph circulatory systems, and illuminating the internal anatomical changes that propelled the rise and diversification of this enduringly successful group.

## Abstract (web summary)

*Youti yuanshi* is a euarthropod species newly described from a fossilized larva from Yunnan Province, China dating approximately to late Atdabanian stage, Cambrian period, and provides insights into the evolution of arthropods.

## Systematic palaeontology

Superphylum Panarthropoda

Lower stem group to Phylum Euarthropoda^10^

*Youti yuanshi* gen. et sp. nov.

**LSID.** urn:lsid:zoobank.org:act:28BD6A01-5FDC-40EC-973A-63AEB05328A4.

**Etymology.** From Pinyin *yòutĭ*, meaning larva, and *yuánshĭ*, meaning primitive; reflecting the early developmental stage of the fossil and its bearing on the origin of the euarthropod body plan.

**Fig. 1:**
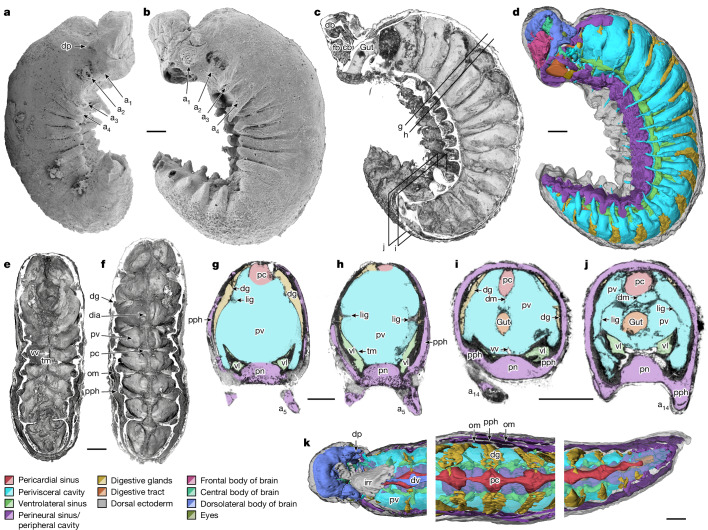
Anatomical overview of *Youti yuanshi*. YKLP 12387. **a**, External scanning electron microscopy, right side. Damage to posterior epidermis exposes lining of perivisceral cavity, demonstrating blind gut. **b**, External scanning electron microscopy, left side. **c**,**g**–**j**, Median virtual dissection from X-ray computed tomography (XCT) data (**c**), showing location of transverse slices intersecting digestive glands (**g**,**i**) and transverse membrane (**h**,**j**). **d**, Semi-manual segmentation of internal chambers from XCT data, viewed from the left side. Dorsolateral aspects of the peripheral cavity are omitted for clarity. **e**,**f**, Virtual dissection parallel to coronal plane, looking ventrally (**e**) and dorsally (**f**), showing digestive glands, pericardial sinus, transverse membranes within perivisceral cavity, and oblique membranes within peripheral cavity. **g**–**j**, XCT sections at positions indicated in **c** at position of digestive glands (**g**,**i**) and at position of ventrolateral lacunae and transverse membrane (**h**,**j**). **g**,**h**, Sections close to the anterior trunk, reflecting segments at late developmental stage. **i**,**j**, Sections close to the posterior trunk, showing superior preservation of internal tissue. **k**, Segmentation of internal chambers from XCT data, viewed from the dorsal perspective at anterior, middle and posterior trunk. Aspects of peripheral cavity are omitted for clarity. a, appendage; cb, central body of brain; db, dorsolateral body of brain; dia, diagenetic grain; dg, digestive gland; dm, dorsal membrane; dp, dorsal projection; dv, dorsal vessel; fb, frontal body of brain; irr, irregular chamber; lig, ligament; om, oblique membrane; pc, pericardial sinus; pph, peripheral cavity; pn, perineural sinus; pv, perivisceral cavity; tm, transverse membrane; vl, ventrolateral sinus; vv, ventral vessel. Scale bars, 200 μm.

**Fig. 2:**
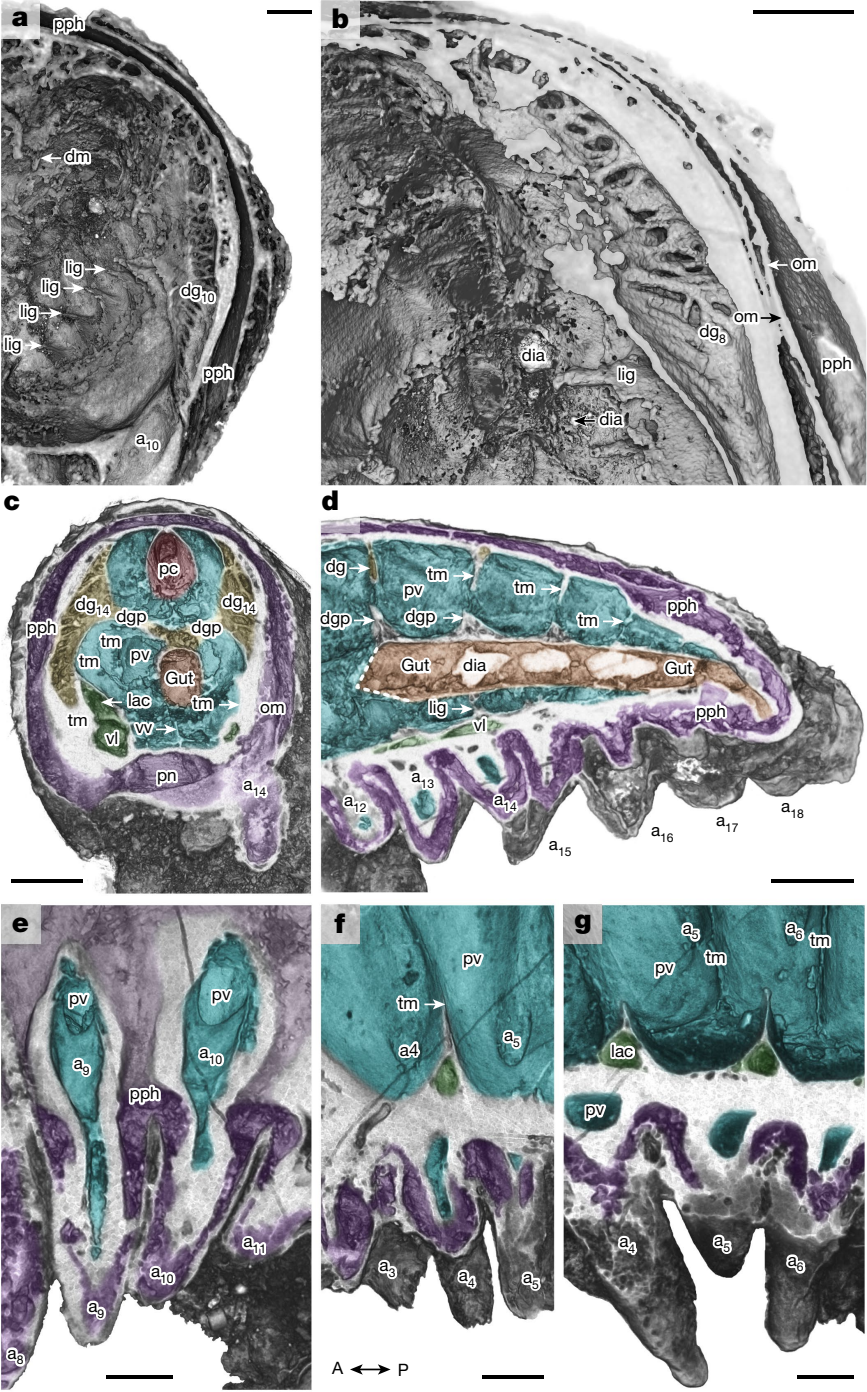
Internal anatomy of *Youti yuanshi*. YKLP 12387. **a**,**b**, Virtual dissections of dorsolateral trunk, looking posteriad, showing internal structure of tenth (**a**) and eighth (**b**) digestive glands, oblique membrane within peripheral cavity and ligaments associated with gut. **c**, Virtual dissection through 14th digestive gland, looking anteriad, showing connection of digestive glands to dorsal gut. **d**, Virtual dissection showing blind termination of posterior gut; dashed line marks anterior limit of gut preservation. **e**–**g**, Disposition of chambers within trunk appendages, showing extensions of perivisceral and peripheral cavities into appendage. Virtual dissections oriented parallel (**e**), oblique (**f**) and subperpendicular (**g**) to appendages. A, anterior; P, posterior. Colour scheme as in Fig. 1. dgp, digestive gland process; lac, lacuna of the ventrolateral sinus. Scale bars, 100 μm.

**Fig. 3:**
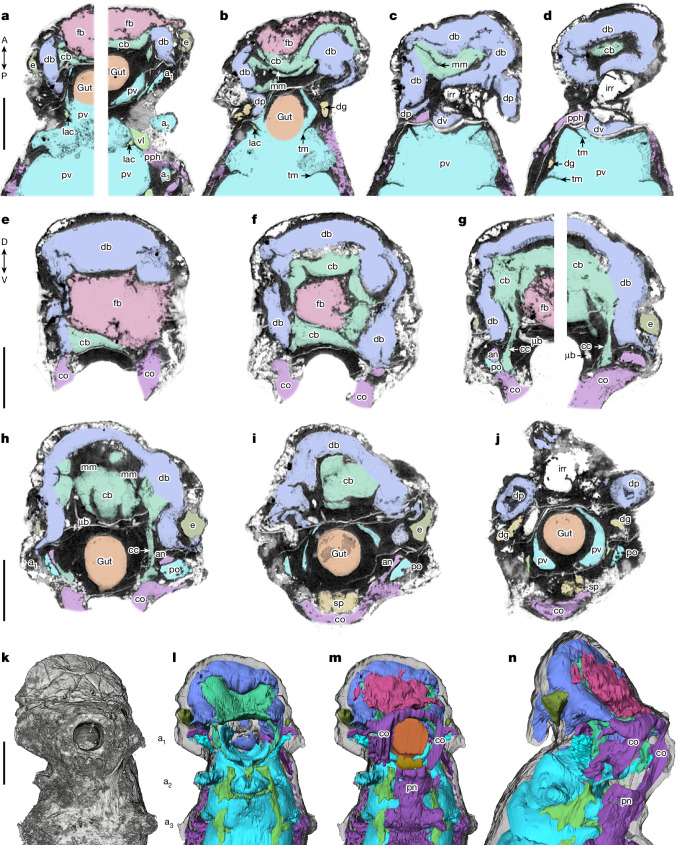
Head structure in *Youti yuanshi*. YKLP 12387. **a**–**d**, XCT sections from ventral (**a**,**b**) to dorsal (**c**,**d**) surface of dorsal lobe, showing internal structure of head and trunk. **e**–**j**, Transverse XCT sections from anterior (**e**) to posterior (**j**) of head. D, dorsal; V, ventral. **k**–**m**, Ventral view of head, showing external XCT volume render (**k**) and segmentation of chambers without (**l**) and with (**m**) ventral structures. **n**, Sublateral view. Colour scheme as in Fig. 1. an, anterior compartment of first appendage; cc, circumpharyngeal connective; co, circumoral ring; e, eye; mm, transverse medial membrane of the central body; po, posterior compartment of first appendage; sp, subpharyngeal gland; µb, microboring. Scale bars, 200 μm.

**Extended Data Fig. 1:**
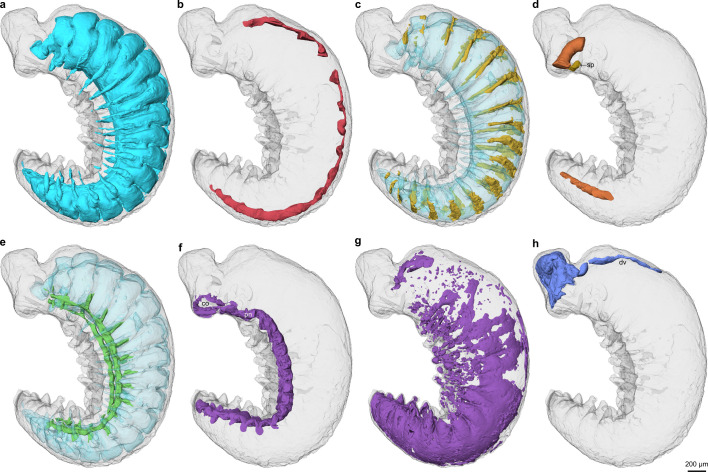
Structure and relationship of major body cavities within *Youti yuanshi*. Location of major organ systems within YKLP 12387, lateral view. **a**, perivisceral cavity; **b**, pericardial sinus; **c**, digestive glands, showing relationship to perivisceral cavity; **d**, gut and subpharyngeal gland; absence of medial gut represents non-preservation; **e**, ventrolateral sinus, showing relationship to perivisceral cavity; **f**, perineural sinus; **g**, peripheral cavity; **h**, dorsolateral body of brain with associated vessel. *Abbreviations:* co, circumoral ring; dv, dorsal vessel; pn, perineural sinus; sp, subpharyngeal gland.

**Extended Data Fig. 2:**
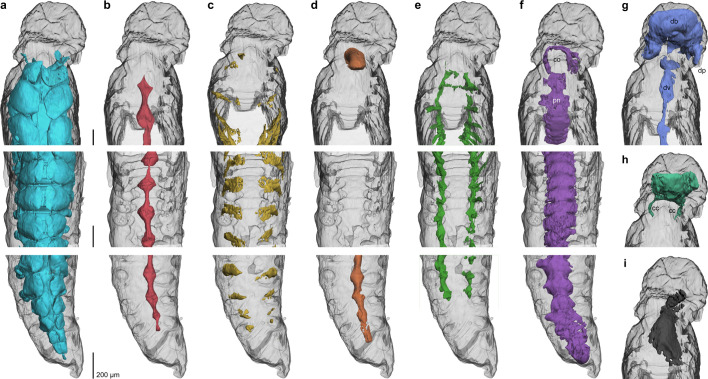
Structure and relationship of major body cavities within *Youti yuanshi*. Location of major organ systems in YKLP 12387, dorsal views. **a**, perivisceral cavity; **b**, pericardial sinus; **c**, digestive glands; **d**, preserved extent of gut; absence of medial gut represents non-preservation; **e**, ventrolateral sinus; **f**, perineural sinus; **g**, dorsolateral body of brain with associated vessel; **h**, central body of brain; **i**, irregular chamber, interpreted as dorsal extra-embryonic ectoderm. *Abbreviations:* cc, circumpharyngeal connective, co, circumoral ring; db, dorsolateral body of brain; dp, dorsal projection; dv, dorsal vessel; pn, perineural sinus.

**Extended Data Fig. 3:**
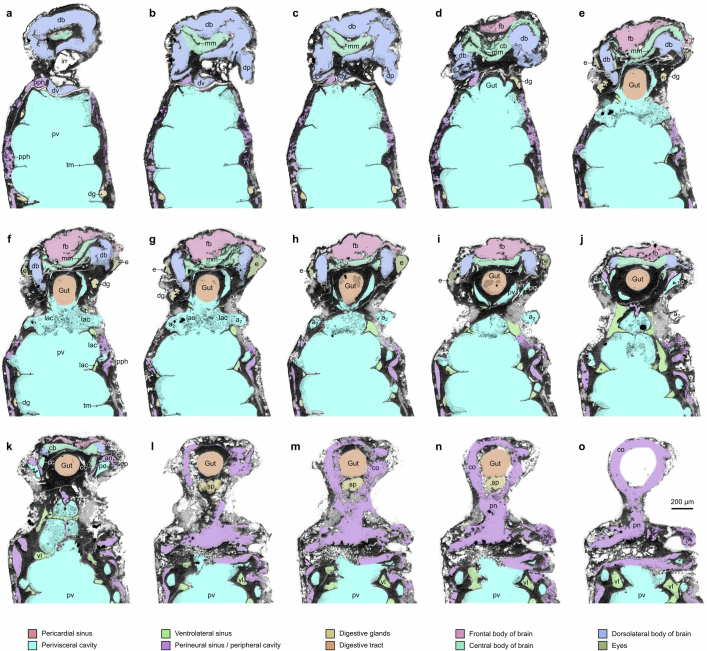
Coronal sections through anterior of *Youti yuanshi*. **a–o**, Virtual dissections of YKLP 12387 rendered from XCT data, from dorsal to ventral. Coloured regions identify organ systems. Raw images available at FigShare^84^. *Abbreviations:* a, appendage; an, anterior compartment of first appendage; cb, central body of brain; cc, circumpharyngeal connective; co, circumoral ring; db, dorsolateral body of brain; dg, digestive gland; dp, dorsal projection; dv, dorsal vessel; e, eye; fb, frontal body of brain; irr, irregular chamber; lac, lacuna; mm, transverse medial membrane of the central body; po, posterior compartment of first appendage; pph, peripheral cavity; sep, membrane separating compartments of the first appendage; sp, subpharyngeal gland; tm, transverse membrane; vl, ventrolateral sinus.

**Extended Data Fig. 4:**
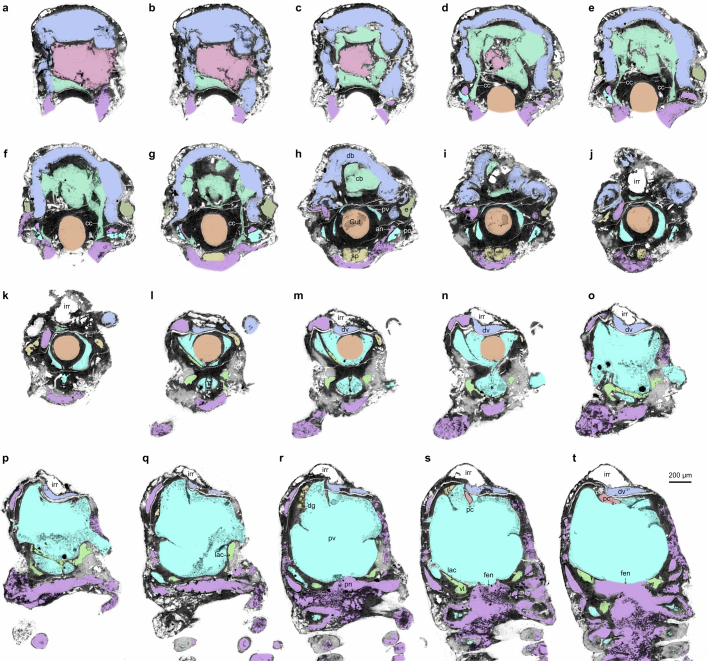
Transverse sections through anterior of *Youti yuanshi*. **a–t**, Virtual dissections of YKLP 12387 rendered from XCT data, from anterior to posterior. Coloured regions identify organ systems; colours as for Extended Data Fig. 3. Raw images available at FigShare^84^. *Abbreviations:* an, anterior compartment of first appendage; cb, central body of brain; cc, circumpharyngeal connective; db, dorsolateral body of brain; dg, digestive gland; dv, dorsal vessel; e, eye; fen, fenestra; irr, irregular chamber; lac, lacuna; pc, pericardial sinus; pn, perineural sinus; po, posterior compartment of first appendage; pv, perivisceral sinus; sp, subpharyngeal gland; vl, ventrolateral sinus.

**Holotype.** YKLP 12387 (Figs. 1–3 and Extended Data Figs. 1–4), recovered by 5% acetic acid digestion of carbonate nodules from black shales of the Yu’anshan Formation (*Eoredlichia–Wutingaspis* Biozone, approximately late Atdabanian stage, Cambrian Period Series 2, Stage 3), Xiaotan section, Yongshan, Yunnan Province.

**Diagnosis.** Euarthropod with paired glands dorsal to lobopods. Bulbous anterior appendages adjacent to ventral mouth. Perivisceral cavity incompletely partitioned by transverse membranes of connective tissue. Ventrolateral sinuses with serially repeated dorsal lacunae. Prominent dorsal head lobe with paired dorsal projections. Subdivided brain with discrete frontal body.

**Preservation.** Orsten-type preservation^11^ typically replicates chitinous cuticle in amorphous apatite; preservation of more labile tissue^12^ is rare. Concretions within the Yu’anshan Formation are exceptional in preserving non-chitinous material, including coprolites and muscle, at exquisite resolution^9,13^. This material is often penetrated by post-phosphatization microborings^13^ with diameters on the scale of 10 µm. Secondary encrustations of diagenetic phosphate, although evident in similar deposits^14^, are absent. Small grains of diagenetic minerals (Figs. 1f and 2b) are readily identified by their higher X-ray attenuation, which corresponds to a higher greyscale value.

Although the limited material available cannot support a detailed taphonomic model, the high fidelity indicates an early onset of phosphatization, with differential preservation of different tissue types^9^. In YKLP 12387, preservation is restricted to the integument and connective tissue, leaving behind voids that correspond to the outlines of non-phosphatized tissue.

## Description

YKLP 12387 is curved, with 20 segments (Fig. 1a–d). Paired ventrolateral lobopods emerge from the midpoint of each segment (Figs. 1a–d,g–j and 2a,c–g). The specimen is 3,900 µm long and reaches 900 µm high by 360 µm wide. Appendage one is highly modified; appendage two is incompletely preserved (Figs. 1a,b and 3a). Appendage four is the longest, with each subsequent appendage being progressively shorter. The appendages transition from subcylindrical to subconical posteriad (Fig. 1a,b). The main cavity within the trunk, interpreted as perivisceral, surrounds the gut and extends into the appendages (Figs. 1c–k and 2 and Extended Data Figs. 1 and 2). It is flanked by dorsal, ventral and ventrolateral sinuses, and surrounded by a peripheral cavity (Figs. 1c–k and 2c,d and Extended Data Figs. 1 and 2).

The gut is an unornamented tube that lies centrally within the perivisceral cavity, opening directly through the mouth (with no buccal cavity), and terminating blindly in the final segment (Figs. 1a–d,i–k and 2c,d). There is no evidence of differentiation within the gut, although the gut is not preserved between segments 2–12 (Extended Data Figs. 1 and 2). We interpret the abrupt change in gut preservation at the posterior limit of segment 1 (Fig. 1c and Extended Data Figs. 3d–h and 4k–n) as denoting the end of the foregut and thus the position of the stomodeum^15^.

**Extended Data Fig. 5:**
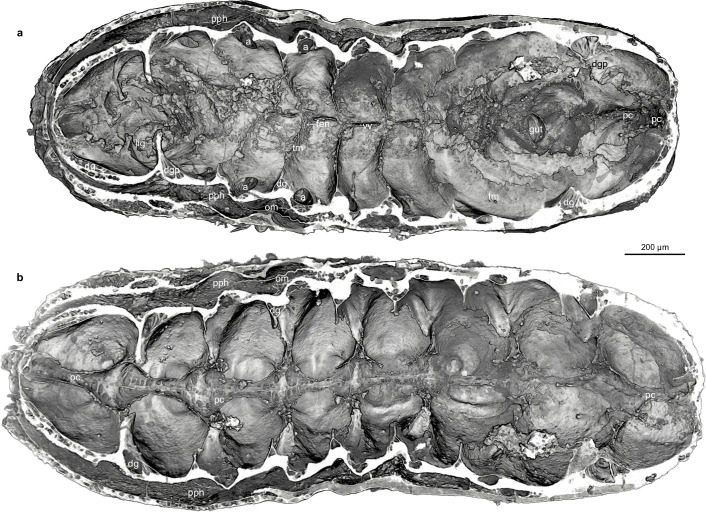
Virtual dissection of *Youti yuanshi*. Enlargement of Fig. 1e–f, Rendered from XCT data parallel to coronal plane, looking **a**, ventrally; **b**, dorsally. *Abbreviations:* a, appendage; dg, digestive gland; dgp, digestive gland process; fen, fenestra; lig, ligament; om, oblique membrane; pc, pericardial sinus; pph, peripheral cavity; tm, transverse membrane; vv, ventral vessel.

The gut attaches to the wall of the perivisceral cavity with lateral and ventral ligaments and a dorsal membrane (Figs. 1g–j and 2a,d). The dorsal membrane connects to a tubular dorsal sinus (Extended Data Figs. 1b and 2b) with a fenestrated wall (Fig. 1f and Extended Data Fig. 5b) and a fluctuating width (Fig. 1f–k and Extended Data Figs. 2b and 5b): it is sub-circular in transverse section where the legs emerge from each segment, but four times narrower between these points. On the basis of its position, size, shape and surface texture, we interpret this sinus as pericardial.

A pair of ventrolateral sinuses run along the base of the perivisceral cavity (Figs. 1d,g–k and 2c,d and Extended Data Figs. 1e and 2e). In the posterior region of each segment, a lacuna extends dorsolaterally from each sinus, following the floor of the perivisceral cavity (Figs. 1d,h,j and 2c,f,g). A further ventral sinus opens dorsally into the perivisceral cavity through fenestrae situated between these lacunae (Fig. 1e,g,j and Extended Data Figs. 1f, 2f and 4s,t). The anterior of this sinus connects to a ring that surrounds the opening of the mouth (Figs. 1d and 3e–j,m,n and Extended Data Figs. 1f, 2f, 3l–o and 4a–o). We interpret the sinus as a perineural sinus, and the ring around the mouth as housing a circumoral nerve ring.

The perineural sinus opens ventrally into a peripheral cavity that surrounds the perivisceral cavity (Extended Data Figs. 1g and 4o–t), and is interrupted at irregular intervals (approximately every three appendages) by oblique membranes (Figs. 1f,k and 2b and Extended Data Fig. 5). The peripheral cavity forms the main chamber within each appendage, which surrounds a sacculus of the perivisceral cavity (Figs. 1d,g,i,j and 2e–g and Extended Data Figs. 3k–o, 4t and 5).

A dorsolateral pair of lunate voids occur at the midpoint of each segment (Extended Data Figs. 1c and 2c). These extend dorsally to the margin of the pericardial sinus, and ventrally to the level of the appendages (Fig. 1d–k); they connect to the dorsal gut via a central process (Fig. 2c). Internally, each void contains branching or anastomosing tubes that extend from its medial inner surface to its outer margin (Fig. 2a,b). We interpret the voids as digestive glands, as their morphology, position and internal structure correspond to digestive glands in Cambrian euarthropods^16^.

The perivisceral cavity is incompletely partitioned by serially repeated transverse membranes of connective tissue that house the digestive glands and the lacunae of the ventrolateral sinuses, and to which the gut ligaments attach (Figs. 1c–j and 2a–d). A diminutive vessel runs along the ventral midline of the cavity (Figs. 1e,i and 2c and Extended Data Fig. 5).

**Fig. 4:**
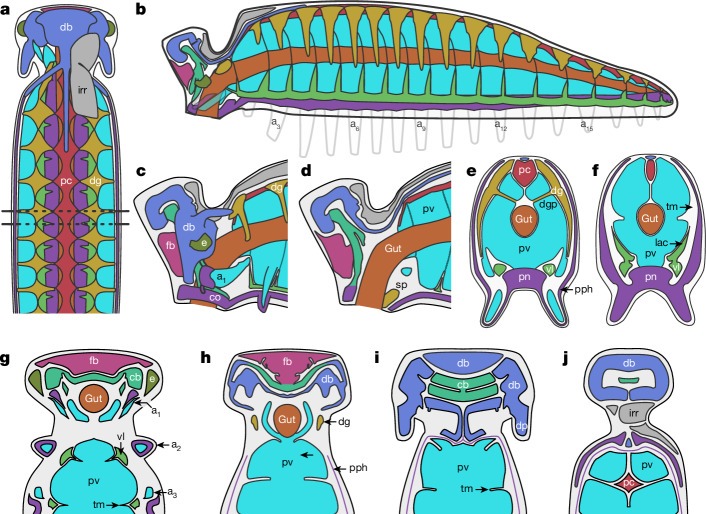
Interpretative drawings of *Youti yuanshi*. **a**, Organ system disposition in sagittal view. Dotted lines denote location of sections shown in **e**,**f**. **b**, Organ system disposition in transverse view. **c**,**d**, Head, from lateral perspective (**c**) and as medial transverse section (**d**). **e**,**f**, Transverse sections through trunk at location of digestive glands (**e**) and transverse membranes (**f**). **g**–**j**, Coronal sections through head, from ventral (**g**) to dorsal (**j**) planes. Colour scheme as in Fig. 1.

Taking these organ systems together, we can thus define a typical body segment as containing: one pair of appendages; one pair of ventrolateral sinus lacunae; one pair of digestive glands; and ligaments that link the gut to a connective membrane (Fig. 4). The only notable differences between trunk segments are changes in the relative proportions of each lacunar system in line with the posteriad reduction in segment size. The exception is the highly modified first segment, which we term the ‘head’. Like other segments, it contains a pair of (modified) appendages, a single (reduced) pair of lacunae of the ventrolateral sinuses, a single pair of digestive glands, and a single (enlarged) transverse membrane within the (significantly reduced) perivisceral cavity (Fig. 3). It additionally bears a dorsal lobe, comprising a pair of dorsal projections, a complex of connected chambers that presumably housed the brain, and further structures that have no equivalents in subsequent segments (Figs. 1a–d,k and 3).

The head appendages form a pair of domed protrusions lateral to the mouth (Figs. 1a–d and 3k–n). Internally, each of these appendages houses a teardrop-shaped chamber that is divided into an anterior and posterior compartment (Fig. 3h–j,l–n and Extended Data Figs. 3i–k and 4e–j). The base of the anterior compartment is contiguous with the circumoral nerve ring (Fig. 3i,m and Extended Data Figs. 3k–m and 4h); by comparison with subsequent appendages, we interpret the posterior compartment as representing a detached sacculus of the perivisceral cavity (Figs. 1d,j, 2d–g and 3i,j,l and Extended Data Figs. 3h–k and 4g–l). The dorsal projections, by contrast, comprise a single undivided cavity that is continuous with the brain (Figs. 1a,k and 3b,c,j,n and Extended Data Figs. 2g, 3b–e and 4g–m), demonstrating that they are not appendicular in origin.

The brain itself—or strictly, the void in which it sat—comprises a frontal, central and dorsolateral body (Fig. 3a–i,l–n and Extended Data Fig. 2g,h). The frontal body is a largely undifferentiated wedge-shaped structure, separate from the rest of the brain except for a posterior connection with the central body (Fig. 3b,g and Extended Data Fig. 3e–j). A transverse median membrane divides the central body into anterior and posterior divisions, which fuse laterally (Fig. 3b,c,h and Extended Data Fig. 3a–k). The anterior division of the central body connects to the perineural sinus, via ventral circumpharyngeal connectives (Fig. 3a,g,h,l); and into the dorsolateral body (Fig. 3a–c,f–i), which caps the head and fills the dorsal projections (Fig. 3a–j,l–n and Extended Data Figs. 2h and 4e–g).

The head also contains subsidiary chambers. We interpret lateral sub-spherical voids (Fig. 3a,g–i,l–n and Extended Data Figs. 3e–i and 4d–i) as eyes. Immediately behind the mouth, within and slightly dorsal to the circumoral nerve ring, is a self-contained three-lobed structure, potentially opening ventro-anteriad into the digestive tract (Figs. 1d and 3i–j,m and Extended Data Figs. 3l–n and 4h–j). This subpharyngeal structure is tentatively interpreted as a digestive or masticatory gland, although comparison might also be made with the subpharyngeal ganglion of certain tardigrades^17^. Finally, an irregular chamber overlies the dorsolateral body of the brain and continues into the second segment on the right-hand side of the body, external to all other cavities (Figs. 1c,d,k and 3c,d,j and Extended Data Figs. 2i and 4i–t). Given its asymmetric location and its position dorsal to the brain and peripheral cavity, we interpret this as dorsal extra-embryonic ectoderm forming an originally yolk-filled trophic vesicle^18^. At points, the ventral wall of this chamber connects (possibly taphonomically) into a medial vessel of varying width that also runs dorsal to the peripheral cavity, extending posteriad from the dorsolateral body of the brain to the posterior limit of segment four (Figs. 1k and 3c,d and Extended Data Figs. 1h, 2g, 3a,b and 4l–t).

## Affinity

The dorsal ectoderm, blind gut and curved body identify YKLP 12387 as a larva. The preserved morphology (Fig. 4) combines euarthropod synapomorphies such as serially repeated digestive glands^16^ with panarthropod plesiomorphies not found in the euarthropod crown group, such as fluid-filled lobopods^19^. In combination, these characters place *Youti* in the euarthropod stem group.

**Fig. 5:**
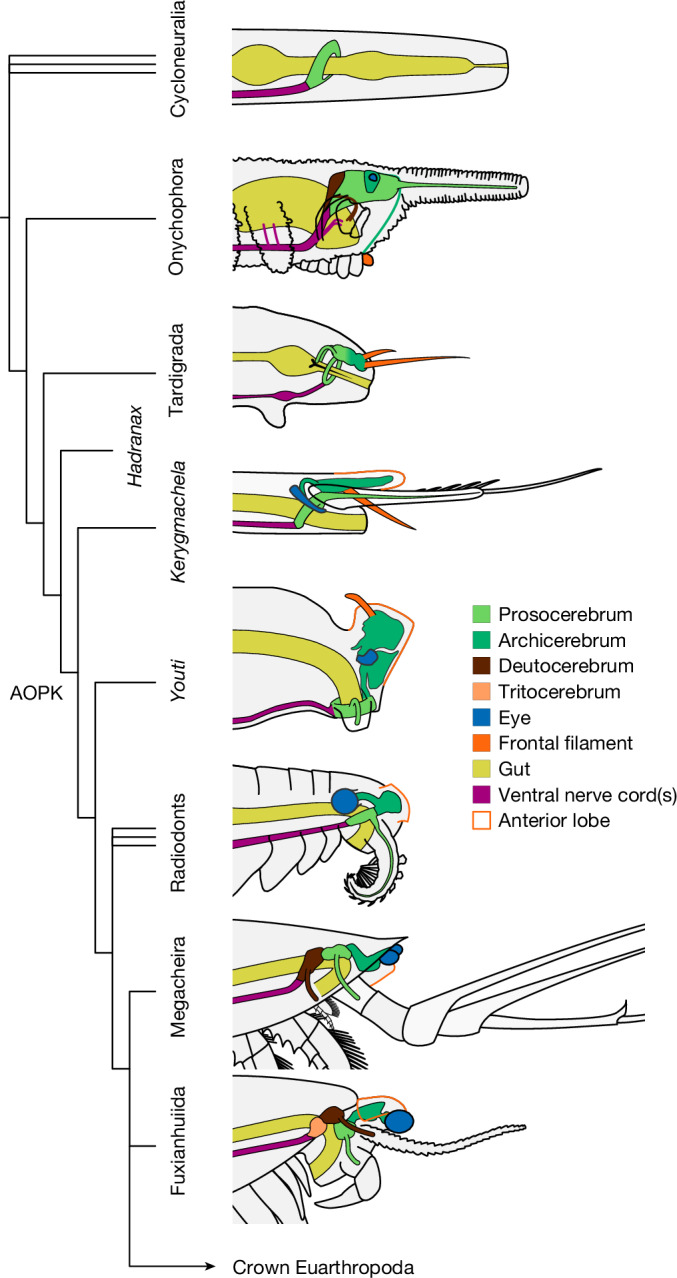
Proposed homology of brain components in early panarthropods. Phylogenetic analysis situates *Youti yuanshi* within the AOPK clade containing *Anomalocaris*, *Opabinia*, *Pambdelurion* and *Kerygmachela*. Under our preferred model, the circumoral brain ring of cycloneuralians corresponds to the panarthropod prosocerebrum, which innervates the first appendage pair (onychophoran antennae, tardigrade stylets or euarthropod labrum). We interpret the archicerebrum as a distinct development dorsal to the prosocerebrum, associated with sensory receptors: specifically the eyes, and the dorsal projections (*Kerygmachela* rostral spines, tardigrade cirri, crustacean frontal filaments or anterior paired projections of stem euarthropods; homology with the anteriormost onychophoran lip papillae is plausible, but may not be parsimonious). The taxa depicted in this figure are selected in order to depict the evolutionary context of *Youti*; the relationships shown are recovered under all analytical conditions.

The first-order relationships of stem group euarthropods are reasonably established (Fig. 5), with many key nodes consistently recovered and defined by morphological synapomorphies^10^. As this phylogenetic framework emphasizes adult morphology, the interpretation of *Youti* requires a degree of caution: the absence of features such as annulations, claws, setal blades, or compound eyes, or the location of its first appendages, may reflect its ontogenetic stage rather than its phylogenetic position.

This said, the presence of midgut glands places the fossil crownwards of *Hadranax* (Fig. 5). The anterodorsal head lobe supports a position within the ‘AOPK’ group containing *Anomalocaris*, *Opabinia*, *Pambdelurion* and *Kerygmachela*, whereas the ventral position of the mouth and the apparent absence of dorsal nodes point to a position crownward of *Kerygmachela*, and—unless sclerotization and segmented appendages arose at a later ontogenetic stage—outside the sub-clade containing radiodonts and crown euarthropods.

Quantitative phylogenetic analysis (summarized in Fig. 5 and Supplementary Information) confirms this position as the most parsimonious, despite the absence of any obvious precursor to dorsal flaps or setal blades: flaps may have evolved multiple times in derived groups^20^, or be secondarily or ontogenetically absent in *Youti*. To explore the possible influence of ontogeny, we repeated our phylogenetic analyses after re-coding as ambiguous any character in *Youti* whose state at adulthood is not unequivocal. This extremely conservative analysis also places *Youti* immediately stemwards of radiodonts, though with less resolution regarding its relationship to *Pambdelurion* and *Kerygmachela*. Whereas our interpretation is not contingent on the relationships between the panarthropod phyla, our analyses recover very strong support (Bayes factor = 11.6) for Tactopoda over Arthropoda (Supplementary Information).

## Discussion

### Digestive glands

Gut glands, which denote carnivory^16^, are offset posteriad within the segments of *Youti*, as in lobopodians^16,21^, dinocaridids^22^ and upper-stem euarthropods^5^, showing that this offset denotes the ancestral euarthropod condition.

### Circulatory system

The onychophoran-like^19^ configuration of the peripheral haemolymph system in *Youti* appendages corroborates the functional equivalence of Cambrian and modern lobopods. The proximity of digestive glands to the pericardial sinus presages the integration of gut glands into the euarthropod (and specifically crustacean) lacunar system^23,24^, and the distribution of the pericardial and ventrolateral sinuses corroborates reconstructions of the ancestral euarthropod vascular system based on modern^19,25^ and fossil^3,26^ representatives, alleviating concerns that vascular systems have been misinterpreted in compression fossils^27^.

In turn, the broad peripheral lacuna that extends into appendages presents a biological interpretation for enigmatic structures in fossil taxa, such as the internal structure at the centre of many lobopods^4,28^, the ambiguous internal structure in *Thanahita*^29^, and the ‘tonguelets’^30,31^ and the interpreted alimentary canal^32^ of radiodonts and higher euarthropods. The perivisceral cavity offers a potential interpretation for the axial structure of *Opabinia*^33^, which circumscribes digestive glands (fig. 3c in ref. ^33^) and extends into lobopods^20,21^; and the ‘pharynx’ of *Megadictyon* (fig. 1a,b in ref. ^16^), which extends into the proximal lobopods. Structures interpreted as a nervous system in *Cardiodictyon*^15^ match the position and arrangement of the perineural sinus and peripheral cavity in *Youti*, including a ventral region that extends into the appendages, and a thin peripheral sheath that encloses the lateral flanks of the trunk.

### Nervous system

The panarthropod protocerebrum comprises a developmentally anterior prosocerebrum^34^, associated with the expression of *six3*^35,36^ (also known as *optix*), which innervates the first appendage pair and is associated with the mouth early in development^37^; and a posterior archicerebrum, associated with *orthodenticle*, which innervates the eyes and frontal sensory elements (Fig. 5). In *Youti*, we interpret the circumoral nerve ring as prosocerebral based on its integration with the mouth and first appendages, and the dorsal lobe as archicerebral due to its association with the eyes and non-appendicular dorsal projections. The *Youti* archicerebrum likely corresponds to the concentration of neural tissue within the *Kerygmachela* anterior lobe^38^; the ‘median eyes’ in *Stanleycaris*^31^, *Kylinxia*^39^ and *Opabinia*^40^; and the frontal organ of higher euarthropods^41^, whose archicerebral nature is corroborated by its position, sensory role, and association with stalked eyes^41–44^ (Fig. 5).

Given its deep position in the euarthropod stem group, we expect *Youti* to retain some characteristics inherited from the ancestral panarthropod, as well as displaying a subset of derived euarthropod characteristics. Features shared with onychophorans are presumably inherited from their common ancestor with euarthropods: (1) the connection of the perineural sinus(es) to the perivisceral sinus and to the main lobopod cavities^19^; and (2) the configuration of the protocerebrum from an undifferentiated frontal body and a central body that is subdivided by a transverse membrane into laterally fusing anterior and posterior divisions^45^.

Characters inferred to be inherited from the ancestral panarthropod include: (1) the protocerebral brain, confirming that the euarthropod deutocerebrum has a separate origin from that of onychophorans^10,36^; (2) the frontal filaments^44,46^; and (3) the circumoral component of the brain, whose vestiges may be represented by circumoesophageal neural tissue in radiodonts^31^ (Fig. 5).

If the prosocerebral nerve ring of *Youti* corresponds to the circumoral nerve ring of cycloneuralians^47,48^ (Fig. 5), then the archicerebrum can be considered as a posterodorsal elaboration of the brain within the panarthropod lineage, potentially accompanying the specialization of sensory structures within this group. The tardigrade brain may reflect an intermediate situation, in which a prosocerebrum, which innervates the appendages (stylets)^17^, and together with the outer connectives forms a ring around the digestive tract^49^, is not prominently differentiated^37^ from an outer, dorsolateral archicerebrum that innervates the eyes and frontal sensory elements (cirri)^17,50^ (Fig. 5).

## Conclusion

The three-dimensionally preserved organ systems in *Youti* offer a revised template for the interpretation of carbonaceous compression fossils. The lacunar system provides a compelling interpretation for structures otherwise attributed to the digestive or nervous systems, or dismissed as taphonomic artefacts. The separate integrations of the deutocerebrum into euarthropod and onychophoran heads denote parallel increases in complexity, indicating that early euarthropod fossils are expected to exhibit protocerebral brains. Segmentally arranged haemolymph chambers and digestive glands denote the sophisticated internal anatomy attained by euarthropods before the arthropodization of the body wall. Together, these observations clarify the sequence of evolutionary events that established Euarthropoda as a diverse and dominant presence in Phanerozoic ecosystems.

## Methods

### X-ray computed tomography

XCT was conducted on the imaging branch of beamline I13, Diamond Light Source under a polychromatic (pink) beam using a 3.2 mm aluminium filter and a 120 ms exposure. One thousand projections were collected during a 180° rotation of the fossil at 4× magnification on a pco.edge camera with an effective pixel size of 1.625 µm. Data were reconstructed using I13 standard filter back projection protocols.

### 3D visualization and analysis

The XCT data were visualized and analysed in Avizo 3D 2022.2 (Thermo Fisher Scientific). Gross anatomy was visualized using ‘standard’ volume rendering of the greyscale reconstructed data, highlighting finer details with the Edge 2D and Edge 3D functions and ‘diffuse’ lighting. Digestive gland anatomy was rendered using ‘physical’ rendering, ‘phong’ lighting and a ‘glossy’ material style. Virtual dissections were achieved using an optimally rotated orthoslice.

The distinct organ structures were semi-manually segmented (Segmentation Workroom) using a combination of the ‘magic wand’ tool (to select a specific range of greyscale values within an additional contrast limit) and the ‘paint brush’ tool for further refinement and subjective delimitation of adjacent and continuous structures into discrete labelled objects. Small structures with complex geometry were manually segmented in each slice, while larger and simpler structures were segmented in every 5th–20th slice (at a resolution sufficient to capture their morphological variation), with the volumes defined by interpolation. Surfaces of the refined labelled objects were rendered using an unconstrained smoothing (extents varying between 3 and 9 depending on the size of the structure). Surface renders were compiled and adjusted as layers in the GNU Image Manipulation Program, version 2.10.32. Additional virtual greyscale dissections were visualized in Dragonfly (Object Research Systems); colour palettes were generated using iwanthue (M. Jacomy).

### Electron microscopy

Scanning electron microscopy was conducted using a Phillips scanning electron microscope at 20 kV and a JCM-6000 bench-top scanning electron microscope at 10 kV.

### Phylogenetic analysis

We conducted phylogenetic analysis on a matrix of 59 taxa and 154 morphological characters (MorphoBank^51^ project 3927); 149 characters were drawn from previous studies^3,4,52–62^, and 5 further characters added to capture details pertinent to this study. All character formulations were updated to reflect the homology framework of ref. ^44^. Taxa were scored according to a conservative survey of published literature, following principles of best practice^63–65^.

Parsimony analysis used implied^66^ and equal weights, using the R^67^ package TreeSearch^68^ to conduct heuristic search with the parsimony ratchet^69^ using an approximate correction for inapplicable characters^64,70^. Convergence of tree search onto optimal trees was verified by inspecting the progress of tree search on two-dimensional mappings of the clustering information distance between trees^68,71,72^.

Bayesian analysis employed the Mk model^73^ with gamma-distributed rate variation across characters^73^, and a Dirichlet prior distribution on branch lengths^74,75^. We ran four runs of eight chains in MrBayes 3.2.7a^76^, discarding the first 100,000 generations as burn-in before sampling every 500th generation for 900,000 generations. Convergence was indicated by minimum estimated sample sizes >500 and potential scale reduction factors^77^ of 1.000 for all parameters. The information content of summary trees was maximized by omitting rogue taxa^78^.

Schematics in Fig. 5 follow data presented in refs. ^17,26,31,35–38,44,45,49,79–83^.

### Reporting summary

Further information on research design is available in the Nature Portfolio Reporting Summary linked to this article.

## Online content

Any methods, additional references, Nature Portfolio reporting summaries, source data, extended data, supplementary information, acknowledgements, peer review information; details of author contributions and competing interests; and statements of data and code availability are available at 10.1038/s41586-024-07756-8.

## Supplementary information

Supplementary Information This file contains supplementary notes and Supplementary Figs. 1–8, detailing scoring of characters and summary of phylogenetic results.

Reporting Summary

## Data Availability

Fossil material is accessioned at the Key Laboratory for Palaeobiology, Yunnan University, Kunming, China (YKLP). Scanning electron microscopy data, virtual dissections and raw XCT data are available at FigShare^84^. Phylogenetic data, results and scripts for analysis are available at MorphoBank^51^ (https://morphobank.org/permalink/?P3927).

## Code availability

Templates for our phylogenetic workflows are available at https://github.com/smithlabdurham/phylo-workflow and are archived at Zenodo^85^.
