# Supplementary material for: Organ systems of a Cambrian euarthropod larva
Source: Nature. 2024 Jul 31;633(8028):120–6. doi: 10.1038/s41586-024-07756-8 (PMC11374701; doi:10.1038/s41586-024-07756-8)
Supplement: Supplementary file 1 — This file contains supplementary notes and Supplementary Figs. 1–8, detailing scoring of characters and summary of phylogenetic results. [file 41586_2024_7756_MOESM1_ESM.docx]

Supplementary Information for: Organ systems of a Cambrian euarthropod larva

Martin R. Smith*, Emma J. Long*, Alavya Dhungana, Katherine J. Dobson, Jie Yang, Xiguang Zhang

Table of Contents

[Character definitions 1](#_Toc167192484)

[Summary of phylogenetic results 218](#_Toc167192485)

[Cluster analysis 223](#_Toc167192486)

[Tree space mapping 225](#_Toc167192487)

[References 229](#_Toc167192488)

# Character definitions

This report defines each character in the matrix ./mbank_X27231_2024-03-22-1117.nex, with a justification of the score for each taxon where relevant. The original matrix, the scripts used for analysis, and analytical results can be viewed interactively at [MorphoBank](https://morphobank.org/) (project [3927](https://morphobank.org/index.php/Projects/ProjectOverview/project_id/3927), matrix [27231](https://morphobank.org/index.php/Projects/Matrices/project_id/3927)). In this document, each character has been mapped onto an arbitrarily selected most parsimonious tree (with *k* = 10), with each tip labelled according to its character coding. These terminal states have been used to reconstruct the condition of each internal node, using the parsimony method of Brazeau *et al.* ([2019](#ref-Brazeau2019)) as implemented in the *R* package “TreeSearch” ([Smith 2023](#ref-Smith2023rj)). As different trees may give different reconstructions, these character mappings are illustrative, and do not represent definitive models of how any given character evolved.

## General organization

### [1] Paired appendages


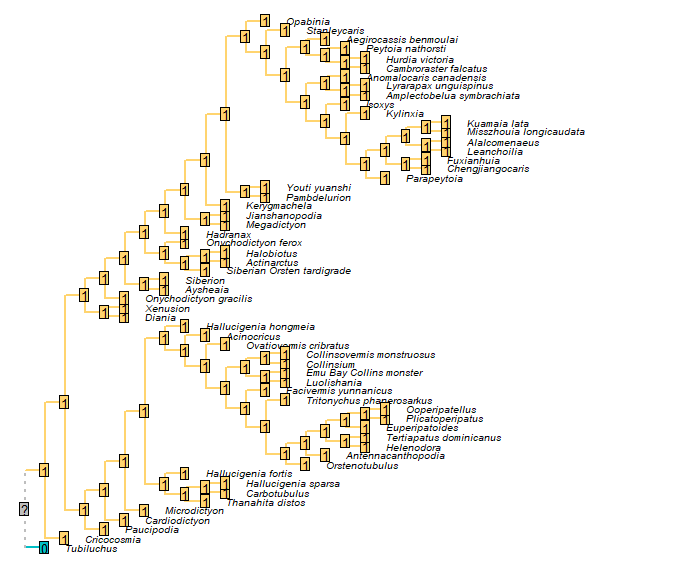


**Supplementary Figure 1: General organization: Paired appendages**

0 absent

1 present

Character 1 in Smith & Caron ([2015](#ref-Smith2015)) and Yang *et al.* ([2015](#ref-Yang2015)).

*Cricocosmia*: Ventral projections are treated as potential homologues to paired appendages ([Dhungana 2024](#ref-Dhungana2024)).

## Head region

### [2] Anterodorsal lobe


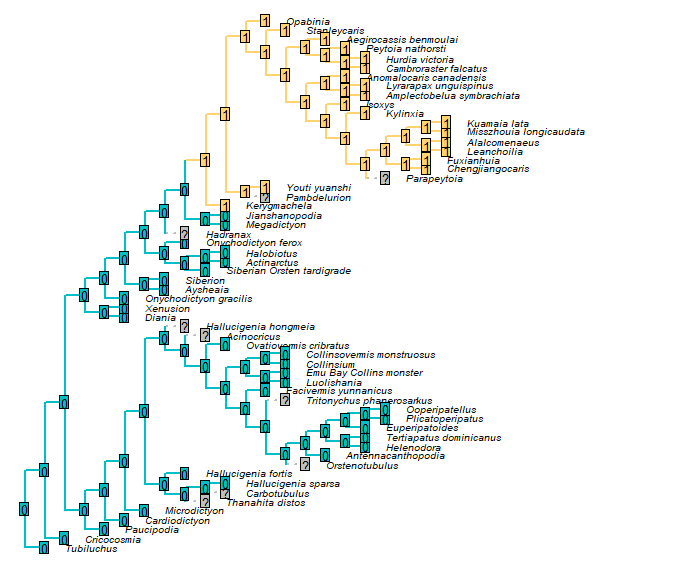


**Supplementary Figure 2: Head region: Anterodorsal lobe**

0 absent

1 present

The head of *Kerygmachela* has a dorsal protruding lobe that contains neural tissue ([Park *et al.* 2018](#ref-Park2018)), presumed homologous to the projection of YKLP 12387. This is distinct from the “swelling” of the anterior trunk in certain hallucigeniid lobopodians, which gives rise to a bulbous “head” region. In higher euarthropods, the anterior lobe may be covered by a dorsal sclerite.

*Kerygmachela*: Present ([Park *et al.* 2018](#ref-Park2018)).

*Kylinxia*: We interpret the sub-rectangular structure that is associated with the round anterior sclerite in *Kylinxia* ([O’Flynn *et al.* 2023](#ref-OFlynn2023cb)) as a potential homologue to the frontal organs of euarthropods that are associated with the anterior sclerite.

*Lyrarapax unguispinus*: Covered by dorsal sclerite.

*Pambdelurion*: Coded ambiguous as the character of the anterior protrusion between the appendages of *Pambdelurion* (e.g. [Vinther *et al.* 2016 fig. 1](#ref-Vinther2016)) is uncertain: this may be a manifestation of the oral apparatus, or may be a *Kerygmachela*-like lobe, as perhaps suggested by the anterior-directed filaments ([Vinther *et al.* 2016 fig. 3](#ref-Vinther2016)), which conceivably correspond to dorsal cirri.

*Youti yuanshi*: Present (this study).

### [3] Anterior region covered by sclerites


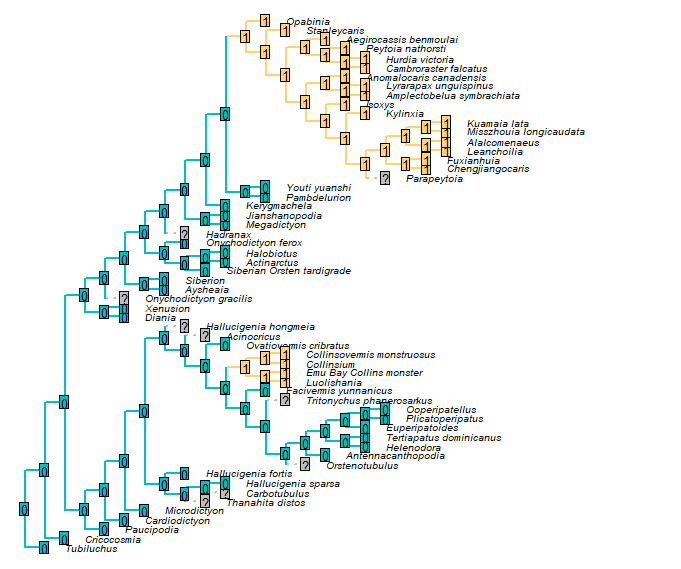


**Supplementary Figure 3: Head region: Anterior region covered by sclerites**

0 absent

1 present

Numerous lobopodians have been considered to have cephalic sclerites (see [Ma *et al.* 2014*a*](#ref-Ma2014jsp), char. 37), but in some cases this interpretation requires revision or confirmation through new material. Following Liu & Dunlop ([2014](#ref-Liu2014ppp)), we score this character as absent in *Hallucigenia* *fortis* (contra [Hou & Bergström 1995](#ref-Hou1995zjls)), *Onychodictyon* *ferox* (contra [Ou *et al.* 2012](#ref-Ou2012)) and *Cardiodictyon* (see [Hou & Bergström 1995](#ref-Hou1995zjls)). It is coded as ambiguous in *Onychodictyon* *gracilis* ([Liu *et al.* 2008*b*](#ref-Liu2008app)) and *Hallucigenia* *hongmeia* ([Steiner *et al.* 2012](#ref-Steiner2012)), as well as *Luolishania* (following [Smith & Ortega-Hernández 2014](#ref-Smith2014)). Fossil taxa with an incomplete anterior region are coded as uncertain.

Character 2 in Smith & Caron ([2015](#ref-Smith2015)) and Yang *et al.* ([2015](#ref-Yang2015)).

*Actinarctus*: Coded as absent as a single dorsal sclerite covers the entire body; this structure does not seem to correspond directly to the anterior sclerites of other taxa ([Boesgaard & Kristensen 2001](#ref-Boesgaard2001)).

*Amplectobelua symbrachiata*: Central oval head shield present ([Cong *et al.* 2017](#ref-Cong2017)).

*Facivermis yunnanicus*: Head sclerite absent ([Howard *et al.* 2020](#ref-Howard2020)).

*Onychodictyon gracilis*: Liu *et al.* ([2008*b*](#ref-Liu2008app)), in fig 2A4–5, suggest that the anterior region is sclerotized, although preservation shows irregular margins therefore more specimens are needed to confirm presence.

*Opabinia*: Present ([Dhungana & Smith 2021](#ref-Dhungana2021)).

*Ovatiovermis cribratus*: Absent ([Caron & Aria 2017](#ref-Caron2017)).

*Youti yuanshi*: No evidence of incipient sclerotization (this study).

### [4] Head shield (cephalic shield) formed by fused cephalic segments


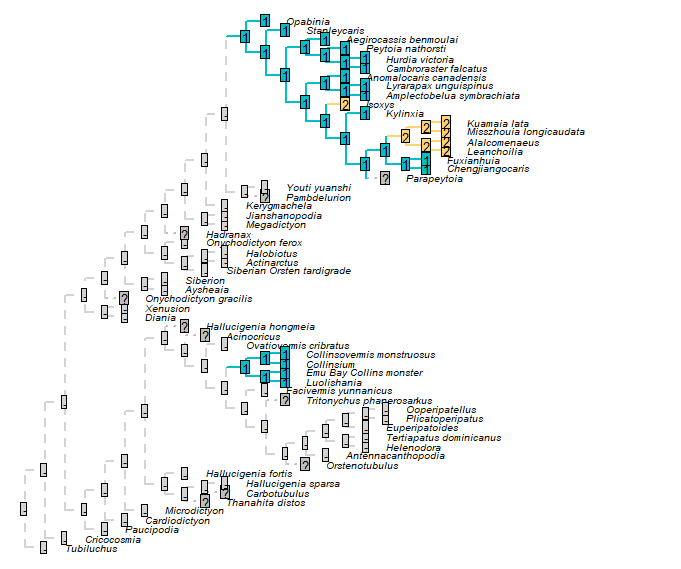


**Supplementary Figure 4: Head region: Head shield (cephalic shield) formed by fused cephalic segments**

- Inapplicable

1 absent

2 present

We score this character as absent for fuxianhuiids, because the cephalic shield is not derived from fused segments ([Chen *et al.* 1995*c*](#ref-Chen1995s); [Waloszek *et al.* 2005](#ref-Waloszek2005); [Bergström *et al.* 2008](#ref-Bergstrom2008); [Yang *et al.* 2013](#ref-Yang2013)), and in anomalocaridids, because the carapace-like structure on the head seems not to cover multiple cephalic segments (e.g. [Daley *et al.* 2009](#ref-Daley2009); [Daley & Edgecombe 2014](#ref-Daley2014)).

Character 3 in Smith & Caron ([2015](#ref-Smith2015)) and Yang *et al.* ([2015](#ref-Yang2015)).

### [5] Shape


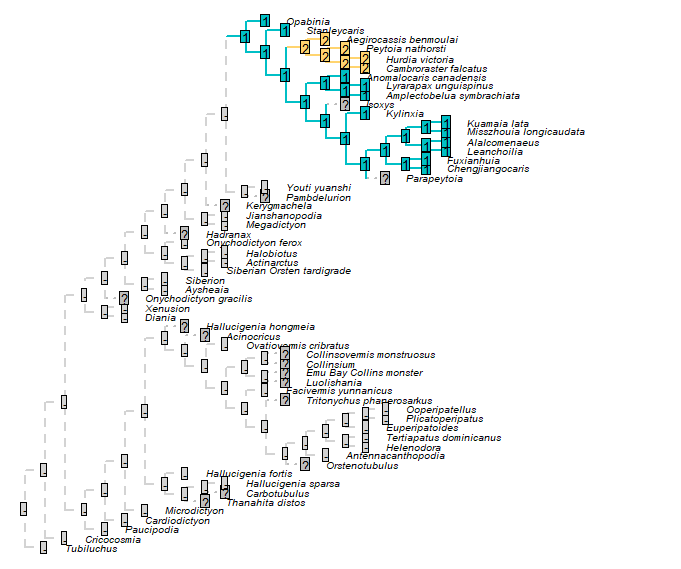


**Supplementary Figure 5: Head region: Dorsal isolated sclerite: Shape**

- Inapplicable

1 oval/rounded

2 elongate

Character adapted from 59 in Van Roy *et al.* ([2015](#ref-VanRoy2015)). Character 5 in Yang *et al.* ([2015](#ref-Yang2015)).

*Amplectobelua symbrachiata*: Oval ([Cong *et al.* 2017](#ref-Cong2017)).

### [6] Position


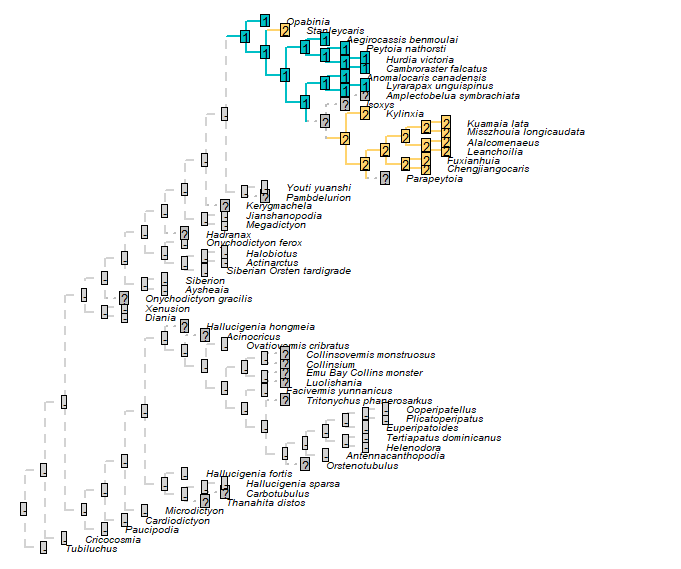


**Supplementary Figure 6: Head region: Dorsal isolated sclerite: Position**

- Inapplicable

1 dorsal

2 anterior

This character represents the hypothetical change in position of the anterior sclerite of the upper-stem euarthropods and the dorsal head sclerite of the anomalocaridids that are associated with protocerebral structures (see [Budd 2021](#ref-Budd2021)).

*Opabinia*: Dorsal sclerite present ([Dhungana & Smith 2021](#ref-Dhungana2021)).

*Stanleycaris*: Prominently anterior ([Moysiuk & Caron 2022](#ref-Moysiuk2022)).

### [7] Reticulate ornament


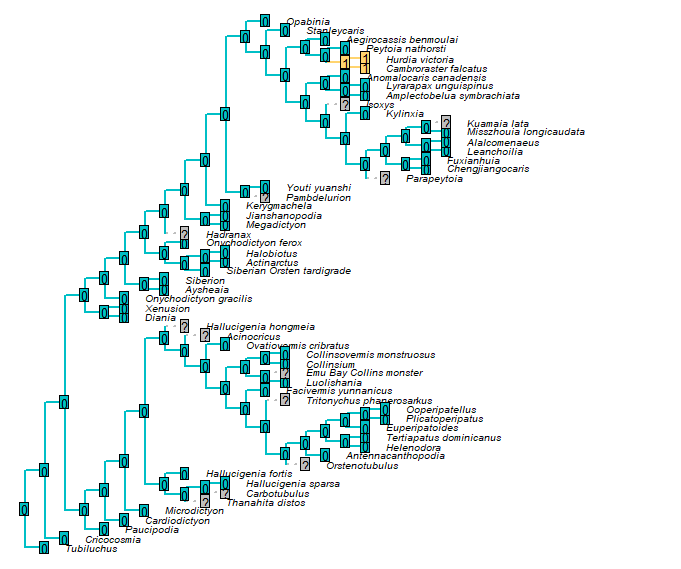


**Supplementary Figure 7: Head region: Dorsal isolated sclerite: Reticulate ornament**

0 absent

1 present

The head sclerites of certain hurdiids exhibit a conspicuous reticulate ornamentation.

Character 26 in Moysiuk & Caron ([2019](#ref-Moysiuk2019)).

*Amplectobelua symbrachiata*, *Anomalocaris canadensis*, *Cambroraster falcatus*, *Hurdia victoria*, *Peytoia nathorsti*, *Aegirocassis benmoulai*, *Lyrarapax unguispinus*: Following Moysiuk & Caron ([2019](#ref-Moysiuk2019)).

### [8] Degree of attachment of dorsal isolated sclerite on head


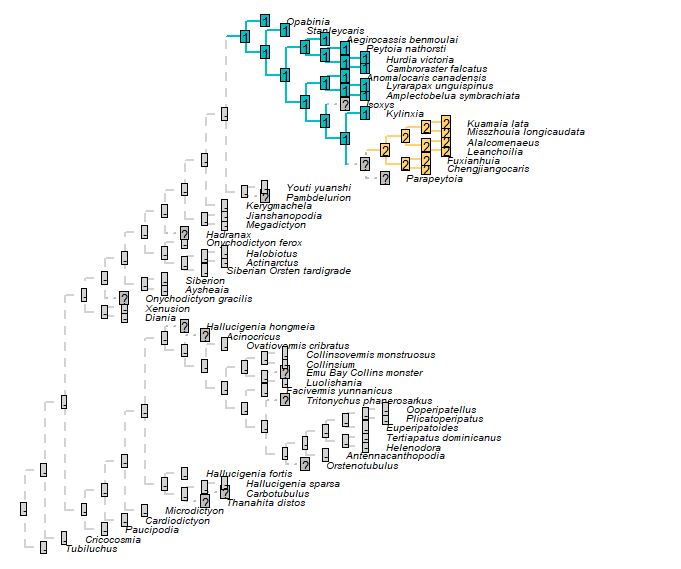


**Supplementary Figure 8: Head region: Degree of attachment of dorsal isolated sclerite on head**

- Inapplicable

1 broad attachment to cephalic region

2 narrow attachment to anterior edge of cephalic region

The dorsal sclerite is attached broadly in Radiodonta ([Daley *et al.* 2009](#ref-Daley2009); [Daley & Bergström 2012](#ref-Daley2012); [Cong *et al.* 2014](#ref-Cong2014); [Daley & Edgecombe 2014](#ref-Daley2014); [Van Roy *et al.* 2015](#ref-VanRoy2015)), whereas the euarthropod anterior sclerite is only narrowly attached to the anterior end of the body in upper-stem and crown-group euarthropods ([Edgecombe & Ramsköld 1999](#ref-Edgecombe1999); [Budd 2008](#ref-Budd2008); [Yang *et al.* 2013](#ref-Yang2013); [Ortega-Hernández 2015](#ref-Ortega2015)).

Character 6 in Yang *et al.* ([2015](#ref-Yang2015)).

### [9] Isolated lateral sclerites, forming tripartite carapace


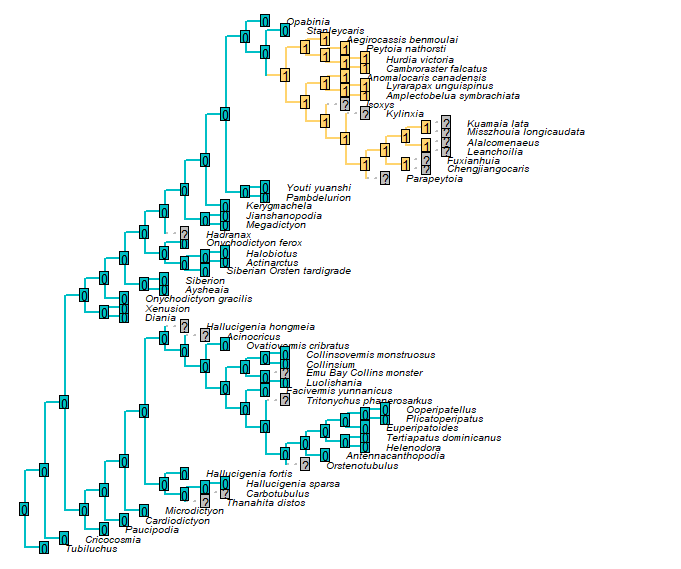


**Supplementary Figure 9: Head region: Isolated lateral sclerites, forming tripartite carapace**

0 absent

1 present

This character refers to the lateral “P” elements that typify the anterior scleritome of hurdiid radiodontans ([Daley *et al.* 2009](#ref-Daley2009); [Daley & Bergström 2012](#ref-Daley2012); [Van Roy *et al.* 2015](#ref-VanRoy2015)).

Character 7 in Yang *et al.* ([2015](#ref-Yang2015)).

*Amplectobelua symbrachiata*: Prominent ovoid structures adjacent to the frontal appendage are interpreted as P-elements, connected by a rod ([Cong *et al.* 2017](#ref-Cong2017)).

*Anomalocaris canadensis*, *Peytoia nathorsti*, *Lyrarapax unguispinus*: Present ([Moysiuk & Caron 2019](#ref-Moysiuk2019)).

*Pambdelurion*: No indication of lateral sclerites in Young & Vinther ([2017](#ref-Young2017)) or Budd ([1998*a*](#ref-Budd1998ar)).

### [10] Shape


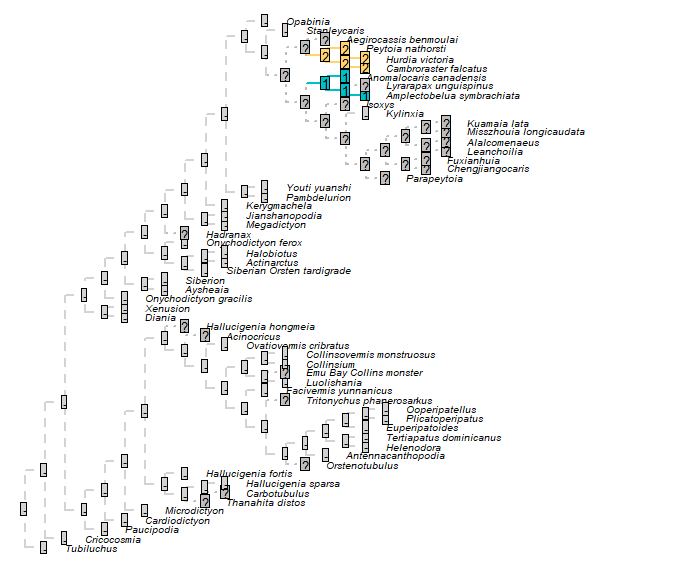


**Supplementary Figure 10: Head region: Isolated lateral sclerites: Shape**

- Inapplicable

1 subcircular

2 elongate

Character 30 in Moysiuk & Caron ([2019](#ref-Moysiuk2019)). The lateral sclerites of certain hurdiids are elongate, whereas those of anomalocaridids are more circular in aspect and shape.

*Aegirocassis benmoulai*: The dorsal sclerite of *Aegirocassis* ([Van Roy *et al.* 2015](#ref-VanRoy2015)) is neither resembles the cub-circular *Anomalocaris*-type sclerite nor as elongate as e.g., *Hurdia*. Therefore we code this character as ambiguous.

*Amplectobelua symbrachiata*, *Anomalocaris canadensis*, *Cambroraster falcatus*, *Hurdia victoria*, *Peytoia nathorsti*, *Lyrarapax unguispinus*: Following Moysiuk & Caron ([2019](#ref-Moysiuk2019)).

### [11] Ventral isolated sclerite


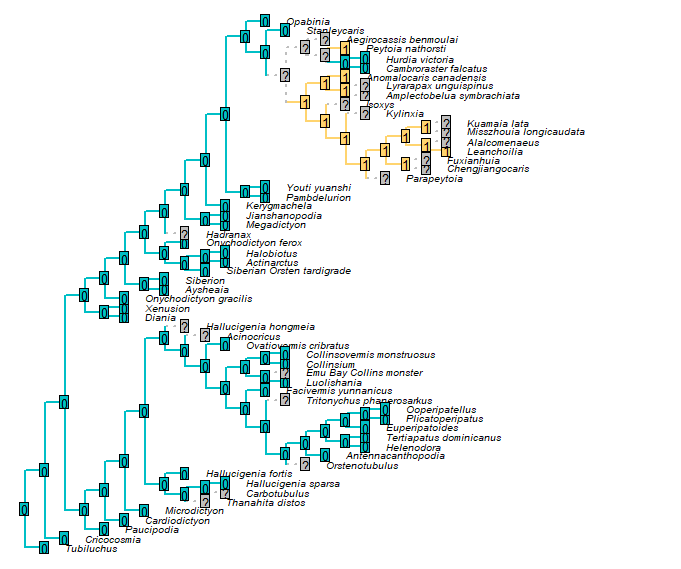


**Supplementary Figure 11: Head region: Ventral isolated sclerite**

0 absent

1 present

Character formulated from possible homology of ventral sclerites in §7 of Budd ([2021](#ref-Budd2021)).

*Amplectobelua symbrachiata*, *Anomalocaris canadensis*, *Hurdia victoria*, *Peytoia nathorsti*, *Leanchoilia*: Following Budd ([2021](#ref-Budd2021)).

*Cambroraster falcatus*: No intermediate plate between p-elements observed ([Moysiuk & Caron 2019](#ref-Moysiuk2019)).

### [12] Anterior trunk flexure in coronal plane


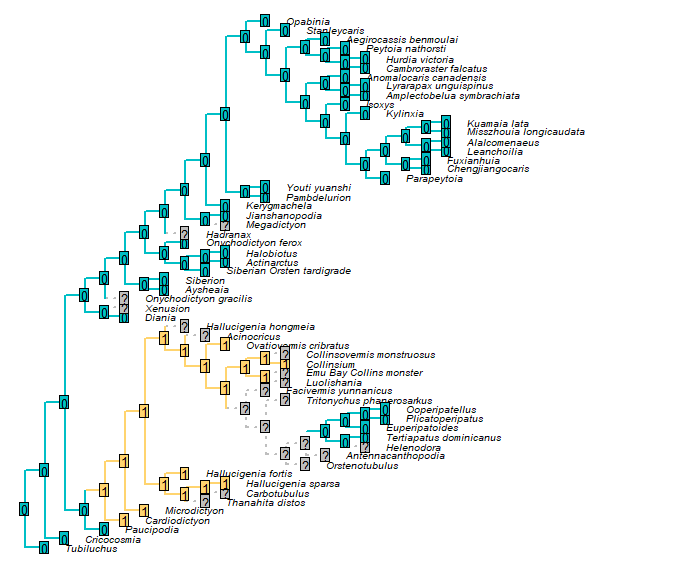


**Supplementary Figure 12: Head region: Anterior trunk flexure in coronal plane**

0 orientation of mouth is fixed relative to main trunk

1 flexible anterior trunk allowing mouth’s dorsal-ventral orientation to be independent of main trunk axis

The terminal mouths of *Hallucigenia* *sparsa* ([Smith & Caron 2015](#ref-Smith2015)), *H. fortis* ([Liu & Dunlop 2014](#ref-Liu2014ppp)), *Collinsium* ([Yang *et al.* 2015](#ref-Yang2015)), *Microdictyon* and *Cardiodictyon* ([Chen *et al.* 1995*a*](#ref-Chen1995bnmns); [Liu & Dunlop 2014](#ref-Liu2014ppp)) are consistently oriented ventrally, perpendicular to the main trunk axis; the anteriormost trunk (or, colloquially, ‘head’) can be manoeuvred independently of the main trunk. In other taxa (e.g. priapulans), the orientation of the mouth is fixed relative to the main trunk.

Character 5 in Smith & Caron ([2015](#ref-Smith2015)).

*Ovatiovermis cribratus*: Interpreted as flexible ([Caron & Aria 2017](#ref-Caron2017)).

### [13] Swelling of anteriormost trunk


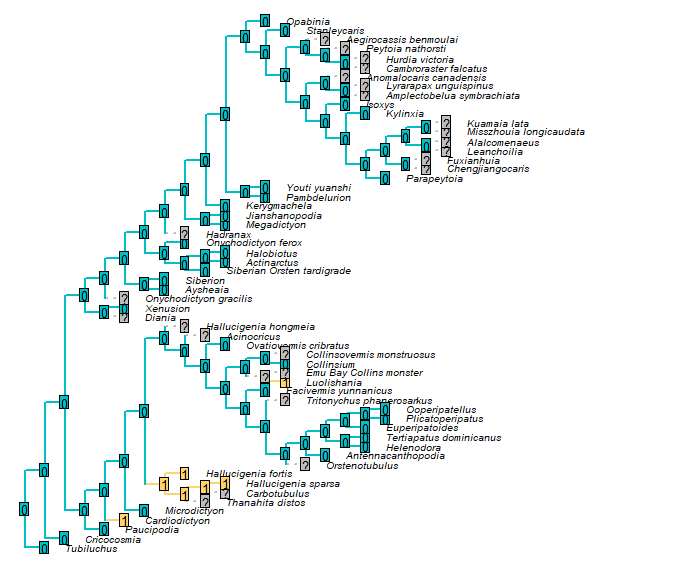


**Supplementary Figure 13: Head region: Swelling of anteriormost trunk**

0 anteriormost trunk contiguous with posterior trunk; no swollen ‘head’

1 anteriormost trunk elliptical, substantially wider than adjacent trunk

Certain lobopodians (*Cardiodictyon*, *Hallucigenia* *fortis*, *Luolishania*) have a differentiated anteriormost trunk that forms a wide ellipse or “head” ([Liu & Dunlop 2014](#ref-Liu2014ppp)). In *Hallucigenia* *sparsa*, the “head” is denoted by a slight increase in the width of the anteriormost trunk, which is most prominent in smaller specimens ([Smith & Caron 2015](#ref-Smith2015)). In other taxa (*Aysheaia*, *Onychodictyon* *ferox*, *Megadictyon*, *Jianshanopodia*, *Ilyodes*, *Collinsium*), the anteriormost trunk is not differentiated in this way ([Thompson & Jones 1980](#ref-Thompson1980); [Ou *et al.* 2012](#ref-Ou2012); [Vannier *et al.* 2014](#ref-Vannier2014); [Yang *et al.* 2015](#ref-Yang2015)). Coded as ambiguous in euarthropods, where the “trunk” has been replaced by sclerotized segments.

Character 6 in Smith & Caron ([2015](#ref-Smith2015)).

*Diania*: Ou & Mayer ([2018](#ref-Ou2018)) describe a swollen anterior ‘helmet like structure’.

*Facivermis yunnanicus*: No swelling ([Howard *et al.* 2020](#ref-Howard2020)).

### [14] Paired anterior projections


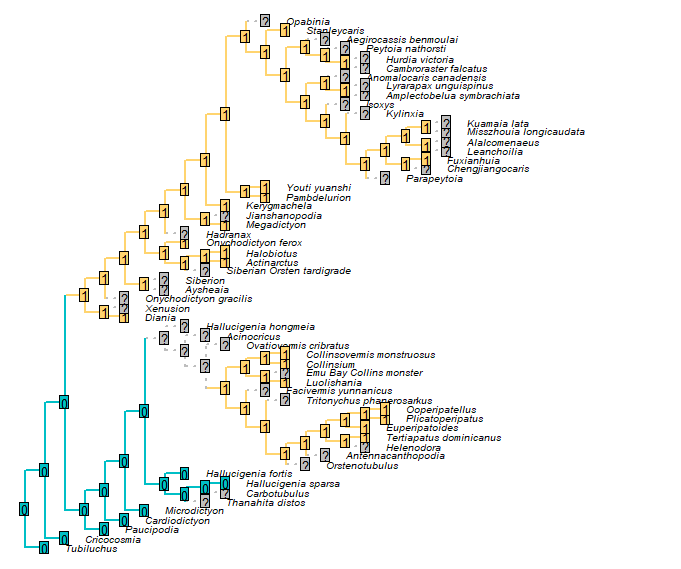


**Supplementary Figure 14: Head region: Paired anterior projections**

0 absent

1 present

Budd ([2021](#ref-Budd2021)) argues that anterior projections in certain lobopodians and tardigrades are homologous to euarthropod frontal filaments. We additionally interpret the anterior projections of *Pambdelurion* and *Megadictyon* as potential homologues.

Potential homologues to the frontal filaments – the frontal processes, which migrate to become the anteriormost pair of lip papillae in adults – are present in crown group Onychophora ([Ortega-Hernández & Budd 2016](#ref-Ortega2016asd)). On this view, we interpret the dorsal, apparently non-appendicular, antenniform appendages of *Collinsovermis* and *Luolishania* as potential homologues to the frontal filaments.

Adapted from character 95 in Yang *et al.* ([2016](#ref-Yang2016)).

*Leanchoilia*, *Alalcomenaeus*: Considered ambiguous in megacheirans by Ortega-Hernández & Budd ([2016](#ref-Ortega2016asd)).

*Anomalocaris canadensis*: Coded ambiguous; although the head is known from many articulated specimens ([Daley & Edgecombe 2014](#ref-Daley2014)), the disposition of large sclerotized head elements leaves the absence of cirri difficult to conclusively demonstrate.

*Antennacanthopodia*: The possibility that one set of antenniform appendages corresponds to enlarged frontal filaments is enticing but difficult to test.

*Aysheaia*: Coded ambiguous, as the papillae surrounding the mouth are plausible, if unconvincing, homologues.

*Cambroraster falcatus*: Ambiguous; head obscured by carapaces ([Moysiuk & Caron 2019](#ref-Moysiuk2019)).

*Chengjiangocaris*, *Misszhouia longicaudata*, *Kuamaia lata*: Coded ambiguous, as anterior region comprises sclerotized segments.

*Collinsovermis monstruosus*: Present ([Caron & Aria 2020](#ref-Caron2020)).

*Diania*: Potentially represented by the “head spines” ([Ou & Mayer 2018](#ref-Ou2018)).

*Facivermis yunnanicus*: Ambiguous ([Howard *et al.* 2020](#ref-Howard2020)).

*Fuxianhuia*: Frontal filaments present ([Budd 2021](#ref-Budd2021)).

*Halobiotus*: Coded as present based on innervation data that suggests that heterotardigrade anterior cephalic structures are homologous to sensory fields in eutardigrades ([Gross *et al.* 2021](#ref-Gross2021)).

*Hurdia victoria*: Ambiguous; head obscured by carapaces ([Daley *et al.* 2013*a*](#ref-Daley2013jsp)).

*Siberion*, *Xenusion*, *Jianshanopodia*: Inadequately preserved for confident scoring.

*Kerygmachela*: Interpreted as present by Ortega-Hernández & Budd ([2016](#ref-Ortega2016asd)). See rostral spines in supplementary figure 8 from Park *et al.* ([2018](#ref-Park2018)).

*Kylinxia*: Difficult to demonstrate absence based on available material ([Zeng *et al.* 2020](#ref-Zeng2020)).

*Lyrarapax unguispinus*: Considered ambiguous due to position of head sclerite and difficulty in interpreting head outline in available material ([Cong *et al.* 2014](#ref-Cong2014), [2016](#ref-Cong2016); [Liu *et al.* 2018](#ref-Liu2018nsr)).

*Megadictyon*: See Figure 1e from Vannier *et al.* ([2014](#ref-Vannier2014)).

*Onychodictyon ferox*: Unannulated, narrow antenniform structures ([Liu *et al.* 2008*b* fig. 3D](#ref-Liu2008app)) are interpreted as potential homologues.

*Onychodictyon gracilis*: Ambiguous: although described as absent by Liu *et al.* ([2008*b*](#ref-Liu2008app)), we do not consider the limited available material sufficient to definitively rule out the presence of these features.

*Ovatiovermis cribratus*: Figure 1H from Caron & Aria ([2017](#ref-Caron2017)) shows a possible anterior projection. More detailed head anatomy is needed to be certain of this feature.

*Pambdelurion*: Interpreted as present by Ortega-Hernández & Budd ([2016](#ref-Ortega2016asd)).

*Peytoia nathorsti*: Ambiguities in head region ([Budd 2021](#ref-Budd2021)) mean the absence of these features cannot be determined with confidence.

*Stanleycaris*: We consider the structures interpreted as “filament-like anterior nerves” ([Moysiuk & Caron 2022](#ref-Moysiuk2022), e.g. fig. 3a) as potential homologues of the frontal filaments.

*Youti yuanshi*: Dorsal filaments treated as potential homologues (this study).

### [15] Incorporated into lips


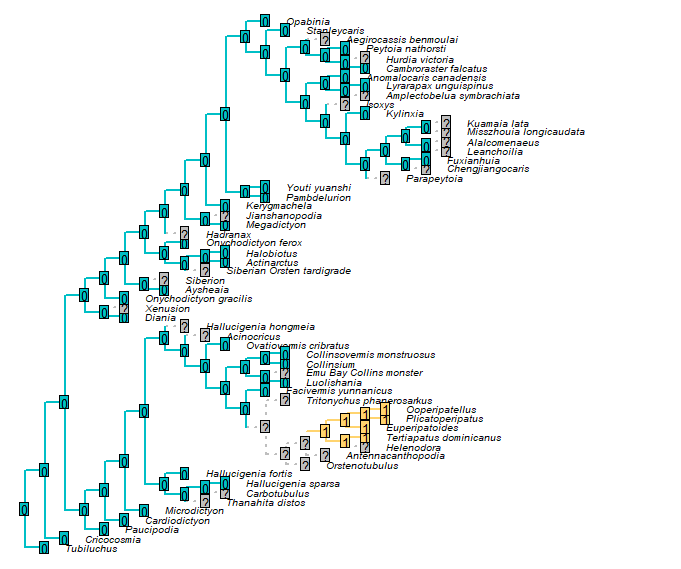


**Supplementary Figure 15: Head region: Paired anterior projections: Incorporated into lips**

0 Frontal filaments not incorporated into lip papillae

1 Incorporated into lip papillae

Potential homologues to the frontal filaments – the frontal processes – migrate to become the anteriormost pair of lip papillae in adult Onychophora ([Ortega-Hernández & Budd 2016](#ref-Ortega2016asd)). As the migration is a derived state, this character is treated as neomorphic.

## Oral structures

### [16] Mouth opening position


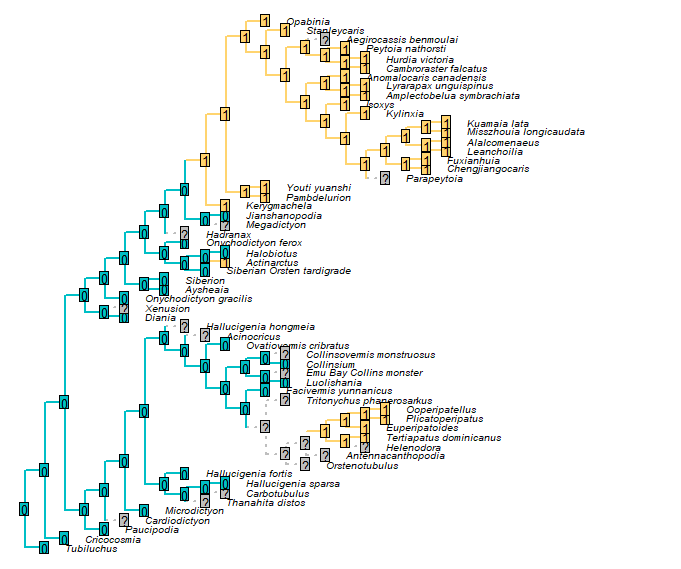


**Supplementary Figure 16: Oral structures: Mouth opening position**

0 terminal

1 ventral

This character identifies the hypothesized evolutionary event of a movement of the mouth position. In Euarthropods the mouth is in a ventral position. In certain lobopodians, including *Collinsium*, hallucigeniids and *Luolishania*, the terminal mouth can superficially appear ventral due to the flexure of the neck ([Ma *et al.* 2009](#ref-Ma2009); [Smith & Caron 2015](#ref-Smith2015); [Yang *et al.* 2015](#ref-Yang2015)).

This character captures the transformation envisaged by characters 23 and 24 in Yang et al. (2015): both these characters appear to code for the same event of movement of mouth position, leading to a rotation in the head area, with appendages moving to an anterior position relative to the mouth.

*Actinarctus*: Ventral in Heterotardigrada ([Persson *et al.* 2014](#ref-Persson2014)).

*Amplectobelua symbrachiata*: Ventral ([Cong *et al.* 2017](#ref-Cong2017)).

*Antennacanthopodia*: *Antennacanthopodia* is coded as ambiguous as there is no direct evidence for the location of the mouth ([Ou *et al.* 2011](#ref-Ou2011)).

*Aysheaia*: Terminal ([Whittington 1978](#ref-Whittington1978)).

*Facivermis yunnanicus*: Terminal ([Howard *et al.* 2020](#ref-Howard2020)).

*Halobiotus*: Mouth is terminal and anterior in Eutardigrada (e.g. Halberg *et al.* ([2009](#ref-Halberg2009)); Persson *et al.* ([2012](#ref-Persson2012))).

*Kerygmachela*: Park *et al.* ([2018](#ref-Park2018)) interpret a ventral position, contra Budd ([1993](#ref-Budd1993)); Budd ([1998*b*](#ref-Budd1998trse)).

*Megadictyon*: Liu *et al.* ([2007](#ref-Liu2007az)) suggest a ventral location, although this could be due to compaction, therefore we code this as uncertain.

*Onychodictyon ferox*: Following Ou *et al.* ([2012](#ref-Ou2012)).

*Pambdelurion*: The mouth opening is ventrally oriented in *Pambdelurion* ([Budd 1998*a*](#ref-Budd1998ar)).

### [17] Ventral mouth orientation


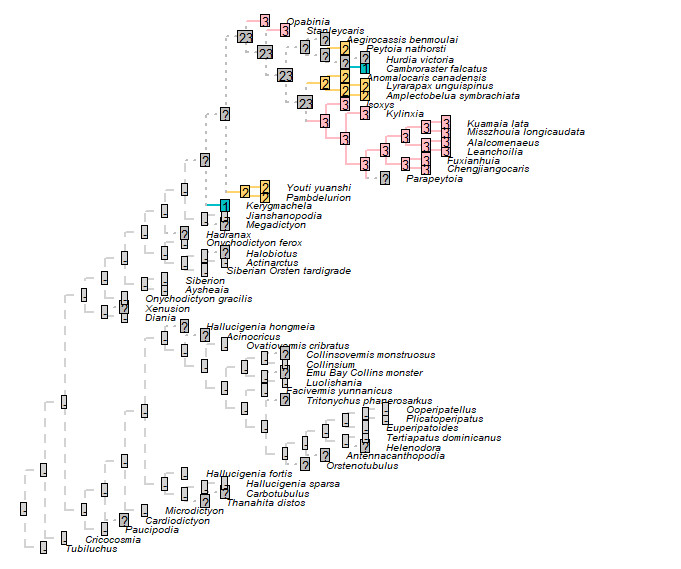


**Supplementary Figure 17: Oral structures: Ventral mouth orientation**

- Inapplicable

1 anterior

2 ventral

3 posterior

When the mouth is anterior and terminal, mouth orientation is fixed as it can only face anteriorly. However, if the mouth is in a ventral position, then it can face anteriorly (following the interpretation of *Kerygmachela* by [Park *et al.* 2018](#ref-Park2018)), ventrally (anomalocaridids) or posteriorly (crown euarthropods, opabiniids). This character is only applicable when mouth is ventral.

Adapted from character 11 in Zhang *et al.* ([2016](#ref-Zhang2016)) [SC: 7; Y: 23].

*Cambroraster falcatus*: Coded as anterior: figure 2J from Moysiuk & Caron ([2019](#ref-Moysiuk2019)) shows that the mouth faces anteriorly, in contrast to the ventral facing mouth of e.g., *Anomalocaris* (see figures 5 and 8 from [Daley & Edgecombe 2014](#ref-Daley2014)). Therefore we code as anterior.

*Hurdia victoria*: *Hurdia* specimens are often markedly disarticulated, and therefore difficult to code orientation of circumoral elements ([Daley *et al.* 2013*a*](#ref-Daley2013jsp)). We conservatively code *Hurdia* ambiguous as there are no specimens in a ventral position to determine mouth orientation (supplementary data in [Daley *et al.* 2009](#ref-Daley2009)).

*Kerygmachela*: Park *et al.* ([2018](#ref-Park2018)) interpret the mouth has moved to a ventral position, yet faces anteriad, therefore has not been rotated to point posteriad.

*Lyrarapax unguispinus*: Cong *et al.* ([2014](#ref-Cong2014)) interpret a ventral-facing mouth.

*Megadictyon*: Unclear mouth position, however mouth clearly not rotated to posteriad ([Liu *et al.* 2007](#ref-Liu2007az)).

*Peytoia nathorsti*: Coded as ventral, per the two specimens in figure 3 of Budd ([2021](#ref-Budd2021)).

### [18] Pre-oral chamber


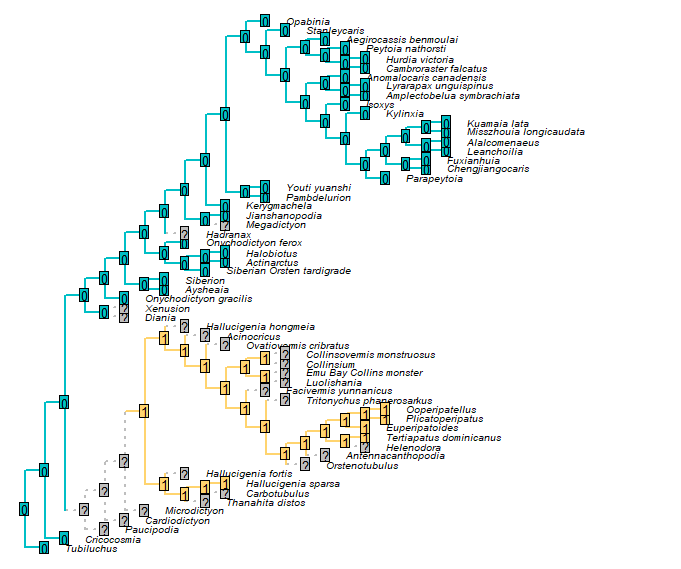


**Supplementary Figure 18: Oral structures: Pre-oral chamber**

0 absent

1 present

We define a pre-oral (‘buccal’) chamber as a region enclosing the mouth and formed by the outgrowth of surrounding body tissue – as observed in modern onychophorans ([Martin & Mayer 2014](#ref-Martin2014)). Taking the circumoral structures of *Hallucigenia* *sparsa* to represent the position of the true mouth, the mouth is located in a chamber within the anteriormost trunk (i.e. ‘head’). The character is coded as absent where the oral region is clearly displayed externally, as in Tardigrada, euarthropods from *Kerygmachela* crownwards (where the position of the mouth is marked by the expression of an ‘oral cone’), *Jianshanopodia* ([Vannier *et al.* 2014](#ref-Vannier2014)), *Aysheaia* ([Whittington 1978](#ref-Whittington1978)), and *Siberion* ([Dzik 2011](#ref-Dzik2011)). It is coded as ambiguous in other taxa, as the location of the original mouth is unclear.

Character 8 in Smith & Caron ([2015](#ref-Smith2015)).

### [19] Radially arranged circumoral structures


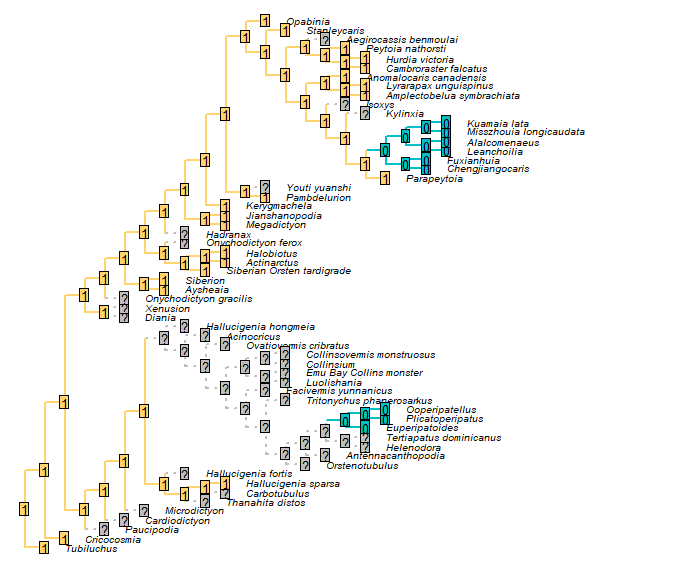


**Supplementary Figure 19: Oral structures: Radially arranged circumoral structures**

0 absent

1 present

The mouth of many ecdysozoans is surrounded by radially-arranged cuticular or sclerotized structures. In priapulans and other cycloneuralians these are typically conical spines, arranged centripetally when the pharynx is contracted and centrifugally when the pharynx is everted ([Conway Morris 1977](#ref-ConwayMorris1977)). In basal panarthropods such as *Aysheaia*, *Kerygmachela*, *Hallucigenia* and *Jianshanopodia*, the structures are regular non-sclerotized lamellae, aciculae or plates ([Whittington 1978](#ref-Whittington1978); [Budd 1998*b*](#ref-Budd1998trse); [Vannier *et al.* 2014](#ref-Vannier2014); [Smith & Caron 2015](#ref-Smith2015)); among anomalocaridids the plates are sclerotized and differentiated ([Daley & Bergström 2012](#ref-Daley2012); [Daley *et al.* 2013*b*](#ref-Daley2013p), [*a*](#ref-Daley2013jsp)). (Although the three or four prominent plates in the anomalocaridid oral cone are differentiated to give rise to bilateral symmetry ([Daley & Bergström 2012](#ref-Daley2012)), the underlying radial arrangement of these plates is clear: some plates straddle the midline, and if rotated by 90° the smaller plates are equivalent to their counterparts.) Tardigrades bear circumoral lamellae ([Budd 2001*a*](#ref-Budd2001za); [Guidetti *et al.* 2012](#ref-Guidetti2012); [Mayer *et al.* 2013*b*](#ref-Mayer2013po)). Other lobopodians are coded as ambiguous; euarthropods and onychophorans are coded as absent (discussed in [Smith & Caron 2015](#ref-Smith2015), char. 9).

Character 9 in Smith & Caron ([2015](#ref-Smith2015)); character 25 in Yang *et al.* ([2015](#ref-Yang2015)).

*Amplectobelua symbrachiata*: Smooth and tuberculate plates are interpreted as elements of an *Anomalocaris*-like oral cone ([Cong *et al.* 2017](#ref-Cong2017)).

*Antennacanthopodia*: *Antennacanthopodia* ([Ou *et al.* 2011](#ref-Ou2011)) is coded as ambiguous as there is no direct evidence for the location of the mouth.

*Facivermis yunnanicus*: Preservation inadequate to evaluate ([Howard *et al.* 2020](#ref-Howard2020)).

*Lyrarapax unguispinus*: Not described in original reports ([Cong *et al.* 2014](#ref-Cong2014), [2016](#ref-Cong2016), [2017](#ref-Cong2017)), but documented in a juvenile by Liu *et al.* ([2018](#ref-Liu2018nsr)), who propose that the absence in larger specimens is taphonomic.

*Megadictyon*: Present ([Vannier *et al.* 2014](#ref-Vannier2014), supplementary figure 6).

*Microdictyon*: The cuticular ring reported in the head of *Microdictyon* ([Liu & Dunlop 2014](#ref-Liu2014ppp)) requires detailed study before its interpretation can be considered secure.

*Opabinia*: Radial structures around the mouth drawn by Whittington ([1975](#ref-Whittington1975)) are interpreted by Dhungana & Smith ([2021](#ref-Dhungana2021)) as circumoral plates.

*Ovatiovermis cribratus*: Detailed arrangement of tooth-like structures compatible with arrangement in *Hallucigenia* ([Smith & Caron 2015](#ref-Smith2015)), but inadequately preserved to evaluate ([Caron & Aria 2017](#ref-Caron2017)). Coded as ambiguous.

*Youti yuanshi*: Uncertain, as oral surface is incompletely preserved (this study), and it is possible that such structures would become more prominent in adults.

### [20] Contact area


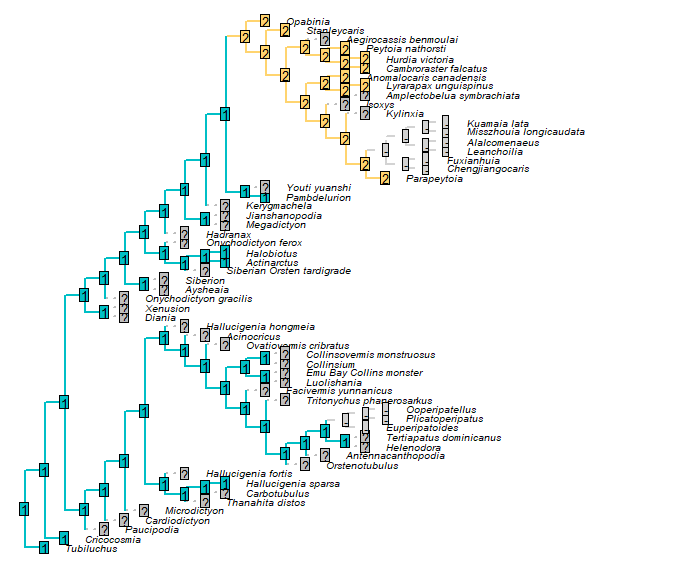


**Supplementary Figure 20: Oral structures: Radially arranged circumoral structures: Contact area**

- Inapplicable

1 small contact area (e.g. coronal spines)

2 large contact area (e.g. *Parapeytoia*)

This character differentiates circumoral structures with a small contact area with the body (e.g. coronal spines of priapulomorph worms) from the plate-like circumoral structures that have a large surface area in contact with the body – found commonly in stem euarthropods (e.g. *Parapeytoia*, *Hurdia*).

*Actinarctus*, *Halobiotus*: We code tardigrades as having a small contact area as the peribuccal lamellae are only basally attached to the body (e.g. [Guidetti *et al.* 2013 fig. 3B](#ref-Guidetti2013)).

*Megadictyon*: Due to the limited material we code this as ambiguous.

*Pambdelurion*: As reconstructed by Vinther *et al.* ([2016](#ref-Vinther2016)) (e.g. Figure 3D) the ‘triangular plates’ are only basally in contact with the body, therefore we code this as a small contact area.

### [21] Differentiated circumoral structures


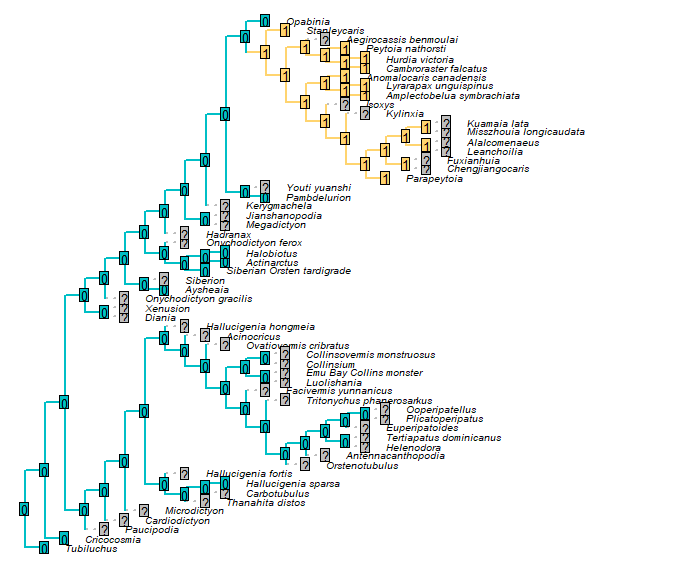


**Supplementary Figure 21: Oral structures: Differentiated circumoral structures**

0 undifferentiated (e.g. *Pambdelurion* plates)

1 differentiated (i.e. Radiodonta – three or four enlarged plates)

This character distinguishes the simple organization of the mouth apparatus in *Hallucigenia* ([Smith & Caron 2015](#ref-Smith2015)) and *Pambdelurion* ([Budd 1998*a*](#ref-Budd1998ar); [Vinther *et al.* 2016](#ref-Vinther2016)) from the more complex mouthparts of anomalocaridids ([Daley *et al.* 2009](#ref-Daley2009); [Daley & Bergström 2012](#ref-Daley2012); [Daley & Edgecombe 2014](#ref-Daley2014)). We score *Megadictyon*, *Schinderhannes* and *Jianshanopodia* as uncertain to reflect their mouthparts” poor preservation ([Liu *et al.* 2006](#ref-Liu2006), [2007](#ref-Liu2007az); [Kühl *et al.* 2009](#ref-Kuhl2009)). The character is treated as neomorphic to reflect the increasing gradient of complexity reflected by differentiation.

Character 10 in Smith & Caron ([2015](#ref-Smith2015)).

*Amplectobelua symbrachiata*: Three or possibly four tuberculate plates ([Cong *et al.* 2017](#ref-Cong2017)).

*Cambroraster falcatus*: Moysiuk & Caron ([2019](#ref-Moysiuk2019)).

*Lyrarapax unguispinus*: Four enlarged plates ([Liu *et al.* 2018](#ref-Liu2018nsr)).

*Opabinia*: Undifferentiated elongate plates ([Dhungana & Smith 2021](#ref-Dhungana2021)).

*Parapeytoia*: Differentiated, following Budd ([2021](#ref-Budd2021)).

### [22] Enlarged plates


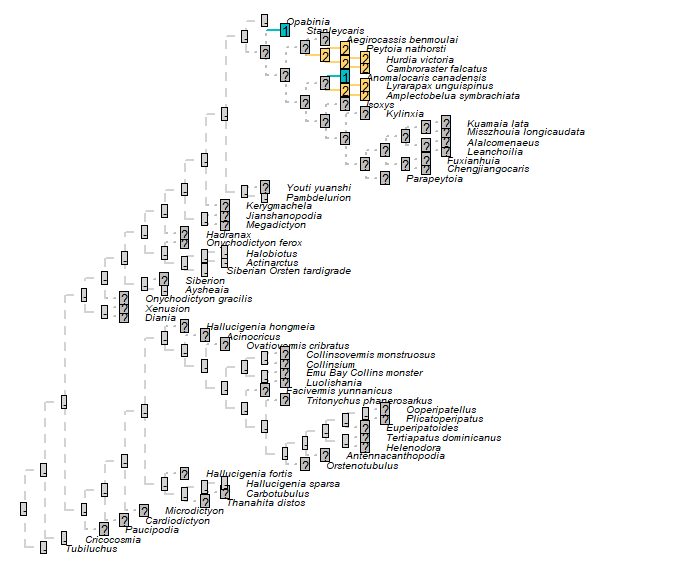


**Supplementary Figure 22: Oral structures: Differentiated circumoral structures: Enlarged plates**

- Inapplicable

1 3 enlarged plates

2 4 enlarged plates

### [23] Nodes on differentiated circumoral structures


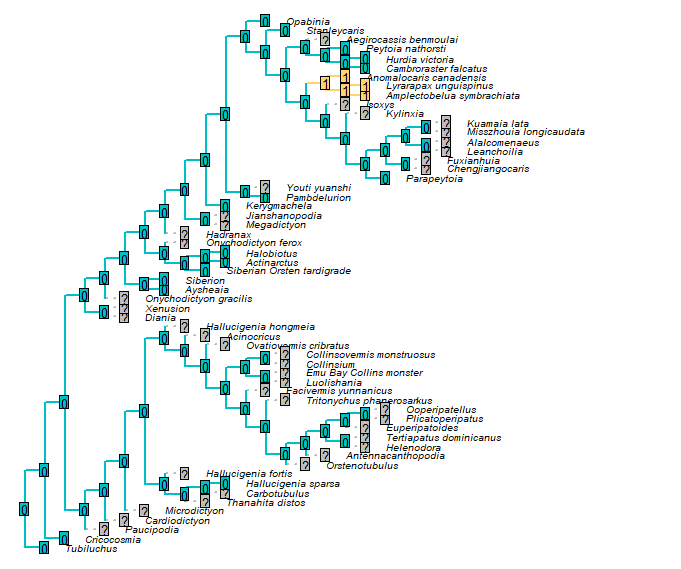


**Supplementary Figure 23: Oral structures: Nodes on differentiated circumoral structures**

0 absent

1 present

[NEW]
Node-bearing plates are considered by Liu *et al.* ([2018](#ref-Liu2018nsr)) to represent a synapomorphy of Anomalocarididae + Amplectobeluidae.

*Amplectobelua symbrachiata*: Tubercles ([Cong *et al.* 2017](#ref-Cong2017)).

*Cambroraster falcatus*: Not evident or interpreted as present by Moysiuk & Caron ([2019](#ref-Moysiuk2019)).

*Lyrarapax unguispinus*: Present ([Liu *et al.* 2018](#ref-Liu2018nsr)).

### [24] Furrowed folds on circumoral structures


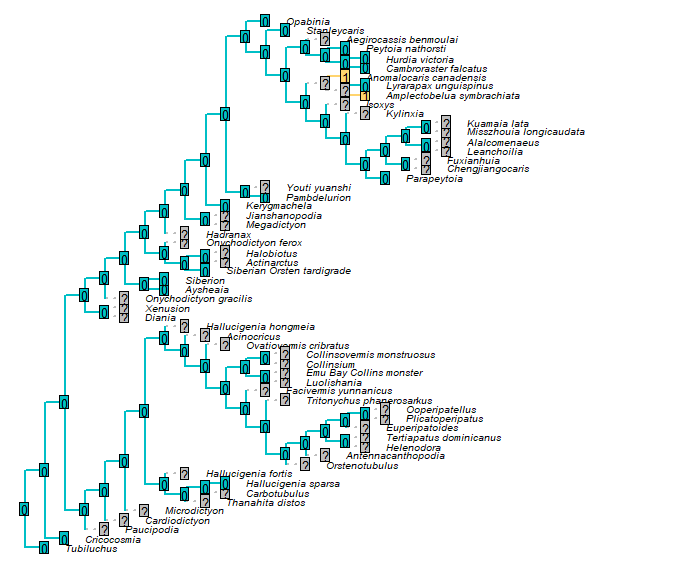


**Supplementary Figure 24: Oral structures: Furrowed folds on circumoral structures**

0 absent

1 present

### [25] Spinose projections from inner face of circumoral structures


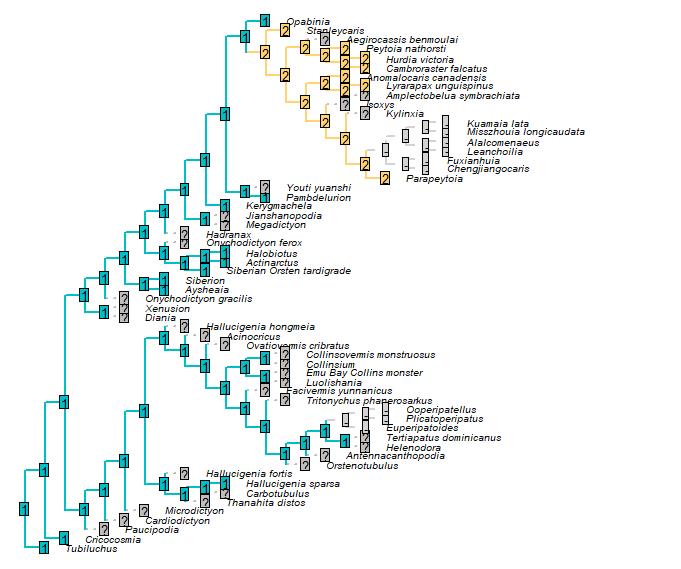


**Supplementary Figure 25: Oral structures: Spinose projections from inner face of circumoral structures**

- Inapplicable

1 proximal surface with single projection

2 proximal surface with multiple spines

The inner surface of the circumoral plates of *Anomalocaris*, *Peytoia* and *Hurdia* bear multiple spinose projections ([Daley & Bergström 2012](#ref-Daley2012); [Daley *et al.* 2013*a*](#ref-Daley2013jsp)), but the equivalent face of plates in priapulans and *Hallucigenia* *sparsa* lacks projections ([Smith & Caron 2015](#ref-Smith2015)).

Character 16 in Zhang *et al.* ([2016](#ref-Zhang2016)).

*Amplectobelua symbrachiata*: Cong *et al.* ([2017](#ref-Cong2017)) identify two spinose projections on one tuberculate plate (fig. 8e), but only one is unambiguously evident. We thus code this character as ambiguous.

*Cambroraster falcatus*: Multiple spines ([Moysiuk & Caron 2019](#ref-Moysiuk2019)).

*Lyrarapax unguispinus*: Multiple spines ([Liu *et al.* 2018](#ref-Liu2018nsr)).

*Opabinia*: Single projection ([Dhungana & Smith 2021](#ref-Dhungana2021)).

### [26] Expanded anterior gut


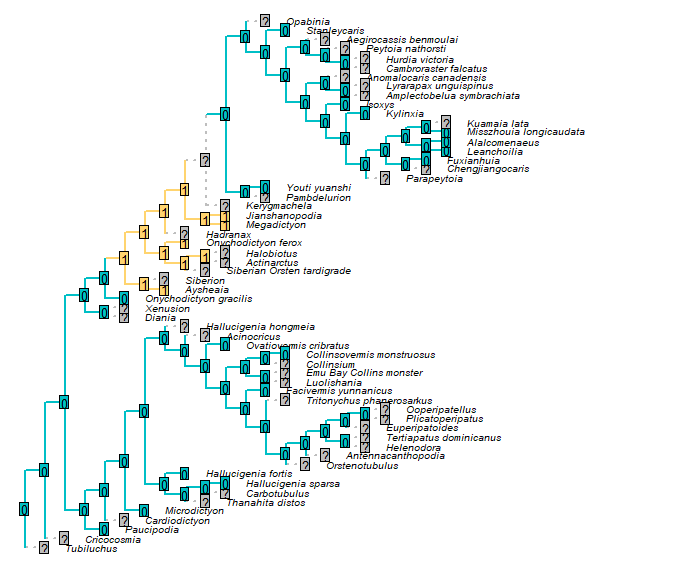


**Supplementary Figure 26: Oral structures: Expanded anterior gut**

0 anterior gut similar diameter to mid gut

1 expanded anterior gut

The gut of *Onychodictyon* *ferox* expands anteriad forming a cone shape (see [Vannier & Martin 2017](#ref-Vannier2017)), in contrast to many other lobopodian guts where the anterior region has a similar diameter to the mid-gut. Taxa with an eversible pharynx are coded as ambiguous.

This replaces the invariant character 17 from Zhang *et al.* ([2016](#ref-Zhang2016)), “Pharynx differentiated from midgut” [SC: 11].

*Aysheaia*: Anterior gut expanded ([Whittington 1978](#ref-Whittington1978), e.g. fig 43).

*Misszhouia longicaudata*: Chen *et al.* ([1997](#ref-Chen1997)).

*Paucipodia*: No indication of gut widening, whichever end is anterior Vannier & Martin ([2017](#ref-Vannier2017)).

### [27] Pharynx eversible


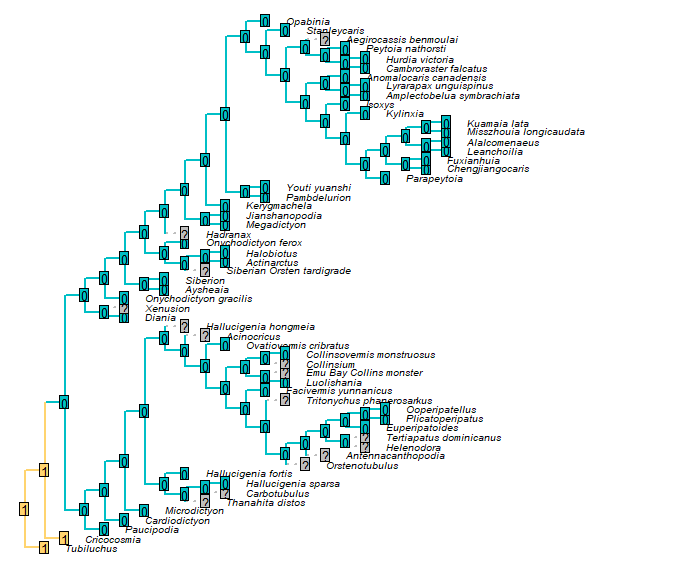


**Supplementary Figure 27: Oral structures: Pharynx eversible**

0 permanently inverted

1 completely eversible

The pharynx of priapulans and palaeoscolecids can be everted ([Conway Morris 1977](#ref-ConwayMorris1977)), whereas the panarthropod foregut is permanently inverted. No lobopodians exhibit complete eversion of the pharynx, even if some taxa display a certain degree of flexibility: as perhaps evident in the proboscis of *Onychodictyon* *ferox* ([Ou *et al.* 2012](#ref-Ou2012)) or the presumed suction-feeding mouthparts of anomalocaridids ([Daley & Bergström 2012](#ref-Daley2012)). Taxa whose mouth region is unknown or is known from a limited sample size are coded ambiguous to reflect the possibility that eversion was possible but not displayed in the available material.

Character 12 in Smith & Caron ([2015](#ref-Smith2015)).

*Kerygmachela*: Anterior position in certain specimens is attributed to post-mortem processes ([Park *et al.* 2018](#ref-Park2018)).

*Pambdelurion*: Oral cone eversible, but pharynx is not ([Vinther *et al.* 2016](#ref-Vinther2016)).

### [28] Sclerotized pharyngeal “teeth”


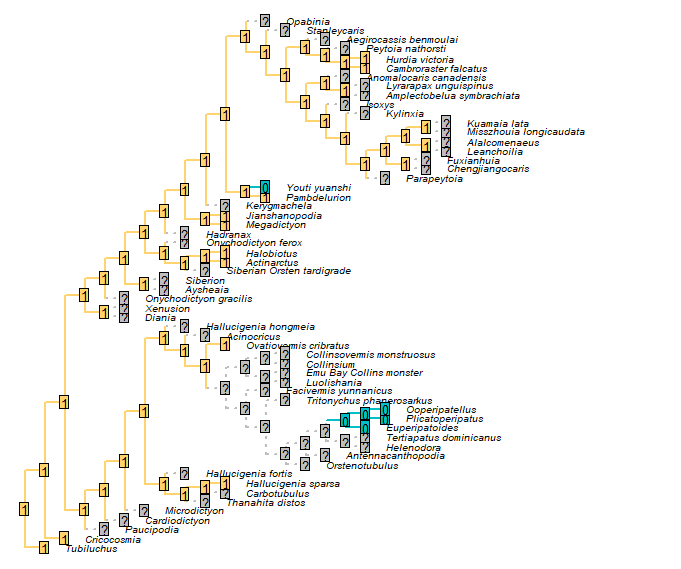


**Supplementary Figure 28: Oral structures: Sclerotized pharyngeal “teeth”**

0 absent

1 present

In many taxa the pharynx is lined with cuticular sclerites or “pharyngeal teeth”. Priapulans have an eversible pharynx lined with pharyngeal teeth ([van der Land 1970](#ref-vanderLand1970); [Conway Morris 1977](#ref-ConwayMorris1977)). *Hallucigenia* *sparsa* has a structurally differentiated (narrower) pharynx lined with acicular teeth ([Smith & Caron 2015](#ref-Smith2015)). *Jianshanopodia* bears a series of pharyngeal teeth with multiple cusps ([Liu *et al.* 2006](#ref-Liu2006); [Vannier *et al.* 2014](#ref-Vannier2014)). This fossil, perhaps alongside *Omnidens* ([Hou *et al.* 2006](#ref-Hou2006)), resembles a longitudinally extended *Hurdia* mouthpart; the inner rows of teeth in *Hurdia* are correspondingly interpreted as pharyngeal teeth ([Daley *et al.* 2009](#ref-Daley2009), [2013*a*](#ref-Daley2013jsp)). Sclerotized teeth have also been reported in the foregut of *Paucipodia* ([Hou *et al.* 2004](#ref-Hou2004)), although the nature and distribution of the teeth is not clear from the fossil material. Onychophorans bear a differentiated pharynx with an oesophageal constriction, but this is unornamented ([Elzinga 1998](#ref-Elzinga1998)). Most tardigrade taxa exhibit two to five rows of buccopharyngeal teeth (= mucrones) caudally to their circumoral lamellae ([Pilato 1972](#ref-Pilato1972); [Schuster *et al.* 1980](#ref-Schuster1980); [Hansen & Katholm 2002](#ref-Hansen2002); [Dastych *et al.* 2003](#ref-Dastych2003); [Guidetti *et al.* 2012](#ref-Guidetti2012)); a further row of sclerotized transverse ridges (= baffles) may also correspond to pharyngeal teeth. Coded as ambiguous in all other taxa due to inadequate preservation.

Character 13 in Smith & Caron ([2015](#ref-Smith2015)).

*Amplectobelua symbrachiata*: Preservation insufficient to evaluate ([Cong *et al.* 2017](#ref-Cong2017)).

*Cambroraster falcatus*: Present ([Moysiuk & Caron 2019](#ref-Moysiuk2019)).

*Ovatiovermis cribratus*: Present ([Caron & Aria 2017](#ref-Caron2017)).

*Pambdelurion*: Present ([Vinther *et al.* 2016](#ref-Vinther2016)).

### [29] Nature of pharyngeal teeth or aciculae


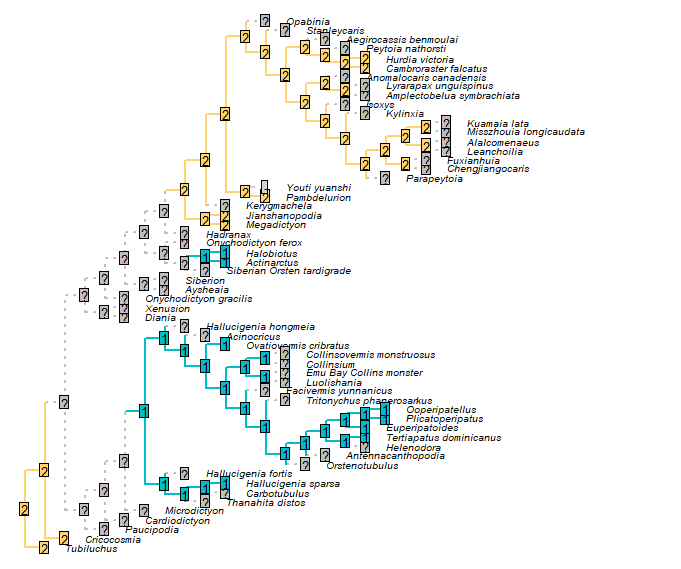


**Supplementary Figure 29: Oral structures: Nature of pharyngeal teeth or aciculae**

- Inapplicable

1 no elaboration of tooth point; spinose/acicular

2 each tooth has multiple cusps, perhaps expressed as denticles or serrations

Priapulans’ pharyngeal teeth exhibit a range of morphologies but always bear multiple cusps ([van der Land 1970](#ref-vanderLand1970); [Smith *et al.* 2015](#ref-Smith2015p)). *Hallucigenia* *sparsa* has acicular teeth that come to a single point ([Smith & Caron 2015](#ref-Smith2015)). The teeth of *Hurdia* and *Jianshanopodia* have multiple cusps ([Daley *et al.* 2009](#ref-Daley2009), [2013*a*](#ref-Daley2013jsp); [Vannier *et al.* 2014](#ref-Vannier2014)); tardigrade teeth do not ([Pilato 1972](#ref-Pilato1972); [Schuster *et al.* 1980](#ref-Schuster1980); [Hansen & Katholm 2002](#ref-Hansen2002); [Dastych *et al.* 2003](#ref-Dastych2003)).

Character 14 in Smith & Caron ([2015](#ref-Smith2015)).

*Cambroraster falcatus*: Multiple cusps ([Moysiuk & Caron 2019](#ref-Moysiuk2019)).

*Megadictyon*: From Vannier *et al.* ([2014](#ref-Vannier2014)) supplementary figure 6c, the pharyngeal teeth appear multicupsate, although only few are preserved well.

*Ovatiovermis cribratus*: Short spines ([Caron & Aria 2017](#ref-Caron2017)).

*Pambdelurion*: Multiple cusps inferred based on similarity to *Omnidens* ([Vinther *et al.* 2016](#ref-Vinther2016)).

### [30] Arrangement of pharyngeal teeth or aciculae


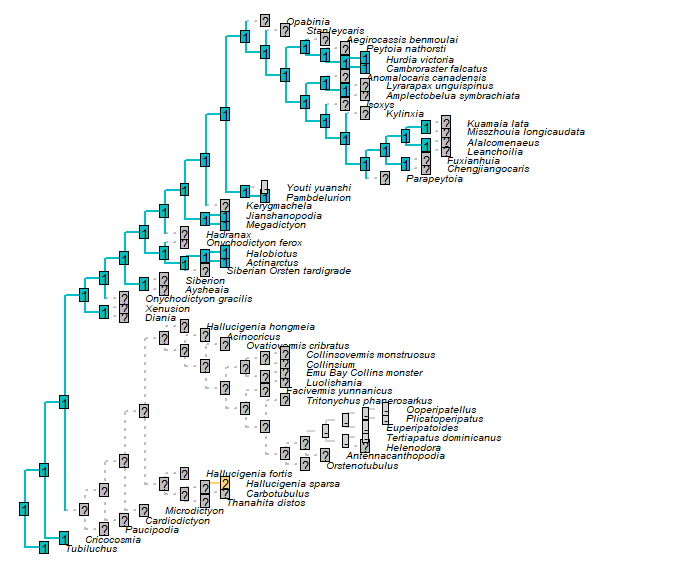


**Supplementary Figure 30: Oral structures: Arrangement of pharyngeal teeth or aciculae**

- Inapplicable

1 uniform distribution around pharynx

2 limited number of longitudinal rows or series

In contrast to the uniform distribution of sclerites in priapulans and total-group euarthropods, the pharyngeal teeth in *Hallucigenia* *sparsa* seem to occupy one or two longitudinal rows and do not cover the entire surface of the pharynx ([Smith & Caron 2015](#ref-Smith2015)).

Character 15 in Smith & Caron ([2015](#ref-Smith2015)).

*Cambroraster falcatus*: Four series ([Moysiuk & Caron 2019](#ref-Moysiuk2019)).

*Ovatiovermis cribratus*: Distribution unclear; possibly uniform around pharynx ([Caron & Aria 2017](#ref-Caron2017)), but coded as ambiguous.

*Pambdelurion*: Uniformly distributed ([Vinther *et al.* 2016](#ref-Vinther2016)).

### [31] Extent of pharyngeal teeth


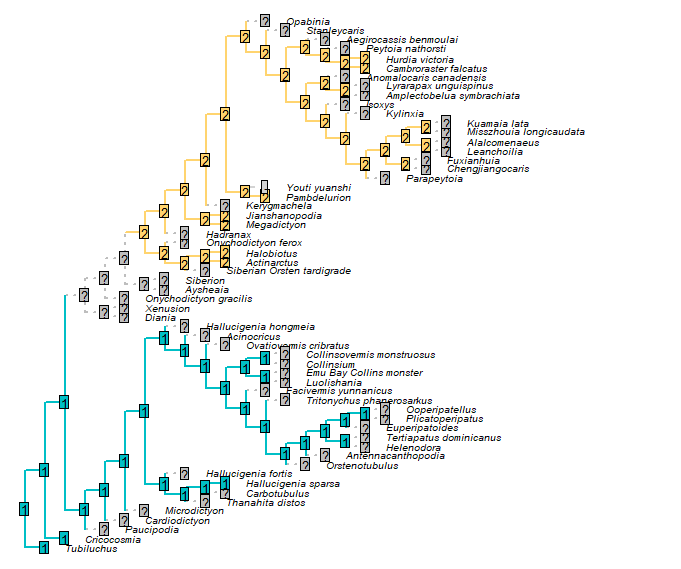


**Supplementary Figure 31: Oral structures: Extent of pharyngeal teeth**

- Inapplicable

1 teeth gap; pharyngeal teeth not directly adjacent

2 no teeth gap; pharyngeal teeth directly adjacent

Pharyngeal teeth in *Cricocosmia* do not extend to the circumoral spines of the introvert (e.g. [Cong *et al.* 2017](#ref-Cong2017)). Similarly in *Hallucigenia* there is an absence of pharyngeal teeth in proximity to the circumoral structures ([Smith & Caron 2015](#ref-Smith2015)). In tardigrades (e.g. [Hansen & Katholm 2002](#ref-Hansen2002)), *Hurdia* ([Daley *et al.* 2009](#ref-Daley2009)) and *Cambroraster* ([Moysiuk & Caron 2019](#ref-Moysiuk2019)), the pharyngeal teeth are directly adjacent to the radially arranged circumoral structures.

*Jianshanopodia*: No gap ([Liu *et al.* 2006](#ref-Liu2006)).

*Pambdelurion*: Directly adjacent ([Vinther *et al.* 2016](#ref-Vinther2016)).

*Tubiluchus*: Gap ([Schmidt-Rhaesa *et al.* 2017](#ref-Schmidt2017), fig 2A).

## Ocular structures [32]


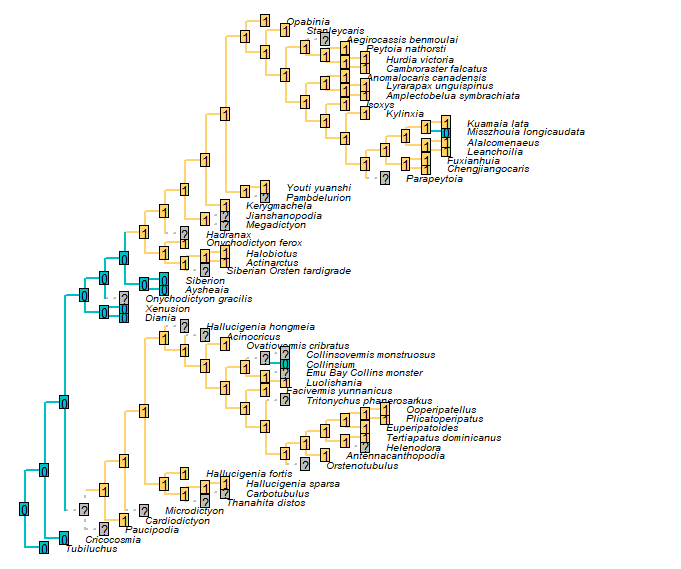


**Supplementary Figure 32: Ocular structures**

0 absent

1 present

Daley *et al.* ([2009](#ref-Daley2009)) (char. 10), Ma *et al.* ([2014*a*](#ref-Ma2014jsp)) (chars. 25, 27) and Lan *et al.* ([2021](#ref-Lan2021)) implicitly treat compound eyes and ocelli as homologous structures. We uphold the case for deep homology between these organs. Modified ocelli can resemble a single ommatidium of a compound eye ([Land & Nilsson 2012 pp. 125–126](#ref-Land2012)) and compound eyes can be de-differentiated into an ocelli during metamorphosis ([Bitsch & Bitsch 2005 sec. 3.1](#ref-Bitsch2005)). This implies a deep homology in fossils of ocelli and compound eyes despite notable differences in certain aspects, such as visual pigments, in ocelli and compound eyes in extant euarthropods ([Henze *et al.* 2012](#ref-Henze2012)). Paleontological support for this homology is reviewed by Schoenemann & Clarkson ([2023](#ref-Schoenemann2023)).

Adapted from characters: 16 and 18 in Smith & Caron ([2015](#ref-Smith2015)); 29 and 31 in Yang *et al.* ([2015](#ref-Yang2015)).

*Amplectobelua symbrachiata*: Stalked eyes present ([Cong *et al.* 2017](#ref-Cong2017)).

*Cambroraster falcatus*: Moysiuk & Caron ([2019](#ref-Moysiuk2019)).

*Cardiodictyon*: Bears a single pair of eyespots ([Liu & Dunlop 2014](#ref-Liu2014ppp)).

*Facivermis yunnanicus*: Pair of simple ocellus-like eyes ([Howard *et al.* 2020](#ref-Howard2020)).

*Hallucigenia fortis*: We follow Liu & Dunlop ([2014](#ref-Liu2014ppp)) in recognizing a single pair of eyespots. The various carbonaceous regions and pigmented patches ([Ma *et al.* 2012*b*](#ref-Ma2012asd)) likely represent a degraded but originally continuous carbon film.

*Hallucigenia sparsa*: Ocelli ([Smith & Caron 2015](#ref-Smith2015)).

*Kerygmachela*: Compound, following Park *et al.* ([2018](#ref-Park2018)).

*Luolishania*: Pit-type eyes (per [Smith & Caron 2015](#ref-Smith2015), char. 18).

*Microdictyon*: Coded ambiguous. A dark structure occurs in a location equivalent to the ocellus of *Hallucigenia* *sparsa* in ELRC 30060 ([Chen *et al.* 1995*a*](#ref-Chen1995bnmns), pl. 6 fig. 2); reexamination of fossil material is necessary before the absence of ocelli can be categorically confirmed.

*Onychodictyon ferox*: Present (cf. [Ou *et al.* 2012](#ref-Ou2012)).

*Ovatiovermis cribratus*: Sessile ocellus-type eyes ([Caron & Aria 2017](#ref-Caron2017)).

*Pambdelurion*: Coded ambiguous: the dorsal surface of *Pambdelurion* is poorly known ([Budd 1998*a*](#ref-Budd1998ar)).

### [33] Number


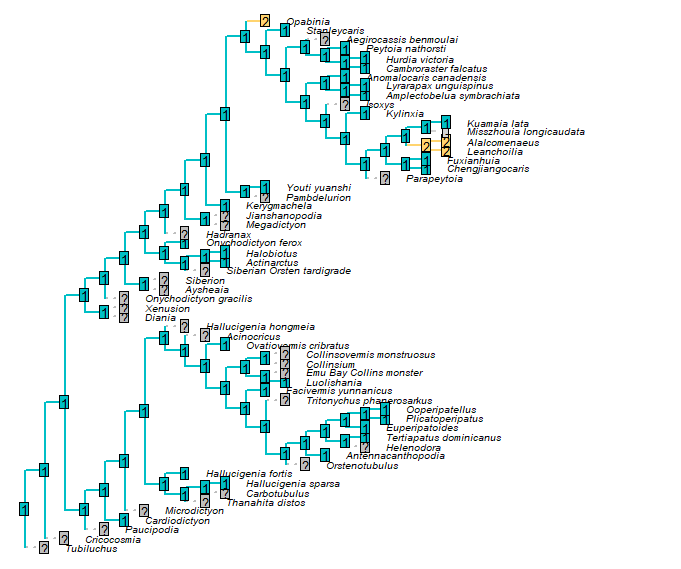


**Supplementary Figure 33: Ocular structures: Number**

- Inapplicable

1 Two

2 Four

Number of discrete visual units, whether compound eyes or ocelli. Despite differences in visual pigmentation and innervation, we hypothesize that all visual units – whether compound or singular – share a deep homology.

*Leanchoilia*, *Alalcomenaeus*: Sideward pair and forward pair ([Lan *et al.* 2021](#ref-Lan2021)).

*Amplectobelua symbrachiata*: Structures interpreted as eyes are not, so this remains ambiguous ([Cong *et al.* 2017](#ref-Cong2017)).

*Chengjiangocaris*: Two. Only two eyes have been described ([Yang *et al.* 2013](#ref-Yang2013)); we have been unable to substantiate the view of Lan *et al.* ([2021](#ref-Lan2021)) that fuxianhuiids exhibit medial ocelli in addition to their lateral compound eyes.

*Facivermis yunnanicus*: Pair of simple ocellus-like eyes ([Howard *et al.* 2020](#ref-Howard2020)).

*Fuxianhuia*: Though Lan *et al.* ([2021](#ref-Lan2021)) contend that fuxianhuiids exhibit medial ocelli in addition to their lateral compound eyes, Ma *et al.* ([2012*a*](#ref-Ma2012n)) interpret putative medial eyes as lateral extensions of the rostrum.

*Hallucigenia fortis*: We follow Liu & Dunlop ([2014](#ref-Liu2014ppp)) in recognizing a single pair of eyespots. In our view the various carbonaceous regions and pigmented patches ([Ma *et al.* 2012*b*](#ref-Ma2012asd)) likely represent a degraded but originally continuous carbon film.

*Hallucigenia sparsa*: Two ([Smith & Caron 2015](#ref-Smith2015)).

*Isoxys*: A large pair of compound eyes is present ([Fu *et al.* 2011](#ref-Fu2011); [Schoenemann & Clarkson 2011](#ref-Schoenemann2011)), but we consider it possible that a small pair of medial ocelli, if present, would be impossible to recognize in the preserved fossil material, so we conservatively code this taxon as ambiguous.

*Kylinxia*: One pair of lateral eyes ([O’Flynn *et al.* 2023](#ref-OFlynn2023cb)).

*Misszhouia longicaudata*: Eyes are secondarily lost in *Misszhouia* and other naraoiids ([Mayers *et al.* 2019](#ref-Mayers2019)).

*Opabinia*: Four ([Dhungana & Smith 2021](#ref-Dhungana2021)).

*Stanleycaris*: Two. We consider the “third eye” of Moysiuk & Caron ([2022](#ref-Moysiuk2022)) to correspond to nervous tissue in an anterior lobe.

### [34] Compound eyes


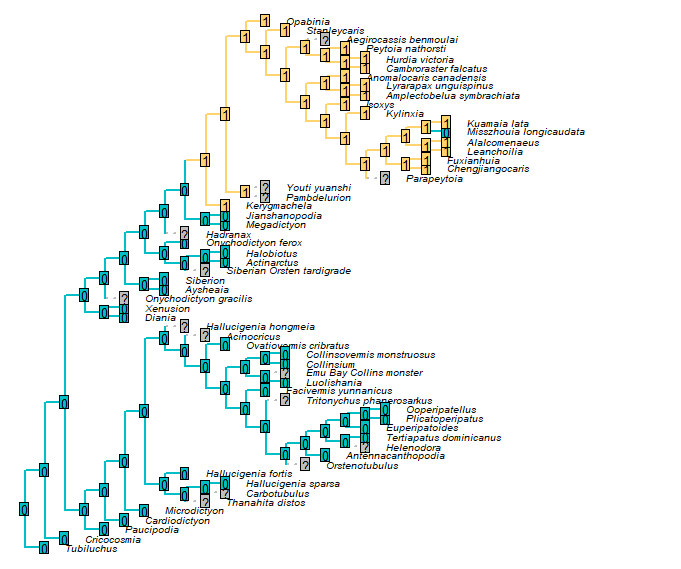


**Supplementary Figure 34: Ocular structures: Compound eyes**

0 absent

1 present

Treated as a separate organ from ocelli; see parent character for discussion.

Adapted from characters: 16 and 18 in Smith & Caron ([2015](#ref-Smith2015)); 29 and 31 in Yang *et al.* ([2015](#ref-Yang2015)).

*Amplectobelua symbrachiata*: Stalked eyes presumed compound ([Cong *et al.* 2017](#ref-Cong2017)).

*Cambroraster falcatus*: Moysiuk & Caron ([2019](#ref-Moysiuk2019)).

*Kerygmachela*: Present, following Park *et al.* ([2018](#ref-Park2018)).

*Misszhouia longicaudata*: Eyes are secondarily lost in *Misszhouia* and other naraoiids ([Mayers *et al.* 2019](#ref-Mayers2019)).

*Pambdelurion*: Coded ambiguous: the dorsal surface of *Pambdelurion* is poorly known ([Budd 1998*a*](#ref-Budd1998ar)).

### [35] Attachment


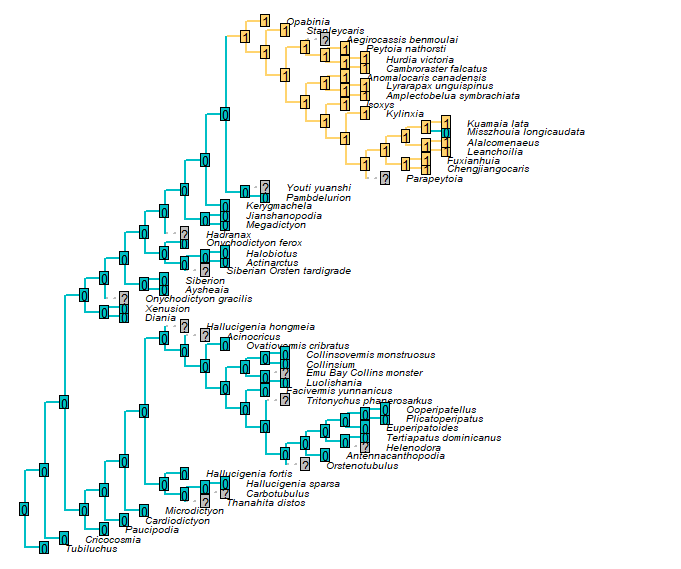


**Supplementary Figure 35: Ocular structures: Compound eyes: Attachment**

0 eye stalks absent

1 eye stalks present

Treated as neomorphic as a stalk represents an additional morphological structure.

Character 26 in Ma *et al.* ([2014*a*](#ref-Ma2014jsp)); character 17 in Smith & Caron ([2015](#ref-Smith2015)); and character 30 in Yang *et al.* ([2015](#ref-Yang2015)). Character 4 in Smith & Caron ([2015](#ref-Smith2015)) and Yang *et al.* ([2016](#ref-Yang2016)) is redundant to this character, so is not included in the present matrix.

*Hallucigenia sparsa*: Sessile ([Smith & Caron 2015](#ref-Smith2015)).

*Kerygmachela*: Sessile, following Park *et al.* ([2018](#ref-Park2018)).

*Misszhouia longicaudata*: Eyes are secondarily lost in *Misszhouia* and other naraoiids ([Mayers *et al.* 2019](#ref-Mayers2019)).

*Pambdelurion*: Whether or not eyes are present, available specimens clearly demonstrate the absence of an eye stalk ([Budd 1998*a*](#ref-Budd1998ar); [Young & Vinther 2017](#ref-Young2017)).

### [36] Posterior displacement


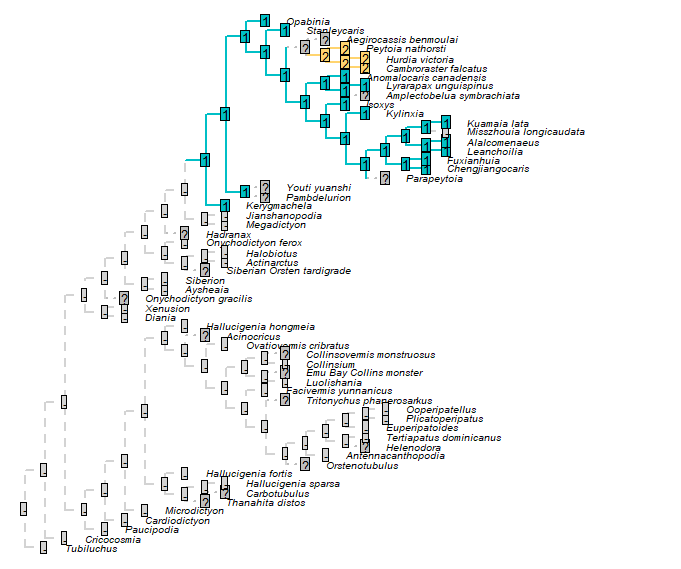


**Supplementary Figure 36: Ocular structures: Compound eyes: Posterior displacement**

- Inapplicable

1 approximately dorsal to mouth

2 significantly posterior of mouth

After character 15 in Moysiuk & Caron ([2019](#ref-Moysiuk2019)).
The eyes of certain hurdiid radiodonts are dislocated to an extremely posterior location.

*Kerygmachela*, *Opabinia*, *Anomalocaris canadensis*, *Cambroraster falcatus*, *Hurdia victoria*, *Peytoia nathorsti*, *Lyrarapax unguispinus*, *Chengjiangocaris*, *Fuxianhuia*, *Leanchoilia*, *Alalcomenaeus*, *Kuamaia lata*: Following Moysiuk & Caron ([2019](#ref-Moysiuk2019)).

*Misszhouia longicaudata*: Eyes are secondarily lost in *Misszhouia* and other naraoiids ([Mayers *et al.* 2019](#ref-Mayers2019)).

## Arthrodial membranes [37]


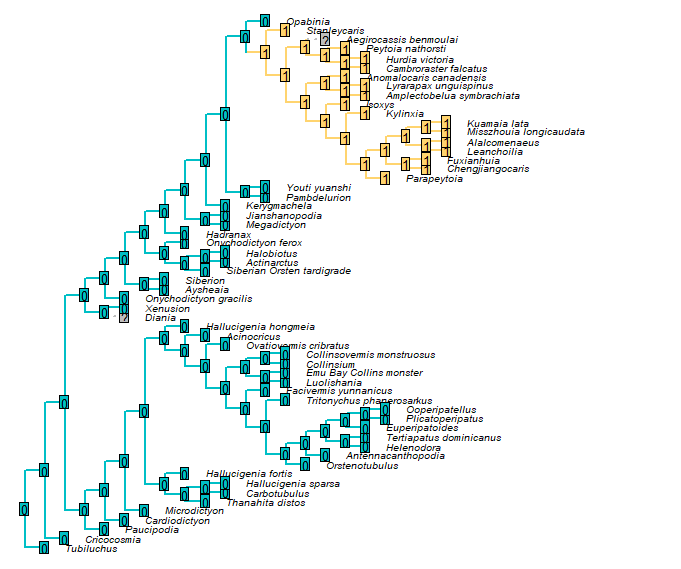


**Supplementary Figure 37: Arthrodial membranes**

0 membrane absent

1 membrane present

This character generalizes the innovation of arthrodial membranes as a part of the arthrodization process. Treated as neomorphic.

*Cambroraster falcatus*, *Hurdia victoria*, *Peytoia nathorsti*: Following De Vivo *et al.* ([2021](#ref-DeVivo2021)).

*Youti yuanshi*: Segmentation defined by arthrodial membranes is evident at all stages of the life cycle, so we discount the possibility that the absence here reflects ontogenetic stage.

## Cephalic/anterior appendages

### [38] Arthrodial membranes


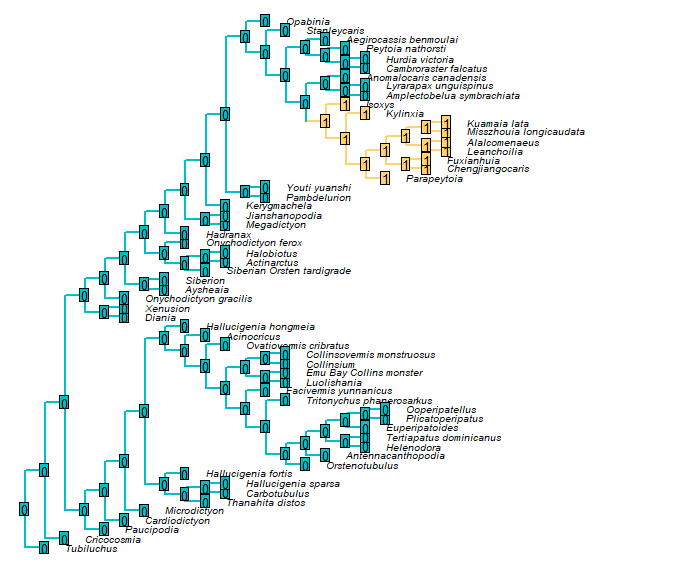


**Supplementary Figure 38: Cephalic/anterior appendages: Post-ocular (post-protocerebral) appendages: Arthrodial membranes**

0 arthrodial membranes absent

1 arthrodial membranes present

Character atomized from previous formulation to reflect complexity in “arthropodization” of the post-ocular appendages.

Adapted from character 19 in Smith & Caron ([2015](#ref-Smith2015)) and 8 in Yang *et al.* ([2015](#ref-Yang2015)).

*Amplectobelua symbrachiata*: Flaps are not sclerotized ([Chen *et al.* 1994](#ref-Chen1994)).

*Diania*: Although Liu *et al.* ([2011](#ref-Liu2011)) described the appendages of the lobopodian *Diania* as having an arthropodized organization, a recent revision of this taxon ([Ma *et al.* 2014*a*](#ref-Ma2014jsp)) concluded that the podomere-like structures on the legs represent taphonomic features on lobopodous appendages.

### [39] Structurally differentiated from trunk appendages


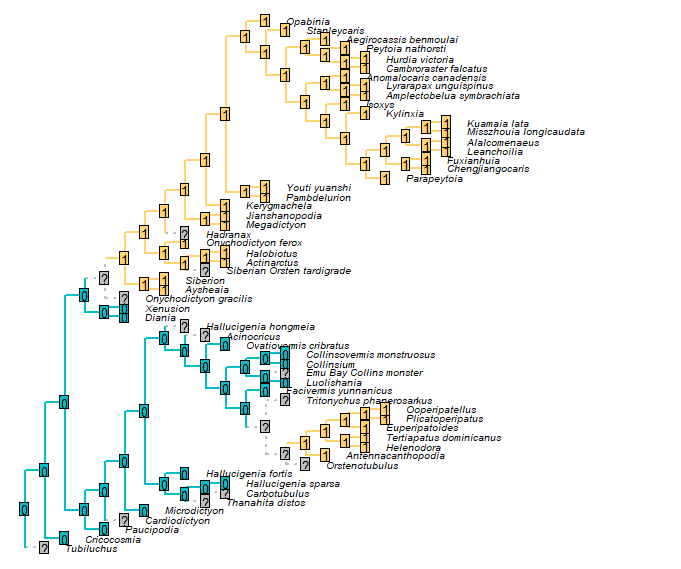


**Supplementary Figure 39: Cephalic/anterior appendages: Pre-ocular (protocerebral) limb pair: Structurally differentiated from trunk appendages**

0 pre-ocular limb pair absent or not differentiated from other limbs

1 distinct pre-ocular limb pair

In most panarthropods, the first pair of limbs is pre-ocular (at least developmentally), is associated with the protocerebral segment, and is structurally differentiated from other limb pairs. In hallucigeniids, however, the first limb pair is not structurally differentiated from its neighbour; moreover, the great distance between the head and the first limb pair in *Hallucigenia* *sparsa* ([Smith & Caron 2015](#ref-Smith2015)) argues against a pre-ocular or indeed cerebral identity of these appendages. Whether or not the first appendage pair truly corresponds to the pre-ocular appendage of other groups, the absence of a differentiated pre-ocular appendage characterizes a number of armoured lobopodians: *Xenusion* ([Dzik & Krumbiegel 1989](#ref-Dzik1989)), *Diania* ([Ma *et al.* 2014*a*](#ref-Ma2014jsp)), *Microdictyon* ([Chen *et al.* 1995*a*](#ref-Chen1995bnmns)), *Paucipodia* ([Chen *et al.* 1995*b*](#ref-Chen1995trse); [Hou *et al.* 2004](#ref-Hou2004)), *H. fortis* ([Ramsköld & Chen 1998](#ref-Ramskold1998)), and *H. sparsa* ([Smith & Caron 2015](#ref-Smith2015)). A distinct structure is evident in onychophorans, *Antennacanthopodia* and *Ilyodes* (antennae); tardigrades (the stylet apparatus); anomalocaridids (great appendages) ([Cong *et al.* 2014](#ref-Cong2014)); *Opabinia* (proboscis) ([Dhungana & Smith 2021](#ref-Dhungana2021)); and euarthropods and basal panarthropods (homologues of the labrum) ([Budd 2021](#ref-Budd2021)). We differ from previous studies in homologizing the antenniform appendages of luolishaniids with frontal filaments, rather than appendage, reflecting their dorsal position and lack of obvious parallels with the differentiated trunk appendages. Coded as ambiguous in taxa where the head is not preserved (including *Carbotubulus*).

Character 20 in Smith & Caron ([2015](#ref-Smith2015)).

*Actinarctus*: In tardigrades, the presence of stylet glands, responsible for the moulting and production of stylet and stylet supports are likely transformed claw glands ([Møbjerg *et al.* 2018](#ref-Mobjerg2018)). As such stylets and stylet supports are interpreted as modified claws ([Nielsen 2001](#ref-Nielsen2001); [Halberg *et al.* 2009](#ref-Halberg2009)). This homology is supported by the presence of microtubules in the epidermal cell attachments of exclusively the retractor muscles of claws and stylets in tardigrades ([Halberg *et al.* 2009](#ref-Halberg2009)). We therefore interpret the frontal appendages as structurally differentiated from trunk appendages.

Kihm *et al.* ([2023](#ref-Kihm2023)) argue that the tardigrade trunk exhibits differentiated anterior and posterior sections in a manner reminiscent of luolishaniids; on this view, the posteriormost appendage pair belong to a separate region of the trunk and are differently patterned. In our view, this overemphasizes the morphological distinction between the anterior and posterior tardigrade appendages. We interpret the four tardigrade appendage pairs as trunk appendages, noting that the reversal/modification of the single posteriormost pair is akin to the modification of the posterior appendage pair (only) in other euarthropods and onychophorans. We do not see an obvious basis for equating the lobopodiform appendages of the anterior tardigrade trunk with the highly modified anterior trunk appendages of hallucishaniids, which have been repurposed for sensing / feeding.

*Cardiodictyon*: Coded as ambiguous, as the detailed morphology of the head is unclear.

*Collinsium*: We code the anterior antennae-like structures ([Yang *et al.* 2015](#ref-Yang2015)) as possible homologous of the frontal filaments. Hence the first pair of limbs are coded as undifferentiated.

*Collinsovermis monstruosus*: The first pair of appendages are not differentiated ([Caron & Aria 2020](#ref-Caron2020)). We code the anterior antennae-like structures as possible homologous of the frontal filaments.

*Cricocosmia*: Following Dhungana ([2024](#ref-Dhungana2024)).

*Facivermis yunnanicus*: Not differentiated ([Howard *et al.* 2020](#ref-Howard2020)).

*Luolishania*: We interpret the antenniform structures ([Ma *et al.* 2009](#ref-Ma2009)) as possible homologues to the frontal filaments rather than appendages.

*Ovatiovermis cribratus*: Not evident ([Caron & Aria 2017](#ref-Caron2017)).

### [40] Sclerotization


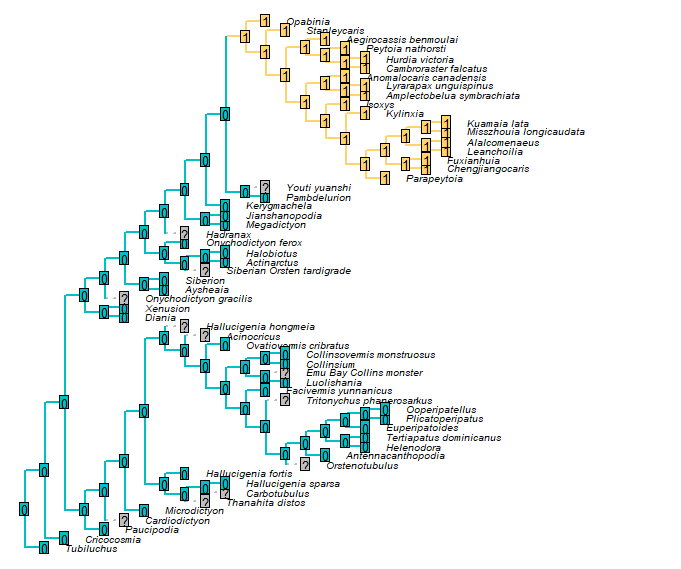


**Supplementary Figure 40: Cephalic/anterior appendages: Protocerebral appendage pair: Sclerotization**

0 not sclerotized

1 sclerotized

This new character reflects the hypothesis that sclerotization originated in the protocerebral (preocular) appendages, before being co-opted in trunk appendages. Modified from Yang *et al.* ([2015](#ref-Yang2015)) character “Cephalic/anterior appendages: Protocerebral limb pair sclerotized”(character 9; also character 21 in Smith & Caron ([2015](#ref-Smith2015))).

We code this character as present in any taxon with sclerotized pre-ocular (protocerebral) limbs, including the podomeres in anomalocaridid “great appendages” ([Daley & Edgecombe 2014](#ref-Daley2014)) and the hypostome that covers the euarthropod labrum (e.g. [Edgecombe & Ramsköld 1999](#ref-Edgecombe1999); [Yang *et al.* 2013](#ref-Yang2013)). We score this character as uncertain in taxa where the presence of a hypostome is suggested, but not verified (e.g. *Alalcomenaeus*), and in the Siberian “Orsten” tardigrade ([Maas & Waloszek 2001](#ref-Maas2001)), where (assuming its modification to a stylet, as in modern tardigrades) it cannot be directly observed.
The character is treated as neomorphic, as sclerotization represents a novel increase in the complexity of the appendage.

*Actinarctus*, *Halobiotus*: The sclerotized stylets and stylet supports of tardigrades are likely modified claws (see [Møbjerg *et al.* 2018](#ref-Mobjerg2018)) hence no appendage sclerotization (or arthrodial membranes) are present.

*Opabinia*: We interpret the claws of *Opabinia*’s protocerebral appendage as podomerous (see [Whittington 1975 figs 75, 79](#ref-Whittington1975)). Therefore the protocerebral appendage is sclerotized.

*Youti yuanshi*: Probably not sclerotized (this study) – but coded conservatively.

### [41] Podomeres


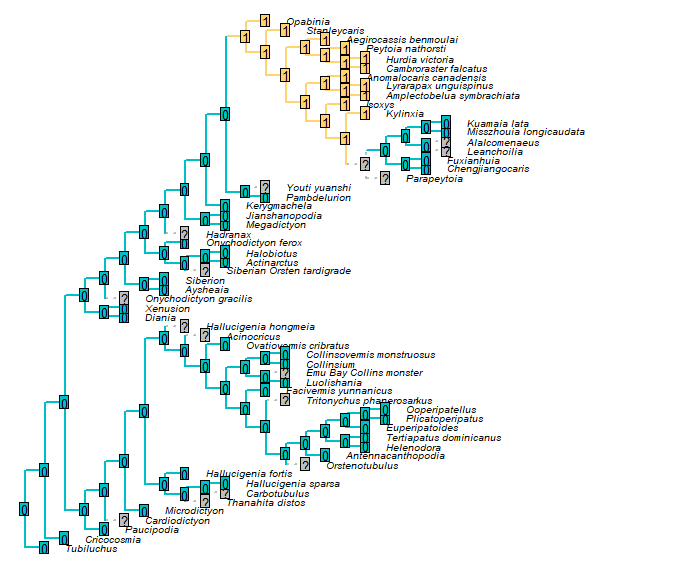


**Supplementary Figure 41: Cephalic/anterior appendages: Protocerebral appendage pair: Podomeres**

0 absent

1 present

This neomorphic character distinguishes the arthropodized “great appendages” of radiodontans ([Daley & Edgecombe 2014](#ref-Daley2014)) from the hypostome of Euarthropoda (e.g. [Edgecombe & Ramsköld 1999](#ref-Edgecombe1999))

Adapted from character 22 in Smith & Caron ([2015](#ref-Smith2015)) and character 10 in Yang *et al.* ([2015](#ref-Yang2015)).

*Actinarctus*, *Halobiotus*: The sclerotized stylets and stylet supports of tardigrades are likely modified claws (see [Møbjerg *et al.* 2018](#ref-Mobjerg2018)) hence no appendage sclerotization (or arthrodial membranes) are present.

*Opabinia*: We interpret the claws of *Opabinia*’s protocerebral appendage as podomerous (see [Whittington 1975 figs 75, 79](#ref-Whittington1975)). Therefore the protocerebral appendage is sclerotized.

*Youti yuanshi*: Conservatively coded as ambiguous to reflect possibility of later development of podomeres.

### [42] Position


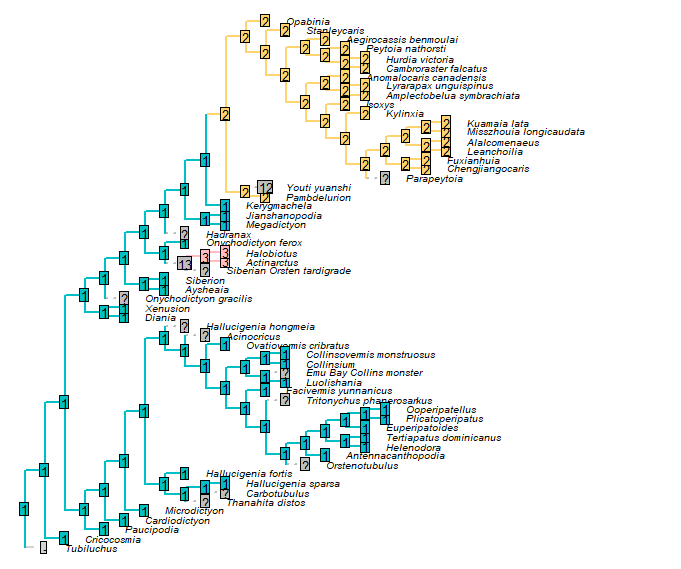


**Supplementary Figure 42: Cephalic/anterior appendages: Protocerebral appendage pair: Position**

- Inapplicable

1 lateral

2 ventral

3 within mouth cavity

We score this character as ventral in Euarthropoda given that the reduced protocerebral appendage pair, transformed into the labrum, occupies a ventral position in association with the mouth (e.g. [Scholtz & Edgecombe 2006](#ref-Scholtz2006)). As the forward-facing stylet apparatus of tardigrades is internalized into the mouth cone ([Halberg *et al.* 2009](#ref-Halberg2009)), the position of the stylets are not independent of the mouth position, therefore we code this as an alternative character state.

Character 26 in Smith & Caron ([2015](#ref-Smith2015)) and character 16 in Yang *et al.* ([2015](#ref-Yang2015)).

*Cricocosmia*: Following Dhungana ([2024](#ref-Dhungana2024)).

*Helenodora*: The first pair of appendages in *Ilyodes* are lateral ([Thompson & Jones 1980](#ref-Thompson1980); [Haug *et al.* 2012*b*](#ref-Haug2012cb)).

*Pambdelurion*: Ventral ([Budd 1998*a*](#ref-Budd1998ar)).

*Youti yuanshi*: Ventrolateral – adult position uncertain.

### [43] Posterior shift


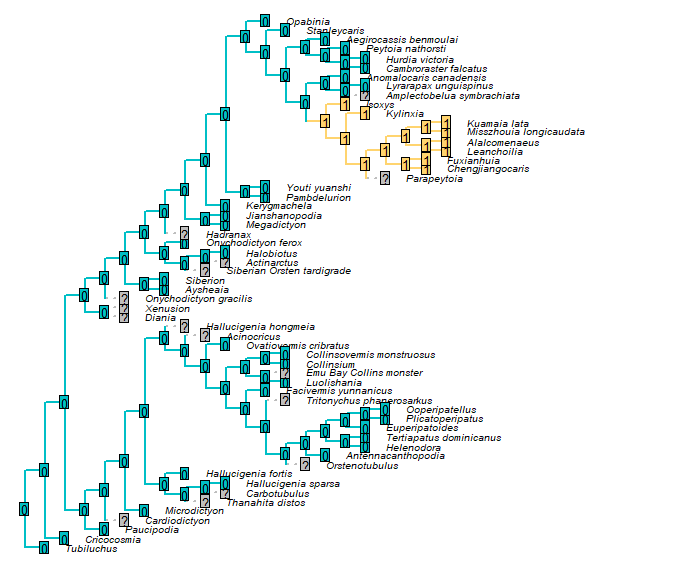


**Supplementary Figure 43: Cephalic/anterior appendages: Protocerebral appendage pair: Posterior shift**

0 frontal appendages not shifted posteriorly

1 frontal appendages shifted posteriorly

This character reflects the migration of the frontal appendages from an ancestrally anterior position, as in lobopodians (e.g. *Kerygmachela*, *Jianshanopodia*, *Pambdelurion*, *Siberion*), to a more posterior (e.g. Megacheirans and Leanchoiliids) and ultimately ventral position, as in the euarthropod labrum ([Budd 2021](#ref-Budd2021)).

As the direction of evolution is well attested by developmental data, we treat this character as neomorphic.

*Halobiotus*: As the mouth is terminal, and the appendages have been assumed to be incorporated into the mouth, we code that the frontal appendages have not shifted posteriorly.

### [44] Directly adjacent to one another


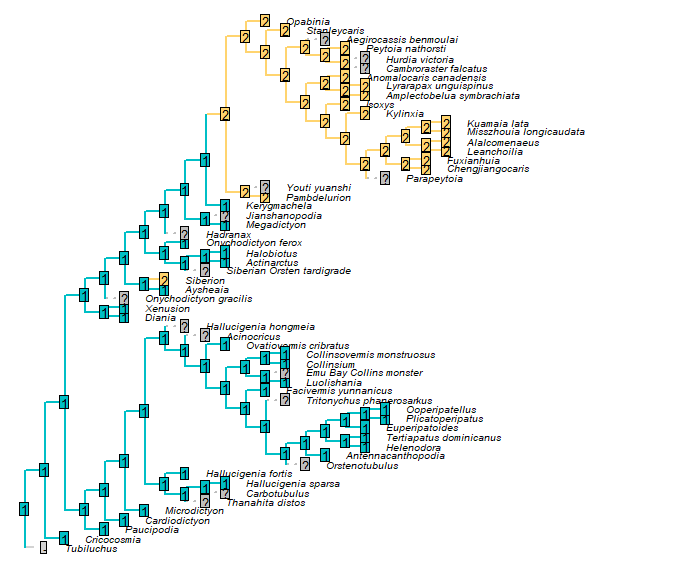


**Supplementary Figure 44: Cephalic/anterior appendages: Protocerebral appendages: Directly adjacent to one another**

- Inapplicable

1 pre-ocular appendages not directly adjacent

2 pre-ocular appendages adjacent to one another, with or without physical fusion

Modified from character 16 in Ma *et al.* ([2014*a*](#ref-Ma2014jsp)) to reflect the posited homology between the anterior appendages of lobopodians and the euarthropod labrum (cf. [Eriksson & Budd 2000](#ref-Eriksson2000); [Budd 2002](#ref-Budd2002)): specifically, the euarthropod labrum is coded as a fused pair of appendages ([Scholtz & Edgecombe 2006](#ref-Scholtz2006); [Liu *et al.* 2009](#ref-Liu2009), [2010](#ref-Liu2010); [Posnien *et al.* 2009](#ref-Posnien2009)). The stylet apparatus of Tardigrada is not coded as fused, as each stylet within the buccal tube remains independent despite significant modification ([Dewel & Eibye-Jacobsen 2006](#ref-Dewel2006); [Halberg *et al.* 2009](#ref-Halberg2009); [Guidetti *et al.* 2012](#ref-Guidetti2012)).

Character 27 in Smith & Caron ([2015](#ref-Smith2015)) and 17 in Yang *et al.* ([2015](#ref-Yang2015)).

*Hurdia victoria*, *Aegirocassis benmoulai*: Coded as ambiguous, as the well-developed dorsal cephalic plate in *Hurdia* and *Aegirocassis* obscures the base of the appendages ([Daley *et al.* 2009](#ref-Daley2009); [Van Roy *et al.* 2015](#ref-VanRoy2015)).

*Amplectobelua symbrachiata*: Adjacent in better-articulated material, and thus presumably in life ([Cong *et al.* 2017](#ref-Cong2017)).

*Cricocosmia*: Following Dhungana ([2024](#ref-Dhungana2024)).

*Helenodora*: The first pair of appendages in *Ilyodes* are not directly adjacent ([Thompson & Jones 1980](#ref-Thompson1980); [Haug *et al.* 2012*b*](#ref-Haug2012cb)).

*Jianshanopodia*: *Jianshanopodia* is coded uncertain due to unclear preservation ([Liu *et al.* 2006](#ref-Liu2006), [2007](#ref-Liu2007az)).

*Kerygmachela*: Not directly adjacent, but separated by anterior lobe ([Park *et al.* 2018](#ref-Park2018)).

*Megadictyon*: The first pair of appendages in *Megadictyon* are not directly adjacent ([Liu *et al.* 2007](#ref-Liu2007az)).

*Youti yuanshi*: Coded as uncertain as protocerebral appendages are known to migrate during development ([Budd 2021](#ref-Budd2021)).

### [45] Basal adjacency


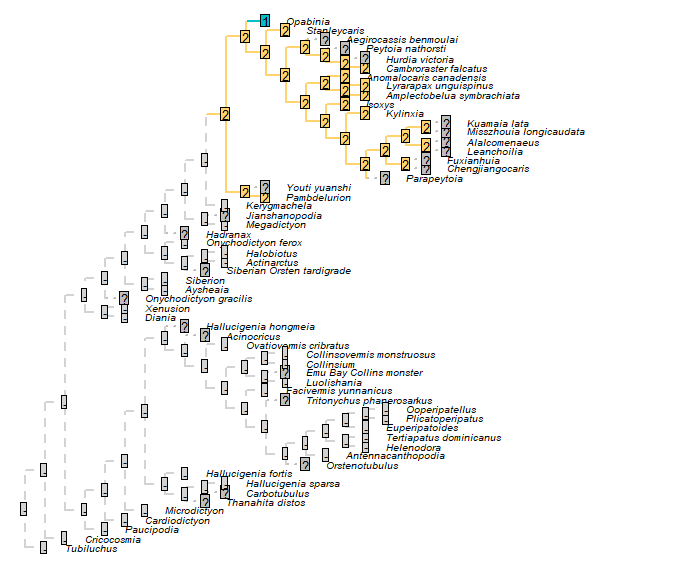


**Supplementary Figure 45: Cephalic/anterior appendages: Protocerebral appendages: Basal adjacency**

- Inapplicable

1 basally adjacent

2 bases separated by physical gap

In *Opabinia*, *Caryosyntrips* and cf. *Peytoia* ([Moysiuk & Caron 2021](#ref-Moysiuk2021)), the protocerebral appendages are adjacent to the other, without a gap; in radiodonts such as *Anomalocaris* *canadensis*, the protocerebral appendages are separated by a gap (e.g. [Daley & Edgecombe 2014 fig. 1](#ref-Daley2014)). The situation is unclear in many hurdiids due to limited preservation of appendage bases. The adjacency of bases is a prerequisite for the physical mechanical fusion of the protocerebral appendages.

*Cambroraster falcatus*: Figure 2J in Moysiuk & Caron ([2019](#ref-Moysiuk2019)) shows a prominent gap between appendages.

*Youti yuanshi*: Coded as uncertain as protocerebral appendages are known to migrate during development ([Budd 2021](#ref-Budd2021)).

### [46] Mechanical fusion


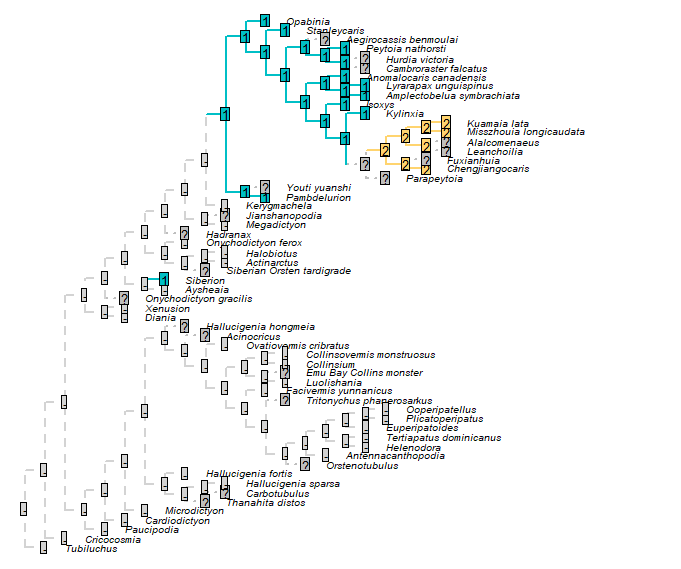


**Supplementary Figure 46: Cephalic/anterior appendages: Protocerebral appendages: Mechanical fusion**

- Inapplicable

1 pre-ocular appendages adjacent but not mechanically fused

2 pre-ocular appendages are mechanically fused to form a single element

In *Kerygmachela*, *Pambdelurion* and *Siberion*, the appendages have migrated into an adjacent position but are not mechanically connected ([Budd 1993](#ref-Budd1993); [1998*a*](#ref-Budd1998ar), [*b*](#ref-Budd1998trse); [Dzik 2011](#ref-Dzik2011)); this also seems to be the case in radiodontans ([Daley *et al.* 2009](#ref-Daley2009); [Daley & Edgecombe 2014](#ref-Daley2014)). In euarthropods, the appendages exhibit a degree of fusion.

Character 28 in Smith & Caron ([2015](#ref-Smith2015)), cf. character 17 in Yang *et al.* ([2015](#ref-Yang2015)).

*Cricocosmia*: Following Dhungana ([2024](#ref-Dhungana2024)).

*Youti yuanshi*: Coded as uncertain as protocerebral appendages are known to migrate during development ([Budd 2021](#ref-Budd2021)).

### [47] Loss of claws


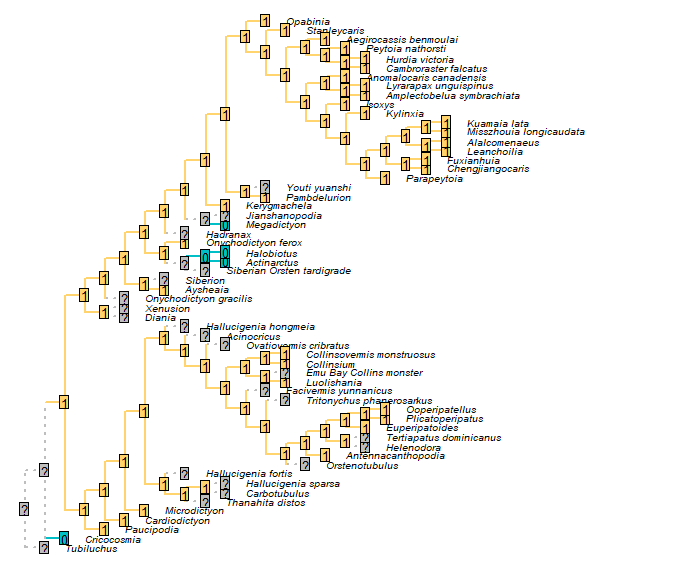


**Supplementary Figure 47: Cephalic/anterior appendages: Protocerebral appendage pair: Loss of claws**

0 no loss of claws on differentiated protocerebral appendage

1 differentiated protocerebral appendage claws lost

This neomorphic character represents the loss of claws on the (differentiated) protocerebral appendage as compared to the (undifferentiated) trunk appendages. By definition, taxa with undifferentiated protocerebral appendages have not undergone loss of claws on those appendages. Taxa without claws are coded as ambiguous as we cannot tell if a claw suppression mechanism acts silently in the protocerebral appendages; in other words, the gain or loss of claws on the trunk represents a separate neomorphic event and is thus independent of this character.

*Actinarctus*, *Halobiotus*: In tardigrades, the presence of stylet glands, responsible for the moulting and production of stylet and stylet supports are likely transformed claw glands ([Møbjerg *et al.* 2018](#ref-Mobjerg2018)). As such stylets and stylet supports are interpreted as modified claws ([Nielsen 2001](#ref-Nielsen2001); [Halberg *et al.* 2009](#ref-Halberg2009)).

*Aysheaia*: No claws on differentiated protocerebral appendages (see [Whittington 1978](#ref-Whittington1978)).

*Facivermis yunnanicus*: Claws absent in multiple anterior appendages ([Howard *et al.* 2020](#ref-Howard2020)), therefore coded as ambiguous (although posterior appendages are clawed).

*Hallucigenia sparsa*: Claws are absent in multiple anterior appendages, therefore coded as ambiguous (although posterior appendages are clawed).

*Jianshanopodia*: Not evident from incompletely preserved available material ([Liu *et al.* 2006](#ref-Liu2006); [Vannier *et al.* 2014](#ref-Vannier2014)).

*Kerygmachela*: Claws absent on protocerebral appendages ([Park *et al.* 2018](#ref-Park2018), supplementary figure 3).

*Megadictyon*: Liu *et al.* ([2007](#ref-Liu2007az)) suggest claws present on differentiated protocerebral appendages of *Megadictyon*; these are figured by Vannier *et al.* ([2014](#ref-Vannier2014)).

*Opabinia*: *Opabinia*’s protocerebral spines are not homologous to lobopodian-style claws.

*Pambdelurion*: Following Vinther *et al.* ([2016](#ref-Vinther2016)) we code terminal claws on protocerebral appendages to be absent. The terminal structures are not well differentiated from the pointed outgrowths along the inner edge of the appendages and no terminal claw can be readily distinguished (see Vinther *et al.* ([2016](#ref-Vinther2016)), fig. 1; contra Vannier *et al.* ([2014](#ref-Vannier2014))).

*Siberion*: Potential claws not preserved, therefore ambiguous ([Dzik 2011](#ref-Dzik2011)).

*Xenusion*: Although protocerebral appendage is undifferentiated, claws are not preserved ([Dzik & Krumbiegel 1989](#ref-Dzik1989)) and as such this cell is coded as ambiguous.

### [48] Spine series


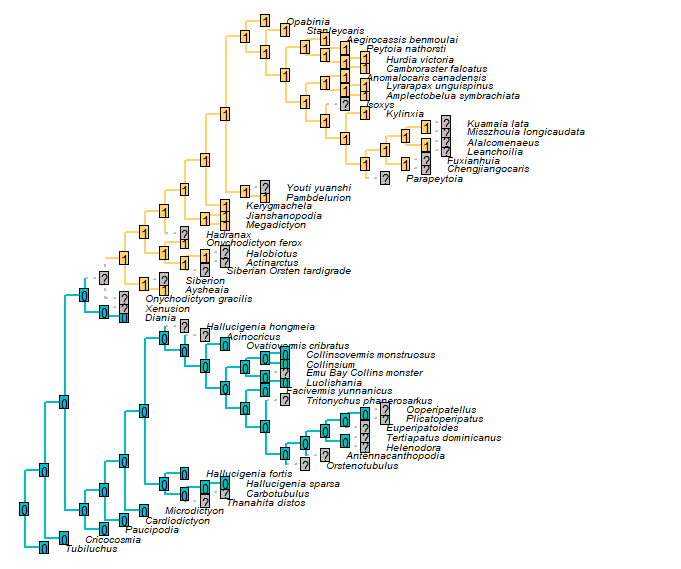


**Supplementary Figure 48: Cephalic/anterior appendages: Protocerebral appendage pair: Spine series**

0 absent

1 present

This neomorphic character refers to the spines/spinules present in the most anterior appendage pair of anomalocaridids ([Daley *et al.* 2009](#ref-Daley2009); [Daley & Edgecombe 2014](#ref-Daley2014)), gilled lobopodians (*Kerygmachela*, see [Budd 1993](#ref-Budd1993), [1998*b*](#ref-Budd1998trse); *Pambdelurion*, see [Budd 1998*a*](#ref-Budd1998ar); *Opabinia*, see [Budd 1996](#ref-Budd1996)) and certain lobopodians (e.g. *Aysheaia*, see [Whittington 1978](#ref-Whittington1978); *Jianshanopodia*, see [Liu *et al.* 2006](#ref-Liu2006); *Megadictyon*, see [Liu *et al.* 2007](#ref-Liu2007az); *Onychodictyon* *ferox*, see [Ou *et al.* 2012](#ref-Ou2012)).

Coded as inapplicable in tardigrades due to the extreme modification of the pre-ocular appendage into a stylet apparatus, which poses challenges to the identification of homologues of appendicular features.

We treat the presence of lateral and ventral spine series as different characters. Certain taxa (e.g. *Stanleycaris*, cf. *Peytoia*) have both series present, whereas other taxa (e.g., *Caryosyntrips*) have only lateral spine series (see [Moysiuk & Caron 2021](#ref-Moysiuk2021)). We extend this homology scheme to stem-euarthropods with lateral spine series such as *Kerygmachela*, *Pambdelurion* and *Opabinia* (following [Dhungana 2024](#ref-Dhungana2024)). Lateral spine series (referred to as “gnathal” spines in [Moysiuk & Caron 2021](#ref-Moysiuk2021)) in sclerotized appendages are often characterized by small asymmetric accessory spines that originate near the base of the main lateral spine ([Moysiuk & Caron 2021](#ref-Moysiuk2021)).

Ventral spine series (endites) characterize most radiodonts (with the notable exception of *Caryosyntrips*). These ventral spines have regularly spaced accessory spines along their length in Hurdiids. *Anomalocaris* and *Lyrarapax* symmetric accessory spines originating at the base of the main ventral spines. The similarity of *Kylinxia*’s “dorsal” spine series to *Anomalocaris* indicates possible homology ([Zeng *et al.* 2020](#ref-Zeng2020)), and rotation of the protocerebral appendages. See Guo *et al.* ([2019](#ref-Guo2019)) for an overview of radiodont appendage morphology.

Characters 42 of Zhang *et al.* ([2016](#ref-Zhang2016)) is redundant under this formulation, so has been removed from our matrix. Adapted from character 30 in Smith & Caron ([2015](#ref-Smith2015)) and 19 in Yang *et al.* ([2015](#ref-Yang2015)).

*Helenodora*: Coded as absent ([Haug *et al.* 2012*b*](#ref-Haug2012cb)).

*Onychodictyon ferox*: Spine series present on differentiated protocerebral appendage, therefore coded as present.

*Youti yuanshi*: Coded ambiguous as protocerebral appendages appear to be in an early developmental stage (this study); adult morphology is uncertain.

### [49] Spine/spinules: Number


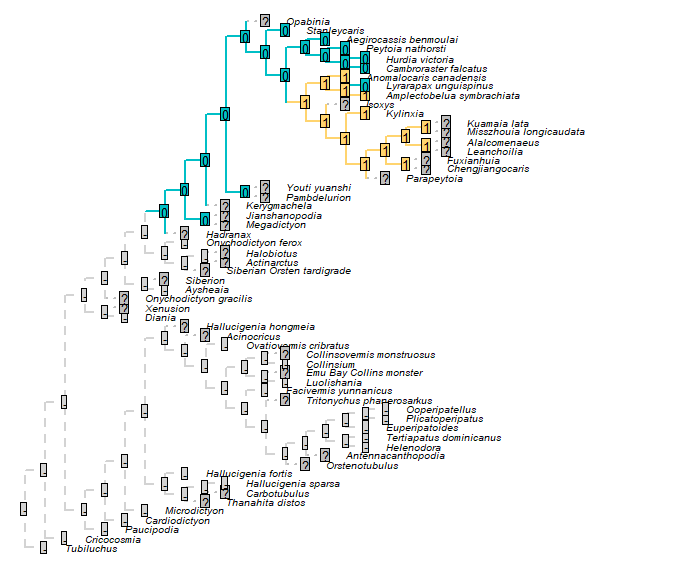


**Supplementary Figure 49: Cephalic/anterior appendages: Protocerebral appendages: Spine/spinules: Number**

0 one row

1 two rows

2 more than two rows

This character records the number of rows of ventral spines (endites). Amplectobeluidae and Anomalocarididae have two rows, Hurdiidae have one row ([Guo *et al.* 2019](#ref-Guo2019)).

Reformulated from char. 31 in Smith & Caron ([2015](#ref-Smith2015)) and char. 20 in Yang *et al.* ([2015](#ref-Yang2015)), which did not distinguish the lateral and ventral spine series (see discussion of character “Protocerebral appendage pair: Spine series”).

*Amplectobelua symbrachiata*: Paired ventral endites are present on podomeres 2–9 only ([Daley & Budd 2010](#ref-Daley2010)).

*Kylinxia*: Following Zeng *et al.* ([2020](#ref-Zeng2020)).

*Youti yuanshi*: Coded ambiguous as protocerebral appendages appear to be in an early developmental stage (this study); adult morphology is uncertain.

### [50] Spine/spinules: Height


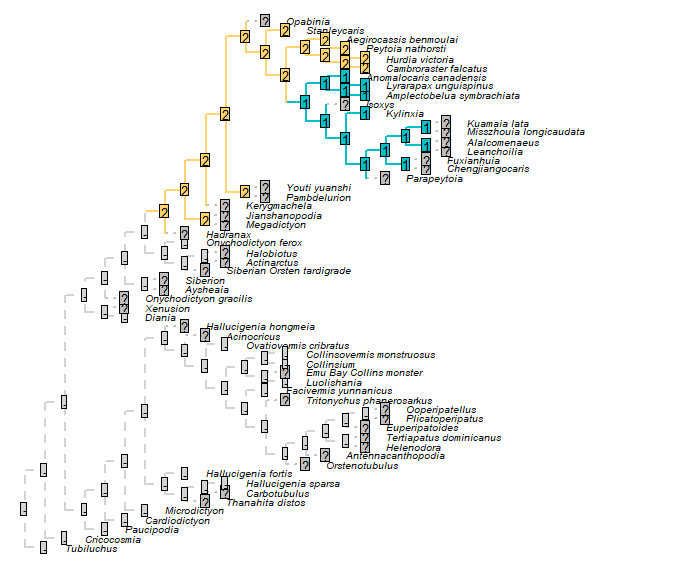


**Supplementary Figure 50: Cephalic/anterior appendages: Protocerebral appendages: Spine/spinules: Height**

- Inapplicable

1 comparable size to shaft

2 significantly larger than shaft

Hurdiids typically have very long main spines (endites) compared to the thickness of the shaft of the appendage. We treat the ventral spine series as distinct from lateral spine series (following [Dhungana & Smith 2021](#ref-Dhungana2021)), and limit this character to ventral spines.

Adapted from Zeng *et al.* ([2020](#ref-Zeng2020)) character 191; Aria & Caron ([2019](#ref-Aria2019)) character 90.

### [51] Spine/spinules: Accessory spines


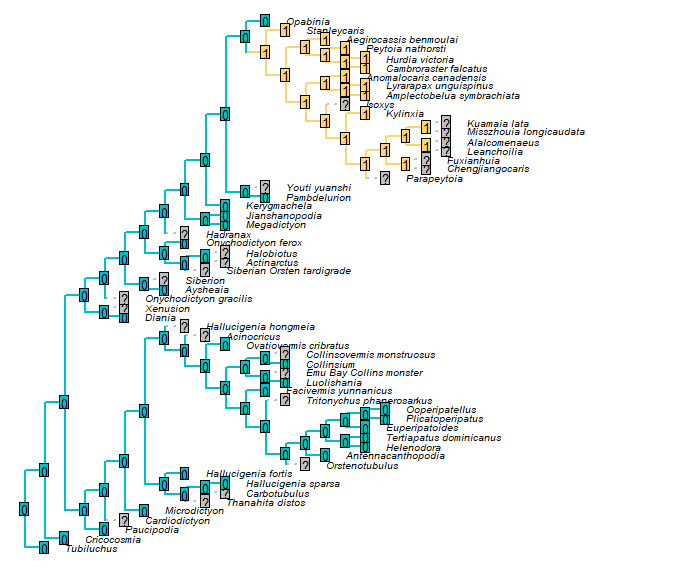


**Supplementary Figure 51: Cephalic/anterior appendages: Protocerebral appendages: Spine/spinules: Accessory spines**

0 absent

1 present

Accessory spines to the main ventral spines of the protocerebral spine series of many radiodonts.

*Youti yuanshi*: Coded ambiguous as protocerebral appendages appear to be in an early developmental stage (this study); adult morphology is uncertain.

### [52] Spine/spinules: Accessory spine distribution


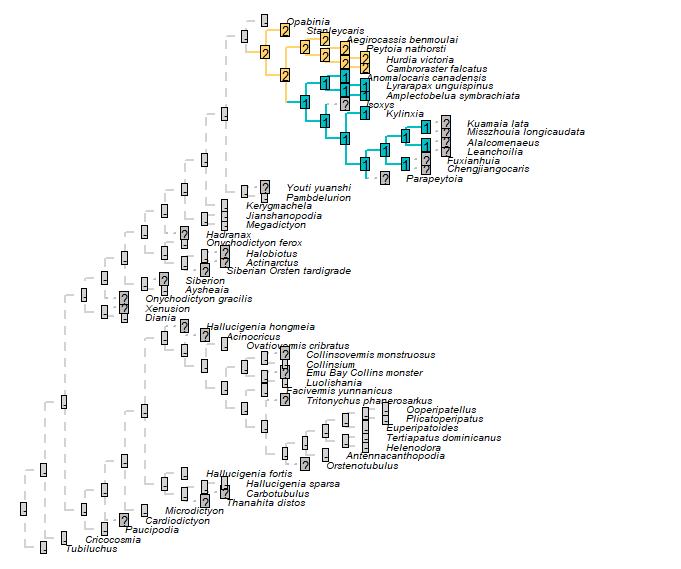


**Supplementary Figure 52: Cephalic/anterior appendages: Protocerebral appendages: Spine/spinules: Accessory spine distribution**

- Inapplicable

1 accessory spines originate near base of main spine

2 accessory spines regularly spaced along main spine

Hurdiids have accessory spines arranged in a regular series along the main spine, whereas e.g. *Anomalocaris* *canadensis* has accessory spines originating near the base of the main spine, giving a multifurcate appearance.

*Parapeytoia*: The serrated margins of the main spines of *Parapeytoia* ([Hou *et al.* 1995](#ref-Hou1995gff)) have been compared to megacheiran appendages (see [Budd 2021](#ref-Budd2021)). We conservatively code this character as ambiguous as the potential homology to accessory endite spines in radiodonts is unclear.

### [53] Spine/spinules: Alternation


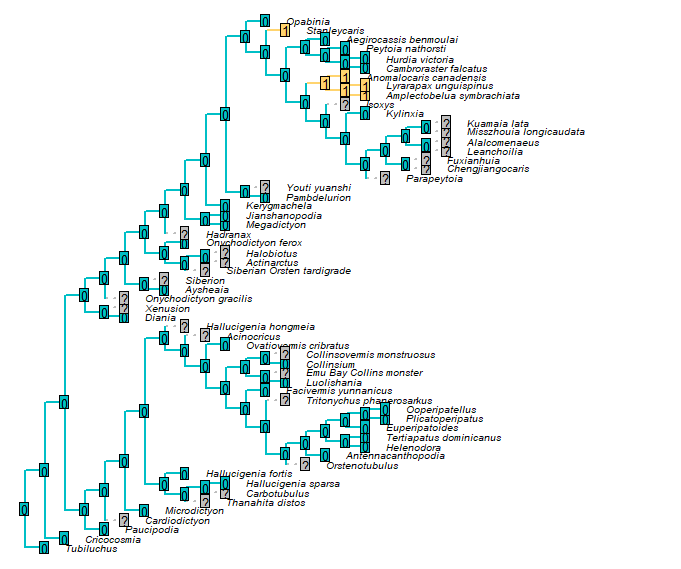


**Supplementary Figure 53: Cephalic/anterior appendages: Protocerebral appendages: Spine/spinules: Alternation**

0 no alternation in length

1 alternation in length from each spine to the next

Character 44 in Vinther *et al.* ([2014](#ref-Vinther2014)) and 41 in Moysiuk & Caron ([2019](#ref-Moysiuk2019)). The endites of certain anomalocaridid appendages alternate in length from podomere to podomere. Treated as neomorphic as alternation represents additional complexity in developmental control.

*Megadictyon*, *Jianshanopodia*, *Kerygmachela*, *Pambdelurion*, *Amplectobelua symbrachiata*, *Anomalocaris canadensis*, *Cambroraster falcatus*, *Hurdia victoria*, *Peytoia nathorsti*, *Aegirocassis benmoulai*, *Lyrarapax unguispinus*: Following Moysiuk & Caron ([2019](#ref-Moysiuk2019)).

*Kylinxia*: No alternation in spines ([O’Flynn *et al.* 2023](#ref-OFlynn2023cb)).

### [54] Spine/spinules: Width


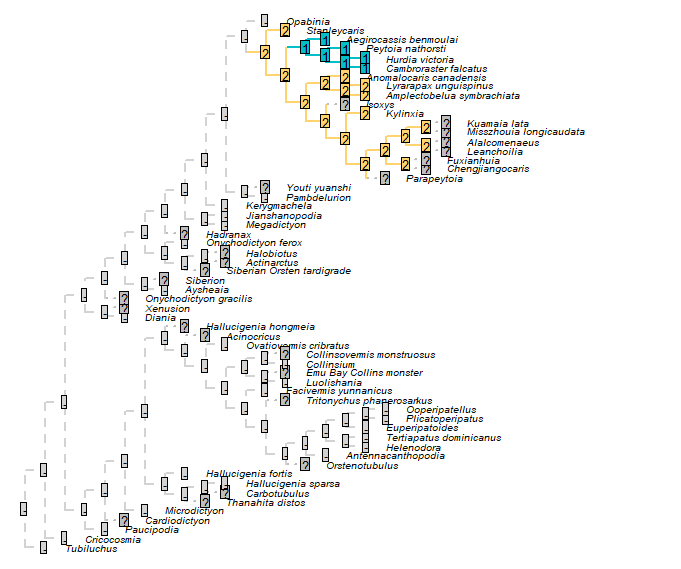


**Supplementary Figure 54: Cephalic/anterior appendages: Protocerebral appendages: Spine/spinules: Width**

- Inapplicable

1 comparable width of spine to podomere width

2 spine width significantly narrower

Spine series can be comparable in width to the base of the podomere/span the entire podomere or significantly narrower.

Character adapted from char. 192 in Zeng *et al.* ([2020](#ref-Zeng2020)); char. 108 in Aria & Caron ([2019](#ref-Aria2019)).

*Kylinxia*: Narrower than podomere: *Kylinxia* is closer to the condition in anomalocaridids than in hurdiids ([Zeng *et al.* 2020](#ref-Zeng2020)).

### [55] Spine/spinules: Base to tip thickness


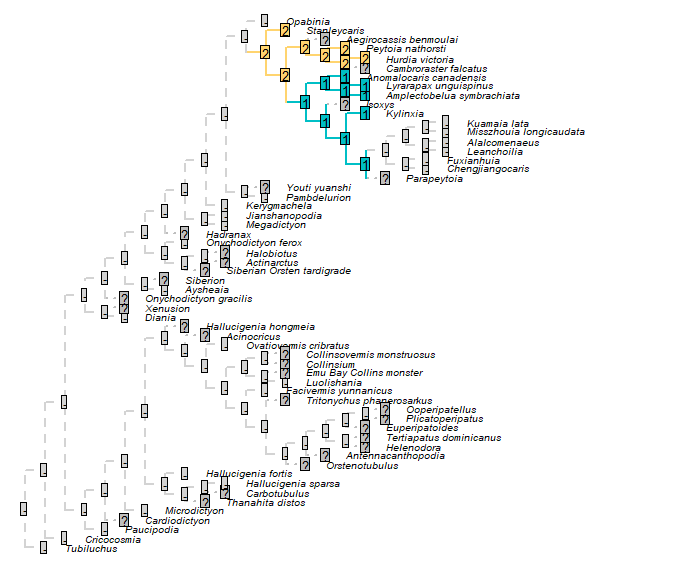


**Supplementary Figure 55: Cephalic/anterior appendages: Protocerebral appendages: Spine/spinules: Base to tip thickness**

- Inapplicable

1 no increase (e.g., *Anomalocaris*)

2 increase (e.g., *Hurdia*)

The large ventral endites of radiodonts can increase in size from base to tip e.g., *Hurdia*, *Peytoia*, *Stanleycaris*. Treated as transformational. See table 1 in Guo *et al.* ([2019](#ref-Guo2019)), and figure 2 in Pates *et al.* ([2019](#ref-Pates2019)).

*Amplectobelua symbrachiata*, *Anomalocaris canadensis*: No increase.

### [56] Multifurcate distal termination


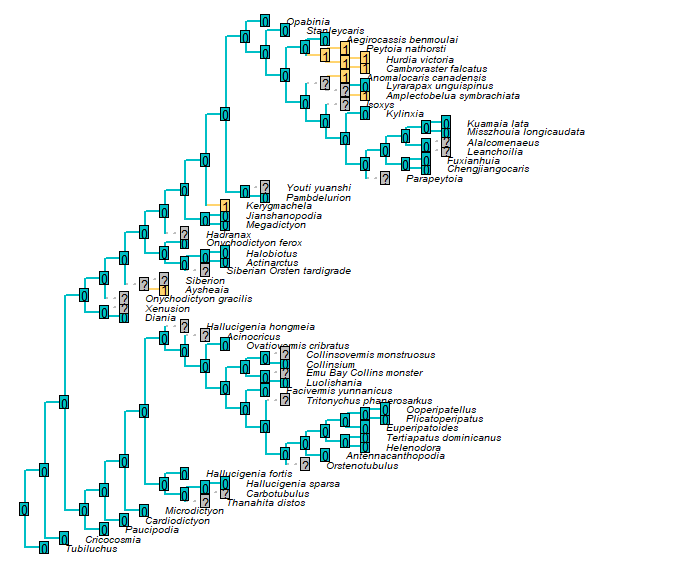


**Supplementary Figure 56: Cephalic/anterior appendages: Protocerebral appendage pair: Multifurcate distal termination**

0 absent

1 present

This neomorphic character describes the multifurcate termination observed in the protocerebral appendages of dinocaridids ([Budd 1996](#ref-Budd1996); [Daley *et al.* 2009](#ref-Daley2009); [Daley & Budd 2010](#ref-Daley2010); [Budd & Daley 2012](#ref-Budd2012); [Daley & Edgecombe 2014](#ref-Daley2014)) and certain lobopodians – such as *Aysheaia* ([Whittington 1978](#ref-Whittington1978)), *Megadictyon* ([Liu *et al.* 2007](#ref-Liu2007az)) and *Kerygmachela* ([Budd 1993](#ref-Budd1993), [1998*b*](#ref-Budd1998trse)) – but absent in *Onychodictyon* *ferox* ([Ou *et al.* 2012](#ref-Ou2012)).
Coded as inapplicable in tardigrades due to the extremely modification of the pre-ocular appendage into a stylet apparatus, which poses challenges to the identification of homologues of appendicular features.

Character 33 in Smith & Caron ([2015](#ref-Smith2015)) and 22 in Yang *et al.* ([2015](#ref-Yang2015)).

*Aegirocassis benmoulai*: “Terminal podomere stout, with pointed tip.” ([Van Roy *et al.* 2015](#ref-VanRoy2015)).

*Lyrarapax unguispinus*: Liu *et al.* ([2018](#ref-Liu2018nsr)) shows that the *Lyrarapax* appendage terminates in a distal claw, and does not have a multifurcate distal termination.

*Megadictyon*: *Megadictyon* protocerebral appendages end in a single claw (e.g. [Vannier *et al.* 2014](#ref-Vannier2014)), therefore have a single rather than multifurcate termination.

*Opabinia*: We interpret the claws of *Opabinia*’s protocerebral appendage as podomerous (see [Whittington 1975 figs 75, 79](#ref-Whittington1975)). The distalmost podomere terminates in a single point (e.g. [Whittington 1975 fig. 79](#ref-Whittington1975)), therefore we code the multifurcate termination as absent.

*Pambdelurion*: Vannier *et al.* ([2014](#ref-Vannier2014)) show *Pambdelurion*’s protocerebral appendage terminates in a single claw.

*Parapeytoia*: As the affinity of the anterior appendages of *Parapeytoia* is unclear, we code this character ambiguously – although there is no indication that the distalmost podomere is multifurcate (e.g. [Hou *et al.* 1995 fig. 12](#ref-Hou1995gff)).

*Youti yuanshi*: Coded ambiguous as protocerebral appendages appear to be in an early developmental stage (this study); adult morphology is uncertain.

### [57] Podomere differentiation


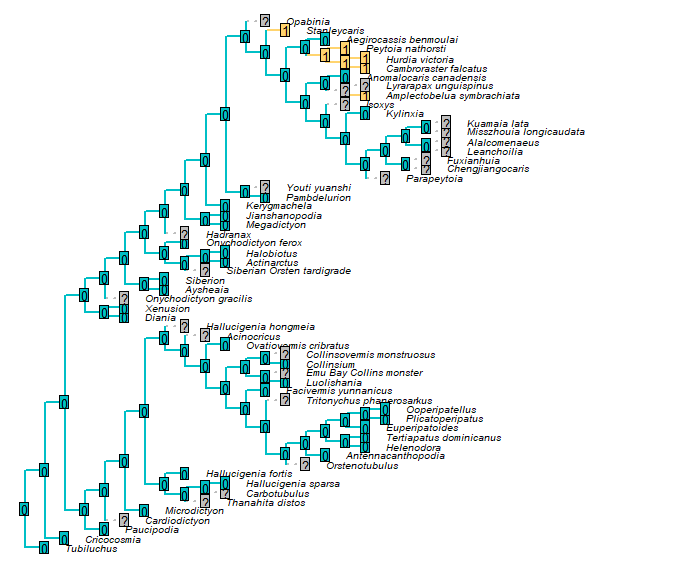


**Supplementary Figure 57: Cephalic/anterior appendages: Protocerebral appendages: Podomere differentiation**

0 no material differentiation of podomeres

1 strong differentiation of proximal from distal podomeres

Character 33 in Moysiuk & Caron ([2019](#ref-Moysiuk2019)). The segments of the first appendage pair are uniform in form (homonomous) along the length of the limb in *Anomalocaris*, whereas in *Hurdia* the segments of the distal and proximal sections are strongly distinct.

The peduncle and outer spines are not considered in this character. The character is treated as neomorphic, as differentiation is seen to reflect a greater degree of developmental and morphological specialization.

*Amplectobelua symbrachiata*, *Anomalocaris canadensis*, *Cambroraster falcatus*, *Hurdia victoria*, *Peytoia nathorsti*, *Aegirocassis benmoulai*, *Lyrarapax unguispinus*: Following Moysiuk & Caron ([2019](#ref-Moysiuk2019)).

*Opabinia*: The basal podomeres are poorly preserved ([Dhungana & Smith 2021](#ref-Dhungana2021)) hence we code as ambiguous.

*Youti yuanshi*: Conservatively coded as ambiguous to reflect possibility of later development of podomeres.

### [58] Distally tapering podomeres


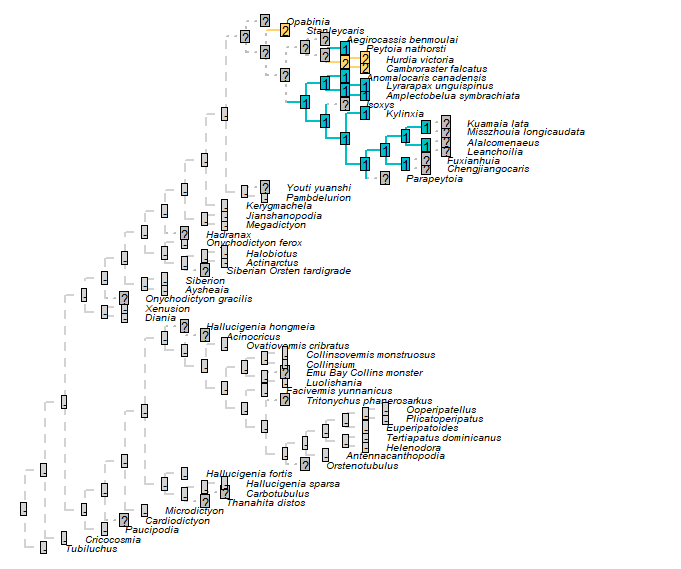


**Supplementary Figure 58: Cephalic/anterior appendages: Protocerebral appendages: Distally tapering podomeres**

- Inapplicable

1 Distal podomeres approximately uniform size

2 Distal podomere diameter strongly reducing distally

The distalmost podomeres of *Caryosyntrips*, *Hurdia* are differentiated and strongly reduce distally, resulting in “inward flexure” of these podomeres.

Treated as applicable even when podomeres are homonomous, as differentiation in size need not depend on differentiation of podomere morphology. Inapplicable in taxa that lack sclerotized protocerebral appendages.

Character 35 in Moysiuk & Caron ([2019](#ref-Moysiuk2019)).

*Amplectobelua symbrachiata*, *Cambroraster falcatus*, *Hurdia victoria*, *Peytoia nathorsti*, *Aegirocassis benmoulai*, *Lyrarapax unguispinus*: Following Moysiuk & Caron ([2019](#ref-Moysiuk2019)).

*Anomalocaris canadensis*: No significant change.

*Opabinia*: We interpret the claws of *Opabinia*’s protocerebral appendage as podomerous (see [Whittington 1975 figs 75, 79](#ref-Whittington1975)). The distal three podomeres are differentiated, and could be homologous to the differentiation of distal podomeres of certain hurdiids, however, given that hurdiid distal podomeres taper in diameter, and *Opabinia*’s terminal podomere is the largest, we code as uncertain for this character.

*Youti yuanshi*: Uncertain as adult morphology unknown; observed tapering (this study) may be a developmental phenomenon.

### [59] Kink


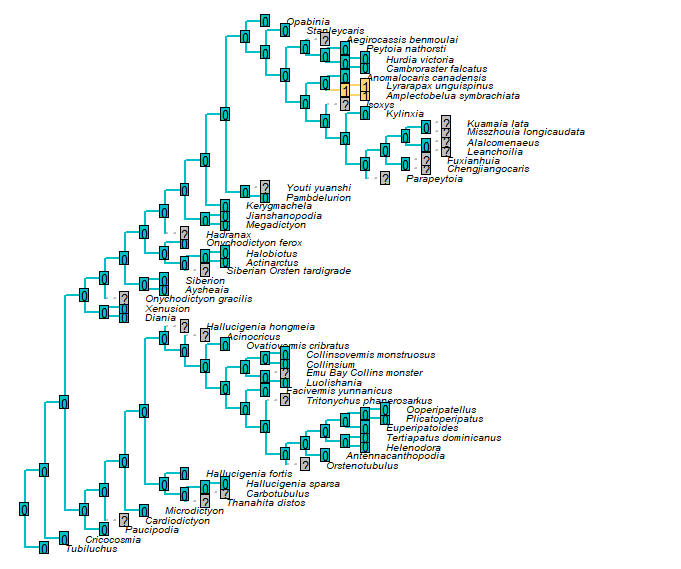


**Supplementary Figure 59: Cephalic/anterior appendages: Protocerebral appendages: Kink**

0 absent

1 present

In *Amplectobelua*, *Lyrarapax* and *Anomalocaris* *saron*, the distal appendage kinks outwards at a high angle relative to the appendage peduncle (shaft).

Character 36 in Moysiuk & Caron ([2019](#ref-Moysiuk2019)), following character 27 in Vinther *et al.* ([2014](#ref-Vinther2014)).

*Anomalocaris canadensis*: Unkinked ([Daley & Edgecombe 2014](#ref-Daley2014)), though kink present in *A. saron*.
Originally coded as kinked by Vinther *et al.* ([2014](#ref-Vinther2014)); updated to not kinked by Moysiuk & Caron ([2019](#ref-Moysiuk2019)).

*Youti yuanshi*: Coded ambiguous as protocerebral appendages appear to be in an early developmental stage (this study); adult morphology is uncertain.

### [60] Pincer


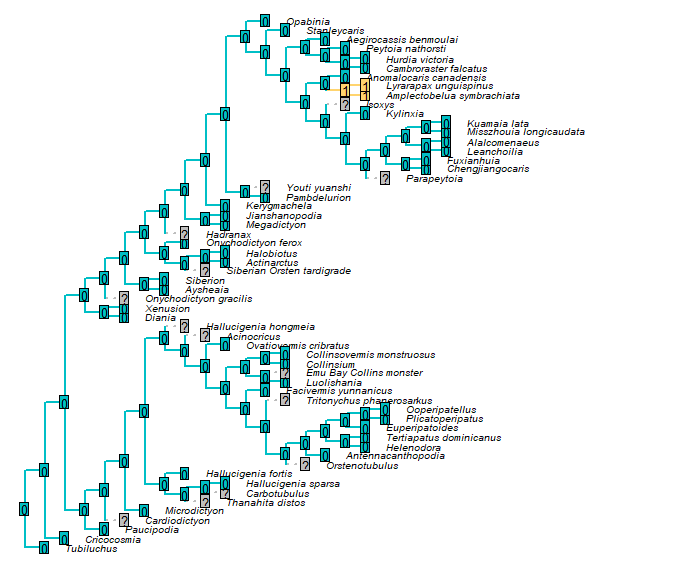


**Supplementary Figure 60: Cephalic/anterior appendages: Protocerebral appendages: Pincer**

0 absent

1 present

Character 35 in Vinther *et al.* ([2014](#ref-Vinther2014)) and 40 in Moysiuk & Caron ([2019](#ref-Moysiuk2019)). In *Amplectobelua* and *Lyrarapax*, a proximal endite projects forwards to oppose the distal endites, forming a “pincer” or “claw”.

*Amplectobelua symbrachiata*, *Anomalocaris canadensis*, *Cambroraster falcatus*, *Hurdia victoria*, *Peytoia nathorsti*, *Aegirocassis benmoulai*, *Lyrarapax unguispinus*: Following Moysiuk & Caron ([2019](#ref-Moysiuk2019)).

*Parapeytoia*: The affinity of the frontal appendages of *Parapeytoia* is unclear, hence we code this character as ambiguous although the ‘pincer’ of *Parapeytoia* is formed by distal endite with opposing curvature ([Hou *et al.* 1995](#ref-Hou1995gff)), rather than the proximal endite (such as in *Lyrarapax*).

*Youti yuanshi*: Coded ambiguous as protocerebral appendages appear to be in an early developmental stage (this study); adult morphology is uncertain.

### [61] Outer spines


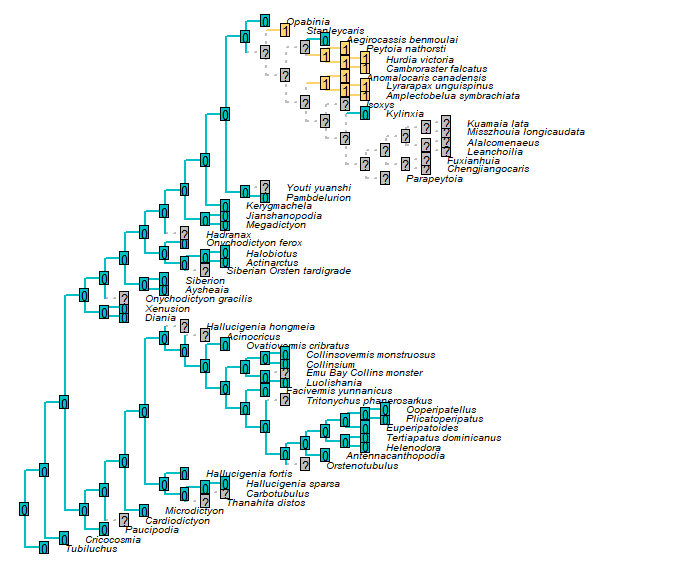


**Supplementary Figure 61: Cephalic/anterior appendages: Protocerebral appendages: Outer spines**

0 absent

1 present

Most radiodonts have outer spine series in addition to inner spine series on the protocerebral appendage ([Moysiuk & Caron 2019](#ref-Moysiuk2019)) also referred to as “dorsal” spines (e.g. [Zeng *et al.* 2020](#ref-Zeng2020)), typically the spines in this series are larger distal-ward. This spine series appears to be independent of the medial/ventral spine series ([Moysiuk & Caron 2019](#ref-Moysiuk2019)).

*Aegirocassis benmoulai*: Following Moysiuk & Caron ([2021](#ref-Moysiuk2021)).

*Youti yuanshi*: Coded ambiguous as protocerebral appendages appear to be in an early developmental stage (this study); adult morphology is uncertain.

### [62] Spine series tip orientation


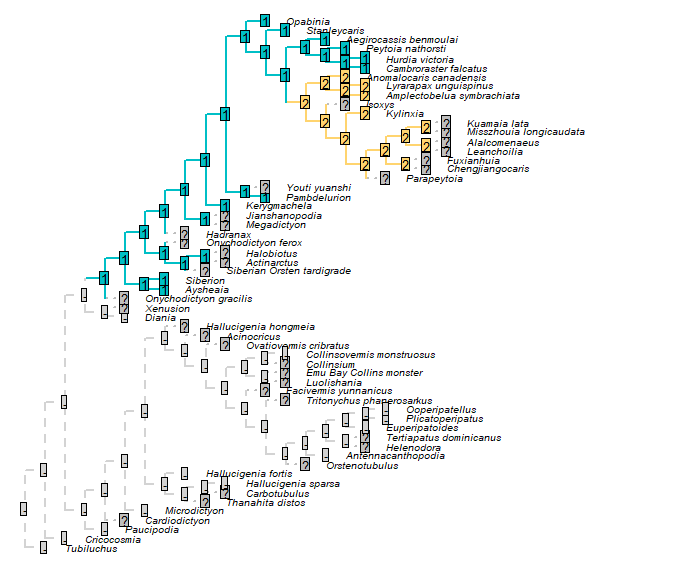


**Supplementary Figure 62: Cephalic/anterior appendages: Protocerebral appendages: Spine series tip orientation**

- Inapplicable

1 spine series point to appendage

2 spine series point outwards

The orientation of spine series are independent of the position of the spine series. In hurdiids, for example, the main enditic spines are in a ventral position, but spines curve such that the distal tips face the other appendage. In gilled lobopodians and *Caryosyntrips*, spine series point towards the other appendage. In *Anomalocaris*, the ventral spine series do not face the other appendage, but are straight and point outwards (ventrally).

*Youti yuanshi*: Coded ambiguous as protocerebral appendages appear to be in an early developmental stage (this study); adult morphology is uncertain.

### [63] Lateral spine series


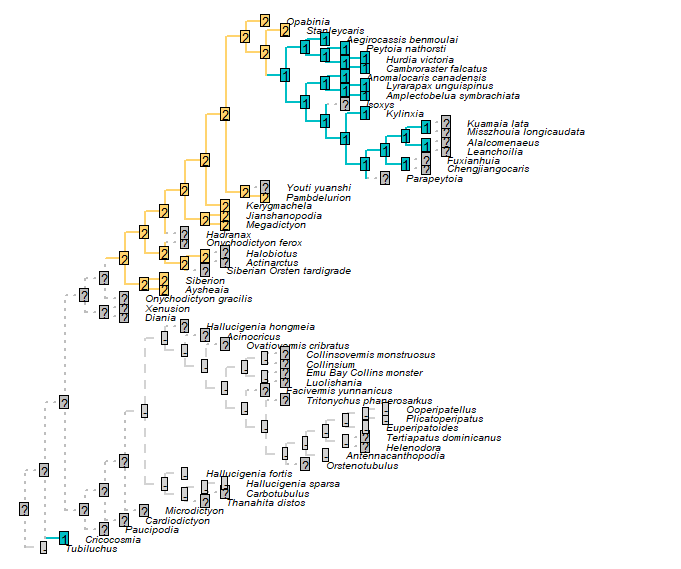


**Supplementary Figure 63: Cephalic/anterior appendages: Protocerebral spine series: Lateral spine series**

- Inapplicable

1 absent

2 present

Moysiuk & Caron ([2021](#ref-Moysiuk2021)) suggest that the laterally located gnathal spine series in e.g. *Caryosyntrips* is independent of the ventral enditic spine series observed in many radiodonts. As the spine series of e.g. gilled lobopodians are also laterally located, we suggest these spine series maybe positionally homologous.

*Cricocosmia*: Following Dhungana ([2024](#ref-Dhungana2024)).

*Youti yuanshi*: Coded ambiguous as protocerebral appendages appear to be in an early developmental stage (this study); adult morphology is uncertain.

### [64] Accessory gnathal spines


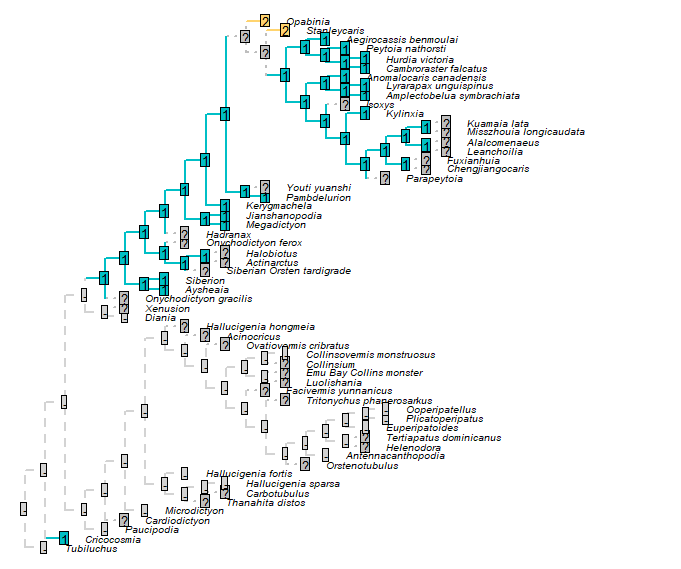


**Supplementary Figure 64: Cephalic/anterior appendages: Protocerebral appendages: Accessory gnathal spines**

- Inapplicable

1 absent

2 present

Adapted from char. 55 in Moysiuk & Caron ([2021](#ref-Moysiuk2021)), who note that auxiliary spines are present on the lateral spines/gnathites of *Stanleycaris* and cf. *Peytoia*.

*Cricocosmia*: Following Dhungana ([2024](#ref-Dhungana2024)).

*Youti yuanshi*: Coded ambiguous as protocerebral appendages appear to be in an early developmental stage (this study); adult morphology is uncertain.

### [65] Nature of post-ocular lobopodous inner branch


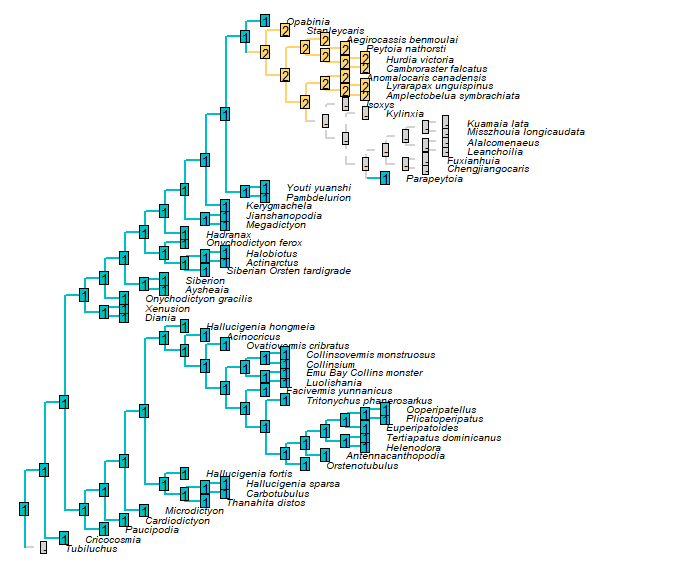


**Supplementary Figure 65: Cephalic/anterior appendages: Nature of post-ocular lobopodous inner branch**

- Inapplicable

1 cylindrical/subconical appendage

2 laterally expanded swimming flap

The cylindrical ambulacral lobopodous leg characteristic of lobopodians is also found in *Opabinia* ([Budd 1996](#ref-Budd1996); [Budd & Daley 2012](#ref-Budd2012)), *Kerygmachela* ([Budd 1993](#ref-Budd1993), [1998*b*](#ref-Budd1998trse)), *Pambdelurion* ([Budd 1998*a*](#ref-Budd1998ar)) and *Aegirocassis* ([Van Roy *et al.* 2015](#ref-VanRoy2015)). Coding for radiodontans follows Van Roy *et al.* ([2015](#ref-VanRoy2015)).

Character 23 from Smith & Caron ([2015](#ref-Smith2015)) and 11 in Yang *et al.* ([2015](#ref-Yang2015)).

*Cricocosmia*: Following Dhungana ([2024](#ref-Dhungana2024)).

### [66] Deutocerebral limb pair structurally differentiated from trunk appendages


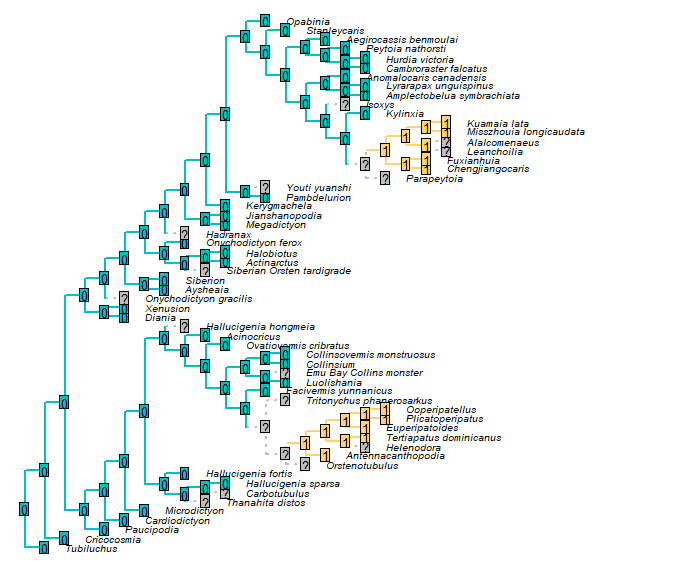


**Supplementary Figure 66: Cephalic/anterior appendages: Deutocerebral limb pair structurally differentiated from trunk appendages**

0 undifferentiated, or differentiated in size only

1 structurally differentiated

There are various taxa in which the deutocerebral appendage pair is morphologically differentiated from the rest of the trunk appendages (see references in [Liu & Dunlop 2014](#ref-Liu2014ppp)). For example, *Antennacanthopodia* has a second set of antenna-like limbs that are morphologically distinct from the walking legs ([Ou *et al.* 2011](#ref-Ou2011)). The first pair of legs in Tardigrada is serially homologous with the deutocerebral segment of Euarthropoda ([Mayer *et al.* 2013*b*](#ref-Mayer2013po)), and thus is not structurally different from the rest of the trunk appendages. The deutocerebral jaws of Onychophora are significantly modified relative to the rest of the appendages in the body ([Eriksson *et al.* 2010](#ref-Eriksson2010); [Sena Oliveira & Mayer 2013](#ref-Oliveira2013)). In Euarthropoda, this morphological differentiation is generally expressed in the presence of an antenniform (e.g. [Edgecombe & Ramsköld 1999](#ref-Edgecombe1999); [Ma *et al.* 2012*a*](#ref-Ma2012n); [Yang *et al.* 2013](#ref-Yang2013)) or raptorial ([Chen *et al.* 2004](#ref-Chen2004); [Haug *et al.* 2012*c*](#ref-Haug2012p); [Tanaka *et al.* 2013](#ref-Tanaka2013)) deutocerebral appendage. The second leg pair of hallucishaniid taxa are not differentiated from their neighbours ([Ramsköld & Chen 1998](#ref-Ramskold1998)) and are therefore coded as undifferentiated; the trunk limbs are instead divided into two morphological zones.

Character 24 in Smith & Caron ([2015](#ref-Smith2015)) and 14 in Yang *et al.* ([2015](#ref-Yang2015)).

*Amplectobelua symbrachiata*: The first three flaps are reduced, but the deutocerebral appendage is not morphologically distinct ([Cong *et al.* 2017](#ref-Cong2017)). The gnathobase-like structures ([Cong *et al.* 2017](#ref-Cong2017)) are captured in a separate character.

*Anomalocaris canadensis*: Daley & Edgecombe ([2014](#ref-Daley2014)) reported the presence of a smaller set of flaps in proximity with the putative head region of *Anomalocaris* *canadensis*; given that this differentiation is expressed in size, rather than structural identity, we score the deutocerebral limbs as undifferentiated in *Anomalocaris*.

*Carbotubulus*: Because the head of *Carbotubulus* is not preserved ([Haug *et al.* 2012*b*](#ref-Haug2012cb)), the identity of the limbs is unclear and this character is coded as ambiguous.

*Cardiodictyon*: The second appendage pair of *Cardiodictyon* does not seem to be differentiated ([Liu *et al.* 2008*b*](#ref-Liu2008app)).

*Youti yuanshi*: Preservation insufficient to evaluate potential differentiation in adult; and appendages may be in an early developmental stage, with differentiation occurring late in development (this study).

### [67] Nature of sclerotized first post-ocular (deutocerebral) appendage


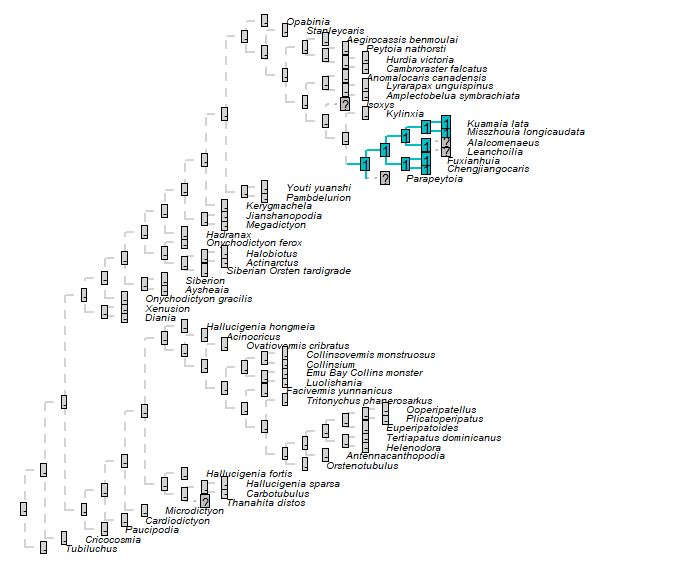


**Supplementary Figure 67: Cephalic/anterior appendages: Nature of sclerotized first post-ocular (deutocerebral) appendage**

- Inapplicable

1 antenniform with distinct podomeres

2 short great-appendage

This character, adapted from char. 25 from Smith & Caron ([2015](#ref-Smith2015)) and char. 12 from Yang *et al.* ([2015](#ref-Yang2015)), has been re-formulated into two separate characters on the basis that the arthropodization of the first post-ocular appendage is not independent from the arthropodization of the subsequent trunk appendages. This formulation makes it unnecessary to distinguish taxa with differentiated deutocerebral appendages (cf. char. 24 in [Smith & Caron 2015](#ref-Smith2015)).

### [68] Nature of lobopodous first post-ocular (deutocerebral) appendage


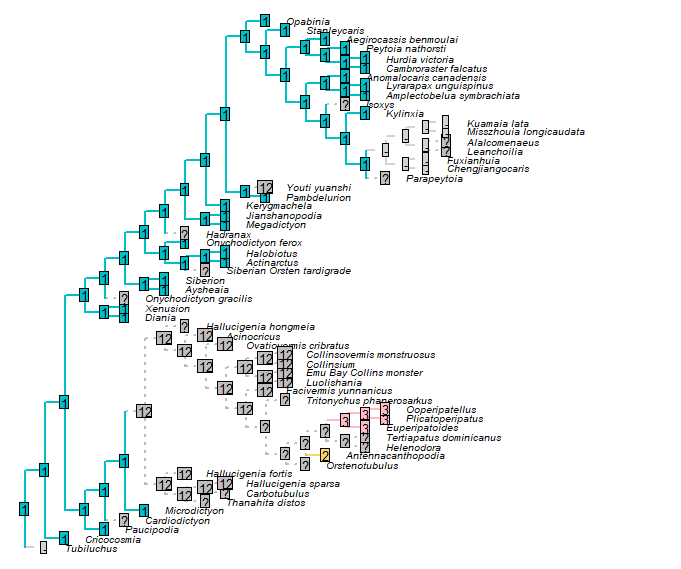


**Supplementary Figure 68: Cephalic/anterior appendages: Nature of lobopodous first post-ocular (deutocerebral) appendage**

- Inapplicable

1 ambulatory

2 sensorial

3 masticatory, with sclerotized jaw

The first post-ocular limb is not observable in *Tertiapatus* or *Ilyodes* ([Poinar 2000](#ref-Poinar2000); [Haug *et al.* 2012*b*](#ref-Haug2012cb)), and is thus scored as ambiguous. It is difficult to evaluate the role of the slender appendages of *Hallucigenia* ([Ramsköld & Chen 1998](#ref-Ramskold1998); [Smith & Caron 2015](#ref-Smith2015)) and the cirrate post-ocular appendages of *Luolishania*, *Collinsium*, *Acinocricus* and the Collins monsters ([Ma *et al.* 2009](#ref-Ma2009); [García-Bellido *et al.* 2013](#ref-Garcia2013); [Yang *et al.* 2015](#ref-Yang2015); [Caron & Aria 2020](#ref-Caron2020)); as such, these are coded as ambiguous for states “ambulatory” and “sensorial”.

Character 25 from Smith & Caron ([2015](#ref-Smith2015)) and 12 from Yang *et al.* ([2015](#ref-Yang2015)).

*Antennacanthopodia*: The ‘second antenna’ of *Antennacanthopodia* ([Ou *et al.* 2011](#ref-Ou2011)) is interpreted as a sensorial appendage.

*Cricocosmia*: Following Dhungana ([2024](#ref-Dhungana2024)).

### [69] Inner blade of deutocerebral jaw with diastema


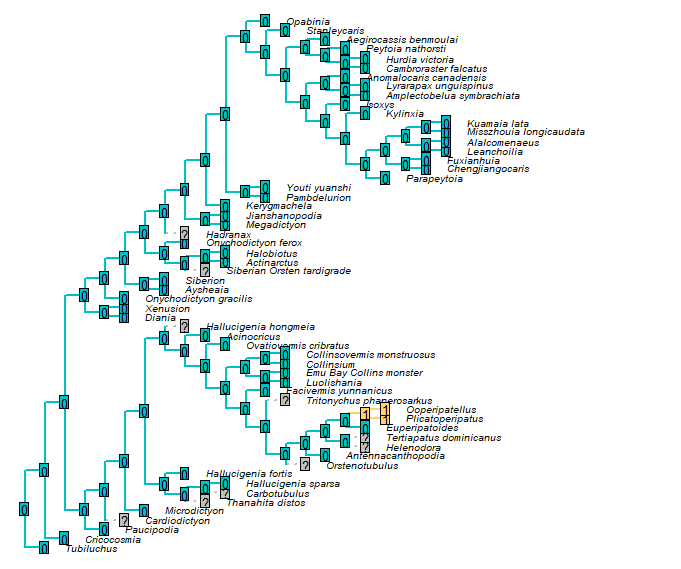


**Supplementary Figure 69: Cephalic/anterior appendages: Inner blade of deutocerebral jaw with diastema**

0 absent

1 present

See character 13 in Yang *et al.* ([2015](#ref-Yang2015)).
Present in Peripatidae ([Sena Oliveira & Mayer 2013](#ref-Oliveira2013)), but absent in *Euperipatoides* ([Smith & Ortega-Hernández 2014](#ref-Smith2014)).

### [70] Nature of lobopodous second post-ocular (tritocerebral) appendage


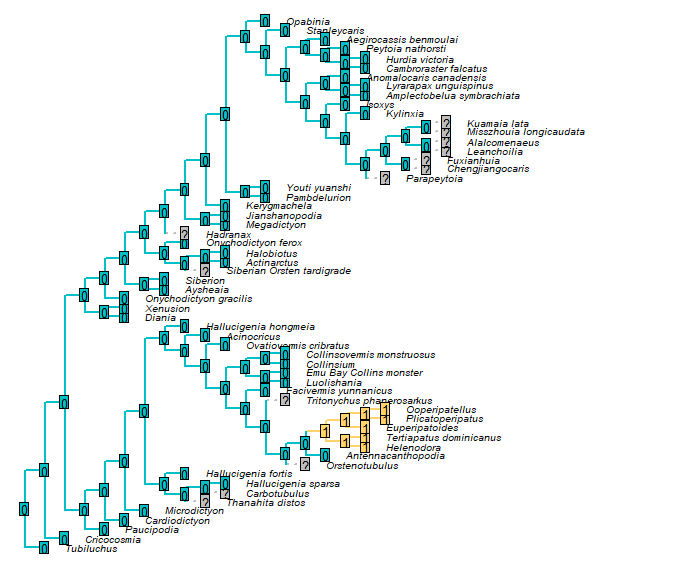


**Supplementary Figure 70: Cephalic/anterior appendages: Nature of lobopodous second post-ocular (tritocerebral) appendage**

0 undifferentiated

1 specialized papilla

As with character relating to the nature of the deutocerebral appendages, this character is coded as a separate character in taxa with lobopodous and with arthropodized appendages. *Ilyodes* ([Haug *et al.* 2012*b*](#ref-Haug2012cb)), *Tertiapatus* ([Poinar 2000](#ref-Poinar2000)) and extant onychophorans are interpreted as bearing paired oral papillae.

Adapted from character 15 in Yang *et al.* ([2015](#ref-Yang2015)).

*Chengjiangocaris*, *Fuxianhuia*, *Leanchoilia*, *Alalcomenaeus*, *Misszhouia longicaudata*, *Kuamaia lata*: Inapplicable in upper stem euarthropods with arthropodized second appendage; coded with ambiguous token as character is neomorphic.

### [71] Nature of arthropodized second post-ocular (tritocerebral) appendage


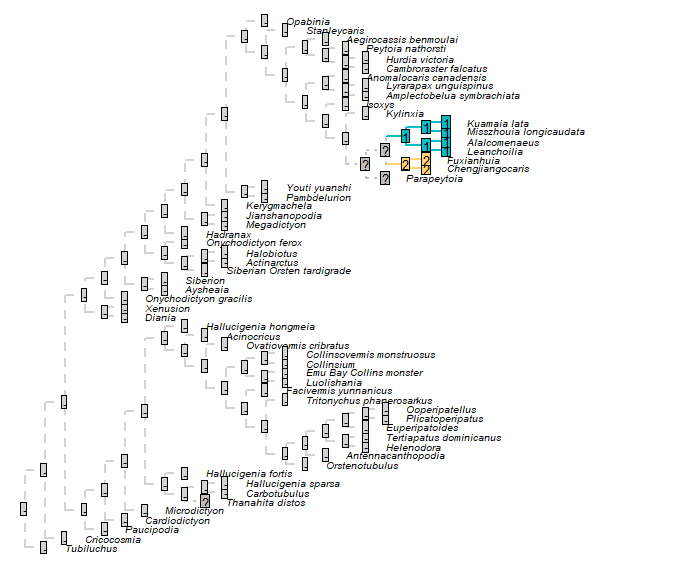


**Supplementary Figure 71: Cephalic/anterior appendages: Nature of arthropodized second post-ocular (tritocerebral) appendage**

- Inapplicable

1 ambulatory limb with distinct podomeres

2 specialized post-antennal appendage

The tritocerebral appendages of fuxianhuiids are reduced for a sweep-feeding function ([Yang *et al.* 2013](#ref-Yang2013)).

Adapted from character 15 in Yang *et al.* ([2015](#ref-Yang2015)).

## Trunk region

### [72] Epidermal segmentation


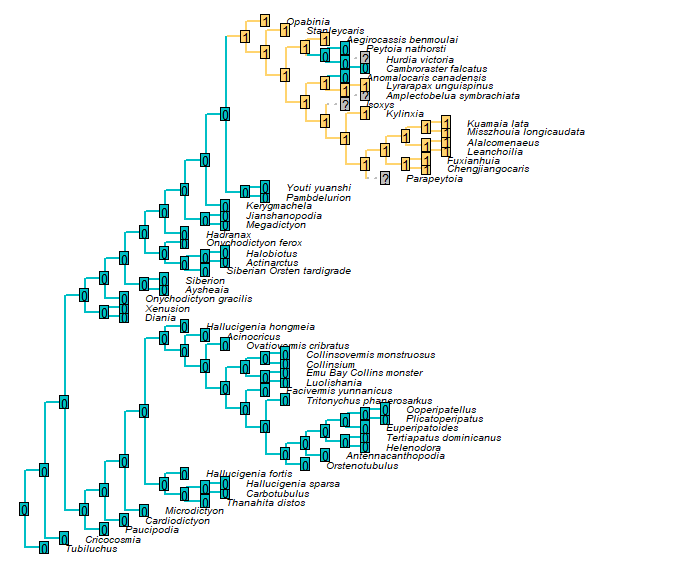


**Supplementary Figure 72: Trunk region: Epidermal segmentation**

0 absent

1 present

Epidermal segmentation is a distinguishing feature of Euarthropoda ([Budd 2001*a*](#ref-Budd2001za); [Edgecombe 2009](#ref-Edgecombe2009)). Although the body of Onychophora and Tardigrada is metamerically organized, both at the level of segment polarity gene expression ([Gabriel & Goldstein 2007](#ref-Gabriel2007); [Eriksson *et al.* 2009](#ref-Eriksson2009)) and musculature (e.g. [Halberg *et al.* 2009](#ref-Halberg2009); [Marchioro *et al.* 2013](#ref-Marchioro2013)), this pattern is not expressed on the epidermis; we thus score it as absent in these phyla. Epidermal segmentation is not evident in most radiodontans (e.g. [Daley & Edgecombe 2014](#ref-Daley2014)), which we score absent.

Character 25 in Daley *et al.* ([2009](#ref-Daley2009)), 34 in Smith & Caron ([2015](#ref-Smith2015)) and 32 in Yang *et al.* ([2015](#ref-Yang2015)).

*Aegirocassis benmoulai*: Interpreted as present by Moysiuk & Caron ([2022](#ref-Moysiuk2022)).

*Hurdia victoria*: The single complete specimen does not conclusively establish the presence or absence of epidermal segmentation ([Daley *et al.* 2009](#ref-Daley2009)).

*Lyrarapax unguispinus*: Interpreted as present by Moysiuk & Caron (2022).

*Opabinia*: Coded as present since has discrete body segments separated by furrows ([Budd 1996](#ref-Budd1996); [Zhang & Briggs 2007](#ref-Zhang2007); [Budd & Daley 2012](#ref-Budd2012)).

*Stanleycaris*: Moysiuk & Caron ([2022](#ref-Moysiuk2022)) observe segmental boundaries (arguably implying arthrodization) in the dorsal trunk cuticle, though these are not apparent on the ventral surface; this recalls the ventrally flexible configuration of *Opabinia*.

*Youti yuanshi*: As epidermal segmentation is evident at all stages of ontogeny, we consider its absence here as denoting an absence in the eventual adult form.

### [73] Dorsal integument sclerotized and connected by arthrodial membranes


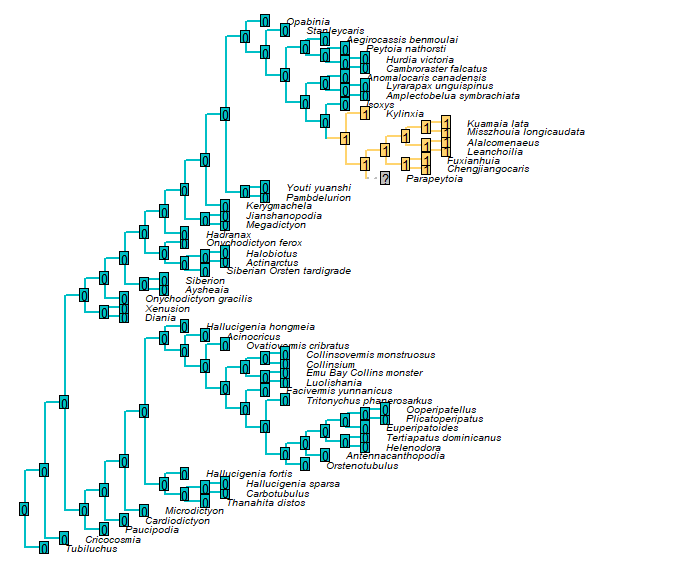


**Supplementary Figure 73: Trunk region: Dorsal integument sclerotized and connected by arthrodial membranes**

0 absent

1 present

The development of sclerotized tergal plates connected by arthrodial membranes is distinctive of body arthrodization, and thus exclusive to Euarthropoda ([Edgecombe & Ramsköld 1999](#ref-Edgecombe1999); [Haug *et al.* 2012*c*](#ref-Haug2012p); [Yang *et al.* 2013](#ref-Yang2013)).

Character 35 in Smith & Caron ([2015](#ref-Smith2015)), 33 in Yang *et al.* ([2015](#ref-Yang2015)).

*Actinarctus*: Although some heterotardigrades possess dorsal plates (e.g. [Nelson 2002](#ref-Nelson2002); [Marchioro *et al.* 2013](#ref-Marchioro2013)), these are not connected by arthrodial membranes. We thus score *Actinarctus* as absent for this character.

### [74] Sternites connected by arthrodial membranes


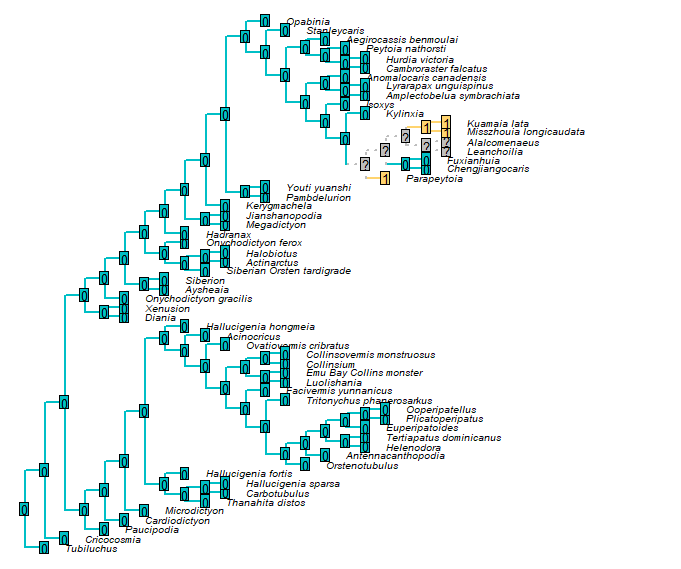


**Supplementary Figure 74: Trunk region: Sternites connected by arthrodial membranes**

0 absent

1 present

Sternites – ventral sclerotized plates – are a key feature of most Euarthropoda, and are well documented in Artiopoda (e.g. [Whittington 1993](#ref-Whittington1993); [Edgecombe & Ramsköld 1999](#ref-Edgecombe1999); [Ortega-Hernández & Brena 2012](#ref-Ortega2012)). Sternites are notably absent in Fuxianhuiida ([Chen *et al.* 1995*c*](#ref-Chen1995s); [Waloszek *et al.* 2005](#ref-Waloszek2005); [Bergström *et al.* 2008](#ref-Bergstrom2008); [Yang *et al.* 2013](#ref-Yang2013)), even though these taxa have a sclerotized dorsal exoskeleton. We code sternites as uncertain in leanchoiliids.

Character 36 in Smith & Caron ([2015](#ref-Smith2015)) and 34 in Yang *et al.* ([2015](#ref-Yang2015)).

*Isoxys*: Trunk not arthrodized ([Zhang *et al.* 2023](#ref-Zhang2023)).

*Kylinxia*: Following Zeng *et al.* ([2020](#ref-Zeng2020)).

### [75] Annulations


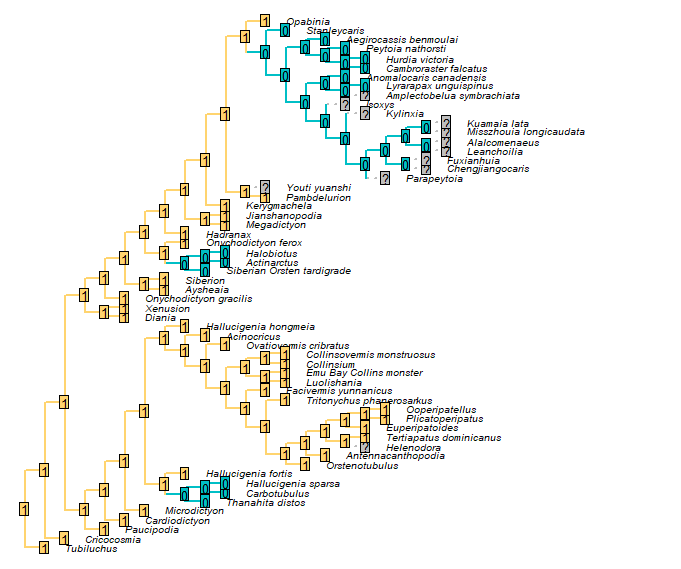


**Supplementary Figure 75: Trunk region: Annulations**

0 absent

1 present

Annulations are repeated superficial integument rings.

Character 26 in Daley *et al.* ([2009](#ref-Daley2009)), 37 in Smith & Caron ([2015](#ref-Smith2015)) and 36 in Yang *et al.* ([2015](#ref-Yang2015)).

*Chengjiangocaris*, *Fuxianhuia*, *Leanchoilia*, *Alalcomenaeus*, *Misszhouia longicaudata*, *Kuamaia lata*: Coded ambiguous as sclerotization of trunk assumed to overprint evidence of annulation.

*Antennacanthopodia*: Present on limbs; it is unclear whether the trunk was annulated, due to effaced preservation ([Ou *et al.* 2011](#ref-Ou2011)).

*Carbotubulus*: A taphonomic absence can be discounted because annulations are preserved in co-occurring specimens of *Ilyodes* ([Haug *et al.* 2012*b*](#ref-Haug2012cb)).

*Facivermis yunnanicus*: Annulated trunk and limbs ([Howard *et al.* 2020](#ref-Howard2020)).

*Hallucigenia sparsa*: Absent ([Smith & Caron 2015](#ref-Smith2015)).

*Ovatiovermis cribratus*: Fine epidermal annuli between limb pairs ([Caron & Aria 2017](#ref-Caron2017)).

*Pambdelurion*: Present on limbs; it is unclear whether the trunk was annulated, due to effaced preservation ([Budd 1998*a*](#ref-Budd1998ar)).

*Youti yuanshi*: Coded ambiguous. Onychophoran larval stages can lack evidence of annulations that are present in adults ([Walker & Tait 2004](#ref-Walker2004)).

### [76] Anterior projection of trunk lacking annulations


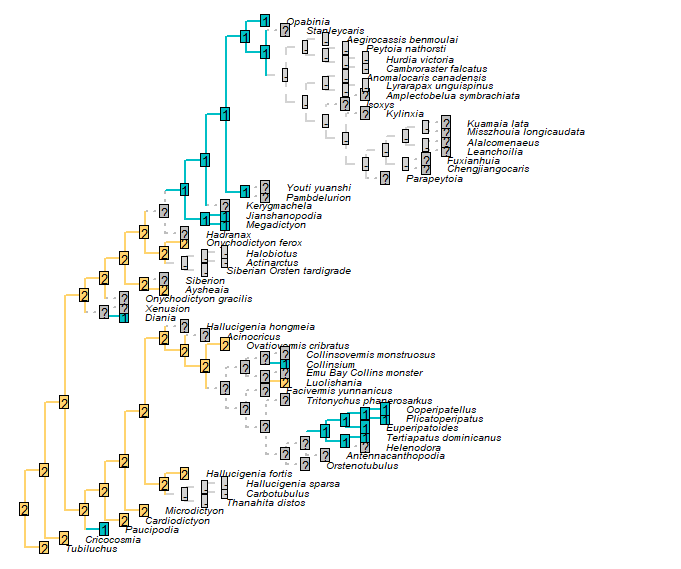


**Supplementary Figure 76: Trunk region: Anterior projection of trunk lacking annulations**

- Inapplicable

1 annulations continue for full length of trunk

2 differentiated anterior region of trunk lacking annulations

The anterior trunk of *Aysheaia* and *Onychodictyon* *ferox* is differentiated into a stout “proboscis”, distinct from the trunk by virtue of its shape and its lack of annulations ([Ou *et al.* 2012](#ref-Ou2012)). The anterior introvert of *Tubiluchus* and *Cricocosmia* is differentiated in a similar fashion, to a greater or lesser extent ([Calloway 1975](#ref-Calloway1975); [Kirsteuer 1976](#ref-Kirsteuer1976); [Han *et al.* 2007](#ref-Han2007app)). The bulbous heads of *Hallucigenia* *fortis*, *Microdictyon*, *Cardiodictyon* and *Luolishania* also lack annulations ([Chen *et al.* 1995*a*](#ref-Chen1995bnmns); [Ma *et al.* 2009](#ref-Ma2009), [2012*b*](#ref-Ma2012asd); [Liu & Dunlop 2014](#ref-Liu2014ppp)). In contrast, annulations continue to the tip of the head in *Paucipodia*, *Onychodictyon* *gracilis*, and *Diania* ([Chen *et al.* 1995*b*](#ref-Chen1995trse); [Hou *et al.* 2004](#ref-Hou2004); [Liu *et al.* 2008*b*](#ref-Liu2008app); [Ma *et al.* 2014*a*](#ref-Ma2014jsp); [Ou & Mayer 2018](#ref-Ou2018)).

Character 39 in Smith & Caron ([2015](#ref-Smith2015)).

*Tertiapatus dominicanus*, *Euperipatoides*, *Plicatoperipatus*, *Ooperipatellus*: Annulations continue to the anterior extremity of the onychophoran trunk.

*Facivermis yunnanicus*: Preservation inadequate to evaluate ([Howard *et al.* 2020](#ref-Howard2020)).

*Megadictyon*, *Jianshanopodia*: Annulations continue on anterior limbs and the terminal position of the mouth ([Vannier *et al.* 2014](#ref-Vannier2014)), suggesting that the anterior trunk had annulations.

*Kerygmachela*: Previously coded as present based on the presence of annulations on the first appendages and up to the mouth ([Budd 1998*b*](#ref-Budd1998trse)); but under the reinterpretation of Park *et al.* ([2018](#ref-Park2018)), the mouth is in a ventral position, making it unclear whether the potentially sub-terminal anterior part of the trunk is annulated. Coded ambiguous accordingly.

*Onychodictyon gracilis*: Coded ambiguous, given the uncertain preservation; see for example Liu *et al.* ([2008*b*](#ref-Liu2008app)), fig. 2A1–5.

*Opabinia*: The anterior frontal process of *Opabinia* is annulated, implying that annulations are present to the anterior head region (see [Whittington 1975](#ref-Whittington1975)).

*Ovatiovermis cribratus*: Proboscis seemingly without annulations ([Caron & Aria 2017](#ref-Caron2017)).

### [77] Organization of trunk annulation


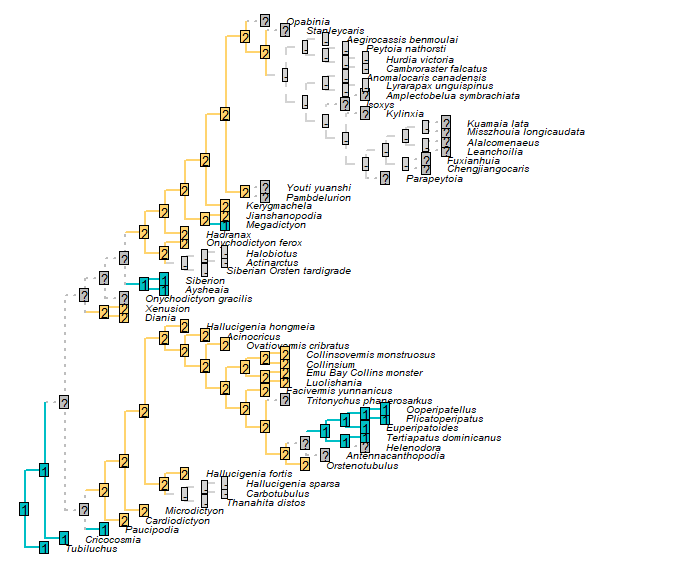


**Supplementary Figure 77: Trunk region: Organization of trunk annulation**

- Inapplicable

1 homonomous

2 heteronomous

This character distinguishes between annulation patterns that are uniform along the length of the trunk (homonomous) from those which display serially repeated differentiated fields (heteronomous), usually associated with the location of limbs.

Character: 29 in Liu *et al.* ([2011](#ref-Liu2011)); 27 in Daley *et al.* ([2009](#ref-Daley2009)); 40 in Smith & Caron ([2015](#ref-Smith2015)) and 38 in Yang *et al.* ([2015](#ref-Yang2015)).

*Kerygmachela*: *Jianshanopodia* exhibits regions of narrower annulations between appendages ([Liu *et al.* 2006](#ref-Liu2006)), so is coded as heteronomous.

*Megadictyon*: Annulations in *Megadictyon* appear regular ([Liu *et al.* 2007](#ref-Liu2007az)), so this taxon is coded as homonomous.

*Pambdelurion*: We code *Pambdelurion* as uncertain, as the trunk is not adequately preserved to make a confident assignation ([Budd 1998*a*](#ref-Budd1998ar); [Young & Vinther 2017](#ref-Young2017)).

### [78] Branching of annular rings


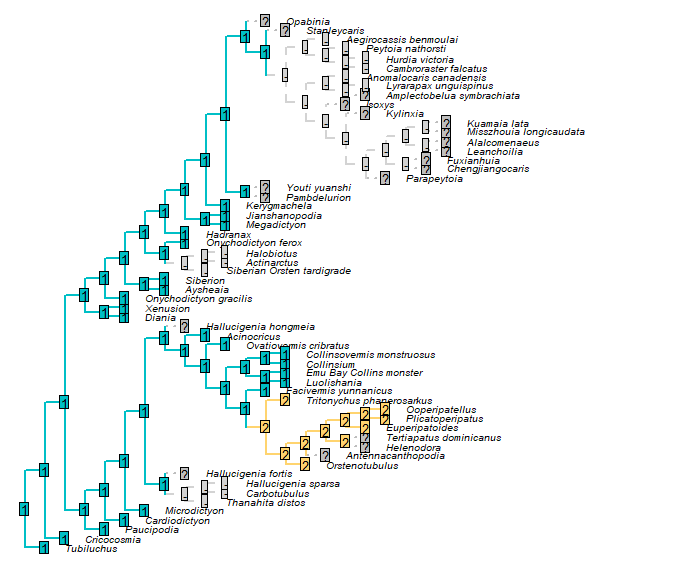


**Supplementary Figure 78: Trunk region: Branching of annular rings**

- Inapplicable

1 unbranched

2 branched

Unbranched in *Aysheaia*, *Siberion*, *Onychodictyon*, *Diania*, *Xenusion*, *Paucipodia*, *Microdictyon*, *Luolishania*, the Collins Monsters, *Acinocricus*, *Jianshanopodia*, *Hadranax* and *Kerygmachela* ([Whittington 1978](#ref-Whittington1978); [Conway Morris & Robison 1988](#ref-ConwayMorris1988); [Dzik & Krumbiegel 1989](#ref-Dzik1989); [Chen *et al.* 1995*a*](#ref-Chen1995bnmns); [Budd & Peel 1998](#ref-Budd1998p); [Hou *et al.* 2004](#ref-Hou2004); [Liu *et al.* 2006](#ref-Liu2006), [2008*b*](#ref-Liu2008app); [Ma *et al.* 2009](#ref-Ma2009), [2014*a*](#ref-Ma2014jsp); [Dzik 2011](#ref-Dzik2011); [Ou *et al.* 2012](#ref-Ou2012); [García-Bellido *et al.* 2013](#ref-Garcia2013); [Yang *et al.* 2015](#ref-Yang2015); [Caron & Aria 2020](#ref-Caron2020)); branched in *Orstenotubulus*, onychophorans (i.e. anastomosing plicae) and the Orsten-type lobopodian segment ([Maas *et al.* 2007](#ref-Maas2007csb); [Sena Oliveira *et al.* 2014](#ref-Oliveira2014)); ambiguous in *Megadictyon*, *Antennacanthopodia* and *Tertiapatus* ([Poinar 2000](#ref-Poinar2000); [Liu *et al.* 2007](#ref-Liu2007az); [Ou *et al.* 2011](#ref-Ou2011)).

Character 51 in Zhang *et al.* ([2016](#ref-Zhang2016)).

*Facivermis yunnanicus*: No branching observed ([Howard *et al.* 2020](#ref-Howard2020)).

*Megadictyon*: No indication of branching in Ramsköld & Chen ([1998](#ref-Ramskold1998)), fig 3.8C.

*Ovatiovermis cribratus*: No evidence of branching ([Caron & Aria 2017](#ref-Caron2017)).

### [79] Metamerically arranged dorsolateral epidermal specializations


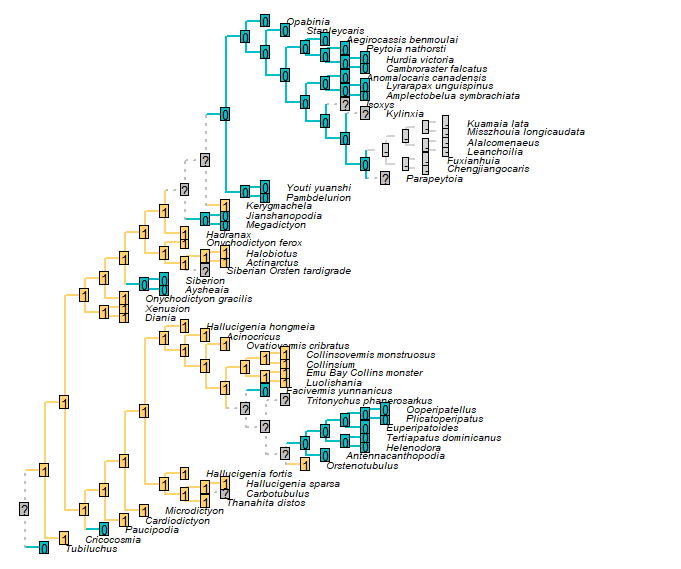


**Supplementary Figure 79: Trunk region: Metamerically arranged dorsolateral epidermal specializations**

0 absent

1 present

This character refers to the differentiated epidermal regions found on the dorsal side of most lobopodians. The epidermal specialization is usually conspicuous, as in the paired nodes of *Xenusion* ([Dzik & Krumbiegel 1989](#ref-Dzik1989)), *Hadranax* ([Budd & Peel 1998](#ref-Budd1998p)) and *Kerygmachela* ([Budd 1993](#ref-Budd1993), [1998*b*](#ref-Budd1998trse)); the sclerotized plates of *Onychodictyon* ([Zhang & Briggs 2007](#ref-Zhang2007); [Ou *et al.* 2012](#ref-Ou2012)); and the spines of *Hallucigenia* ([Ramsköld 1992](#ref-Ramskold1992); [Hou & Bergström 1995](#ref-Hou1995zjls); [Steiner *et al.* 2012](#ref-Steiner2012)), luolishaniids ([Ma *et al.* 2009](#ref-Ma2009); [Yang *et al.* 2015](#ref-Yang2015)) and *Orstenotubulus* ([Maas *et al.* 2007](#ref-Maas2007csb)). The transformation is also coded as present in the modern tardigrades, denoting the paired pit-like structures associated with each pair of legs. These have been described as sites for muscular attachment in the visceral side of the body wall (e.g. [Halberg *et al.* 2009](#ref-Halberg2009); [Marchioro *et al.* 2013](#ref-Marchioro2013)); the epidermal specializations of lobopodians have also been interpreted as muscle attachment sites ([Budd 2001*b*](#ref-Budd2001ed); [Zhang & Briggs 2007](#ref-Zhang2007)).

Character 41 in Smith & Caron ([2015](#ref-Smith2015)) and 39 in Yang *et al.* ([2015](#ref-Yang2015)).

*Aysheaia*, *Paucipodia*: *Paucipodia* ([Chen *et al.* 1995*b*](#ref-Chen1995trse)) and *Aysheaia* ([Liu & Dunlop 2014 fig. 1](#ref-Liu2014ppp)) have been reported to bear subtle sub-circular specializations, but these putative structures in fact represent flattened appendages ([Hou *et al.* 2004](#ref-Hou2004); [Yang *et al.* 2015](#ref-Yang2015)).

*Carbotubulus*: Ambiguous, as the dorsal surface is not visible in the available material ([Haug *et al.* 2012*b*](#ref-Haug2012cb)).

*Cricocosmia*: Present ([Han *et al.* 2007](#ref-Han2007app); [Steiner *et al.* 2012](#ref-Steiner2012)).

*Diania*: Coded as present based on the shield-like specializations associated with each leg pair ([Ma *et al.* 2014*a* fig. 2](#ref-Ma2014jsp)).

*Facivermis yunnanicus*: Not evident ([Howard *et al.* 2020](#ref-Howard2020)).

*Ovatiovermis cribratus*: We interpret the ‘gut diverculata’ described by Caron & Aria ([2017](#ref-Caron2017)) to be dorsal epidermal evaginations. As Caron & Aria ([2017](#ref-Caron2017)) point out, these features are located above limb pairs. Their additional file 1 shows these features are pointed dorsally and rounded ventrally, and exhibit more consistent shape. They also overprint the annulations in their additional file 4 panel c, consistent with being an external feature – and where they do so they exhibit a well-defined gut margin. These features have a paired appearance in Additional file 2 panel b.

Furthermore, elemental mapping in their fig 1C shows no hint of a gut characterization in the posteriormost element (see also panel D, E in their additional file 6). Instead, they are associated with elevated concentrations of carbon, as are the claws; see the carbon distribution in additional file 6 panel A. They extend beyond the body wall (additional figure 9), indicating an external feature.

*Pambdelurion*: Not evident in well-preserved specimens of Budd ([1998*a*](#ref-Budd1998ar)) or Young & Vinther ([2017](#ref-Young2017)).

*Siberion*: Impressions of the dorsal and ventral surfaces are interpreted as evident on the single specimen; neither surface shows evidence of epidermal specializations ([Dzik 2011](#ref-Dzik2011)).

### [80] Concavity


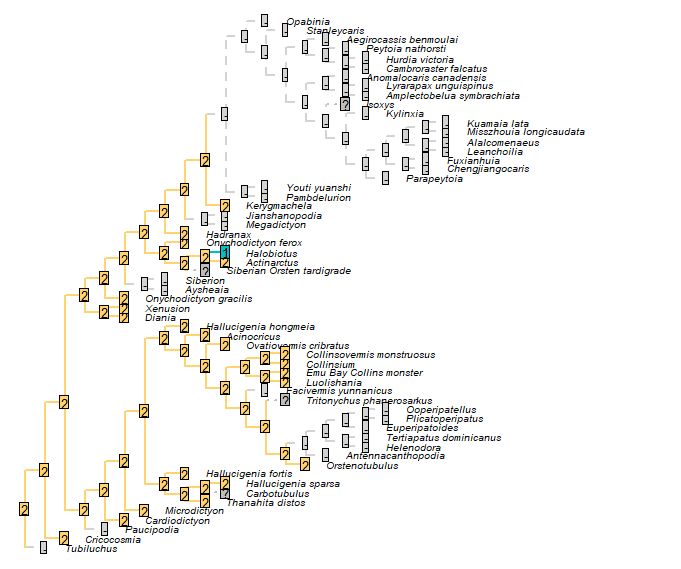


**Supplementary Figure 80: Trunk region: Epidermal specializations: Concavity**

- Inapplicable

1 epidermal depressions

2 epidermal evaginations

The nodes, plates and spines of *Cricocosmia* and lobopodian taxa represent epidermal evaginations; the paired sclerotized dorsal plates of *Actinarctus* (Heterotardigrada) have also been interpreted as epidermal evaginations (e.g. [Nelson 2002](#ref-Nelson2002); [Marchioro *et al.* 2013](#ref-Marchioro2013); [Persson *et al.* 2014](#ref-Persson2014)). *Halobiotus* (Eutardigrada) has epidermal depressions, represented by the paired pits that serve as muscle attachment sites ([Halberg *et al.* 2009](#ref-Halberg2009); [Marchioro *et al.* 2013](#ref-Marchioro2013)). The nodes in *Paucipodia* and *Diania* are scored as epidermal evaginations ([Chen *et al.* 1995*b*](#ref-Chen1995trse); [Liu & Dunlop 2014](#ref-Liu2014ppp); [Ma *et al.* 2014*a*](#ref-Ma2014jsp)).

Character 42 in Smith & Caron ([2015](#ref-Smith2015)) and 40 in Yang *et al.* ([2015](#ref-Yang2015)).

### [81] Evaginations: Proportions


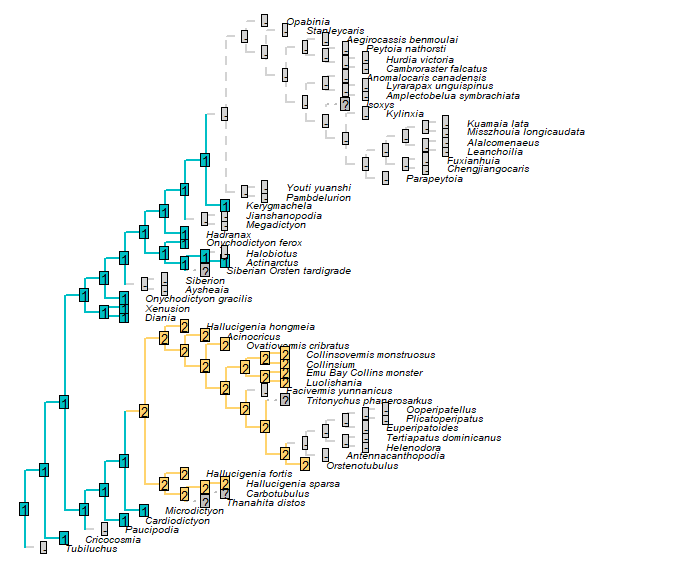


**Supplementary Figure 81: Trunk region: Epidermal specializations: Evaginations: Proportions**

- Inapplicable

1 wider than tall (e.g. nodes or plates)

2 taller than wide (e.g. spines)

Epidermal evaginations fall into two geometric categories: flat nodes or plates and tall spines.

Character 43 in Smith & Caron ([2015](#ref-Smith2015)) and 41 in Yang *et al.* ([2015](#ref-Yang2015)).

*Thanahita distos*: Equant ([Siveter *et al.* 2018](#ref-Siveter2018)), so coded ambiguous.

### [82] Evaginations: Acute distal termination


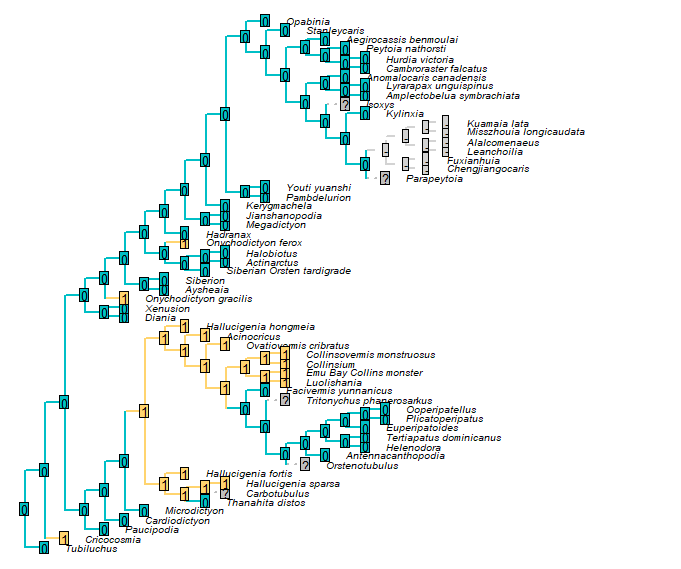


**Supplementary Figure 82: Trunk region: Epidermal specializations: Evaginations: Acute distal termination**

0 absent

1 present

This character refers solely to the shape of the trunk evaginations’ apices. It is independent from the evaginations’ proportions, as demonstrated by *Onychodictyon* *ferox*, where sclerites are wider than tall (i.e. plates) but display an acute distal termination ([Zhang & Briggs 2007](#ref-Zhang2007); [Ou *et al.* 2012](#ref-Ou2012); [Topper *et al.* 2013](#ref-Topper2013)).

Character 44 in Smith & Caron ([2015](#ref-Smith2015)) and 42 in Yang *et al.* ([2015](#ref-Yang2015)).

*Onychodictyon gracilis*: Apical tip in sclerotized evaginations of *O. gracilis* evident in Liu *et al.* ([2008*b*](#ref-Liu2008app)) fig. 3, panels A1-A3, B1.

*Orstenotubulus*: Ambiguously preserved ([Maas *et al.* 2007](#ref-Maas2007csb)).

### [83] Evaginations: Acute distal termination: Curvature


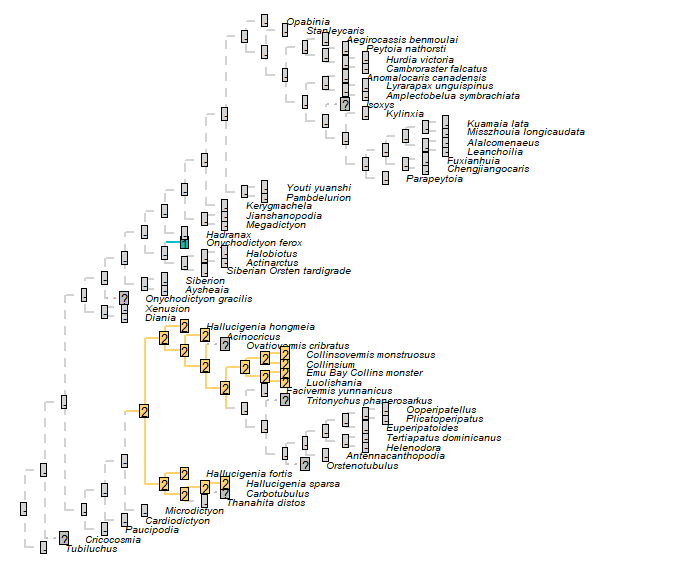


**Supplementary Figure 83: Trunk region: Epidermal specializations: Evaginations: Acute distal termination: Curvature**

- Inapplicable

1 straight

2 curved

The spines of *Hallucigenia* ([Hou & Bergström 1995](#ref-Hou1995zjls); [Steiner *et al.* 2012](#ref-Steiner2012); [Smith & Caron 2015](#ref-Smith2015)), *Luolishania* ([Ma *et al.* 2009](#ref-Ma2009)) and the Emu Bay “Collins Monster” ([García-Bellido *et al.* 2013](#ref-Garcia2013)) are distinctively curved, whereas those *Onychodictyon* *ferox* ([Topper *et al.* 2013](#ref-Topper2013)) are essentially straight.

Character 45 in Smith & Caron ([2015](#ref-Smith2015)) and 43 in Yang *et al.* ([2015](#ref-Yang2015)).

*Cricocosmia*: The distal termination of *Cricocosmia*’s epidermal evaginations appear blunted [Han *et al.* ([2007](#ref-Han2007app)), fig 2C; contra the reconstruction given in fig 6A1] compared to the pointed tips of e.g. *Onychodictyon* or *Hallucigenia*, therefore we code the shape of the distal termination to be ambiguous.

*Hallucigenia sparsa*: The spines of *Hallucigenia* *sparsa* are gently curved ([Smith & Ortega-Hernández 2014](#ref-Smith2014); [Smith & Caron 2015](#ref-Smith2015)).

*Onychodictyon ferox*: Figs 1B.1, 2C in Liu *et al.* ([2008*b*](#ref-Liu2008app)) show *O. ferox* with a straight distal termination in epidermal evagination.

*Onychodictyon gracilis*: Liu *et al.* ([2008*b*](#ref-Liu2008app)) figures 3a, b depict a curved morphology; however, as fossil photographs do not convincingly demonstrate this interpretation, we code as ambiguous.

### [84] Evaginations: Sclerotization


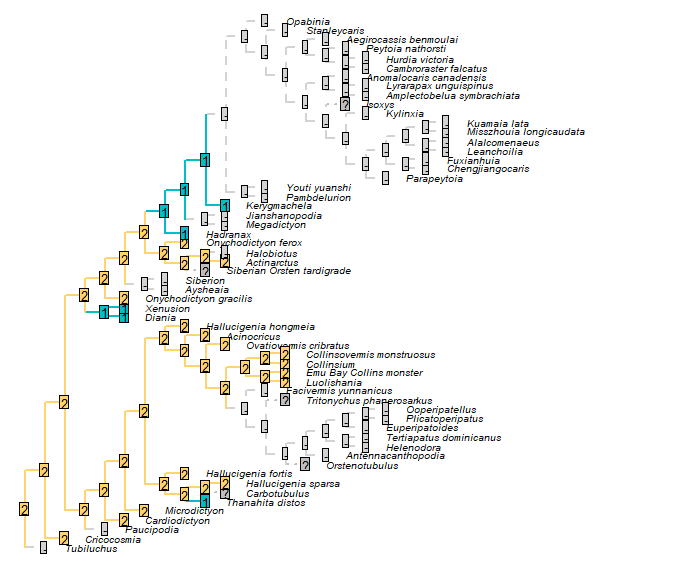


**Supplementary Figure 84: Trunk region: Epidermal specializations: Evaginations: Sclerotization**

- Inapplicable

1 epidermal evaginations not sclerotized

2 epidermal evaginations sclerotized

The epidermal evaginations of *Cricocosmia* and “armoured” lobopodians are substantially sclerotized ([Hou & Bergström 1995](#ref-Hou1995zjls); [Han *et al.* 2007](#ref-Han2007app); [Steiner *et al.* 2012](#ref-Steiner2012); [Caron *et al.* 2013](#ref-Caron2013)), in contrast to those of *Xenusion* ([Dzik & Krumbiegel 1989](#ref-Dzik1989)), *Hadranax* ([Budd & Peel 1998](#ref-Budd1998p)), *Diania* ([Ma *et al.* 2014*a*](#ref-Ma2014jsp)) and *Kerygmachela* Budd ([1998*b*](#ref-Budd1998trse)).

Character 46 in Smith & Caron ([2015](#ref-Smith2015)) and 44 in Yang *et al.* ([2015](#ref-Yang2015)).

*Thanahita distos*: Not obviously sclerotized ([Siveter *et al.* 2018](#ref-Siveter2018)).

### [85] Evaginations: Ornament


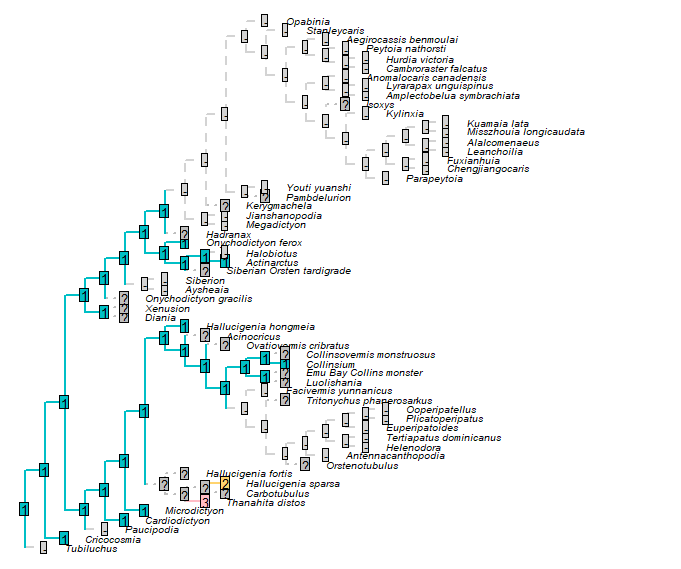


**Supplementary Figure 85: Trunk region: Epidermal specializations: Evaginations: Ornament**

- Inapplicable

1 net-like

2 scaly

3 tufted

We code this character as uncertain in taxa that are not well enough preserved for the ornament to be apparent. *Hallucigenia* *sparsa* has a scaly ornament ([Caron *et al.* 2013](#ref-Caron2013)) whereas *H. hongmeia* and *Collinsium* bear a net-like pattern ([Steiner *et al.* 2012](#ref-Steiner2012); [Yang *et al.* 2015](#ref-Yang2015)) shared with *Onychodictyon*, *Microdictyon* and *Cricocosmia* ([Han *et al.* 2007](#ref-Han2007app); [Topper *et al.* 2013](#ref-Topper2013)); *Cardiodictyon* specimens show a comparable ornament ([Liu & Dunlop 2014 fig. 4f](#ref-Liu2014ppp)).

Character 47 in Smith & Caron ([2015](#ref-Smith2015)) and 45 in Yang *et al.* ([2015](#ref-Yang2015)).

*Actinarctus*: *Actinarctus* sclerites exhibit a net-like ornament ([Marchioro *et al.* 2013](#ref-Marchioro2013)).

*Collinsium*: “The dorsolateral spines of *Collinsium* have a distinctive punctate-like ornamentation similar to that of *H. hongmeia*.” ([Yang *et al.* 2015](#ref-Yang2015)).

*Hallucigenia fortis*: Unclear from Hou & Bergström ([1995](#ref-Hou1995zjls)), but clearly not inapplicable.

*Onychodictyon ferox*: Liu *et al.* ([2008*b*](#ref-Liu2008app)), fig. 2B, shows a net like texture of sclerite ornaments for *O. ferox*, similar to those described by Topper *et al.* ([2013](#ref-Topper2013)) in *Onychodictyon* sp. plates.

*Onychodictyon gracilis*: Coded ambiguous as the texture is difficult to discern from the figures of Liu *et al.* ([2008*b*](#ref-Liu2008app)).

*Thanahita distos*: *Thanahita* exhibits a distinct tuft-like morphology ([Siveter *et al.* 2018](#ref-Siveter2018)).

### [86] Sclerites consist of a stack of constituent elements


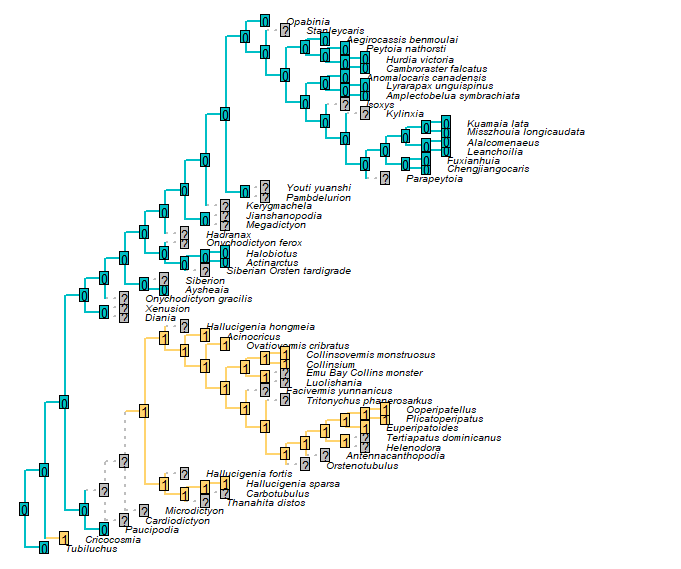


**Supplementary Figure 86: Trunk region: Sclerites consist of a stack of constituent elements**

0 sclerites comprise single element

1 sclerites comprise stacked elements

This neomorphic character is coded as present in any taxon where exoskeletal elements (claws or epidermal evaginations) comprise stacked constituent elements at all stages of growth (as in *Hallucigenia* *sparsa*, *Euperipatoides* and *Collinsium*, see [Smith & Ortega-Hernández 2014](#ref-Smith2014); [Yang *et al.* 2015](#ref-Yang2015)), not just during ecdysis (as in *Onychodictyon*, see [Topper *et al.* 2013](#ref-Topper2013)). Whereas the dorsal sclerites of *Microdictyon* and *Onychodictyon* do not express constituent elements, the detailed construction of their claws is not known; as such these taxa are coded as ambiguous. Where sclerites are not preserved in sufficient detail to assess their construction, this character is coded as ambiguous.

Character 48 in Smith & Caron ([2015](#ref-Smith2015)) and 46 in Yang *et al.* ([2015](#ref-Yang2015)).

*Acinocricus*: Stacked elements present in spines ([Caron & Aria 2020](#ref-Caron2020)).

*Aysheaia*: *Aysheaia* claws do not have stacked elements ([Smith & Ortega-Hernández 2014](#ref-Smith2014)).

*Collinsovermis monstruosus*: Present; see discussion in Caron & Aria ([2020](#ref-Caron2020)).

*Cricocosmia*: Stacked elements present in claws ([Dhungana 2024](#ref-Dhungana2024)).

*Facivermis yunnanicus*: Spines not described in adequate detail to evaluate presence of stacked elements ([Howard *et al.* 2020](#ref-Howard2020)).

*Ovatiovermis cribratus*: Claws comprise stacked elements ([Caron & Aria 2017](#ref-Caron2017)).

*Paucipodia*: No stacked element construction evident in claws ([Vannier & Martin 2017](#ref-Vannier2017)).

### [87] Maximum number above each leg pair


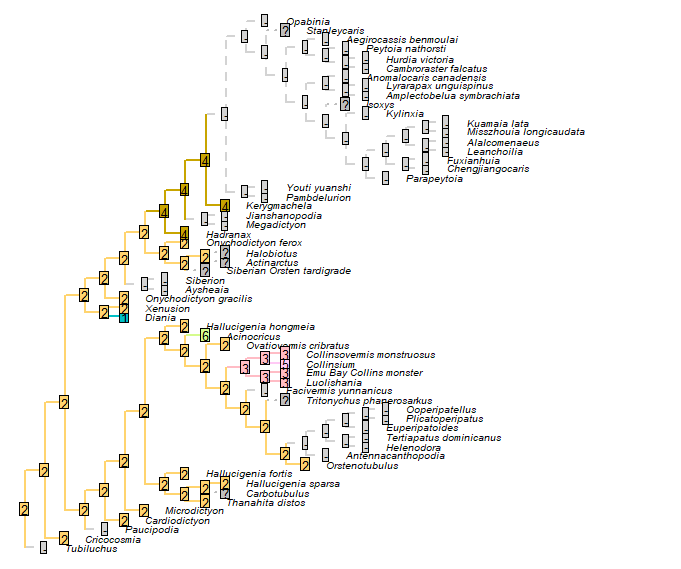


**Supplementary Figure 87: Trunk region: Epidermal specializations: Maximum number above each leg pair**

- Inapplicable

1 one

2 two

3 three

4 four

5 five

6 seven

We score *Cardiodictyon* as having two epidermal specializations, following suggestions that the apparently single dorsal sclerite is formed by the fusion of a pair of elements ([Liu & Dunlop 2014](#ref-Liu2014ppp)). The plates of *Cricocosmia* occur in pairs ([Han *et al.* 2007](#ref-Han2007app)). *Collinsium* bears five primary spines ([Yang *et al.* 2015](#ref-Yang2015)); *Acinocricus* bears seven ([Conway Morris & Robison 1988](#ref-ConwayMorris1988)). Tardigrades are coded as ambiguous in view of the complex integration of their dorsal plates.

Character 49 in Smith & Caron ([2015](#ref-Smith2015)) and 47 in Yang *et al.* ([2015](#ref-Yang2015)).

*Collinsovermis monstruosus*: Three ([Caron & Aria 2020](#ref-Caron2020)).

*Halobiotus*: *Halobiotus* (Eutardigrada) has paired epidermal specialisations (depressions), represented by pits that serve as muscle attachment sites ([Halberg *et al.* 2009](#ref-Halberg2009); [Marchioro *et al.* 2013](#ref-Marchioro2013)).

*Thanahita distos*: Two papillae reported per leg pair, with additional in between leg pairs ([Siveter *et al.* 2018](#ref-Siveter2018)).

### [88] “Secondary” structures


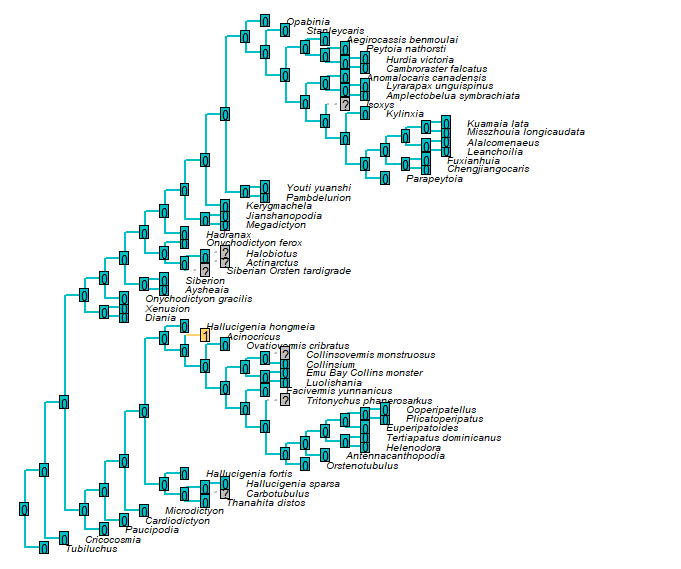


**Supplementary Figure 88: Trunk region: Epidermal specializations: “Secondary” structures**

0 absent

1 present

Uniquely, *Acinocricus* bears diminutive spines between the “primary” spines that decrease gradually in length along the length of the body ([Conway Morris & Robison 1988](#ref-ConwayMorris1988)).

Character 48 in Yang *et al.* ([2015](#ref-Yang2015)).

### [89] Consistent size


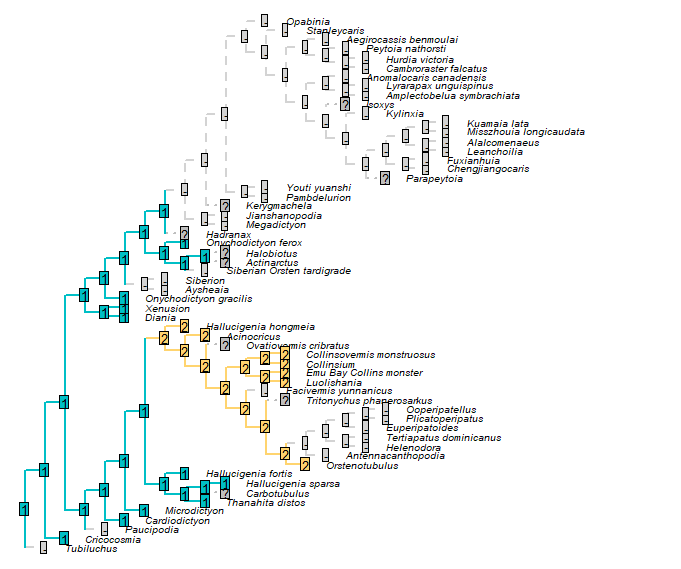


**Supplementary Figure 89: Trunk region: Epidermal specializations: Consistent size**

- Inapplicable

1 each group of dorsal elements of equivalent size

2 size of dorsal elements varies between groups

In most armoured lobopodians, each group of dorsal spines or plates exhibits a similar size (e.g. [Smith & Caron 2015](#ref-Smith2015)). In *Collinsium*, *Hallucigenia* *hongmeia*, *Luolishania*, *Acinocricus* and the Emu Bay Collins Monster, the size of spines varies between each group ([Conway Morris & Robison 1988](#ref-ConwayMorris1988); [Liu *et al.* 2007](#ref-Liu2007az); [Ma *et al.* 2009](#ref-Ma2009); [Steiner *et al.* 2012](#ref-Steiner2012); [García-Bellido *et al.* 2013](#ref-Garcia2013)).

Character 49 in Yang *et al.* ([2015](#ref-Yang2015)).

*Hadranax*: Not all dorsal specialisations present, as trunk incomplete in Budd & Peel ([1998](#ref-Budd1998p)), therefore coded as ambiguous (applicable).

*Orstenotubulus*: *Orstenotubulus* has prominent spines and buttresses above some leg pairs, but these are profoundly diminished above others ([Maas *et al.* 2007](#ref-Maas2007csb)).

### [90] Spacing


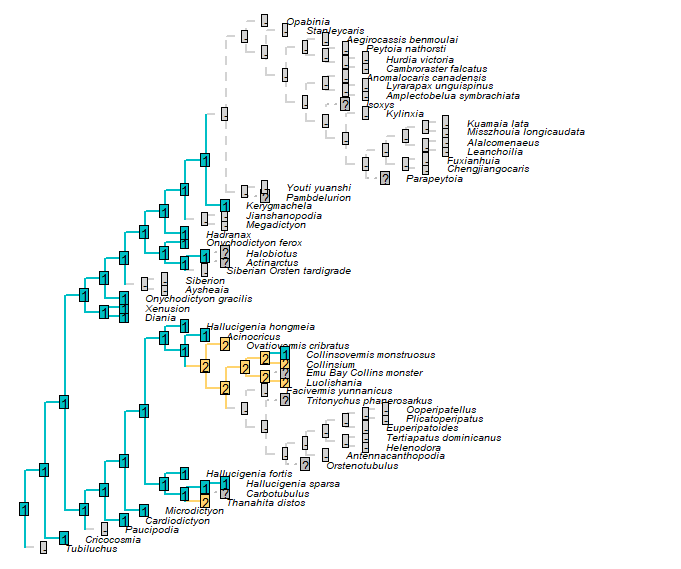


**Supplementary Figure 90: Trunk region: Epidermal specializations: Spacing**

- Inapplicable

1 regular

2 variable

In most lobopodian taxa, the epidermal specializations exhibit a regular spacing, even if the spacing of appendages varies along the body ([Smith & Caron 2015](#ref-Smith2015)). Both *Collinsium* and *Luolishania*, by contrast, exhibit an extended spacing between spines in the medial portion of the trunk ([Ma *et al.* 2009](#ref-Ma2009); [Yang *et al.* 2015](#ref-Yang2015)).

Character 50 in Yang *et al.* ([2015](#ref-Yang2015)).

### [91] Spines on trunk


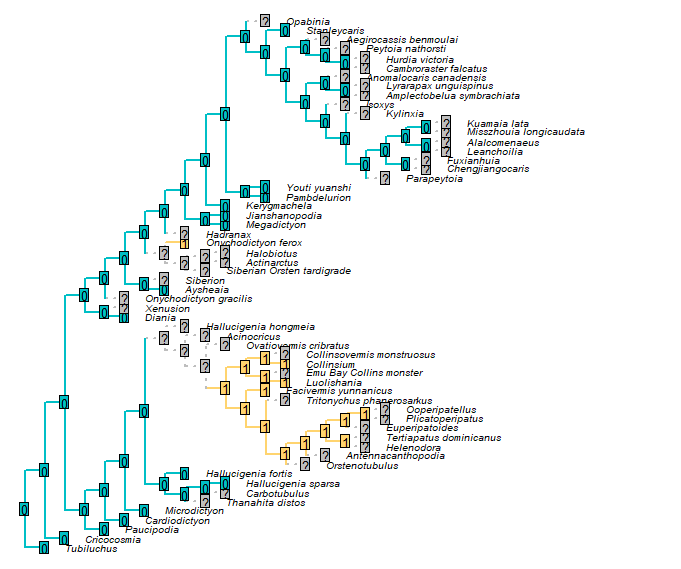


**Supplementary Figure 91: Trunk region: Spines on trunk**

0 absent

1 present

Some lobopodians such as *Onychodictyon* *ferox* have spines on the trunk in addition to spines on appendages. Others, such as *Aysheaia*, have spines on some appendages but lack spines on the trunk.

Treated as Neomorphic.

### [92] Papillae on trunk annulations


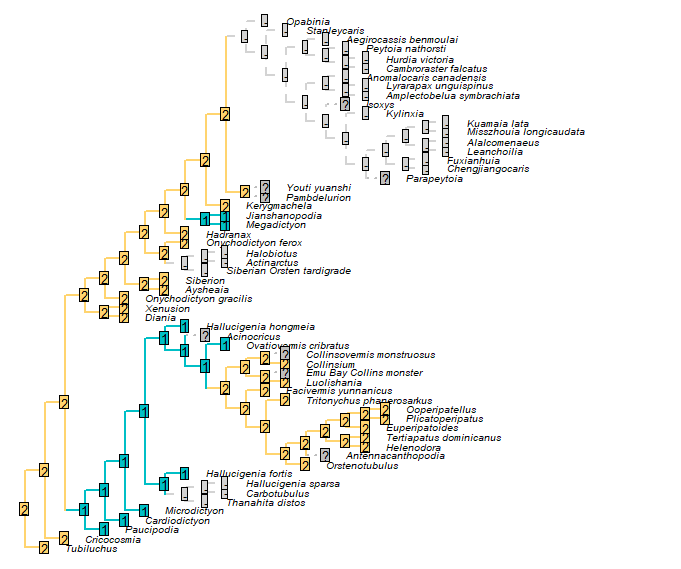


**Supplementary Figure 92: Trunk region: Papillae on trunk annulations**

- Inapplicable

1 absent

2 present

Treated as transformational as it is not clear whether the absence of papillae on limbs represents a differentiation of the limbs (and the introduction of a separate developmental regime to pattern them independently from the trunk)

Character 41 in Ma *et al.* ([2014*a*](#ref-Ma2014jsp)); character 50 in Smith & Caron ([2015](#ref-Smith2015)) and 51 in Yang *et al.* ([2015](#ref-Yang2015)).

*Antennacanthopodia*: Coded as ambiguous in *Antennacanthopodia* ([Ou *et al.* 2011](#ref-Ou2011)) as its trunk annulations are not clearly apparent.

*Facivermis yunnanicus*: Present ([Howard *et al.* 2020](#ref-Howard2020)).

*Orstenotubulus*: Present ([Maas *et al.* 2007](#ref-Maas2007csb)).

### [93] Serially repeated mid-gut glands


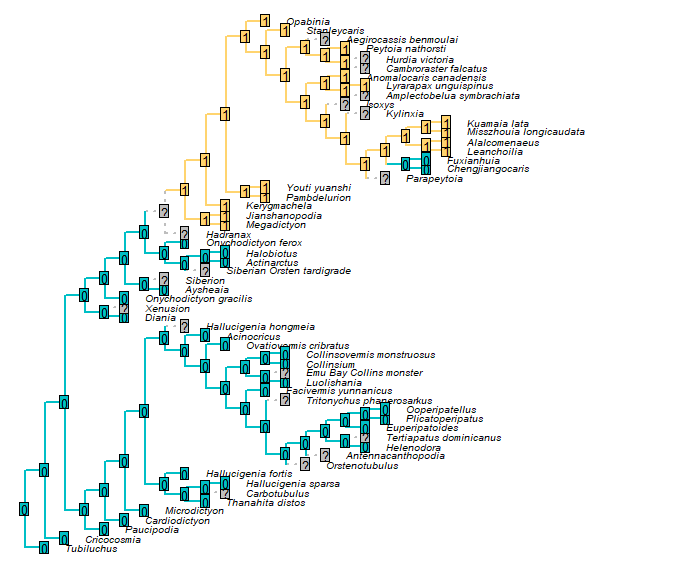


**Supplementary Figure 93: Trunk region: Serially repeated mid-gut glands**

0 absent

1 reniform, submillimetric lamellar

The nature of the mid-gut glands of *Megadictyon*, *Jianshanopodia*, *Pambdelurion* and *Opabinia* is elucidated by ([Vannier *et al.* 2014](#ref-Vannier2014)). Midgut glands were biologically, rather than taphonomically, absent in *Ilyodes* ([Haug *et al.* 2012*b*](#ref-Haug2012cb)), *Hallucigenia* *sparsa* ([Smith & Caron 2015](#ref-Smith2015)), *Lyrarapax* ([Cong *et al.* 2014](#ref-Cong2014)), *Acinocricus* ([Conway Morris & Robison 1988](#ref-ConwayMorris1988)) and *Collinsium* ([Yang *et al.* 2015](#ref-Yang2015)).

Character 42 in Ma *et al.* ([2014*a*](#ref-Ma2014jsp)); 16 in Daley *et al.* ([2009](#ref-Daley2009)); 53 in Smith & Caron ([2015](#ref-Smith2015)) and 52 in Yang *et al.* ([2015](#ref-Yang2015)).

*Anomalocaris canadensis*: Present ([Briggs & Robison 1984](#ref-Briggs1984); [Daley & Edgecombe 2014](#ref-Daley2014)).

*Antennacanthopodia*: Coded as uncertain in *Antennacanthopodia* ([Ou *et al.* 2011](#ref-Ou2011)) because the dark infilling of the type material may represent decayed internal organs.

*Facivermis yunnanicus*: Absent ([Howard *et al.* 2020](#ref-Howard2020)).

*Lyrarapax unguispinus*: Present in *L. trilobus* ([Cong *et al.* 2016](#ref-Cong2016)); reported absence in *L. unguispinus* ([Cong *et al.* 2014](#ref-Cong2014)) attributed to non-preservation.

*Youti yuanshi*: Present (this study).

### [94] Differentiated anterior trunk


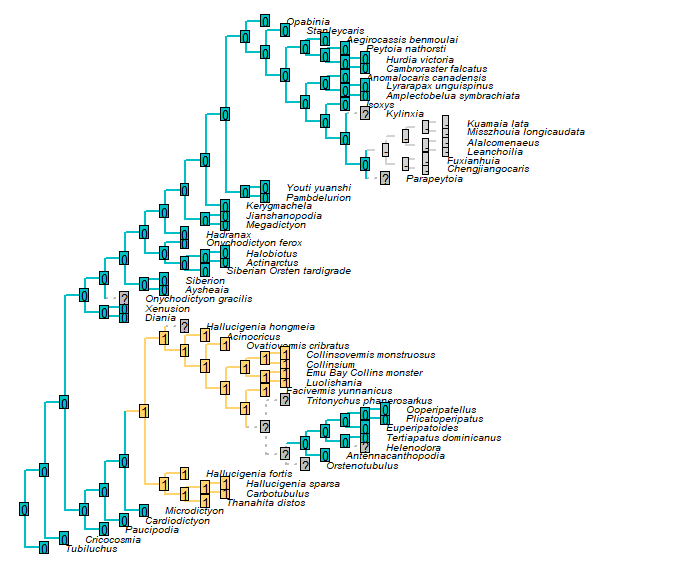


**Supplementary Figure 94: Trunk region: Differentiated anterior trunk**

0 trunk of uniform construction

1 anterior trunk differentiated from posterior trunk by abrupt change in thickness, armature and appendage construction

This character reflects the pronounced differentiation of the posterior and anterior trunk – not just the trunk appendages – in certain lobopodians. In *Hallucigenia* *sparsa*, the region of the trunk anterior of the third appendage pair is narrower, lacks dorsal armature, and expresses differentiated appendages ([Smith & Caron 2015](#ref-Smith2015)). The short constricted region anterior of the first spine pair in *H. fortis* is associated with two differentiated appendage pairs ([Ramsköld & Chen 1998](#ref-Ramskold1998)) and apparently corresponds with the ‘neck’ of *H. sparsa*. In luolishaniids, the anterior body bears elongate limbs with accentuated armature ([Ma *et al.* 2009](#ref-Ma2009); [García-Bellido *et al.* 2013](#ref-Garcia2013)). The portion of the trunk in *Carbotubulus* corresponding to the first two or three leg pairs is substantially narrower than the posterior trunk and its associated appendages are narrower and less prominent than the posterior appendages, indicating trunk differentiation ([Haug *et al.* 2012*b*](#ref-Haug2012cb)). Although the width of the trunk narrows gradually towards the front of *Paucipodia*, this tapering is gradual and does not correspond to the differentiation of the anterior trunk ([Chen *et al.* 1995*b*](#ref-Chen1995trse); [Hou *et al.* 2004](#ref-Hou2004)). Coded ambiguous in *Orstenotubulus*, *Hallucigenia* *hongmeia*, and *Ilyodes* due to incomplete preservation ([Thompson & Jones 1980](#ref-Thompson1980); [Maas *et al.* 2007](#ref-Maas2007csb); [Steiner *et al.* 2012](#ref-Steiner2012)).

Character 54 in Smith & Caron ([2015](#ref-Smith2015)) and 72 in Yang *et al.* ([2015](#ref-Yang2015)).

*Facivermis yunnanicus*: Differentiated: posterior trunk lacks appendages ([Howard *et al.* 2020](#ref-Howard2020)).

*Ovatiovermis cribratus*: Short posterior trunk comprising three appendage pairs ([Caron & Aria 2017](#ref-Caron2017)).

*Thanahita distos*: Change in appendage construction, and possibly thickness ([Siveter *et al.* 2018](#ref-Siveter2018)).

### [95] Narrowing posteriad


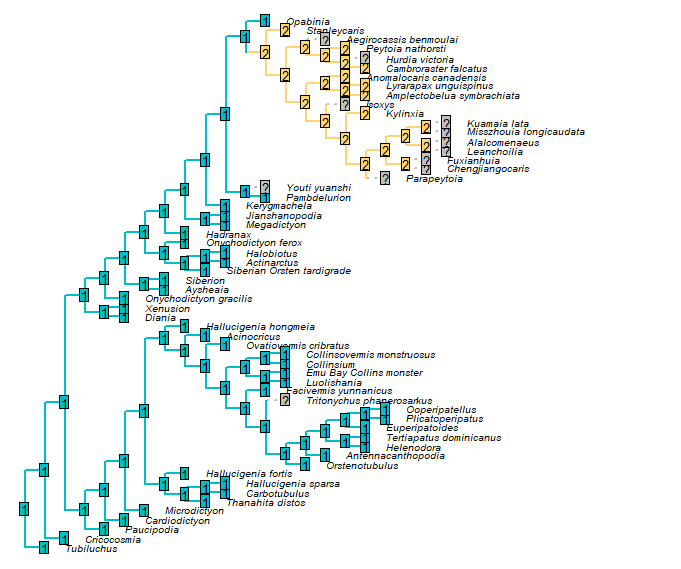


**Supplementary Figure 95: Trunk region: Narrowing posteriad**

- Inapplicable

1 broadly uniform trunk width

2 substantial posteriad trend to narrower trunk

Lobopodians have a relatively cylindrical trunk with a uniform width, whereas the trunk of anomalocaridids narrows markedly towards the posterior.

Character 65 in Moysiuk & Caron ([2019](#ref-Moysiuk2019)).

*Kerygmachela*, *Pambdelurion*, *Opabinia*, *Amplectobelua symbrachiata*, *Anomalocaris canadensis*, *Cambroraster falcatus*, *Hurdia victoria*, *Peytoia nathorsti*, *Aegirocassis benmoulai*, *Lyrarapax unguispinus*: Following Moysiuk & Caron ([2019](#ref-Moysiuk2019)).

*Kylinxia*: Zeng *et al.* ([2020](#ref-Zeng2020)) supplementary info clarifies narrowing trend posteriad.

*Youti yuanshi*: Unknown whether narrowing (this study) is developmental or would be retained to adulthood.

## Trunk appendages

### [96] Sclerotization


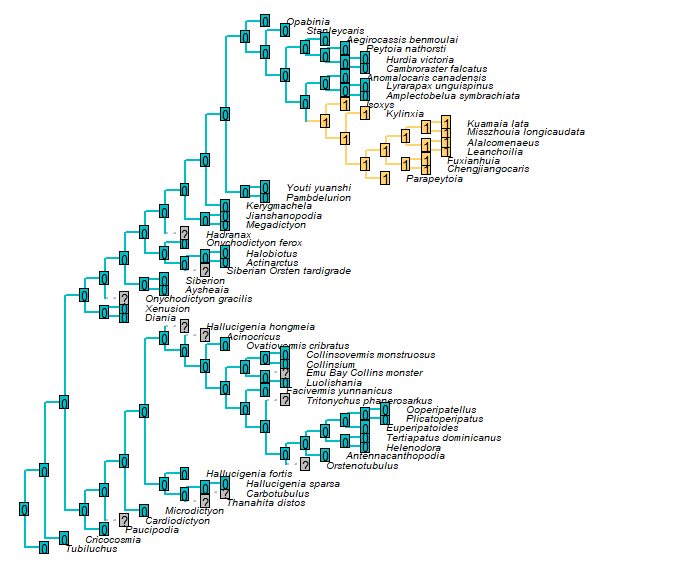


**Supplementary Figure 96: Trunk appendages: Sclerotization**

0 not sclerotized

1 sclerotized

Arthropodization is thought to happen first in pre-ocular appendages, then co-opted to the rest of the appendages ([Chipman & Edgecombe 2019](#ref-Chipman2019)). This character reflects this hypothesised event. Sclerotization is thought to occur simultaneously in all trunk appendages (as they have been co-opted from the pre-ocular appendages), therefore we code this in one character. This is treated as a neomorphic character as the trunk appendages’ co-option of sclerotization from pre-ocular appendages requires additional genetic control.

### [97] Trunk exites


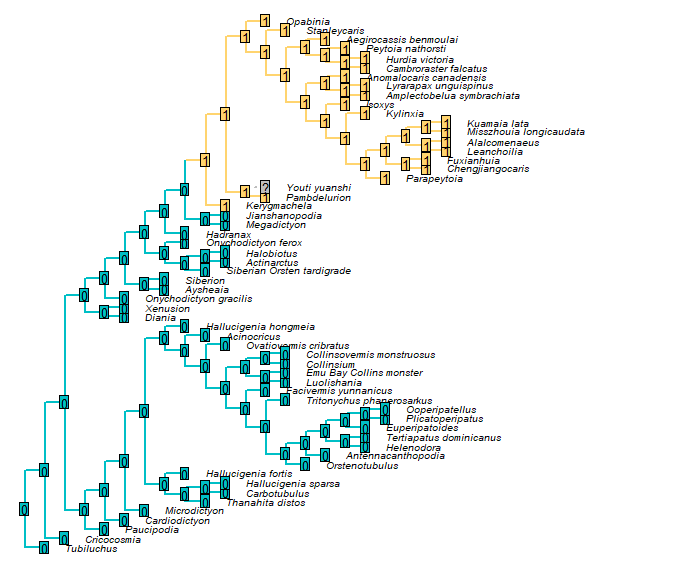


**Supplementary Figure 97: Trunk appendages: Trunk exites**

0 absent

1 present

To summarise Daley *et al.* ([2009](#ref-Daley2009)) and Van Roy *et al.* ([2015](#ref-VanRoy2015)): Exopods, the outer branch of a true biramous limb, are unique to Mandibulata. The outer appendage branch of chelicerates and many stem-group euarthropods is interpreted as an exite, a lateral flap which is not homologous to the mandibulate exopod (see also [Bruce & Patel 2020](#ref-Bruce2020)). Daley *et al.* ([2009](#ref-Daley2009)) treated this flap as homologous with the lateral flaps of anomalocaridids and gilled lobopodians, which often bear dorsal lanceolate blades (= setal blades). Van Roy *et al.* ([2015](#ref-VanRoy2015)) considered the setal blades themselves to represent the exite, homologizing the wrinkling on *Kerygmachela* and *Pambdelurion* flaps, the setal blades of *Opabinia* and anomalocaridids, and the exites of upper stem euarthropods. Dorsal flaps are therefore not necessarily present in addition to the setal blades; indeed Van Roy *et al.* ([2015](#ref-VanRoy2015)) code them as absent in Amplectobeluids and *Anomalocaris* (as well as euarthropods).

Adapted from character 31 in Daley *et al.* ([2009](#ref-Daley2009)). Character 55 in Smith & Caron ([2015](#ref-Smith2015)) and 53 in Yang *et al.* ([2015](#ref-Yang2015)). Treated as neomorphic.

*Youti yuanshi*: Coded ambiguous: not present at larval stage (this study), but dorsal extensions of the haemolymph system are plausible precursors of a feature that may be added in an adult stage with different metabolic requirements.

### [98] Form of exite


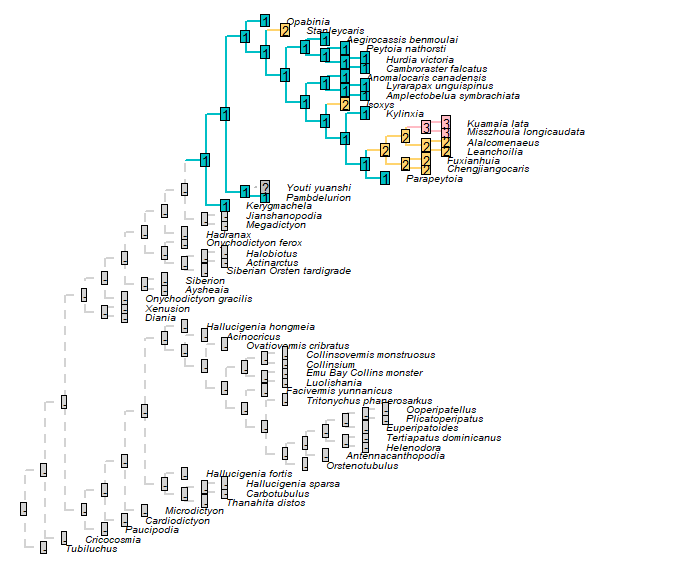


**Supplementary Figure 98: Trunk appendages: Form of exite**

- Inapplicable

1 lateral lobes

2 setal blades

3 simple oval paddle with marginal spines

4 bipartite shaft with lamellar setae

The description of *Aegirocassis* Van Roy *et al.* ([2015](#ref-VanRoy2015)) clarifies the relationship of the dorsal lanceolate (setal) blades in gilled lobopodians and radiodontans, and establishes their homology with setae borne on the outer appendage branches (i.e. exites) of upper-stem Euarthropoda.

Adapted from characters 51, 56 and 68 from Smith & Caron ([2015](#ref-Smith2015)) and 54 in Yang *et al.* ([2015](#ref-Yang2015)).

### [99] Exites fused with endopod to form biramous appendage


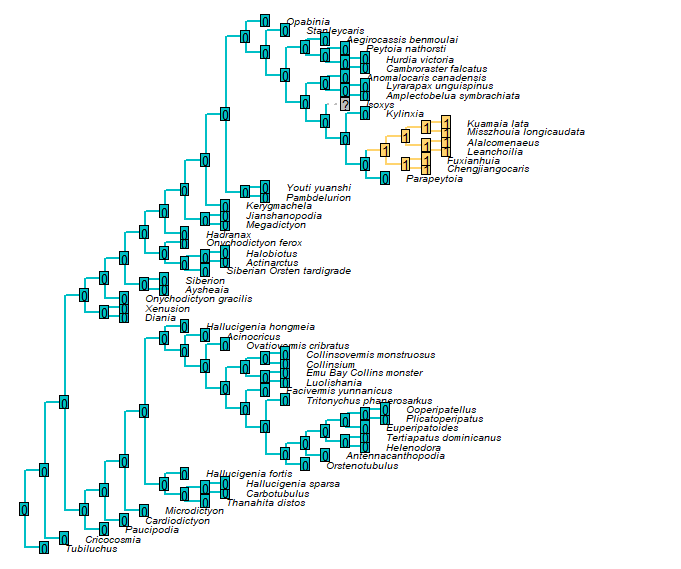


**Supplementary Figure 99: Trunk appendages: Exites fused with endopod to form biramous appendage**

0 not fused

1 fused

The dorsal flaps of anomalocaridids and gilled lobopodians are considered as homologous with euarthropod exites. See character 57 in Van Roy *et al.* ([2015](#ref-VanRoy2015)).

Character 57 in Smith & Caron ([2015](#ref-Smith2015)) and Yang *et al.* ([2015](#ref-Yang2015)). Treated as neomorphic.

### [100] Dorsal flaps


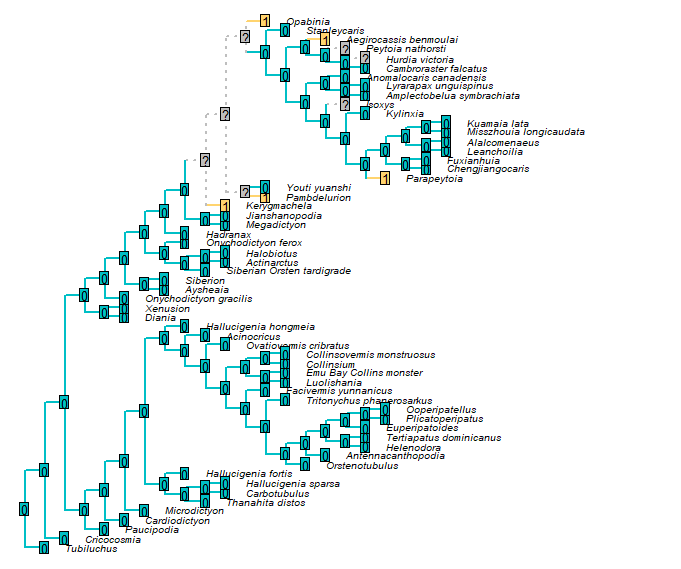


**Supplementary Figure 100: Trunk appendages: Dorsal flaps**

0 absent

1 present

Van Roy *et al.* ([2015](#ref-VanRoy2015)) consider the setal blades to represent exites. Dorsal flaps are not always present in addition to the setal blades: the (ventral) flaps of amplectobeluids and *Anomalocaris* correspond to the euarthropod endopod. Dorsal flaps are considered to represent an elaboration of the setal blades, and thus treated as a neomorphic character.

Modified from character 21 in Van Roy *et al.* ([2015](#ref-VanRoy2015)). Character 67 in Smith & Caron ([2015](#ref-Smith2015)) and 55 in Yang *et al.* ([2015](#ref-Yang2015)).

*Amplectobelua symbrachiata*: Coded as absent by Van Roy *et al.* ([2015](#ref-VanRoy2015)).

*Cambroraster falcatus*: Only a single series of lateral flaps is reconstructed ([Moysiuk & Caron 2019](#ref-Moysiuk2019)).

*Hurdia victoria*: Coded ambiguous: the presence of dorsal and ventral flaps is tentatively interpreted by Van Roy *et al.* ([2015](#ref-VanRoy2015)), though Moysiuk & Caron ([2019](#ref-Moysiuk2019)) consider them absent.

*Kerygmachela*, *Pambdelurion*: Setal blades are expressed as wrinkles on the dorsal flaps of gilled lobopodians ([Van Roy *et al.* 2015](#ref-VanRoy2015)).

*Peytoia nathorsti*: Van Roy *et al.* ([2015](#ref-VanRoy2015)) identify “clear evidence” of two sets of flaps, though Moysiuk & Caron ([2019](#ref-Moysiuk2019)) consider the evidence equivocal. We thus take the *conservative* position of coding this taxon ambiguous.

*Youti yuanshi*: Coded as absent, reflecting the absence of any indication of flaps, despite expression of appendages (this study) – though it remains possible that these structures were not expressed until a later instar.

### [101] Exite distribution

**Supplementary Figure 101: Trunk appendages: Exite distribution**

- Inapplicable

1 confined laterally

2 present dorsally

Character 51 of Van Roy *et al.* ([2015](#ref-VanRoy2015)), reflecting the continuation of setal blades in certain dinocaridids across the dorsal surface.

Character 56 in Yang *et al.* ([2015](#ref-Yang2015)).

*Cambroraster falcatus*: Crossing the body ([Moysiuk & Caron 2019](#ref-Moysiuk2019)).

### [102] Antero-posteriorly compressed protopodite with gnathobasic endites in post-deutocerebral appendage pair

**Supplementary Figure 102: Trunk appendages: Antero-posteriorly compressed protopodite with gnathobasic endites in post-deutocerebral appendage pair**

0 absent

1 present

Gnathobasic appendages are absent in fuxianhuiids ([Chen *et al.* 1995*c*](#ref-Chen1995s); [Waloszek *et al.* 2005](#ref-Waloszek2005); [Bergström *et al.* 2008](#ref-Bergstrom2008); [Yang *et al.* 2013](#ref-Yang2013)) but present in Artiopoda ([Edgecombe & Ramsköld 1999](#ref-Edgecombe1999); [Ortega-Hernández *et al.* 2013](#ref-Ortega2013)) and megacheirans ([Chen *et al.* 2004](#ref-Chen2004); [Haug *et al.* 2012*a*](#ref-Haug2012bmceb), [*c*](#ref-Haug2012p)).

Character 8 of Ma *et al.* ([2014*a*](#ref-Ma2014jsp)); 35 in Daley *et al.* ([2009](#ref-Daley2009)); 58 in Smith & Caron ([2015](#ref-Smith2015)) and Yang *et al.* ([2015](#ref-Yang2015)).

*Amplectobelua symbrachiata*: Coded as present to reflect proposed homology of gnathobasic endites with those of euarthropods ([Cong *et al.* 2017](#ref-Cong2017)).

### [103] Shape of lobopodous appendages

**Supplementary Figure 103: Trunk appendages: Shape of lobopodous appendages**

- Inapplicable

1 cylindrical (e.g. *Hallucigenia* *sparsa*)

2 conical; significantly tapered (e.g. *Aysheaia*)

Some lobopodians have cylindrical appendages (e.g. *Microdictyon*, *Hallucigenia*) whereas others have more conical or tapered lobopods.
Inapplicable when lobopodous limbs are absent.

*Collinsium*: Ambiguous ([Yang *et al.* 2015](#ref-Yang2015)).

*Megadictyon*: Preservation inadequate to distinguish ([Liu *et al.* 2007](#ref-Liu2007az)).

*Onychodictyon ferox*: Neither entirely slender and cylindrical or conical.

*Paucipodia*: Figure 5a in Vannier & Martin ([2017](#ref-Vannier2017)) establishes that lobopods, when oriented parallel to bedding, are cylindrical.

*Siberion*: Appendages not completely preserved ([Dzik 2011](#ref-Dzik2011)), so coded ambiguous.

### [104] Secondary structures on non-sclerotized (lobopodous) limbs

**Supplementary Figure 104: Trunk appendages: Secondary structures on non-sclerotized (lobopodous) limbs**

0 absent

1 present

Ambiguous in euarthropods as sclerotization of limbs is considered to overprint and obscure any secondary structures that may have been present.

Modified from character 9 in Ma *et al.* ([2014*a*](#ref-Ma2014jsp)). 59 in Smith & Caron ([2015](#ref-Smith2015)) and Yang2015.

*Anomalocaris canadensis*: *Anomalocaris* is treated as uncertain ([Van Roy *et al.* 2015](#ref-VanRoy2015)).

*Facivermis yunnanicus*: Double series of *Luolishania*-like spines ([Howard *et al.* 2020](#ref-Howard2020)).

*Hurdia victoria*, *Peytoia nathorsti*: Absent ([Van Roy *et al.* 2015](#ref-VanRoy2015)).

*Onychodictyon gracilis*: We code as *O. gracilis* as uncertain as its longitudinal series of dot-like structures ([Liu *et al.* 2008*a* fig. 2A6](#ref-Liu2008csb)) could indicate an organization of appendicules similar to those of *O. ferox* (see [Ou *et al.* 2012 fig. 2a](#ref-Ou2012)).

*Siberion*: Coded as uncertain because its limbs are poorly preserved ([Dzik 2011](#ref-Dzik2011)).

### [105] Nature of secondary structure

**Supplementary Figure 105: Trunk appendages: Nature of secondary structure**

- Inapplicable

1 spines/setae

2 appendicules

Spines and setae taper to sharp point, whereas appendicules have a uniform length and a flattened terminus.

Character 60 in Smith & Caron ([2015](#ref-Smith2015)) and Yang *et al.* ([2015](#ref-Yang2015)).

*Ovatiovermis cribratus*: Two series of spines, arranged in chevrons ([Caron & Aria 2017](#ref-Caron2017)).

### [106] Length of spines on secondary structure

**Supplementary Figure 106: Trunk appendages: Length of spines on secondary structure**

- Inapplicable

1 short/equant

2 needle-like

Luolishaniids have long setiform spines ([Conway Morris & Robison 1988](#ref-ConwayMorris1988); [Ma *et al.* 2009](#ref-Ma2009); [García-Bellido *et al.* 2013](#ref-Garcia2013); [Yang *et al.* 2015](#ref-Yang2015); [Caron & Aria 2020](#ref-Caron2020)), which contrast with the short, more equant spines of *Diania* and *Aysheaia* ([Whittington 1978](#ref-Whittington1978); [Ma *et al.* 2014*a*](#ref-Ma2014jsp)).

Character 61 in Yang *et al.* ([2015](#ref-Yang2015)).

### [107] Papillae on non-sclerotized (lobopodous) limbs

**Supplementary Figure 107: Trunk appendages: Papillae on non-sclerotized (lobopodous) limbs**

0 absent

1 present

In contrast to appendicules and spines, papillae are short projections associated with the annulations. The preservation of papillae in *Ilyodes* indicates that the absence of papillae in *Carbotubulus* is not taphonomic ([Haug *et al.* 2012*b*](#ref-Haug2012cb)). Ambiguous in euarthropods as sclerotization is considered to overprint and obscure any papillae that may have been present.

Character 10 in Ma *et al.* ([2014*a*](#ref-Ma2014jsp)); 61 in Smith & Caron ([2015](#ref-Smith2015)) and 62 in Yang *et al.* ([2015](#ref-Yang2015)).

*Facivermis yunnanicus*: Seemingly present ([Howard *et al.* 2020](#ref-Howard2020)).

*Ovatiovermis cribratus*: Absent ([Caron & Aria 2017](#ref-Caron2017)).

### [108] Papillae with terminal spine

**Supplementary Figure 108: Trunk appendages: Papillae with terminal spine**

0 spine absent

1 spine present

A cuticularized spine is borne by the papillae of the partial Orsten-type lobopodian and crown-group onychophorans.

Character 77 in Zhang *et al.* ([2016](#ref-Zhang2016)).

### [109] Finger-like elements in distal tip of limbs

**Supplementary Figure 109: Trunk appendages: Finger-like elements in distal tip of limbs**

0 absent

1 present

The finger-like projections in the legs of tardigrades can bear sets of terminal claws or sucking discs ([Schuster *et al.* 1980](#ref-Schuster1980); [Nelson 2002](#ref-Nelson2002)).

Character 62 in Smith & Caron ([2015](#ref-Smith2015)) and 63 in Yang *et al.* ([2015](#ref-Yang2015)).

### [110] Claws on trunk limbs

**Supplementary Figure 110: Trunk appendages: Claws on trunk limbs**

0 absent

1 present

From character 63 in Smith & Caron ([2015](#ref-Smith2015)) and 64 in Yang *et al.* ([2015](#ref-Yang2015)).

*Anomalocaris canadensis*: Coded ambiguous, as there is no definitive information on the presence of lobopodous limbs or a second set of flaps ([Van Roy *et al.* 2015](#ref-VanRoy2015)).

*Aysheaia*: The lobopod claws of *Aysheaia* are sub-terminal; the lobopods extend beyond the claws ([Whittington 1978](#ref-Whittington1978)).

*Cricocosmia*: Coded as ambiguous: the potential homology between the pair of terminal hooks of *Cricocosmia* and the similarly-shaped claws on trunk appendages ([Steiner *et al.* 2012](#ref-Steiner2012)) is difficult to evaluate.

*Diania*: Coded as ambiguous as it is difficult to distinguish possible terminal claws from its myriad accessory spines ([Liu *et al.* 2011](#ref-Liu2011); [Ma *et al.* 2014*a*](#ref-Ma2014jsp); [Ou & Mayer 2018](#ref-Ou2018)).

*Facivermis yunnanicus*: The “trunk spines” ([Howard *et al.* 2020](#ref-Howard2020)) are interpreted as corresponding to terminal claws on vestigial trunk limbs.

*Hallucigenia sparsa*: *Hallucigenia* *sparsa* is coded with two claws as this is the state on most trunk limbs, even if a second claw is not evident on the posteriormost appendages ([Smith & Caron 2015](#ref-Smith2015)).

*Megadictyon*, *Jianshanopodia*: *Jianshanopodia* ([Liu *et al.* 2006](#ref-Liu2006)) and *Megadictyon* ([Liu *et al.* 2007](#ref-Liu2007az)) are also coded as uncertain as the preservation of the type material does not allow the presence or absence of terminal claws to be confirmed.

*Leanchoilia*: *Leanchoilia* is coded as ambiguous for one or three claws to reflect the conflicting interpretations of García-Bellido & Collins ([2007](#ref-Garcia2007)) and Haug *et al.* ([2012*a*](#ref-Haug2012bmceb)).

*Luolishania*: Coded as present (one claw) as this represents the state of its typical trunk limbs. Spinose elements on its anterior limbs do not exhibit a claw-like morphology and may represent cirri rather than claws.

*Opabinia*: Absent, following Budd & Daley ([2012](#ref-Budd2012)).

*Paucipodia*: Simple elongate claws ([Vannier & Martin 2017](#ref-Vannier2017)).

*Thanahita distos*: Present ([Siveter *et al.* 2018](#ref-Siveter2018)).

*Youti yuanshi*: As claws are not evident until a rather late stage of onychophoran development ([Walker & Tait 2004](#ref-Walker2004)), we cannot be confident that their absence in YKLP 12387 (this study) reflects the adult condition.

### [111] Morphology

**Supplementary Figure 111: Trunk appendages: Claws: Morphology**

- Inapplicable

1 no enlarged base (e.g. *Paucipodia*’s claws)

2 enlarged base (e.g. Onychophora claws)

The outer edge of e.g. onychophoran claws have a similar curvature along its length, whereas the inner edge has a distinct inflection/step in curvature along its length, forming an enlarged attachment base.

*Actinarctus*: Coded as ambiguous as difficult to score for this character due to modification/organization of claws (see [Gallo D’Addabbo *et al.* 1999](#ref-Gallo1999)).

*Alalcomenaeus*: see Briggs & Collins ([1999](#ref-Briggs1999)).

*Aysheaia*: Enlarged base; figured in supplementary material of Smith & Ortega-Hernández ([2014](#ref-Smith2014)).

*Cardiodictyon*: No enlarged base ([Ramsköld & Chen 1998](#ref-Ramskold1998)).

*Cricocosmia*: Following Dhungana ([2024](#ref-Dhungana2024)).

*Emu Bay Collins monster*: Enlarged base ([García-Bellido *et al.* 2013](#ref-Garcia2013)).

*Hallucigenia sparsa*: *Hallucigenia*’s claws do not have an enlarged base, with similar curvature through the length of the claw ([Smith & Ortega-Hernández 2014](#ref-Smith2014)).

*Halobiotus*: Eutardigrades have a two-branched claw with differing morphologies, however, the base of most claws appear enlarged [including Halobiotidae, Doryphoribiidae, Eohysibiidae, Rhichtersiidae; see Gąsiorek *et al.* ([2019](#ref-Gasiorek2019))] hence we code this as present for this taxon.

*Leanchoilia*: No enlarged base ([García-Bellido & Collins 2007](#ref-Garcia2007)).

*Luolishania*: Enlarged base in the claws of posterior lobopods ([Ma *et al.* 2009 fig. 10](#ref-Ma2009)).

*Onychodictyon gracilis*: The claws of *Onychodictyon* *gracilis* appear to have an enlarged base (see [Liu *et al.* 2008*b*](#ref-Liu2008app), fig 2A6), although few other claws have been described.

*Paucipodia*: *Paucipodia*’s claws do not have an enlarged base ([Vannier & Martin 2017](#ref-Vannier2017)).

### [112] Claw position

**Supplementary Figure 112: Trunk appendages: Claw position**

- Inapplicable

1 terminal

2 sub-terminal

Whilst many lobopodians have terminal claws, *Aysheaia*’s claws are sub-terminal; lobopods extend beyond the claws ([Whittington 1978](#ref-Whittington1978)).
Inapplicable when terminal or sub-terminal claws absent.

*Cricocosmia*: Following Dhungana ([2024](#ref-Dhungana2024)).

*Paucipodia*: Vannier & Martin ([2017](#ref-Vannier2017)) (fig. 5a, 5b) indicate that *Paucipodia*’s claws are sub-terminal; however, there is a possibility that this is taphonomic (cf. [Murdock *et al.* 2014](#ref-Murdock2014)), as the musculature attached to the claws may have shrunk relative to the cuticle, giving the false impression of sub-terminal claws. As previous studies describe the claws as terminal ([Hou *et al.* 2004](#ref-Hou2004)), we code claw position as ambiguous.

### [113] Terminal claws with multiple branches

**Supplementary Figure 113: Trunk appendages: Terminal claws with multiple branches**

0 absent

1 present

Present in Eutardigrada ([Schuster *et al.* 1980](#ref-Schuster1980); [Nelson 2002](#ref-Nelson2002); [Halberg *et al.* 2009](#ref-Halberg2009)) and the Siberian Orsten-type tardigrade ([Maas & Waloszek 2001](#ref-Maas2001)). Absent in heterotardigrades and Palaeozoic lobopodians, which express simple concavo-convex claws.

Character 64 in Smith & Caron ([2015](#ref-Smith2015)) and 65 in Yang *et al.* ([2015](#ref-Yang2015)).

*Cricocosmia*: Following Dhungana ([2024](#ref-Dhungana2024)).

### [114] Maximum number of claws on walking limbs

**Supplementary Figure 114: Trunk appendages: Maximum number of claws on walking limbs**

- Inapplicable

1 one

2 two

3 three

4 four

5 seven

The differentiated anterior appendages of hallucishaniids do not bear unambiguous claws: structures interpreted as such (e.g. in *Ovatiovermis*, *Luolishania*) are not morphologically or compositionally distinct from co-occurring setae/spinules. As such, only the walking trunk limbs are considered.

Character 65 in Smith & Caron ([2015](#ref-Smith2015)) and 66 in Yang *et al.* ([2015](#ref-Yang2015)).

*Cardiodictyon*: *Cardiodictyon* unambiguously has two claws on each leg ([Ramsköld & Chen 1998](#ref-Ramskold1998)).

*Collinsium*: No claws on anterior appendages; single claw on each posterior appendage ([Yang *et al.* 2015](#ref-Yang2015)).

*Cricocosmia*: Following Dhungana ([2024](#ref-Dhungana2024)).

*Emu Bay Collins monster*: Single claw present on posterior appendages; claws are not apparent on cirrate anterior appendages ([García-Bellido *et al.* 2013](#ref-Garcia2013)).

*Hallucigenia hongmeia*: Single claw ([Steiner *et al.* 2012](#ref-Steiner2012)).

*Hallucigenia sparsa*: Two claws on anterior trunk appendages, one on posterior ([Smith & Caron 2015](#ref-Smith2015)).

*Leanchoilia*: Coded as ambiguous (one or three claws) to reflect the conflicting interpretations of García-Bellido & Collins ([2007](#ref-Garcia2007)) and Haug *et al.* ([2012*a*](#ref-Haug2012bmceb)).

*Luolishania*: Only one claw is observed on the unmodified (i.e. posterior) trunk limbs ([Ma *et al.* 2009](#ref-Ma2009)). Spinose elements on anterior limbs do not exhibit a claw-like morphology and may represent cirri rather than claws.

*Microdictyon*: Two claws. Though Hou & Bergström ([1995](#ref-Hou1995zjls)) observe a single claw, they leave open the possibility of a second; Liu *et al.* ([2008*b*](#ref-Liu2008app)) interpret the presence of two claws.

*Onychodictyon ferox*: A pair of claws occurs on each appendage ([Liu *et al.* 2008*b*](#ref-Liu2008app)).

*Onychodictyon gracilis*: A pair of claws is evident in one appendage ([Liu *et al.* 2008*b*](#ref-Liu2008app)), and taken to represent the typical number.

*Ovatiovermis cribratus*: The two “claws” on anterior limbs have the same shape and elemental composition as cirri ([Caron & Aria 2017](#ref-Caron2017)), so are not treated as homologous with claws. The posterior appendages each bear a single claw ([Caron & Aria 2017](#ref-Caron2017)).

*Paucipodia*: Hou *et al.* ([2004](#ref-Hou2004)) report that each lobopod carries two claws, whereas Vannier & Martin ([2017](#ref-Vannier2017)) only observe a single claw on the two complete and exceptionally well preserved appendages of ELI-JS0001A. We interpret this discrepancy as representing variation in claw number between appendages.

*Thanahita distos*: One on some appendages, two on others ([Siveter *et al.* 2018](#ref-Siveter2018)).

### [115] Number of claws varies between appendages

**Supplementary Figure 115: Trunk appendages: Number of claws varies between appendages**

- Inapplicable

1 equal number of claws on all claw-bearing appendages

2 variable number of claws

In many lobopodians, posterior trunk appendages bear fewer claws than anterior appendages.

*Cardiodictyon*: *Cardiodictyon* unambiguously has two claws on each leg ([Ramsköld & Chen 1998](#ref-Ramskold1998)).

*Cricocosmia*: Equal number ([Dhungana 2024](#ref-Dhungana2024)).

*Emu Bay Collins monster*: Single claw presumed on all posterior appendages.

*Hallucigenia sparsa*: Two claws on anterior trunk appendages, one on posterior ([Smith & Caron 2015](#ref-Smith2015)).

*Onychodictyon ferox*: A pair of claws occurs on each appendage ([Liu *et al.* 2008*b*](#ref-Liu2008app)).

*Onychodictyon gracilis*: Only one appendage is adequately preserved to identify claws ([Liu *et al.* 2008*b*](#ref-Liu2008app)).

*Ovatiovermis cribratus*: Two claws on anterior limbs, one on posterior ([Caron & Aria 2017](#ref-Caron2017)).

*Paucipodia*: Hou *et al.* ([2004](#ref-Hou2004)) report that each lobopod carries two claws, whereas Vannier & Martin ([2017](#ref-Vannier2017)) only observe a single claw on the two complete and exceptionally well preserved appendages of ELI-JS0001A. We interpret this discrepancy as representing variation in claw number between appendages.

*Thanahita distos*: One on some appendages, two on others ([Siveter *et al.* 2018](#ref-Siveter2018)).

### [116] Nature of claws on each trunk limb

**Supplementary Figure 116: Trunk appendages: Nature of claws on each trunk limb**

- Inapplicable

1 claws on single limb all identical

2 claws on single limb differentiated

Character 66 in Smith & Caron ([2015](#ref-Smith2015)).

Character 66 in Smith & Caron ([2015](#ref-Smith2015)).

*Aysheaia*: All seven claws are identical ([Whittington 1978](#ref-Whittington1978)).

*Cricocosmia*: Following Dhungana ([2024](#ref-Dhungana2024)).

*Euperipatoides*: *Euperipatoides* claws are identical on trunk limbs, although the jaw elements are differentiated ([Smith & Ortega-Hernández 2014](#ref-Smith2014)).

*Hallucigenia sparsa*: Not visibly differentiated ([Smith & Caron 2015](#ref-Smith2015)).

*Onychodictyon ferox*: *Onychodictyon* *ferox* has a large and a small claw ([Steiner *et al.* 2012 fig. 8](#ref-Steiner2012)).

*Paucipodia*: Not visibly differentiated ([Hou *et al.* 2004](#ref-Hou2004)).

### [117] Differentiated distal foot in lobopodous trunk limbs

**Supplementary Figure 117: Trunk appendages: Differentiated distal foot in lobopodous trunk limbs**

0 absent

1 present

A movable foot is present in the Onychophoran crown group, but not in *Tertiapatus* ([Poinar 2000](#ref-Poinar2000)).

Character 67 in Yang *et al.* ([2015](#ref-Yang2015)).

*Youti yuanshi*: Ambiguous as distal foot does not arise in Onychophora until a late stage in development ([Walker & Tait 2004](#ref-Walker2004)).

### [118] Strengthening rays in lateral flaps

**Supplementary Figure 118: Trunk appendages: Strengthening rays in lateral flaps**

0 absent

1 present

Character 37 in Daley *et al.* ([2009](#ref-Daley2009)); 69 in Smith & Caron ([2015](#ref-Smith2015)) and Yang *et al.* ([2015](#ref-Yang2015)).

*Amplectobelua symbrachiata*: “Oblique veins” Chen *et al.* ([1994](#ref-Chen1994)) interpreted as strengthening rays.

*Hurdia victoria*: Treated as ambiguous by Moysiuk & Caron ([2019](#ref-Moysiuk2019)).

*Lyrarapax unguispinus*: Present in *L. trilobus* ([Cong *et al.* 2016](#ref-Cong2016)); possibly reflected by striations in *L. unguispinus*?

### [119] Posterior tapering of lateral flaps

**Supplementary Figure 119: Trunk appendages: Posterior tapering of lateral flaps**

- Inapplicable

1 absent

2 even body outline

3 present

4 pronounced decrease in lobe width posteriad

Character 40 in Daley *et al.* ([2009](#ref-Daley2009)): “Posterior tapering of the width of the lateral lobes is pronounced in *Anomalocaris* and *Laggania*, while other lateral lobe-bearing taxa, including *Hurdia*, have a more even body outline.”

Character 70 in Smith & Caron ([2015](#ref-Smith2015)) and Yang *et al.* ([2015](#ref-Yang2015)).

*Cambroraster falcatus*: Relatively even ([Moysiuk & Caron 2019](#ref-Moysiuk2019)).

### [120] Anteriormost limb pair hypertrophied

**Supplementary Figure 120: Trunk appendages: Anteriormost limb pair hypertrophied**

0 first pair of trunk limbs comparable in size to subsequent pairs

1 first pair of trunk limbs hypertrophied

The first pair of body flaps (posterior of segments lacking flaps) are enlarged into “paddles” in *Schinderhannes* and *Lyrarapax* ([Kühl *et al.* 2009](#ref-Kuhl2009); [Cong *et al.* 2014](#ref-Cong2014), [2016](#ref-Cong2016)). Because the body flaps of these radiodontans are homologous with endopods and lobopods ([Van Roy *et al.* 2015](#ref-VanRoy2015)), this character has been generalized from Yang *et al.* ([2016](#ref-Yang2016)) in order to apply to all appendage-bearing taxa.

Character 68 in Yang *et al.* ([2015](#ref-Yang2015)).

### [121] Anterior limbs reduced

**Supplementary Figure 121: Trunk appendages: Anterior limbs reduced**

0 no reduction of anterior limbs

1 anterior limbs reduced in size or absent

In *Lyrarapax*, *Hurdia*, *Peytoia* and *Anomalocaris*, the flaps of the anterior region are reduced ([Daley *et al.* 2009](#ref-Daley2009); [Cong *et al.* 2014](#ref-Cong2014); [Daley & Edgecombe 2014](#ref-Daley2014)), whereas in *Opabinia*, *Kerygmachela* and *Pambdelurion*, the equivalent flaps remain expressed ([Whittington 1975](#ref-Whittington1975); [Budd 1998*a*](#ref-Budd1998ar), [*b*](#ref-Budd1998trse)). The preservation of *Aegirocassis* and *Schinderhannes* in inadequate to resolve this feature.
Because the ventral body flaps of the radiodonts are homologous with endopods and lobopods ([Van Roy *et al.* 2015](#ref-VanRoy2015)), this character has been generalized from Yang *et al.* ([2015](#ref-Yang2015)) and Yang *et al.* ([2016](#ref-Yang2016)) in order to apply to all limb-bearing taxa.

Character 71 in Yang *et al.* ([2015](#ref-Yang2015)).

*Cambroraster falcatus*: First three flaps reduced ([Moysiuk & Caron 2019](#ref-Moysiuk2019)).

*Carbotubulus*: Single anterior pair reduced in size ([Haug *et al.* 2012*b*](#ref-Haug2012cb)); as it is unclear whether this represents a homologous reduction, we code as ambiguous.

### [122] Number of limbs on differentiated anterior trunk

**Supplementary Figure 122: Trunk appendages: Number of limbs on differentiated anterior trunk**

- Inapplicable

1 two

2 three

3 five

4 six

*Hallucigenia* *fortis* has two pairs of elongate limbs ([Ma *et al.* 2012*b*](#ref-Ma2012asd)); *Hallucigenia* *sparsa* has three ([Smith & Caron 2015](#ref-Smith2015)); *Luolishania*, *Facivermis*, *Acinocricus* and the Emu Bay Collins Monster have five ([Ramsköld & Chen 1998](#ref-Ramskold1998); [Ma *et al.* 2009](#ref-Ma2009); [García-Bellido *et al.* 2013](#ref-Garcia2013); [Howard *et al.* 2020](#ref-Howard2020)); *Collinsium*, *Ovatiovermis* and *Collinsovermis* bear six ([Yang *et al.* 2015](#ref-Yang2015); [Caron & Aria 2017](#ref-Caron2017), [2020](#ref-Caron2020)).

Character 73 in Yang *et al.* ([2015](#ref-Yang2015)).

### [123] Nature of lobopodous limbs on differentiated anterior trunk

**Supplementary Figure 123: Trunk appendages: Nature of lobopodous limbs on differentiated anterior trunk**

- Inapplicable

1 slender, simple

2 cirrate

The anterior limbs of *Hallucigenia* *sparsa* are simple and lack cirri; the anterior limbs of luolishaniids bear multiple cirri. The trunk is not differentiated into distinct anterior and posterior components in any other taxon.

Character 71 in Yang *et al.* ([2015](#ref-Yang2015)).

### [124] Appendages comprise 15 or more podomeres

**Supplementary Figure 124: Trunk appendages: Appendages comprise 15 or more podomeres**

- Inapplicable

1 Fewer than 15 podomeres

2 15 or more podomeres

The endopods of certain taxa in the euarthropod stem-group, such as fuxianhuiids, bear 15 or more podomeres and are considered “multipodomerous” ([Chen *et al.* 1995*c*](#ref-Chen1995s); [Waloszek *et al.* 2005](#ref-Waloszek2005); [Bergström *et al.* 2008](#ref-Bergstrom2008); [Yang *et al.* 2013](#ref-Yang2013)).

Character 72 in Smith & Caron ([2015](#ref-Smith2015)) and 74 in Yang *et al.* ([2015](#ref-Yang2015)).

## Posterior termination

### [125] Limbless posterior extension of the lobopodous trunk

**Supplementary Figure 125: Posterior termination: Limbless posterior extension of the lobopodous trunk**

0 absent

1 present: tubular portion of the body extends beyond the last observable appendage pair

This character has been modified by that of previous analyses (e.g. character 34 in [Ma *et al.* 2014*a*](#ref-Ma2014jsp)) to reflect the fact that, in extant onychophorans, the posterior extension of the lobopodous trunk (i.e. anal cone) corresponds to a segment that has lost its appendage pair, as evinced by the prevalence of nephridia in this region ([Mayer & Koch 2005](#ref-Mayer2005)). As it is not possible to determine whether the posterior extension of the trunk in Palaeozoic lobopodians arises through the loss of the last appendage pair (as in Onychophora) or as an elongation of the trunk, we code this character as present in all taxa where the trunk extends posteriad of the last observable pair of limbs.

Character 73 in Smith & Caron ([2015](#ref-Smith2015)) and 75 in Yang *et al.* ([2015](#ref-Yang2015)).

*Jianshanopodia*, *Kerygmachela*, *Anomalocaris canadensis*: We code this character as absent in *Kerygmachela* ([Budd 1993](#ref-Budd1993), [1998*b*](#ref-Budd1998trse)), *Jianshanopodia* ([Liu *et al.* 2006](#ref-Liu2006)) and *Anomalocaris* ([Daley & Edgecombe 2014](#ref-Daley2014)) as their tails likely represent modified appendages.

*Carbotubulus*: Absent ([Haug *et al.* 2012*b*](#ref-Haug2012cb)).

*Cricocosmia*: Present ([Dhungana 2024](#ref-Dhungana2024)).

*Facivermis yunnanicus*: Pear-shaped posterior bulge ([Howard *et al.* 2020](#ref-Howard2020)).

*Hallucigenia fortis*, *Hallucigenia hongmeia*: *H. fortis* and *H. hongmeia* are coded as ambiguous, as the preservation is insufficiently clear to determine whether possible “posterior extensions” correspond to the trunk or to legs ([Hou & Bergström 1995](#ref-Hou1995zjls); [Steiner *et al.* 2012](#ref-Steiner2012); [Liu & Dunlop 2014](#ref-Liu2014ppp)).

*Hallucigenia sparsa*: Absent ([Smith & Caron 2015](#ref-Smith2015)).

*Luolishania*: Although *Luolishania* is described as bearing a protruding posterior termination, this is not unambiguously evident in specimens or camera lucida images; this taxon is thus coded as ambiguous ([Liu *et al.* 2008*a*](#ref-Liu2008csb); [Ma *et al.* 2009](#ref-Ma2009)).

*Microdictyon*: Present ([Chen *et al.* 1995*a*](#ref-Chen1995bnmns)).

*Opabinia*: There is possible, but inconclusive, evidence for a small posterior extension in *Opabinia* ([Whittington 1975](#ref-Whittington1975); [Budd 1996](#ref-Budd1996); [Budd & Daley 2012](#ref-Budd2012)), which is thus coded as uncertain.

*Ovatiovermis cribratus*: Absent ([Caron & Aria 2017](#ref-Caron2017)).

*Pambdelurion*: We score *Pambdelurion* as uncertain because its posterior trunk is poorly known ([Budd 1998*a*](#ref-Budd1998ar)).

*Siberion*: *Siberion* is scored as uncertain as it is difficult to distinguish the possible body termination from a posterior leg or pair of legs ([Dzik 2011](#ref-Dzik2011)).

*Thanahita distos*: Conical extension present ([Siveter *et al.* 2018](#ref-Siveter2018)).

*Youti yuanshi*: The posterior appendages are incompletely formed at this developmental stage (this study), so it is impossible to evaluate their condition in mature individuals.

### [126] Posterior tagma composed of three paired lateral flaps

**Supplementary Figure 126: Posterior termination: Posterior tagma composed of three paired lateral flaps**

0 absent

1 present

Character 42 in Daley *et al.* ([2009](#ref-Daley2009)), 74 in Smith & Caron ([2015](#ref-Smith2015)) and 76 in Yang *et al.* ([2015](#ref-Yang2015)).

*Amplectobelua symbrachiata*: *Amplectobelua* “resembles *Anomalocaris* in the number of lateral flaps, the flap venation, tail fan, and long furcae” ([Chen *et al.* 1994](#ref-Chen1994)).

*Pambdelurion*: We score *Pambdelurion* as uncertain because its posterior trunk is poorly known ([Budd 1998*a*](#ref-Budd1998ar)).

### [127] Posteriormost pair of trunk appendages structurally differentiated

**Supplementary Figure 127: Posterior termination: Posteriormost pair of trunk appendages structurally differentiated**

0 undifferentiated

1 differentiated

Character 75 in Smith & Caron ([2015](#ref-Smith2015)) and 77 in Yang *et al.* ([2015](#ref-Yang2015)). See also character 35 in Ma *et al.* ([2014*a*](#ref-Ma2014jsp)).

*Amplectobelua symbrachiata*: *Amplectobelua* “resembles *Anomalocaris* in the number of lateral flaps, the flap venation, tail fan, and long furcae” ([Chen *et al.* 1994](#ref-Chen1994)).

*Cambroraster falcatus*: Several pairs of lobes incorporated into tail fan ([Moysiuk & Caron 2019](#ref-Moysiuk2019)).

*Cricocosmia*: Undifferentiated ([Dhungana 2024](#ref-Dhungana2024)).

*Euperipatoides*, *Plicatoperipatus*, *Ooperipatellus*: Onychophora are scored as undifferentiated, as the posteriormost appendages are appendages are lost, not structurally differentiated ([Mayer & Koch 2005](#ref-Mayer2005)).

*Helenodora*: The preservation is inadequate to evaluate this feature.

*Hurdia victoria*: *Hurdia* and *Schinderhannes* bear a single flap-like appendage on the posterior end ([Daley *et al.* 2009](#ref-Daley2009); [Kühl *et al.* 2009](#ref-Kuhl2009)).

*Jianshanopodia*: We score *Jianshanopodia* ([Liu *et al.* 2006](#ref-Liu2006)) as present because the lateral extensions of the tail fan likely correspond to a modified pair of appendages.

*Pambdelurion*: We score *Pambdelurion* as uncertain because its posterior trunk is poorly known ([Budd 1998*a*](#ref-Budd1998ar)).

### [128] Nature of differentiated posteriormost appendages

**Supplementary Figure 128: Posterior termination: Nature of differentiated posteriormost appendages**

- Inapplicable

1 appendicular tail

2 partially fused/reduced walking legs

In fuxianhuiids, the posteriormost appendage pair is modified into a tail fan or tail flukes (e.g. [Chen *et al.* 1995*c*](#ref-Chen1995s); [Yang *et al.* 2013](#ref-Yang2013)); a similar condition is also observed in *Opabinia* ([Whittington 1975](#ref-Whittington1975); [Budd 1996](#ref-Budd1996); [Budd & Daley 2012](#ref-Budd2012)), *Anomalocaris* ([Daley & Edgecombe 2014](#ref-Daley2014)) and *Hurdia* ([Daley *et al.* 2009](#ref-Daley2009)). Partial fusion of the last pair of legs occurs in *Aysheaia* ([Whittington 1978](#ref-Whittington1978)), *Onychodictyon* *gracilis* ([Liu *et al.* 2008*b*](#ref-Liu2008app)), *O. ferox* ([Ou *et al.* 2012](#ref-Ou2012)) and Tardigrada (e.g. [Halberg *et al.* 2009](#ref-Halberg2009); [Marchioro *et al.* 2013](#ref-Marchioro2013)); in these taxa, this characteristic is expressed as an incipient fusion of the medioproximal bases of the posteriormost appendage pair.

Character 76 in Smith & Caron ([2015](#ref-Smith2015)) and 78 in Yang *et al.* ([2015](#ref-Yang2015)).

*Amplectobelua symbrachiata*: *Amplectobelua* “resembles *Anomalocaris* in the number of lateral flaps, the flap venation, tail fan, and long furcae” ([Chen *et al.* 1994](#ref-Chen1994)).

*Jianshanopodia*: The last appendage pair of *Jianshanopodia* is modified into a set of lateral flaps, which form a tail fan together with the flattened terminal portion of the body ([Liu *et al.* 2006](#ref-Liu2006)).

*Kerygmachela*: The paired tail rami of *Kerygmachela* ([Budd 1993](#ref-Budd1993), [1998*b*](#ref-Budd1998trse)) likely represent modified appendages.

*Pambdelurion*: We score *Pambdelurion* as uncertain because its posterior trunk is poorly known ([Budd 1998*a*](#ref-Budd1998ar)).

*Siberian Orsten tardigrade*: The Siberian Orsten tardigrade is scored as having a reduced posteriormost appendage pair based on the vestigial rudiment present on its posteroventral body region ([Maas & Waloszek 2001](#ref-Maas2001)).

### [129] Nature

**Supplementary Figure 129: Posterior termination: Appendicular tail: Nature**

- Inapplicable

1 tail rami

2 tail flaps

This character distinguishes the long tail rami of *Kerygmachela* ([Budd 1993](#ref-Budd1993), [1998*b*](#ref-Budd1998trse)) from the flaps observed in *Jianshanopodia* ([Liu *et al.* 2006](#ref-Liu2006)), *Opabinia* ([Budd 1996](#ref-Budd1996); [Budd & Daley 2012](#ref-Budd2012)), anomalocaridids ([Daley *et al.* 2009](#ref-Daley2009); [Daley & Edgecombe 2014](#ref-Daley2014)), and fuxianhuiids (e.g. [Yang *et al.* 2013](#ref-Yang2013)).

Character 77 in Smith & Caron ([2015](#ref-Smith2015)) and 79 in Yang *et al.* ([2015](#ref-Yang2015)).

This character distinguishes the long tail rami of *Kerygmachela* ([Budd 1993](#ref-Budd1993), [1998*b*](#ref-Budd1998trse)) from the flaps observed in *Jianshanopodia* ([Liu *et al.* 2006](#ref-Liu2006)), *Opabinia* ([Budd 1996](#ref-Budd1996); [Budd & Daley 2012](#ref-Budd2012)), anomalocaridids ([Daley *et al.* 2009](#ref-Daley2009); [Daley & Edgecombe 2014](#ref-Daley2014)), and fuxianhuiids (e.g. [Yang *et al.* 2013](#ref-Yang2013)).

Character 77 in Smith & Caron ([2015](#ref-Smith2015)) and 79 in Yang *et al.* ([2015](#ref-Yang2015)).

*Amplectobelua symbrachiata*: *Amplectobelua* “resembles *Anomalocaris* in the number of lateral flaps, the flap venation, tail fan, and long furcae” ([Chen *et al.* 1994](#ref-Chen1994)).

*Pambdelurion*: We score *Pambdelurion* as uncertain because its posterior trunk is poorly known ([Budd 1998*a*](#ref-Budd1998ar)).

### [130] Flaps: Morphology

**Supplementary Figure 130: Posterior termination: Appendicular tail: Flaps: Morphology**

0 blade-like

1 paddle-like

Opabiniids ([Budd & Daley 2012](#ref-Budd2012); [Pates *et al.* 2022](#ref-Pates2022)) and Anomalocaridids ([Daley *et al.* 2009](#ref-Daley2009)) have differentiated posterior appendages that form a tail fan. Fuxianhuiids have similar modified appendicular tail flukes (e.g. [Yang *et al.* 2013](#ref-Yang2013)). *Opabinia* regalis has a paddle-like, more symmetric morphology to its tail appendages, whereas *Anomalocaris* and *Utaurora* appendages are more asymmetric, with a sharp anterior edge forming a blade-like morphology ([Pates *et al.* 2022](#ref-Pates2022)).
Coded as inapplicable when posterior tagma tail flaps are absent.

Character 106 from Pates *et al.* ([2022](#ref-Pates2022)).

*Chengjiangocaris*: Tail flukes appear more paddle-like than blade like ([Yang *et al.* 2013](#ref-Yang2013), supplementary figure 4b).

### [131] Direction of claws on posteriormost appendage pair

**Supplementary Figure 131: Posterior termination: Direction of claws on posteriormost appendage pair**

- Inapplicable

1 same direction as claws on other appendages

2 rotated anteriad

The last pair of legs are rotated anteriad in tardigrades (e.g. [Marchioro *et al.* 2013](#ref-Marchioro2013)), *Aysheaia* ([Whittington 1978](#ref-Whittington1978)) and *O. ferox* ([Ou *et al.* 2012](#ref-Ou2012)), but not in *O. gracilis*, *Cardiodictyon*, *Hallucigenia* *fortis* or *Microdictyon* ([Hou & Bergström 1995](#ref-Hou1995zjls)).

Character 78 in Smith & Caron ([2015](#ref-Smith2015)) and 80 in Yang *et al.* ([2015](#ref-Yang2015)).

The last pair of legs are rotated anteriad in tardigrades (e.g. [Marchioro *et al.* 2013](#ref-Marchioro2013)), *Aysheaia* ([Whittington 1978](#ref-Whittington1978)) and *O. ferox* ([Ou *et al.* 2012](#ref-Ou2012)), but not in *O. gracilis*, *Cardiodictyon*, *Hallucigenia* *fortis* or *Microdictyon* ([Hou & Bergström 1995](#ref-Hou1995zjls)).

Character 78 in Smith & Caron ([2015](#ref-Smith2015)) and 80 in Yang *et al.* ([2015](#ref-Yang2015)).

*Cricocosmia*: Following Dhungana ([2024](#ref-Dhungana2024)).

*Hallucigenia sparsa*: The claws of *Hallucigenia* *sparsa* seem to be oriented in the same direction on all appendage pairs ([Smith & Caron 2015](#ref-Smith2015)).

*Onychodictyon gracilis*: Uncertain ([Liu *et al.* 2008*b*](#ref-Liu2008app)).

*Ovatiovermis cribratus*: Claw direction on posteriormost pair (appendage 9) matches that of adjacent appendages (7 and 8) ([Caron & Aria 2017](#ref-Caron2017)). Caron & Aria ([2017](#ref-Caron2017)) assert that the posteriormost two or three claws of *Hallucigenia* and *Collinsium* are directed in a different direction to those of other trunk limbs, citing references that do not obviously support this assertion.

*Pambdelurion*: We score *Pambdelurion* as uncertain because its posterior trunk is poorly known ([Budd 1998*a*](#ref-Budd1998ar)).

*Siberian Orsten tardigrade*: Uncertain ([Maas *et al.* 2007](#ref-Maas2007csb)).

## Neuroanatomy

### [132] Ventral nerve cord with paired ganglia

**Supplementary Figure 132: Neuroanatomy: Ventral nerve cord with paired ganglia**

0 absent

1 present

Tardigrada and Euarthropoda have a ganglionated ventral nerve cord ([Schulze *et al.* 2014](#ref-Schulze2014)), in contrast to the ladder-like ventral nerve cord in Onychophora ([Mayer *et al.* 2013*a*](#ref-Mayer2013bmceb)). Priapulida have an unpaired nerve cord associated with a net-like system of neural connectives ([Storch 1991](#ref-Storch1991); [Rothe & Schmidt-Rhaesa 2010](#ref-Rothe2010)).

Character 2 in Tanaka *et al.* ([2013](#ref-Tanaka2013)), 79 in Smith & Caron ([2015](#ref-Smith2015)) and 81 in Yang *et al.* ([2015](#ref-Yang2015)).

*Chengjiangocaris*, *Alalcomenaeus*: Recent data on the neurological organization of stem-euarthropods indicate that paired ganglia are present in *Chengjiangocaris* ([Yang *et al.* 2013](#ref-Yang2013)) and *Alalcomenaeus* ([Tanaka *et al.* 2013](#ref-Tanaka2013)).

*Kerygmachela*: We code this ambiguously as Park *et al.* ([2018](#ref-Park2018)) only implicitly reconstruct paired ganglia (in their figure 4); the ‘nerve cords’ referred to in the text could represent the circumpharyngeal connectives that lead to the ventral nerve cord.

*Lyrarapax unguispinus*: Ambiguous ([Cong *et al.* 2014](#ref-Cong2014)).

*Paucipodia*: Hou *et al.* ([2004](#ref-Hou2004)) (figs 2f, 4f) reported faint paired structures adjacent to the gut of *Paucipodia*, which were interpreted as potential nerve ganglia. We nevertheless code *Paucipodia* as ambiguous: the structures cannot be observed in the figured material, and are described as “faintly preserved with a pink colour” in contrast to the conspicuously dark colouration of unambiguous nervous tissue in Chengjiang-type fossils (see [Ma *et al.* 2012*a*](#ref-Ma2012n); [Tanaka *et al.* 2013](#ref-Tanaka2013); [Yang *et al.* 2013](#ref-Yang2013)).

### [133] Dorsal condensed brain

**Supplementary Figure 133: Neuroanatomy: Dorsal condensed brain**

0 absent

1 present

Whereas typical cycloneuralians have a circumoesophageal nerve ring (e.g. [Storch 1991](#ref-Storch1991); [Telford *et al.* 2008](#ref-Telford2008); [Edgecombe 2009](#ref-Edgecombe2009); [Rothe & Schmidt-Rhaesa 2010](#ref-Rothe2010)), Panarthropoda is characterized by dorsal condensed brain neuromeres ([Eriksson *et al.* 2003](#ref-Eriksson2003); [Mittmann & Scholtz 2003](#ref-Mittmann2003); [Harzsch *et al.* 2005](#ref-Harzsch2005asd); [Mayer *et al.* 2010](#ref-Mayer2010), [2013*b*](#ref-Mayer2013po)). A dorsal condensed brain has been described in *Fuxianhuia* ([Ma *et al.* 2012*a*](#ref-Ma2012n)) and *Alalcomenaeus* ([Tanaka *et al.* 2013](#ref-Tanaka2013)).

Character 80 in Smith & Caron ([2015](#ref-Smith2015)) and 82 in Yang *et al.* ([2015](#ref-Yang2015)).

*Kerygmachela*: Present ([Park *et al.* 2018](#ref-Park2018)).

### [134] Number of neuromeres integrated into the dorsal condensed brain

**Supplementary Figure 134: Neuroanatomy: Number of neuromeres integrated into the dorsal condensed brain**

0 one

1 two

2 three

See the introductory statements for char. 81 in Smith & Caron ([2015](#ref-Smith2015)) and char. 83 in Yang *et al.* ([2015](#ref-Yang2015)).

*Kerygmachela*: The brain is protocerebral ([Park *et al.* 2018](#ref-Park2018)).

### [135] Mouth innervation relative to brain neuromeres

**Supplementary Figure 135: Neuroanatomy: Mouth innervation relative to brain neuromeres**

0 protocerebral innervation

1 deutocerebral innervation

2 innervation from multiple neuromeres

3 tritocerebral innervation

Recent fossil data suggest a likely deutocerebral innervation for the mouth in *Fuxianhuia* and *Alalcomenaeus* based on the position of the oesophageal foramen relative to the brain ([Ma *et al.* 2012*a*](#ref-Ma2012n); [Tanaka *et al.* 2013](#ref-Tanaka2013)), which is congruent with the organization found in phylogenetically basal extant euarthropods such as Chelicerata and Myriapoda ([Mittmann & Scholtz 2003](#ref-Mittmann2003); [Harzsch *et al.* 2005](#ref-Harzsch2005asd); [Scholtz & Edgecombe 2005](#ref-Scholtz2005), [2006](#ref-Scholtz2006)). Tritocerebral innervation is observed in Pancrustacea, but not among the taxa included in this study.

Character 82 in Smith & Caron ([2015](#ref-Smith2015)) and 84 in Yang *et al.* ([2015](#ref-Yang2015)).

*Actinarctus*, *Halobiotus*: The tardigrade mouth cone is innervated from the protocerebrum ([Mayer *et al.* 2013*b*](#ref-Mayer2013po)).

*Euperipatoides*, *Plicatoperipatus*, *Ooperipatellus*: Onychophora are coded as innervated from multiple neuromeres to reflect their complex neurological organization: although the jaws have a deutocerebral segmental affinity and innervation, the lip papillae that delineate the oral opening are formed as epidermal derivatives of the three anteriormost body segments, and thus receive nervous terminals from the protocerebrum, deutocerebrum and part of the ventral nerve cord ([Eriksson & Budd 2000](#ref-Eriksson2000); [Martin & Mayer 2014](#ref-Martin2014)).

*Lyrarapax unguispinus*: *Lyrarapax* has protocerebral mouth innervation ([Cong *et al.* 2014](#ref-Cong2014)).

### [136] Circumoral nerve ring

**Supplementary Figure 136: Neuroanatomy: Circumoral nerve ring**

0 absent

1 present

[NEW]
Circumpharyngeal nerve rings are found in the nematode brain ([White *et al.* 1997](#ref-White1997); [Henne *et al.* 2017](#ref-Henne2017)) and the anterior nervous systems of extant tardigrades ([Mayer *et al.* 2013*a*](#ref-Mayer2013bmceb); [Smith *et al.* 2017](#ref-Smith2017)).

*Euperipatoides*, *Plicatoperipatus*, *Ooperipatellus*: Following Martin *et al.* ([2022](#ref-Martin2022)), who argue that the circumpharyngeal connective represents the last vestiges of the circumoral nerve ring.

*Kerygmachela*: The ‘nerve cords’ interpreted by Park *et al.* ([2018](#ref-Park2018)) could represent circumoral connectives (interpreted by Martin *et al.* ([2022](#ref-Martin2022)) as homologous to the circumoral nerve ring).

*Lyrarapax unguispinus*: Not interpreted as present ([Cong *et al.* 2014](#ref-Cong2014); [Park *et al.* 2018](#ref-Park2018)).

*Tubiluchus*: [Present in nematodes].

### [137] Ventral nerve cord (VNC) paired

**Supplementary Figure 137: Neuroanatomy: Ventral nerve cord (VNC) paired**

0 unpaired

1 paired

This neomorphic character codes the transformation from a single ventral nerve cord (e.g., priapulans) to a pair (e.g., extant panarthropods, *Chengjiangocaris*).

Character 85 in Yang *et al.* ([2016](#ref-Yang2016)).

*Euperipatoides*, *Plicatoperipatus*, *Ooperipatellus*, *Actinarctus*, *Halobiotus*, *Chengjiangocaris*: Paired ([Yang *et al.* 2016](#ref-Yang2016)).

*Alalcomenaeus*: Paired ([Tanaka *et al.* 2013](#ref-Tanaka2013)).

*Kerygmachela*: Tentatively interpreted as paired, unfused ([Park *et al.* 2018](#ref-Park2018)).

*Lyrarapax unguispinus*: Paired. Two descending tracts on the anterior trunk region ([Cong *et al.* 2014](#ref-Cong2014)).

*Paucipodia*: Ambiguous. Hou *et al.* ([2004](#ref-Hou2004)) report the presence of a ventral nerve cord, although it is not possible to discern if it is paired or not ([Yang *et al.* 2016](#ref-Yang2016)).

*Tubiluchus*: Unpaired see Yang *et al.* ([2016](#ref-Yang2016)).

### [138] VNC with morphologically discrete condensed hemiganglia connected by medial commissures

**Supplementary Figure 138: Neuroanatomy: VNC with morphologically discrete condensed hemiganglia connected by medial commissures**

0 hemiganglia absent

1 morphologically discrete condensed hemiganglia connected by medial commissures

Neural concentrations (ganglia) along the ventral nerve cord give a “rope ladder-like” appearance in tardigrades and euarthropods, in contrast to a ladder-like VNC, found in onychophorans ([Yang *et al.* 2016](#ref-Yang2016)). The presence of transverse commissures likely are fundamentally linked neurological features ([Yang *et al.* 2016](#ref-Yang2016)).

Character 86 in Yang *et al.* ([2016](#ref-Yang2016)).

### [139] Paired nerve cord lateralized

**Supplementary Figure 139: Neuroanatomy: Paired nerve cord lateralized**

0 absent (*Alalcomenaeus*, *Fuxianhuia*, Tardigrada)

1 present (Onychophora)

Character 1 in Tanaka *et al.* ([2013](#ref-Tanaka2013)); revised by Yang *et al.* ([2016](#ref-Yang2016)) to apply only to paired nerve cords. This character distinguishes the organization of the ventral nerve cord in Onychophora (e.g. [Mayer *et al.* 2013*a*](#ref-Mayer2013bmceb)) from that in other phyla.

Character 83 in Smith & Caron ([2015](#ref-Smith2015)), 85 in Yang *et al.* ([2015](#ref-Yang2015)) and 87 in Yang *et al.* ([2016](#ref-Yang2016)).

*Kerygmachela*: Hints of a paired nerve cord in the anterior of *Kerygmachela* ([Park *et al.* 2018](#ref-Park2018)) are insufficient to establish their lateralization, though the positioning seems to correspond to that of *Lyrarapax* ([Cong *et al.* 2014](#ref-Cong2014)).

*Lyrarapax unguispinus*: Medial (Cong et al. 2014).

*Youti yuanshi*: Lateralization is reflected by the presence of discrete lateral chambers in the onychophoran lacunar system ([Jahn *et al.* 2023](#ref-Jahn2023)), in contrast to the single medial chamber observed here.

### [140] Paired nerve cord bears medial interpedal commissures

**Supplementary Figure 140: Neuroanatomy: Paired nerve cord bears medial interpedal commissures**

0 medial interpedal commissures absent

1 medial interpedal commissures present

Present in Onychophora and Tardigrada; absent in Euarthropoda, including *Alalcomenaeus*. Ambiguous in *Lyrarapax* and *Chengjiangocaris*. See Yang *et al.* ([2016](#ref-Yang2016)) for further discussion.

Character 88 in Yang *et al.* ([2016](#ref-Yang2016)).

### [141] Regularly spaced peripheral nerves along the entire length of the nerve cord

**Supplementary Figure 141: Neuroanatomy: Regularly spaced peripheral nerves along the entire length of the nerve cord**

0 absent, or not occurring regularly along entire length of nerve cord

1 present along entire length of nerve cord

Present in Priapulida, Onychophora, Tardigrada and *Chengjiangocaris*; absent in Euarthropoda and *Alalcomenaeus*. See Yang *et al.* ([2016](#ref-Yang2016)) for further discussion.

Character 93 in Yang *et al.* ([2016](#ref-Yang2016)).

### [142] Nerve cord has orthogonal organization

**Supplementary Figure 142: Neuroanatomy: Nerve cord has orthogonal organization**

0 not orthogonally organized

1 orthogonally organized

Orthogonal organization of several ring-like commissures and peripheral nerves that intersect longitudinal dorsal and lateral nerve strands to form a reticulate pattern. Present in Priapulida, Onychophora and Tardigrada. Uncertain in *Chengjiangocaris*; absent in *Alalcomenaeus* and crown Euarthropoda. See Yang *et al.* ([2016](#ref-Yang2016)) for further discussion. Contra Yang *et al.* ([2016](#ref-Yang2016)), we score this character as inapplicable in taxa where the regularly spaced peripheral nerves that constitute the transverse component of the orthogonal organization are not present.

Character 89 in Yang *et al.* ([2016](#ref-Yang2016)).

### [143] Orthogonal nerve cord has complete ring commissures

**Supplementary Figure 143: Neuroanatomy: Orthogonal nerve cord has complete ring commissures**

0 ring commissures incomplete or absent

1 complete ring commissures

Complete in Priapulida and Onychophora; incomplete in Tardigrada. Inapplicable in Euarthropoda. See Yang *et al.* ([2016](#ref-Yang2016)) for further discussion.

Character 90 in Yang *et al.* ([2016](#ref-Yang2016)).

### [144] Segmental leg nerves shifted anteriorly relative to appendages

**Supplementary Figure 144: Neuroanatomy: Segmental leg nerves shifted anteriorly relative to appendages**

0 not shifted anteriorly

1 shifted anteriorly

Anteriorly displaced in Tardigrada and Euarthropoda; not in Onychophora. Ambiguous in fossil taxa. See Yang *et al.* ([2016](#ref-Yang2016)) for further discussion.

Character 91 in Yang *et al.* ([2016](#ref-Yang2016)).

### [145] Segmental leg nerves paired

**Supplementary Figure 145: Neuroanatomy: Segmental leg nerves paired**

0 unpaired

1 paired

Two nerves innervate each leg in Onychophora and Eutardigrada, but a single nerve innervates each Euarthropod leg. The configuration is ambiguous in fossil material. See Yang *et al.* ([2016](#ref-Yang2016)) for further discussion.

Character 92 in Yang *et al.* ([2016](#ref-Yang2016)).

### [146] Stomatogastric ganglion

**Supplementary Figure 146: Neuroanatomy: Stomatogastric ganglion**

0 absent

1 present

Present in Eutardigrada and Euarthropoda; uncertain in Heterotardigrada; absent in Onychophora and Priapulida. See Yang *et al.* ([2016](#ref-Yang2016)) for further discussion.

Character 64 in Yang *et al.* ([2016](#ref-Yang2016)).

*Leanchoilia*, *Alalcomenaeus*: Coded present by proxy as unambiguously present in crown-Euarthropoda (see [Budd 2021](#ref-Budd2021)).

## Musculature

### [147] Heart

**Supplementary Figure 147: Musculature: Heart**

0 absent

1 present

Character(s) 84 in Smith & Caron ([2015](#ref-Smith2015)), 86 and 81 in Yang *et al.* ([2015](#ref-Yang2015)).

*Actinarctus*, *Halobiotus*: Coded inapplicable in tardigrades, following Budd ([2001*b*](#ref-Budd2001ed)), as their miniscule body size renders a heart unnecessary.

*Fuxianhuia*: Present ([Ma *et al.* 2014*b*](#ref-Ma2014nc)).

*Kerygmachela*: We suggest that the pericardial region represents the musculature of the heart.

### [148] Skeletal musculature

**Supplementary Figure 148: Musculature: Skeletal musculature**

0 peripheral longitudinal and circular muscle

1 metamerically arranged skeletal muscle

Budd ([2001*a*](#ref-Budd2001za)) proposes the distribution of musculature as a key phylogenetic character. The musculature of tardigrades, *Pambdelurion*, *Anomalocaris* and more derived euarthropods is metamerically arranged and runs through the body cavity, whereas muscles in cycloneuralians, onychophorans and *Kerygmachela* are seemingly dominated by longitudinal and circular structures Budd ([2001*a*](#ref-Budd2001za)).

Character 52 in Smith & Caron ([2015](#ref-Smith2015)).

*Anomalocaris canadensis*: Present ([Daley & Edgecombe 2014](#ref-Daley2014)).

*Carbotubulus*: A longitudinal arrangement of musculature is suggested by the longitudinal wrinkling ([Haug *et al.* 2012*b*](#ref-Haug2012cb)).

*Fuxianhuia*: Coded as present in *Fuxianhuia* based on a probable fuxianhuiid with muscle tissue from Kaili ([Zhu *et al.* 2004](#ref-Zhu2004)).

*Misszhouia longicaudata*, *Kuamaia lata*: The metameric distribution of musculature in artiopodans is inferred by comparison with *Campanamuta* ([Budd 2011](#ref-Budd2011)).

*Lyrarapax unguispinus*: Present ([Cong *et al.* 2014](#ref-Cong2014)).

### [149] Longitudinal peripheral musculature

**Supplementary Figure 149: Musculature: Longitudinal peripheral musculature**

0 absent

1 present

The presence of longitudinal muscles in the peripheral region (see Zhang et al., 2016) which may exist in addition to circular and/or metameric musculature. Present in priapulans and onychophorans ([Carnevali & Ferraguti 1979](#ref-Carnevali1979); [Hoyle & Williams 1980](#ref-Hoyle1980)); absent in tardigrades and euarthropods ([Halberg *et al.* 2009](#ref-Halberg2009)), and presumed absent in *Fuxianhuia*.

Character 113 in Zhang *et al.* ([2016](#ref-Zhang2016)).

*Kerygmachela*: Well-developed longitudinal muscles “appear to sheath the entire body” ([Young & Vinther 2017](#ref-Young2017)).

*Misszhouia longicaudata*: An axial distribution of longitudinal muscle is inferred in artiopodans by comparison with *Campanamuta* ([Young & Vinther 2017](#ref-Young2017)).

*Pambdelurion*: *Pambdelurion* exhibits longitudinal peripheral musculature ([Budd 1998*c*](#ref-Budd1998l); [Young & Vinther 2017](#ref-Young2017)).

### [150] Ventromedian longitudinal muscle

**Supplementary Figure 150: Musculature: Ventromedian longitudinal muscle**

0 absent

1 present

[NEW]
Observed in *Pambdelurion*, tardigrades and onychophorans ([Young & Vinther 2017](#ref-Young2017)).

*Misszhouia longicaudata*, *Kuamaia lata*: Inferred in artiopodans by comparison with *Kiisortoqia* ([Young & Vinther 2017](#ref-Young2017)).

*Pambdelurion*: Present ([Young & Vinther 2017](#ref-Young2017)).

*Tubiluchus*: Priapulans exhibit undifferentiated longitudinal muscle bands ([Young & Vinther 2017](#ref-Young2017)).

### [151] Longitudinal muscle attachment points

**Supplementary Figure 151: Musculature: Longitudinal muscle attachment points**

- Inapplicable

1 anterior and posterior of trunk only

2 successive attachment points along the body

[NEW]
In tardigrades, longitudinal muscles attach at successive points along the body; on onychophorans and gilled lobopodians, they attach only at the anterior and posterior end of the trunk ([Young & Vinther 2017](#ref-Young2017)). Inapplicable if longitudinal muscles are absent.

*Misszhouia longicaudata*, *Kuamaia lata*: The successive attachment points in artiopodans are inferred by comparison with *Campanamuta* ([Young & Vinther 2017](#ref-Young2017)).

### [152] Circular peripheral musculature

**Supplementary Figure 152: Musculature: Circular peripheral musculature**

0 absent

1 present

Present in priapulans and onychophorans ([Carnevali & Ferraguti 1979](#ref-Carnevali1979); [Hoyle & Williams 1980](#ref-Hoyle1980)); absent in tardigrades and euarthropods ([Halberg *et al.* 2009](#ref-Halberg2009)), and presumed absent in *Fuxianhuia*.

Character 114 in Zhang *et al.* ([2016](#ref-Zhang2016)).

*Kerygmachela*: Circular muscle reported by multiple studies ([Budd 1993](#ref-Budd1993), [1998*b*](#ref-Budd1998trse); [Young & Vinther 2017](#ref-Young2017)).

*Misszhouia longicaudata*, *Kuamaia lata*: The absence of circular muscle in artiopodans is inferred by comparison with *Campanamuta*, in which no circular musculature is evident despite preservation of individual myofibrils ([Young & Vinther 2017](#ref-Young2017)).

*Pambdelurion*: *Pambdelurion* exhibits longitudinal peripheral musculature; the presence of circular muscle is equivocal ([Budd 1998*c*](#ref-Budd1998l); [Young & Vinther 2017](#ref-Young2017)).

### [153] Circular musculature inside longitudinal musculature

**Supplementary Figure 153: Musculature: Circular musculature inside longitudinal musculature**

- Inapplicable

1 circular muscles inside longitudinal

2 longitudinal muscles inside circular

Longitudinal muscles occur inside circular muscles in priapulans and onychophorans ([Carnevali & Ferraguti 1979](#ref-Carnevali1979); [Hoyle & Williams 1980](#ref-Hoyle1980)).

Character 115 in Zhang *et al.* ([2016](#ref-Zhang2016)).

### [154] Box-truss

**Supplementary Figure 154: Musculature: Box-truss**

0 absent

1 present

[NEW]
Metamerically arranged dorsoventral and oblique muscles connecting the lateral and ventral muscle groups are present in tardigrades and euarthropods, resulting in a “box-truss trunk musculature system” ([Young & Vinther 2017](#ref-Young2017)).

*Kerygmachela*: Oblique muscles are evident in the anterior, but there is no good evidence of dorsoventral muscles ([Young & Vinther 2017](#ref-Young2017)).

*Pambdelurion*: Dorsoventral muscles not reported; extent of oblique muscles disputed ([Budd 1998*c*](#ref-Budd1998l); [Young & Vinther 2017](#ref-Young2017)), and orientation does not match that of box-truss.

*Tritonychus phanerosarkus*: Oblique musculature, but no dorsoventral ([Zhang *et al.* 2016](#ref-Zhang2016)).

# Summary of phylogenetic results

Phylogenetic inference was conducted using Bayesian and parsimony methods. Whereas there are many reasons to prefer implied weights to equal weights parsimony ([Goloboff *et al.* 2018](#ref-Goloboff2018); [Smith 2019*a*](#ref-Smith2019); [Ezcurra 2024](#ref-Ezcurra2024c)), neither Bayesian nor parsimony approaches consistently recover ‘better’ trees once precision and accuracy are taken into account ([Smith 2019*a*](#ref-Smith2019)). We present results of multiple methods, on the basis that relationships that are consistently recovered are less likely to represent methodological artefacts, and so more likely to represent genuine evolutionary signal in the data.

Wu *et al.* ([2023](#ref-Wu2023bl)) contend that previous phylogenetic studies do not convincingly discriminate the relationships between panarthropod phyla. We note that the Bayes Factors in their table 1 are incorrectly calculated: the log-likelihoods associated with specific hypotheses are divided, rather than subtracted.

To evaluate the strength of support for Tactopoda (Tardigrada + Euarthropoda) over Arthropoda (= Onychophora + Euarthropoda), we calculated the Bayes Factor using marginal likelihoods estimated from our own dataset using stepping stone sampling ([Xie *et al.* 2011](#ref-Xie2011sb)). For comparison, we calculated Bayes Factors from the marginal likelihood estimates presented by Wu *et al.* ([2023](#ref-Wu2023bl)).

| **Study** | **Scope** | **Bayes Factor** | **Interpretation** per Kass & Raftery ([1995](#ref-Kass1995jasa)) |
| --- | --- | --- | --- |
| Aria *et al.* ([2021](#ref-Aria2021)) | Euarthropoda | -3.66 | “strong” evidence for Arthropoda |
| Yang *et al.* ([2016](#ref-Yang2016)) | Panarthropoda | 1.99 | “positive” evidence for Tactopoda |
| Legg *et al.* ([2013](#ref-Legg2013nc)) | Euarthropoda | -8.71 | “very strong” evidence for Arthropoda (less reliable, as computed from harmonic means of likelihood estimates) |
| This study | Panarthropoda | 11.61 | “very strong” evidence for Tactopoda |

To summarize our results, we present consensus trees constructed after identifying rogue taxa using method H1 from Smith ([2022*a*](#ref-SmithRogue)). Rogue taxa are wildcards whose position varies from tree to tree due to ambiguity or conflict in their character data ([Kearney 2002](#ref-Kearney2002)); removal of such taxa from input trees reveals consistent relationships between the remaining taxa in a consensus summary tree that would otherwise be masked by the uncertain position of the rogues ([Wilkinson 1994](#ref-Wilkinson1994), [1996](#ref-Wilkinson1996mbe); [Wilkinson *et al.* 1996](#ref-Wilkinson1996hb)). Splits in Bayesian trees are labelled with their posterior probability; in parsimony trees, splits are labelled with their quartet concordance ([Minh *et al.* 2020](#ref-Minh2020mbe)).

## Bayesian analysis

Supplementary Figure 155. Majority rule consensus of Bayesian trees; splits labelled with posterior probabilities.

## Maximum parsimony

Supplementary Figure 156. Strict consensus of all implied weights parsimony trees, 6 < *k* < $\boldsymbol{\infty}$. Splits are labelled with their quartet concordance ([Minh *et al.* 2020](#ref-Minh2020mbe)).

Supplementary Figure 157. Strict consensus of results for $\boldsymbol{k}\boldsymbol{\in}\boldsymbol{3}\mathbf{,}\boldsymbol{6}$, after removal of rogue taxa. Trees obtained under these concavity constants are consistent with the homology of frontal filaments with onychophoran lip papillae. Splits are labelled with their quartet concordance ([Minh *et al.* 2020](#ref-Minh2020mbe)).

Supplementary Figure 158. Strict consensus of results for equal weights parsimony, after removal of rogue taxa. Splits are labelled with their quartet concordance ([Minh *et al.* 2020](#ref-Minh2020mbe)).

# Cluster analysis

Clusters of trees were evaluated using partitioning around medoids ([Maechler *et al.* 2022](#ref-Maechler2022)), hierarchical clustering with minimax linkage ([Murtagh 1983](#ref-Murtagh1983); [Bien & Tibshirani 2011](#ref-Bien2011)), K-means++ clustering ([Hartigan & Wong 1979](#ref-Hartigan1979); [Arthur & Vassilvitskii 2007](#ref-Arthur2007); [Smith 2020](#ref-TreeDist)), and hierarchical density based clustering ([Campello *et al.* 2015](#ref-Campello2015); [Hahsler *et al.* 2019](#ref-Hahsler2019); [Hahsler & Piekenbrock 2023](#ref-Hahsler2023)). The clustering quality under each method, with 2–14 clusters, was compared using the silhouette coefficient ([Rousseeuw 1987](#ref-Rousseeuw1987); [Maechler *et al.* 2022](#ref-Maechler2022)); high silhouette coefficients distinguish the most clearly defined clusters.

Supplementary Figure 159. Evaluation of optimal clustering

Plotting the consensus tree for each cluster can reveal resolution that is consistent within each cluster, but obscured in a single consensus of all topologies.

Supplementary Figure 160. 50% majority rule consensus trees for each cluster demonstrate the topographic changes underpinning the differences between clusters.

# Tree space mapping

To evaluate the consistency of phylogenetic results, we map the phylogenetic trees obtained by different methods using the Quartet distance ([Estabrook *et al.* 1985](#ref-Estabrook1985); [Smith 2022*b*](#ref-SmithSpace)), using the R ([R Core Team 2023](#ref-R)) packages “Quartet” ([Sand *et al.* 2014](#ref-Sand2014); [Smith 2019*b*](#ref-Quartet)) and “TreeDist” ([Smith 2020](#ref-TreeDist)). Distances in the mapping correspond to the degree of difference between tree topologies; dashed lines depict the shortest path to connect all trees, depicting any distortion introduced by the mapping ([Smith 2022*b*](#ref-SmithSpace)).

The mapping is conducted on a subsample of 24 trees from each parsimony search, and 120 samples from the Bayesian posterior tree set.

Supplementary Figure 161. Two-dimensional mapping of phylogenetic results

The mapping shows that Bayesian and parsimony trees occupy a similar region of tree space, except at low concavity constants ($k\leq6$) – consistent with evidence that higher concavity constants produce more reliable results ([Goloboff *et al.* 2018](#ref-Goloboff2018); [Smith 2019*a*](#ref-Smith2019)).

Whilst the two-dimensional mapping does not faithfully depict all aspects of the distances between pairs of trees (trustworthiness = 0.793; continuity = 0.895), the structure depicted captures the main aspects of a 6-dimensional plot, the lowest dimensionality necessary to meet trustworthiness × continuity > 0.9.

Supplementary Figure 162. The structure of tree space in higher dimensions is shown using a 6-dimensional tree space mapping, with clusters numbered and their convex hulls marked.

# References

ARIA, C. and CARON, J.-B. 2019. [A middle Cambrian arthropod with chelicerae and proto-book gills](https://doi.org/10.1038/s41586-019-1525-4). *Nature*, **573**, 586–589.

———, ZHAO, F. and ZHU, M. 2021. [Fuxianhuiids are mandibulates and share affinities with total-group Myriapoda](https://doi.org/10.1144/jgs2020-246). *Journal of the Geological Society*, **178**, jgs2020–246.

ARTHUR, D. and VASSILVITSKII, S. 2007. K-means++: The advantages of careful seeding. *Proceedings of the eighteenth annual ACM-SIAM symposium on Discrete algorithms*, 1027–1035.

BERGSTRÖM, J., HOU, X.-G., ZHANG, X.-G. and CLAUSEN, S. 2008. [A new view of the Cambrian arthropod *Fuxianhuia*](https://doi.org/10.1080/11035890809452772). *GFF*, **130**, 189–201.

BIEN, J. and TIBSHIRANI, R. 2011. [Hierarchical clustering with prototypes via minimax linkage](https://doi.org/10.1198/jasa.2011.tm10183). *Journal of the American Statistical Association*, **106**, 1075–1084.

BITSCH, C. and BITSCH, J. 2005. Evolution of eye structure and arthropod phylogeny. *In* KOENEMANN, S. and JENNER, R. A. (eds.) *Crustacea and Arthropod Relationships*, Taylor & Francis, 185–214 pp.

BOESGAARD, T. M. and KRISTENSEN, R. M. 2001. [Tardigrades from Australian marine caves. With a redescription of *Actinarctus* *neretinus* (Arthrotardigrada)](https://doi.org/10.1078/0044-5231-00033). *Zoologischer Anzeiger*, **240**, 253–264.

BRAZEAU, M. D., GUILLERME, T. and SMITH, M. R. 2019. [An algorithm for morphological phylogenetic analysis with inapplicable data](https://doi.org/10.1093/sysbio/syy083). *Systematic Biology*, **68**, 619–631.

BRIGGS, D. E. G. and ROBISON, R. A. 1984. Exceptionally preserved nontrilobite arthropods and *Anomalocaris* from the Middle Cambrian of Utah. *University of Kansas Paleontological Contributions*, **111**, 1–23.

——— and COLLINS, D. H. 1999. [The Arthropod *Alalcomenaeus* *cambricus* Simonetta, from the Middle Cambrian Burgess Shale of British Columbia](https://doi.org/10.1111/1475-4983.00104). *Palaeontology*, **42**, 953–977.

BRUCE, H. and PATEL, N. 2020. [A unified framework to homologize appendage segments across Arthropoda](https://doi.org/10.20944/preprints202004.0505.v1). *Preprints*, 2020040505.

BUDD, G. E. 1993. [A Cambrian gilled lobopod from Greenland](https://doi.org/10.1038/364709a0). *Nature*, **364**, 709–711.

———. 1996. [The morphology of *Opabinia* *regalis* and the reconstruction of the arthropod stem-group](https://doi.org/10.1111/j.1502-3931.1996.tb01831.x). *Lethaia*, **29**, 1–14.

———. 1998*c*. [Arthropod body-plan evolution in the Cambrian with an example from anomalocaridid muscle](https://doi.org/10.1111/j.1502-3931.1998.tb00508.x). *Lethaia*, **31**, 197–210.

———. 1998*a*. Stem group arthropods from the Lower Cambrian Sirius Passet fauna of North Greenland. *In* FORTEY, R. A. and THOMAS, R. H. (eds.) *Arthropod Relationships*, Vol. 55. Chapman and Hall, London, 125–138 pp.

———. 1998*b*. [The morphology and phylogenetic significance of *Kerygmachela* *kierkegaardi* Budd (Buen Formation, Lower Cambrian, N Greenland)](https://doi.org/10.1017/S0263593300002418). *Transactions of the Royal Society of Edinburgh: Earth Sciences*, **89**, 249–290.

———. 2001*a*. [Tardigrades as ‘stem-group arthropods’: The evidence from the Cambrian fauna](https://doi.org/10.1078/0044-5231-00034). *Zoologischer Anzeiger*, **240**, 265–279.

———. 2001*b*. [Why are arthropods segmented?](https://doi.org/10.1046/j.1525-142X.2001.01041.x) *Evolution & Development*, **3**, 332–342.

———. 2002. [A palaeontological solution to the arthropod head problem](https://doi.org/10.1038/417271a). *Nature*, **417**, 271–275.

———. 2008. [Head structure in upper stem-group euarthropods](https://doi.org/10.1111/j.1475-4983.2008.00752.x). *Palaeontology*, **51**, 561–573.

———. 2011. [*Campanamuta* *mantonae* gen. et. [sic] sp. nov., an exceptionally preserved arthropod from the Sirius Passet Fauna (Buen Formation, lower Cambrian, North Greenland)](https://doi.org/10.1080/14772019.2010.492644). *Journal of Systematic Palaeontology*, **9**, 217–260.

———. 2021. [The origin and evolution of the euarthropod labrum](https://doi.org/10.1016/j.asd.2021.101048). *Arthropod Structure & Development*, **62**, 101048.

——— and PEEL, J. S. 1998. A new xenusiid lobopod from the Early Cambrian Sirius Passet fauna of North Greenland. *Palaeontology*, **41**, 1201–1213.

——— and DALEY, A. C. 2012. [The lobes and lobopods of *Opabinia* *regalis* from the middle Cambrian Burgess Shale](https://doi.org/10.1111/j.1502-3931.2011.00264.x). *Lethaia*, **45**, 83–95.

CALLOWAY, C. B. 1975. [Morphology of the introvert and associated structures of the priapulid *Tubiluchus* *corallicola* from Bermuda](https://doi.org/10.1007/BF00391628). *Marine Biology*, **31**, 161–174.

CAMPELLO, R. J. G. B., MOULAVI, D., ZIMEK, A. and SANDER, J. 2015. [Hierarchical Density Estimates for Data Clustering, Visualization, and Outlier Detection](https://doi.org/10.1145/2733381). *ACM Transactions on Knowledge Discovery from Data*, **10**, 1–51.

CARNEVALI, M. D. C. and FERRAGUTI, M. 1979. [Structure and ultrastructure of muscles in the priapulid *Halicryptus* *spinulosus*: Functional and phylogenetic remarks](https://doi.org/10.1017/S0025315400045719). *Journal of the Marine Biological Association of the United Kingdom*, **59**, 737–744.

CARON, J.-B. and ARIA, C. 2017. [Cambrian suspension-feeding lobopodians and the early radiation of panarthropods](https://doi.org/10.1186/s12862-016-0858-y). *BMC Evolutionary Biology*, **17**, 29.

——— and ———. 2020. [The Collins’ monster, a spinous suspension-feeding lobopodian from the Cambrian Burgess Shale of British Columbia](https://doi.org/10.1111/pala.12499). *Palaeontology*, **63**, 979–994.

———, SMITH, M. R. and HARVEY, T. H. P. 2013. [Beyond the Burgess Shale: Cambrian microfossils track the rise and fall of hallucigeniid lobopodians](https://doi.org/10.1098/rspb.2013.1613). *Proceedings of the Royal Society B: Biological Sciences*, **280**, 20131613.

CHEN, J.-Y., RAMSKÖLD, L. and ZHOU, G.-Q. 1994. [Evidence for monophyly and arthropod affinity of Cambrian giant predators](https://doi.org/10.1126/science.264.5163.1304). *Science*, **264**, 1304–1308.

———, ZHOU, G.-Q. and RAMSKÖLD, L. 1995*a*. The Cambrian lobopodian *Microdictyon* *sinicum*. *Bulletin of the National Museum of Natural Science*, **5**, 1–93.

———, ZHOU, G.-Q. and RAMSKÖLD, L. 1995*b*. [A new Early Cambrian onychophoran-like animal, *Paucipodia* gen. nov., from the Chengjiang fauna, China](https://doi.org/10.1017/S0263593300002042). *Transactions of the Royal Society of Edinburgh: Earth Sciences*, **85**, 275–282.

———, EDGECOMBE, G. D. and RAMSKÖLD, L. 1997. [Morphological and ecological disparity in naraoiids (Arthropoda) from the Early Cambrian Chengjiang fauna, China](https://doi.org/10.3853/j.0067-1975.49.1997.249). *Records of the Australian Museum*, **49**, 1–24.

———, WALOSZEK, D. and MAAS, A. 2004. [A new ‘great-appendage’ arthropod from the Lower Cambrian of China and homology of chelicerate chelicerae and raptorial antero-ventral appendages](https://doi.org/10.1080/00241160410004764). *Lethaia*, **37**, 3–20.

———, EDGECOMBE, G. D., RAMSKÖLD, L. and ZHOU, G.-Q. 1995*c*. [Head segmentation in Early Cambrian *Fuxianhuia*: Implications for arthropod evolution](https://doi.org/10.1126/science.268.5215.1339). *Science*, **268**, 1339–1343.

CHIPMAN, A. D. and EDGECOMBE, G. D. 2019. [Developing an integrated understanding of the evolution of arthropod segmentation using fossils and evo-devo](https://doi.org/10.1098/rspb.2019.1881). *Proceedings of the Royal Society B: Biological Sciences*, **286**, 20191881.

CONG, P.-Y., DALEY, A. C., EDGECOMBE, G. D. and HOU, X.-G. 2017. [The functional head of the Cambrian radiodontan (stem-group Euarthropoda) *Amplectobelua* *symbrachiata*](https://doi.org/10.1186/s12862-017-1049-1). *BMC Evolutionary Biology*, **17**, 208.

———, MA, X.-Y., HOU, X.-G., EDGECOMBE, G. D. and STRAUSFELD, N. J. 2014. [Brain structure resolves the segmental affinity of anomalocaridid appendages](https://doi.org/10.1038/nature13486). *Nature*, **513**, 538–542.

———, ———, ———, HOU, X. and CHEN, A. 2016. [Morphology of the radiodontan *Lyrarapax* from the early Cambrian Chengjiang biota](https://doi.org/10.1017/jpa.2016.67). *Journal of Paleontology*.

CONWAY MORRIS, S. 1977. Fossil priapulid worms. *Special Papers in Palaeontology*, **20**, 1–95.

——— and ROBISON, R. A. 1988. More soft-bodied animals and algae from the Middle Cambrian of Utah and British Columbia. *The University of Kansas Paleontological Contributions*, **122**, 23–84.

DALEY, A. C. and BUDD, G. E. 2010. [New anomalocaridid appendages from the Burgess Shale, Canada](https://doi.org/10.1111/j.1475-4983.2010.00955.x). *Palaeontology*, **53**, 721–738.

——— and BERGSTRÖM, J. 2012. [The oral cone of *Anomalocaris* is not a classic "peytoia"](https://doi.org/10.1007/s00114-012-0910-8). *Naturwissenschaften*, **99**, 501–504.

——— and EDGECOMBE, G. D. 2014. [Morphology of *Anomalocaris* *canadensis* from the Burgess Shale](https://doi.org/10.1666/13-067). *Journal of Paleontology*, **88**, 68–91.

———, ——— and CARON, J.-B. 2013*a*. [Morphology and systematics of the anomalocaridid arthropod *Hurdia* from the Middle Cambrian of British Columbia and Utah](https://doi.org/10.1080/14772019.2012.732723). *Journal of Systematic Palaeontology*, **11**, 743–787.

———, ———, CARON, J.-B., EDGECOMBE, G. D. and COLLINS, D. H. 2009. [The Burgess Shale anomalocaridid *Hurdia* and its significance for early euarthropod evolution](https://doi.org/10.1126/science.1169514). *Science*, **323**, 1597–1600.

———, PATERSON, J. R., EDGECOMBE, G. D., GARCÍA-BELLIDO, D. C. and JAGO, J. B. 2013*b*. [New anatomical information on *Anomalocaris* from the Cambrian Emu Bay Shale of South Australia and a reassessment of its inferred predatory habits](https://doi.org/10.1111/pala.12029). *Palaeontology*, **56**, 971–990.

DASTYCH, H., KRAUS, H. and THALER, K. 2003. Redescription and notes on the biology of the glacier tardigrade *Hypsibius* *klebelsbergi* Mihelčič, 1959 (Tardigrada), based on material from the Ötztal Alps. *Mitteilungen aus dem Hamburgischen Zoologischen Museum und Institut*, **100**, 77–100.

DE VIVO, G., LAUTENSCHLAGER, S. and VINTHER, J. 2021. [Three-dimensional modelling, disparity and ecology of the first Cambrian apex predators](https://doi.org/10.1098/rspb.2021.1176). *Proceedings of the Royal Society B: Biological Sciences*, **288**, 20211176.

DEWEL, R. A. and EIBYE-JACOBSEN, J. 2006. [The mouth cone and mouth ring of *Echiniscus* *viridissimus* Peterfi, 1956 (Heterotardigrada) with comparisons to corresponding structures in other tardigrades](https://doi.org/10.1007/s10750-005-1406-8). *Hydrobiologia*, **558**, 41–51.

DHUNGANA, A. 2024. The origin and early evolution of Panarthropoda.

——— and SMITH, M. R. 2021. Is early euarthropod evolution characterized by ‘creeps’ or ‘jerks’? *Palaeontological Association annual meeting*, 22.

DZIK, J. 2011. The xenusian-to-anomalocaridid transition within the lobopodians. *Bollettino della Società Paleontologica Italiana*, **50**, 65–74.

DZIK, J. and KRUMBIEGEL, G. 1989. [The oldest ’onychophoran’ *Xenusion*: A link connecting phyla?](https://doi.org/10.1111/j.1502-3931.1989.tb01679.x) *Lethaia*, **22**, 169–181.

EDGECOMBE, G. D. 2009. [Palaeontological and molecular evidence linking arthropods, onychophorans, and other Ecdysozoa](https://doi.org/10.1007/s12052-009-0118-3). *Evolution: Education and Outreach*, **2**, 178–190.

——— and RAMSKÖLD, L. 1999. [Relationships of Cambrian Arachnata and the systematic position of Trilobita](https://doi.org/10.1017/S0022336000027761). *Journal of Paleontology*, **73**, 263–287.

ELZINGA, R. J. 1998. [Microspines in the alimentary canal of Arthropoda, Onychophora, Annelida](https://doi.org/10.1016/S0020-7322(98)00027-0). *International Journal of Insect Morphology and Embryology*, **27**, 341–349.

ERIKSSON, B. J. and BUDD, G. E. 2000. [Onychophoran cephalic nerves and their bearing on our understanding of head segmentation and stem-group evolution of Arthropoda](https://doi.org/10.1016/s1467-8039(00)00027-x). *Arthropod Structure & Development*, **29**, 197–209.

ERIKSSON, B. J., TAIT, N. N. and BUDD, G. E. 2003. [Head development in the onychophoran *Euperipatoides* *kanangrensis* with particular reference to the central nervous system](https://doi.org/10.1002/jmor.10034). *Journal of Morphology*, **255**, 1–23.

ERIKSSON, B. J., TAIT, N. N., BUDD, G. E. and AKAM, M. 2009. [The involvement of *Engrailed* and *Wingless* during segmentation in the onychophoran *Euperipatoides* *kanangrensis* (Peripatopsidae: Onychophora) (Reid 1996)](https://doi.org/10.1007/s00427-009-0287-7). *Development Genes and Evolution*, **219**, 249–264.

———, ———, ———, JANSSEN, R. and AKAM, M. 2010. [Head patterning and Hox gene expression in an onychophoran and its implications for the arthropod head problem](https://doi.org/10.1007/s00427-010-0329-1). *Development Genes and Evolution*, **220**, 117–122.

ESTABROOK, G. F., MCMORRIS, F. R. and MEACHAM, C. A. 1985. [Comparison of undirected phylogenetic trees based on subtrees of four evolutionary units](https://doi.org/10.2307/sysbio/34.2.193). *Systematic Zoology*, **34**, 193–200.

EZCURRA, M. D. 2024. [Exploring the effects of weighting against homoplasy in genealogies of palaeontological phylogenetic matrices](https://doi.org/10.1111/cla.12581). *Cladistics*, online ahead of print.

FU, D.-J., ZHANG, X.-L. and SHU, D.-G. 2011. [Soft anatomy of the Early Cambrian arthropod *Isoxys* *curvirostratus* from the Chengjiang biota of South China with a discussion on the origination of great appendages](https://doi.org/10.4202/app.2010.0090). *Acta Palaeontologica Polonica*, **56**, 843–852.

GABRIEL, W. N. and GOLDSTEIN, B. 2007. [Segmental expression of Pax3/7 and Engrailed homologs in tardigrade development](https://doi.org/10.1007/s00427-007-0152-5). *Development Genes and Evolution*, **217**, 421–433.

GALLO D’ADDABBO, M., PIETANZA, R., D’ADDABBO, R., DE LUCIA MORONE, M. R. and DE ZIO GRIMALDI, S. 1999. A redescription of *Actinarctus* *doryphorus* (Tardigrada, Heterotardigrada). *Cahiers de Biologie Marine*, **40**, 21–27.

GARCÍA-BELLIDO, D. C. and COLLINS, D. H. 2007. [Reassessment of the genus *Leanchoilia* (Arthropoda, Arachnomorpha) from the Middle Cambrian Burgess Shale, British Columbia, Canada](https://doi.org/10.1111/j.1475-4983.2007.00649). *Palaeontology*, **50**, 693–709.

———, EDGECOMBE, G. D., PATERSON, J. R. and MA, X.-Y. 2013. [A ‘Collins’ monster’-type lobopodian from the Emu Bay Shale Konservat-Lagerstätte (Cambrian), South Australia](https://doi.org/10.1080/03115518.2013.792456). *Alcheringa*, **37**, 474–478.

GĄSIOREK, P., STEC, D., MOREK, W. and MICHALCZYK, Ł. 2019. [Deceptive conservatism of claws: Distinct phyletic lineages concealed within Isohypsibioidea (Eutardigrada) revealed by molecular and morphological evidence](https://doi.org/10.1163/18759866-20191350). *Contributions to Zoology*, **88**, 78–132.

GOLOBOFF, P. A., TORRES, A. and ARIAS, J. S. 2018. [Weighted parsimony outperforms other methods of phylogenetic inference under models appropriate for morphology](https://doi.org/10.1111/cla.12205). *Cladistics*, **34**, 407–437.

GROSS, V., EPPLE, L. and MAYER, G. 2021. [Organization of the central nervous system and innervation of cephalic sensory structures in the water bear *Echiniscus* *testudo* (Tardigrada: Heterotardigrada) revisited](https://doi.org/10.1002/jmor.21386). *Journal of Morphology*, **282**, 1298–1312.

GUIDETTI, R., PELUFFO, J. R., ROCHA, A. M., CESARI, M. and DE PELUFFO, M. C. M. 2013. [The morphological and molecular analyses of a new South American urban tardigrade offer new insights on the biological meaning of the *Macrobiotus* *hufelandi* group of species (Tardigrada: Macrobiotidae)](https://doi.org/10.1080/00222933.2013.800610). *Journal of Natural History*, **47**, 2409–2426.

———, ALTIERO, T., MARCHIORO, T., SARZI AMADÈ, L., AVDONINA, A. M., BERTOLANI, R. and REBECCHI, L. 2012. [Form and function of the feeding apparatus in Eutardigrada (Tardigrada)](https://doi.org/10.1007/s00435-012-0149-0). *Zoomorphology*, **131**, 127–148.

GUO, J., PATES, S., CONG, P.-Y., DALEY, A. C., EDGECOMBE, G. D., CHEN, T.-M. and HOU, X.-G. 2019. [A new radiodont (stem Euarthropoda) frontal appendage with a mosaic of characters from the Cambrian (Series 2 Stage 3) Chengjiang biota](https://doi.org/10.1002/spp2.1231). *Papers in Palaeontology*, **5**, 99–110.

HAHSLER, M. and PIEKENBROCK, M. 2023. [*Dbscan: Density-based spatial clustering of applications with noise (DBSCAN) and related algorithms*](https://CRAN.R-project.org/package=dbscan).

———, ——— and DORAN, D. 2019. [dbscan: Fast density-based clustering with R](https://doi.org/10.18637/jss.v091.i01). *Journal of Statistical Software*, **91**, 1–30.

HALBERG, K. A., PERSSON, D., MØBJERG, N., WANNINGER, A. and KRISTENSEN, R. M. 2009. [Myoanatomy of the marine tardigrade *Halobiotus* *crispae* (Eutardigrada: Hypsibiidae)](https://doi.org/10.1002/jmor.10734). *Journal of Morphology*, **270**, 996–1013.

HAN, J., LIU, J.-N., ZHANG, Z.-F., ZHANG, X.-L. and SHU, D.-G. 2007. Trunk ornament on the palaeoscolecid worms *Cricocosmia* and *Tabelliscolex* from the Early Cambrian Chengjiang deposits of China. *Acta Palaeontologica Polonica*, **52**, 423–431.

HANSEN, J. and KATHOLM, A. K. 2002. A study of the genus *Amphibolus* from Disko Island with special attention on the life cycle of *Amphibolus* *nebulosus* (Eutardigrada: Eohypsibiidae). *Arctic Biology Field Course Quqertarsuaq 2002*, 129–163.

HARTIGAN, J. A. and WONG, M. A. 1979. [Algorithm AS 136: A *K*-means clustering algorithm](https://doi.org/10.2307/2346830). *Journal of the Royal Statistical Society. Series C (Applied Statistics)*, **28**, 100–108.

HARZSCH, S., WILDT, M., BATTELLE, B. and WALOSZEK, D. 2005. [Immunohistochemical localization of neurotransmitters in the nervous system of larval *Limulus* *polyphemus* (Chelicerata, Xiphosura): Evidence for a conserved protocerebral architecture in Euarthropoda](https://doi.org/10.1016/j.asd.2005.01.006). *Arthropod Structure & Development*, **34**, 327–342.

HAUG, J. T., BRIGGS, D. E. G. and HAUG, C. 2012*a*. [Morphology and function in the Cambrian Burgess Shale megacheiran arthropod *Leanchoilia* *superlata* and the application of a descriptive matrix](https://doi.org/10.1186/1471-2148-12-162). *BMC Evolutionary Biology*, **12**, 162.

———, MAYER, G., HAUG, C. and BRIGGS, D. E. G. 2012*b*. [A Carboniferous non-onychophoran lobopodian reveals long-term survival of a Cambrian morphotype](https://doi.org/10.1016/j.cub.2012.06.066). *Current Biology*, **22**, 1673–1675.

———, WALOSZEK, D., MAAS, A., LIU, Y. and HAUG, C. 2012*c*. [Functional morphology, ontogeny and evolution of mantis shrimp-like predators in the Cambrian](https://doi.org/10.1111/j.1475-4983.2011.01124.x). *Palaeontology*, **55**, 369–399.

HENNE, S., SOMBKE, A. and SCHMIDT-RHAESA, A. 2017. [Immunohistochemical analysis of the anterior nervous system of the free-living nematode *Plectus* spp. (Nematoda, Plectidae)](https://doi.org/10.1007/s00435-017-0347-x). *Zoomorphology*, **136**, 175–190.

HENZE, M. J., DANNENHAUER, K., KOHLER, M., LABHART, T. and GESEMANN, M. 2012. [Opsin evolution and expression in arthropod compound eyes and ocelli: Insights from the cricket *Gryllus* *bimaculatus*](https://doi.org/10.1186/1471-2148-12-163). *BMC Evolutionary Biology*, **12**, 163.

HOU, X.-G. and BERGSTRÖM, J. 1995. [Cambrian lobopodians–ancestors of extant onychophorans?](https://doi.org/10.1111/j.1096-3642.1995.tb00110.x) *Zoological Journal of the Linnean Society*, **114**, 3–19.

———, ——— and AHLBERG, P. 1995. [*Anomalocaris* and other large animals in the lower Cambrian Chengjiang fauna of southwest China](https://doi.org/10.1080/11035899509546213). *GFF*, **117**, 163–183.

———, ——— and YANG, J. 2006. [Distinguishing anomalocaridids from arthropods and priapulids](https://doi.org/10.1002/gj.1050). *Geological Journal*, **41**, 259–269.

———, MA, X.-Y., ZHAO, J. and BERGSTRÖM, J. 2004. [The lobopodian *Paucipodia* *inermis* from the Lower Cambrian Chengjiang fauna, Yunnan, China](https://doi.org/10.1080/00241160410006555). *Lethaia*, **37**, 235–244.

HOWARD, R. J., HOU, X., EDGECOMBE, G. D., SALGE, T., SHI, X. and MA, X. 2020. [A tube-dwelling early Cambrian lobopodian](https://doi.org/10.1016/j.cub.2020.01.075). *Current Biology*, **30**, 1529–1536.e2.

HOYLE, G. and WILLIAMS, M. 1980. [The musculature of *Peripatus* and its innervation](https://doi.org/10.1098/rstb.1980.0024). *Philosophical Transactions of the Royal Society B: Biological Sciences*, **288**, 481–510.

JAHN, H., HAMMEL, J. U., GÖPEL, T., WIRKNER, C. S. and MAYER, G. 2023. [A multiscale approach reveals elaborate circulatory system and intermittent heartbeat in velvet worms (Onychophora)](https://doi.org/10.1038/s42003-023-04797-z). *Communications Biology*, **6**, 468.

KASS, R. E. and RAFTERY, A. E. 1995. [Bayes factors](https://doi.org/10.1080/01621459.1995.10476572). *Journal of the American Statistical Association*, **90**, 773–795.

KEARNEY, M. 2002. [Fragmentary taxa, missing data, and ambiguity: Mistaken assumptions and conclusions](https://doi.org/10.1080/10635150252899824). *Systematic Biology*, **51**, 369–381.

KIHM, J.-H., SMITH, F. W., KIM, S., RHO, H. S., ZHANG, X., LIU, J. and PARK, T.-Y. S. 2023. [Cambrian lobopodians shed light on the origin of the tardigrade body plan](https://doi.org/10.1073/pnas.2211251120). *Proceedings of the National Academy of Sciences*, **120**, e2211251120.

KIRSTEUER, E. 1976. [Notes on adult morphology and larval development of *Tubiluchus* *corallicola* (Priapulida), based on in vivo and scanning electron microscopic examinations of specimens from Bermuda](https://doi.org/10.1111/j.1463-6409.1976.tb00706.x). *Zoologica Scripta*, **5**, 239–255.

KÜHL, G., BRIGGS, D. E. G. and RUST, J. 2009. [A great-appendage arthropod with a radial mouth from the Lower Devonian Hunsrück Slate, Germany](https://doi.org/10.1126/science.116658). *Science*, **323**, 771–773.

LAN, T., ZHAO, Y., ZHAO, F., HE, Y., MARTINEZ, P. and STRAUSFELD, N. J. 2021. [Leanchoiliidae reveals the ancestral organization of the stem euarthropod brain](https://doi.org/10.1016/j.cub.2021.07.048). *Current Biology*, **31**, 4397–4404.e2.

LAND, M. F. and NILSSON, D.-E. 2012. *Animal Eyes*. Oxford University Press, Oxford.

LEGG, D. A., SUTTON, M. D. and EDGECOMBE, G. D. 2013. [Arthropod fossil data increase congruence of morphological and molecular phylogenies](https://doi.org/10.1038/ncomms3485). *Nature Communications*, **4**, 2485.

LIU, J.-N. and DUNLOP, J. A. 2014. [Cambrian lobopodians: A review of recent progress in our understanding of their morphology and evolution](https://doi.org/10.1016/j.palaeo.2013.06.008). *Palaeogeography, Palaeoclimatology, Palaeoecology*, **398**, 4–15.

———, SHU, D.-G., HAN, J. and ZHANG, Z.-F. 2008*a*. [Comparative study of Cambrian lobopods *Miraluolishania* and *Luolishania*](https://doi.org/10.1007/s11434-007-0428-1). *Chinese Science Bulletin*, **53**, 87–93.

———, SHU, D.-G., HAN, J., ZHANG, Z.-F. and ZHANG, X.-L. 2006. A large xenusiid lobopod with complex appendages from the Lower Cambrian Chengjiang Lagerstätte. *Acta Palaeontologica Polonica*, **51**, 215–222.

———, SHU, D.-G., HAN, J., ZHANG, Z.-F. and ZHANG, X.-L. 2007. [Morpho-anatomy of the lobopod *Magadictyon* [sic] cf. *haikouensis* from the Early Cambrian Chengjiang Lagerstätte, South China](https://doi.org/10.1111/j.1463-6395.2007.00281.x). *Acta Zoologica*, **88**, 279–288.

———, SHU, D.-G., HAN, J., ZHANG, Z.-F. and ZHANG, X.-L. 2008*b*. [The lobopod *Onychodictyon* from the lower Cambrian Chengjiang Lagerstätte revisited](https://doi.org/10.4202/app.2008.0209). *Acta Palaeontologica Polonica*, **53**, 285–292.

———, LEROSEY-AUBRIL, R., STEINER, M., DUNLOP, J. A., SHU, D. and PATERSON, J. R. 2018. [Origin of raptorial feeding in juvenile euarthropods revealed by a Cambrian radiodontan](https://doi.org/10.1093/nsr/nwy057). *National Science Review*, **5**, 863–869.

———, STEINER, M., DUNLOP, J. A., KEUPP, H., SHU, D.-G., OU, Q., HAN, J., ZHANG, Z.-F. and ZHANG, X.-L. 2011. [An armoured Cambrian lobopodian from China with arthropod-like appendages](https://doi.org/10.1038/nature09704). *Nature*, **470**, 526–530.

LIU, Y., MAAS, A. and WALOSZEK, D. 2009. [Early development of the anterior body region of the grey widow spider *Latrodectus* *geometricus* Koch, 1841 (Theridiidae, Araneae)](https://doi.org/10.1016/j.asd.2009.04.001). *Arthropod Structure & Development*, **38**, 401–416.

———, ——— and ———. 2010. [Early embryonic development of the head region of *Gryllus* *assimilis* Fabricius, 1775 (Orthoptera, Insecta)](https://doi.org/10.1016/j.asd.2010.05.008). *Arthropod Structure & Development*, **39**, 382–395.

MA, X.-Y., HOU, X.-G. and BERGSTRÖM, J. 2009. [Morphology of *Luolishania* *longicruris* (Lower Cambrian, Chengjiang Lagerstätte, SW China) and the phylogenetic relationships within lobopodians](https://doi.org/10.1016/j.asd.2009.03.001). *Arthropod Structure & Development*, **38**, 271–291.

———, ———, EDGECOMBE, G. D. and STRAUSFELD, N. J. 2012*a*. [Complex brain and optic lobes in an early Cambrian arthropod](https://doi.org/10.1038/nature11495). *Nature*, **490**, 258–261.

———, EDGECOMBE, G. D., LEGG, D. A. and HOU, X.-G. 2014*a*. [The morphology and phylogenetic position of the Cambrian lobopodian *Diania* *cactiformis*](https://doi.org/10.1080/14772019.2013.770418). *Journal of Systematic Palaeontology*, **12**, 445–457.

———, CONG, P.-Y., HOU, X.-G., EDGECOMBE, G. D. and STRAUSFELD, N. J. 2014*b*. [An exceptionally preserved arthropod cardiovascular system from the early Cambrian](https://doi.org/10.1038/ncomms4560). *Nature Communications*, **5**, 3560.

———, ———, ALDRIDGE, R. J., SIVETER, D. J., SIVETER, D. J., GABBOTT, S. E., PURNELL, M. A., PARKER, A. R. and EDGECOMBE, G. D. 2012*b*. [Morphology of Cambrian lobopodian eyes from the Chengjiang Lagerstätte and their evolutionary significance](https://doi.org/10.1016/j.asd.2012.03.002). *Arthropod Structure & Development*, **41**, 495–504.

MAAS, A. and WALOSZEK, D. 2001. [Cambrian derivatives of the early arthropod stem lineage, pentastomids, tardigrades and lobopodians – an ’Orsten’ perspective](https://doi.org/10.1078/0044-5231-00053). *Zoologischer Anzeiger*, **240**, 451–459.

MAAS, A., MAYER, G., KRISTENSEN, R. M. and WALOSZEK, D. 2007. [A Cambrian micro-lobopodian and the evolution of arthropod locomotion and reproduction](https://doi.org/10.1007/s11434-007-0515-3). *Chinese Science Bulletin*, **52**, 3385–3392.

MAECHLER, M., ROUSSEEUW, P., STRUYF, A., HUBERT, M. and HORNIK, K. 2022. Cluster: Cluster Analysis Basics and Extensions. *Comprehensive R Archive Network*, **2.1.4**.

MARCHIORO, T., REBECCHI, L., CESARI, M., HANSEN, J. G., VIOTTI, G. and GUIDETTI, R. 2013. [Somatic musculature of Tardigrada: Phylogenetic signal and metameric patterns](https://doi.org/10.1111/zoj.12079). *Zoological Journal of the Linnean Society*, **169**, 580–603.

MARTIN, C. and MAYER, G. 2014. [Neuronal tracing of oral nerves in a velvet worm—implications for the evolution of the ecdysozoan brain](https://doi.org/10.3389/fnana.2014.00007). *Frontiers in Neuroanatomy*, **8**, 7.

———, JAHN, H., KLEIN, M., HAMMEL, J. U., STEVENSON, P. A., HOMBERG, U. and MAYER, G. 2022. [The velvet worm brain unveils homologies and evolutionary novelties across panarthropods](https://doi.org/10.1186/s12915-021-01196-w). *BMC Biology*, **20**, 26.

MAYER, G., MARTIN, C., RÜDIGER, J., KAUSCHKE, S., STEVENSON, P. A., POPRAWA, I., HOHBERG, K., SCHILL, R. O., PFLÜGER, H. and SCHLEGEL, M. 2013*a*. [Selective neuronal staining in tardigrades and onychophorans provides insights into the evolution of segmental ganglia in panarthropods](https://doi.org/10.1186/1471-2148-13-230). *BMC Evolutionary Biology*, **13**, 230.

——— and KOCH, M. 2005. [Ultrastructure and fate of the nephridial anlagen in the antennal segment of *Epiperipatus* *biolleyi* (Onychophora, Peripatidae)— evidence for the onychophoran antennae being modified legs](https://doi.org/10.1016/j.asd.2005.03.004). *Arthropod Structure & Development*, **34**, 471–480.

———, KAUSCHKE, S., RÜDIGER, J. and STEVENSON, P. A. 2013*b*. [Neural markers reveal a one-segmented head in tardigrades (water bears)](https://doi.org/10.1371/journal.pone.0059090). *PLoS ONE*, **8**, e59090.

———, WHITINGTON, P. M., SUNNUKS, P., PFLÜGER, H.-J. and SUNNUCKS, P. 2010. [A revision of brain composition in Onychophora (velvet worms) suggests that the tritocerebrum evolved in arthropods](https://doi.org/10.1186/1471-2148-10-255). *BMC Evolutionary Biology*, **10**, 255.

MAYERS, B., ARIA, C. and CARON, J.-B. 2019. [Three new naraoiid species from the Burgess Shale, with a morphometric and phylogenetic reinvestigation of Naraoiidae](https://doi.org/10.1111/pala.12383). *Palaeontology*, **62**, 19–50.

MINH, B. Q., HAHN, M. W. and LANFEAR, R. 2020. [New methods to calculate concordance factors for phylogenomic datasets](https://doi.org/10.1093/molbev/msaa106). *Molecular Biology and Evolution*, **37**, 2727–2733.

MITTMANN, B. and SCHOLTZ, G. 2003. [Development of the nervous system in the "head" of *Limulus* *polyphemus* (Chelicerata: Xiphosura): Morphological evidence for a correspondence between the segments of the chelicerae and of the (first) antennae of Mandibulata](https://doi.org/10.1007/s00427-002-0285-5). *Development Genes and Evolution*, **213**, 9–17.

MØBJERG, N., JØRGENSEN, A., KRISTENSEN, R. M. and NEVES, R. C. 2018. [Morphology and Functional Anatomy](https://doi.org/10.1007/978-3-319-95702-9_2). *In* SCHILL, R. O. (ed.) *Water Bears: The Biology of Tardigrades*, Springer International Publishing, Cham, 57–94 pp.

MOYSIUK, J. and CARON, J.-B. 2019. [A new hurdiid radiodont from the Burgess Shale evinces the exploitation of Cambrian infaunal food sources](https://doi.org/10.1098/rspb.2019.1079). *Proceedings of the Royal Society B: Biological Sciences*, **286**, 20191079.

——— and ———. 2021. [Exceptional multifunctionality in the feeding apparatus of a mid-Cambrian radiodont](https://doi.org/10.1017/pab.2021.19). *Paleobiology*, **47**, 704–724.

——— and ———. 2022. [A three-eyed radiodont with fossilized neuroanatomy informs the origin of the arthropod head and segmentation](https://doi.org/10.1016/j.cub.2022.06.027). *Current Biology*, **32**, 3302–3316.e2.

MURDOCK, D. J. E., GABBOTT, S. E., MAYER, G. and PURNELL, M. A. 2014. [Decay of velvet worms (Onychophora), and bias in the fossil record of lobopodians](https://doi.org/10.1186/s12862-014-0222-z). *BMC Evolutionary Biology*, **14**, 222.

MURTAGH, F. 1983. [A survey of recent advances in hierarchical clustering algorithms](https://doi.org/10.1093/comjnl/26.4.354). *The Computer Journal*, **26**, 354–359.

NELSON, D. R. 2002. [Current status of the Tardigrada: Evolution and ecology](https://doi.org/10.1093/icb/42.3.652). *Integrative and Comparative Biology*, **42**, 652–659.

NIELSEN, C. 2001. *Animal Evolution: Interrelationships of the Living Phyla*. Oxford University Press, Oxford.

O’FLYNN, R. J., LIU, Y., HOU, X.-G., MAI, H.-J., YU, M.-X., ZHUANG, S.-L., WILLIAMS, M., GUO, J. and EDGECOMBE, G. D. 2023. [The early Cambrian *Kylinxia* *zhangi* and evolution of the arthropod head](https://doi.org/10.1016/j.cub.2023.08.022). *Current Biology*, **33**, 4006–4013.e2.

ORTEGA-HERNÁNDEZ, J. 2015. [Homology of head sclerites in Burgess Shale euarthropods](https://doi.org/10.1016/j.cub.2015.04.034). *Current Biology*, **25**, 1625–1631.

——— and BRENA, C. 2012. [Ancestral patterning of tergite formation in a centipede suggests derived mode of trunk segmentation in trilobites](https://doi.org/10.1371/journal.pone.0052623). *PLoS ONE*, **7**, e52623.

——— and BUDD, G. E. 2016. [The nature of non-appendicular anterior paired projections in Palaeozoic total-group Euarthropoda](https://doi.org/10.1016/j.asd.2016.01.006). *Arthropod Structure & Development*, **45**, 185–199.

———, LEGG, D. and BRADDY, S. 2013. [The phylogeny of aglaspidid arthropods and the internal relationships within Artiopoda](https://doi.org/10.1111/j.1096-0031.2012.00413.x). *Cladistics*, **29**, 15–45.

OU, Q. and MAYER, G. 2018. [A Cambrian unarmoured lobopodian, †*Lenisambulatrix* *humboldti* gen. et sp. nov., compared with new material of †*Diania* *cactiformis*](https://doi.org/10.1038/s41598-018-31499-y). *Scientific Reports*, **8**, 13667.

———, SHU, D.-G. and MAYER, G. 2012. [Cambrian lobopodians and extant onychophorans provide new insights into early cephalization in Panarthropoda](https://doi.org/10.1038/ncomms2272). *Nature Communications*, **3**, 1261.

———, LIU, J.-N., SHU, D.-G., HAN, J., ZHANG, Z.-F., WAN, X.-Q. and LEI, Q.-P. 2011. [A rare onychophoran-like lobopodian from the lower Cambrian Chengjiang Lagerstätte, southwestern China, and its phylogenetic implications](https://doi.org/10.1666/09-147R2.1). *Journal of Paleontology*, **85**, 587–594.

PARK, T. Y. S., KIHM, J. H., WOO, J., PARK, C., LEE, W. Y., SMITH, M. P., HARPER, D. A. T., YOUNG, F., NIELSEN, A. T. and VINTHER, J. 2018. [Brain and eyes of *Kerygmachela* reveal protocerebral ancestry of the panarthropod head](https://doi.org/10.1038/s41467-018-03464-w). *Nature Communications*, **9**, 1019.

PATES, S., DALEY, A. C. and BUTTERFIELD, N. J. 2019. [First report of paired ventral endites in a hurdiid radiodont](https://doi.org/10.1186/s40851-019-0132-4). *Zoological Letters*, **5**, 18.

———, WOLFE, J. M., LEROSEY-AUBRIL, R., DALEY, A. C. and ORTEGA-HERNÁNDEZ, J. 2022. [New opabiniid diversifies the weirdest wonders of the euarthropod stem group](https://doi.org/10.1098/rspb.2021.2093). *Proceedings of the Royal Society B: Biological Sciences*, **289**, 20212093.

PERSSON, D. K., HALBERG, K. A., JØRGENSEN, A., MØBJERG, N. and KRISTENSEN, R. M. 2012. [Neuroanatomy of *Halobiotus* *crispae* (Eutardigrada: Hypsibiidae): Tardigrade brain structure supports the clade Panarthropoda](https://doi.org/10.1002/jmor.20054). *Journal of Morphology*, **273**, 1227–1245.

PERSSON, D. K., HALBERG, K. A., JØRGENSEN, A., MØBJERG, N. and KRISTENSEN, R. M. 2014. [Brain anatomy of the marine tardigrade *Actinarctus* *doryphorus* (Arthrotardigrada)](https://doi.org/10.1002/jmor.20207). *Journal of Morphology*, **275**, 173–190.

PILATO, G. 1972. Structure, intraspecific variability and systematic value of the buccal armature of eutardigrades. *Zeitschrift fur Zoologische Systematik und Evolutionsforschung*, **10**, 65–78.

POINAR, G. 2000. [Fossil onychophorans from Dominican and Baltic amber: *Tertiapatus* *dominicanus* n.g., n.sp. (Tertiapatidae n.fam.) and *Succinipatopsis* *balticus* n.g., n.sp. (Succinipatopsidae n.fam.) with a proposed classification of the subphylum Onychophora](https://doi.org/10.1111/j.1744-7410.2000.tb00178.x). *Invertebrate Biology*, **119**, 104–109.

POSNIEN, N., BASHASAB, F. and BUCHER, G. 2009. [The insect upper lip (labrum) is a nonsegmental appendage-like structure](https://doi.org/10.1111/j.1525-142X.2009.00356.x). *Evolution & Development*, **11**, 480–488.

R CORE TEAM. 2023. R: A language and environment for statistical computing.

RAMSKÖLD, L. 1992. [The second leg row of *Hallucigenia* discovered](https://doi.org/10.1111/j.1502-3931.1992.tb01389.x). *Lethaia*, **25**, 221–224.

——— and CHEN, J.-Y. 1998. Cambrian lobopodians: Morphology and phylogeny. *In* EDGECOMBE, G. D. (ed.) *Arthropod Fossils and Phylogeny*, Columbia University Press, New York, 107–150 pp.

ROTHE, B. H. and SCHMIDT-RHAESA, A. 2010. [Structure of the nervous system in *Tubiluchus troglodytes* (Priapulida)](https://doi.org/10.1111/j.1744-7410.2010.00185.x). *Invertebrate Biology*, **129**, 39–58.

ROUSSEEUW, P. J. 1987. [Silhouettes: A graphical aid to the interpretation and validation of cluster analysis](https://doi.org/10.1016/0377-0427(87)90125-7). *Journal of Computational and Applied Mathematics*, **20**, 53–65.

SAND, A., HOLT, M. K., JOHANSEN, J., BRODAL, G. S., MAILUND, T. and PEDERSEN, C. N. S. 2014. [tqDist: A library for computing the quartet and triplet distances between binary or general trees](https://doi.org/10.1093/bioinformatics/btu157). *Bioinformatics*, **30**, 2079–2080.

SCHMIDT-RHAESA, A., PANPENG, S. and YAMASAKI, H. 2017. [Two new species of *Tubiluchus* (Priapulida) from Japan](https://doi.org/10.1016/j.jcz.2017.03.004). *Zoologischer Anzeiger*, **267**, 155–167.

SCHOENEMANN, B. and CLARKSON, E. N. K. 2011. [Eyes and vision in the Chengjiang arthropod *Isoxys* indicating adaptation to habitat](https://doi.org/10.1111/j.1502-3931.2010.00239.x). *Lethaia*, **44**, 223–230.

——— and ———. 2023. [The median eyes of trilobites](https://doi.org/10.1038/s41598-023-31089-7). *Scientific Reports*, **13**, 3917.

SCHOLTZ, G. and EDGECOMBE, G. D. 2005. Heads, Hox and the phylogenetic position of trilobites. *In* KOENEMANN, S. and JENNER, R. A. (eds.) *Crustacean Issues 16: Crustacea and Arthropod Relationships*, CRC Press, Boca Raton, FL, 139–165 pp.

——— and ———. 2006. [The evolution of arthropod heads: Reconciling morphological, developmental and palaeontological evidence.](https://doi.org/10.1007/s00427-006-0085-4) *Development Genes and Evolution*, **216**, 395–415.

SCHULZE, C., NEVES, R. C. and SCHMIDT-RHAESA, A. 2014. [Comparative immunohistochemical investigation on the nervous system of two species of Arthrotardigrada (Heterotardigrada, Tardigrada)](https://doi.org/10.1016/j.jcz.2013.11.001). *Zoologischer Anzeiger*, **253**, 225–235.

SCHUSTER, R., NELSON, D., GRIGARICK, A. A. and CHRISTENBERRY, D. 1980. [Systematic criteria of the Eutardigrada](https://doi.org/10.2307/3226004). *Transactions of the American Microscopical Society*, **99**, 284–303.

SENA OLIVEIRA, I. de and MAYER, G. 2013. [Apodemes associated with limbs support serial homology of claws and jaws in Onychophora (velvet worms)](https://doi.org/10.1002/jmor.20171). *Journal of Morphology*, **274**, 1180–1190.

———, LÜTER, C., WOLF, K. W. and MAYER, G. 2014. [Evolutionary changes in the integument of the onychophoran *Plicatoperipatus* *jamaicensis* (Peripatidae)](https://doi.org/10.1111/ivb.12063). *Invertebrate Biology*, **133**, 274–280.

SIVETER, D. J., BRIGGS, D. E. G., SIVETER, D. J., SUTTON, M. D. and LEGG, D. 2018. [A three-dimensionally preserved lobopodian from the Herefordshire (Silurian) Lagerstätte, UK](https://doi.org/10.1098/rsos.172101). *Royal Society Open Science*, **5**, 172101.

SMITH, F. W., BARTELS, P. J. and GOLDSTEIN, B. 2017. [A hypothesis for the composition of the tardigrade brain and its implications for panarthropod brain evolution](https://doi.org/10.1093/icb/icx081). *Integrative and Comparative Biology*, **57**, 546–559.

SMITH, M. R. 2019*b*. [Quartet: Comparison of phylogenetic trees using quartet and bipartition measures](https://doi.org/10.5281/zenodo.2536318). *Comprehensive R Archive Network*, doi:10.5281/zenodo.2536318.

———. 2019*a*. [Bayesian and parsimony approaches reconstruct informative trees from simulated morphological datasets](https://doi.org/10.1098/rsbl.2018.0632). *Biology Letters*, **15**, 20180632.

———. 2020. [TreeDist: Calculate and map distances between phylogenetic trees](https://doi.org/10.5281/zenodo.3528123). *Comprehensive R Archive Network*, doi:10.5281/zenodo.3528123.

———. 2022*a*. [Using information theory to detect rogue taxa and improve consensus trees](https://doi.org/10.1093/sysbio/syab099). *Systematic Biology*, **71**, 1088–1094.

———. 2022*b*. [Robust analysis of phylogenetic tree space](https://doi.org/10.1093/sysbio/syab100). *Systematic Biology*, **71**, 1255–1270.

———. 2023. [TreeSearch: Morphological phylogenetic analysis in R](https://doi.org/10.32614/RJ-2023-019). *R Journal*, **14**, 305–315.

——— and ORTEGA-HERNÁNDEZ, J. 2014. [*Hallucigenia*’s onychophoran-like claws and the case for Tactopoda](https://doi.org/10.1038/nature13576). *Nature*, **514**, 363–366.

——— and CARON, J.-B. 2015. [*Hallucigenia*’s head and the pharyngeal armature of early ecdysozoans](https://doi.org/10.1038/nature14573). *Nature*, **523**, 75–78.

———, HARVEY, T. H. P. and BUTTERFIELD, N. J. 2015. [The macro- and microfossil record of the Cambrian priapulid *Ottoia*](https://doi.org/10.1111/pala.12168). *Palaeontology*, **58**, 705–721.

STEINER, M., HU, S., LIU, J.-N. and KEUPP, H. 2012. [A new species of *Hallucigenia* from the Cambrian Stage 4 Wulonging Fromation of Yunnan (South China) and the structure of sclerites in lobopodians](https://doi.org/10.3140/bull.geoscri.1280). *Bulletin of Geosciences*, **87**, 107–124.

STORCH, V. 1991. Priapulida. *In* HARRISON, F. W. and RUPERT, E. E. (eds.) *Microscopic Anatomy of Invertebrates Volume 4*, Wiley-Liss, Inc., New York, 333–350 pp.

TANAKA, G., HOU, X.-G., MA, X.-Y., EDGECOMBE, G. D. and STRAUSFELD, N. J. 2013. [Chelicerate neural ground pattern in a Cambrian great appendage arthropod](https://doi.org/10.1038/nature12520). *Nature*, **502**, 364–367.

TELFORD, M. J., BOURLAT, S. J., ECONOMOU, A., PAPILLON, D. and ROTA-STABELLI, O. 2008. [The evolution of the Ecdysozoa](https://doi.org/10.1098/rstb.2007.2243). *Philosophical Transactions of the Royal Society of London B: Biological Sciences*, **363**, 1529–1537.

THOMPSON, I. and JONES, D. 1980. [A possible onychophoran from the Middle Pennsylvanian Mazon Creek beds of northern Illinois](https://doi.org/10.2307/1304204). *Journal of Paleontology*, **54**, 588–596.

TOPPER, T. P., SKOVSTED, C. B., PEEL, J. S. and HARPER, D. A. T. 2013. [Moulting in the lobopodian *Onychodictyon* from the lower Cambrian of Greenland](https://doi.org/10.1111/let.12026). *Lethaia*, **46**, 490–495.

VAN DER LAND, J. 1970. Systematics, zoogeography, and ecology of the Priapulida. *Zoologische Verhandelingen*, **112**, 1–118.

VAN ROY, P., DALEY, A. C. and BRIGGS, D. E. G. 2015. [Anomalocaridid trunk limb homology revealed by a giant filter-feeder with paired flaps](https://doi.org/10.1038/nature14256). *Nature*, **522**, 77–80.

VANNIER, J. and MARTIN, E. L. O. 2017. [Worm-lobopodian assemblages from the Early Cambrian Chengjiang biota: Insight into the ‘pre-arthropodan ecology’?](https://doi.org/10.1016/j.palaeo.2016.12.002) *Palaeogeography, Palaeoclimatology, Palaeoecology*, **468**, 373–387.

———, LIU, J.-N., LEROSEY-AUBRIL, R., VINTHER, J. and DALEY, A. C. 2014. [Sophisticated digestive systems in early arthropods](https://doi.org/10.1038/ncomms4641). *Nature Communications*, **5**, 3641.

VINTHER, J., STEIN, M., LONGRICH, N. R. and HARPER, D. A. T. 2014. [A suspension-feeding anomalocarid from the Early Cambrian](https://doi.org/10.1038/nature13010). *Nature*, **507**, 496–9.

———, PORRAS, L., YOUNG, F. J., BUDD, G. E. and EDGECOMBE, G. D. 2016. [The mouth apparatus of the Cambrian gilled lobopodian *Pambdelurion* *whittingtoni*](https://doi.org/10.1111/pala.12256). *Palaeontology*.

WALKER, M. H. and TAIT, N. N. 2004. [Studies of embryonic development and the reproductive cycle in ovoviviparous Australian Onychophora (Peripatopsidae)](https://doi.org/10.1017/S0952836904005837). *Journal of Zoology*, **264**, 333–354.

WALOSZEK, D., CHEN, J.-Y., MAAS, A. and WANG, X. 2005. [Early Cambrian arthropods—new insights into arthropod head and structural evolution](https://doi.org/10.1016/j.asd.2005.01.005). *Arthropod Structure & Development*, **34**, 189–205.

WHITE, J. G., SOUTHGATE, E., THOMSON, J. N. and BRENNER, S. 1997. [The structure of the nervous system of the nematode *Caenorhabditis* *elegans*](https://doi.org/10.1098/rstb.1986.0056). *Philosophical Transactions of the Royal Society of London. B, Biological Sciences*, **314**, 1–340.

WHITTINGTON, H. B. 1975. [The enigmatic animal *Opabinia* *regalis*, Middle Cambrian, Burgess Shale, British Columbia](https://doi.org/10.1098/rstb.1975.0033). *Philosophical Transactions of the Royal Society of London B: Biological Sciences*, **271**, 1–43.

WHITTINGTON, H. B. 1978. [The lobopod animal *Aysheaia* *pedunculata* Walcott, Middle Cambrian, Burgess Shale, British Columbia](https://doi.org/10.1098/rstb.1978.0061). *Philosophical Transactions of the Royal Society of London B: Biological Sciences*, **284**, 165–197.

WHITTINGTON, H. B. 1993. [Anatomy of the Ordovician trilobite *Placoparia*](https://doi.org/10.1098/rstb.1993.0008). *Philosophical Transactions of the Royal Society B: Biological Sciences*, **339**, 109–118.

WILKINSON, M. 1994. [Common cladistic information and its consensus representation: Reduced Adams and reduced cladistic consensus trees and profiles](https://doi.org/10.2307/2413673). *Systematic Biology*, **43**, 343–368.

———. 1996. [Majority-rule reduced consensus trees and their use in bootstrapping](https://doi.org/10.1093/oxfordjournals.molbev.a025604). *Molecular Biology and Evolution*, **13**, 437–444.

———, SUTER, S. J. and SHIRES, V. L. 1996. [The reduced cladistic consensus method and cassiduloid echinoid phylogeny](https://doi.org/10.1080/08912969609386554). *Historical Biology*, **12**, 63–73.

WU, R., PISANI, D. and DONOGHUE, P. C. J. 2023. [The unbearable uncertainty of panarthropod relationships](https://doi.org/10.1098/rsbl.2022.0497). *Biology Letters*, **19**, 20220497.

XIE, W., LEWIS, P. O., FAN, Y., KUO, L. and CHEN, M.-H. 2011. [Improving marginal likelihood estimation for Bayesian phylogenetic model selection](https://doi.org/10.1093/sysbio/syq085). *Systematic Biology*, **60**, 150–160.

YANG, J., ORTEGA-HERNÁNDEZ, J., BUTTERFIELD, N. J. and ZHANG, X.-G. 2013. [Specialized appendages in fuxianhuiids and the head organization of early euarthropods](https://doi.org/10.1038/nature11874). *Nature*, **494**, 468–471.

———, ———, GERBER, S., BUTTERFIELD, N. J., HOU, J.-B., LAN, T. and ZHANG, X.-G. 2015. [A superarmored lobopodian from the Cambrian of China and early disparity in the evolution of Onychophora](https://doi.org/10.1073/pnas.1505596112). *Proceedings of the National Academy of Sciences*, **112**, 8678–8683.

———, ———, BUTTERFIELD, N. J., LIU, Y., BOYAN, G. S., HOU, J., LAN, T. and ZHANG, X. 2016. [Fuxianhuiid ventral nerve cord and early nervous system evolution in Panarthropoda](https://doi.org/10.1073/pnas.1522434113). *Proceedings of the National Academy of Sciences*, **113**, 2988–2993.

YOUNG, F. J. and VINTHER, J. 2017. [Onychophoran-like myoanatomy of the Cambrian gilled lobopodian *Pambdelurion* *whittingtoni*](https://doi.org/10.1111/pala.12269). *Palaeontology*, **60**, 27–54.

ZENG, H., ZHAO, F., NIU, K., ZHU, M. and HUANG, D. 2020. [An early Cambrian euarthropod with radiodont-like raptorial appendages](https://doi.org/10.1038/s41586-020-2883-7). *Nature*, **588**, 101–105.

ZHANG, C.-X., LIU, Y., ORTEGA-HERNÁNDEZ, J., WOLFE, J. M., JIN, C.-F., MAI, H.-J., HOU, X.-G., GUO, J. and ZHAI, D.-Y. 2023. [Three-dimensional morphology of the biramous appendages in *Isoxys* from the early Cambrian of South China, and its implications for early euarthropod evolution](https://doi.org/10.1098/rspb.2023.0335). *Proceedings of the Royal Society B: Biological Sciences*, **290**, 20230335.

ZHANG, X.-G., SMITH, M. R., YANG, J. and HOU, J.-B. 2016. [Onychophoran-like musculature in a phosphatized Cambrian lobopodian](https://doi.org/10.1098/rsbl.2016.0492). *Biology Letters*, **12**, 20160492.

ZHANG, X.-L. and BRIGGS, D. E. G. 2007. [The nature and significance of the appendages of *Opabinia* from the Middle Cambrian Burgess Shale](https://doi.org/10.1111/j.1502-3931.2007.00013.x). *Lethaia*, **40**, 161–173.

ZHU, M.-Y., VANNIER, J., VAN ITEN, H. and ZHAO, Y.-L. 2004. [Direct evidence for predation on trilobites in the Cambrian](https://doi.org/10.1098/rsbl.2004.0194). *Proceedings of the Royal Society B: Biological Sciences*, **271 Suppl**, S277–80.
