# Supplementary material for: Organ systems of a Cambrian euarthropod larva
Source: Nature. 2024 Jul 31;633(8028):120–6. doi: 10.1038/s41586-024-07756-8 (PMC11374701; doi:10.1038/s41586-024-07756-8)
Supplement: Supplementary file 2 — Reporting Summary [file 41586_2024_7756_MOESM2_ESM.pdf]

nature portfolio

Corresponding author(s): M R SMITH

Last updated by author(s): Jun 7, 2024

# Reporting Summary

Nature Portfolio wishes to improve the reproducibility of the work that we publish. This form provides structure for consistency and transparency in reporting. For further information on Nature Portfolio policies, see our Editorial Policies and the Editorial Policy Checklist.

## Statistics

For all statistical analyses, confirm that the following items are present in the figure legend, table legend, main text, or Methods section.

| n/a | Confirmed | |
|---|---|---|
| ☒ | ☐ | The exact sample size (*n*) for each experimental group/condition, given as a discrete number and unit of measurement |
| ☒ | ☐ | A statement on whether measurements were taken from distinct samples or whether the same sample was measured repeatedly |
| ☒ | ☐ | The statistical test(s) used AND whether they are one- or two-sided<br>*Only common tests should be described solely by name; describe more complex techniques in the Methods section.* |
| ☒ | ☐ | A description of all covariates tested |
| ☒ | ☐ | A description of any assumptions or corrections, such as tests of normality and adjustment for multiple comparisons |
| ☒ | ☐ | A full description of the statistical parameters including central tendency (e.g. means) or other basic estimates (e.g. regression coefficient) AND variation (e.g. standard deviation) or associated estimates of uncertainty (e.g. confidence intervals) |
| ☒ | ☐ | For null hypothesis testing, the test statistic (e.g. *F*, *t*, *r*) with confidence intervals, effect sizes, degrees of freedom and *P* value noted<br>*Give P values as exact values whenever suitable.* |
| ☐ | ☒ | For Bayesian analysis, information on the choice of priors and Markov chain Monte Carlo settings |
| ☒ | ☐ | For hierarchical and complex designs, identification of the appropriate level for tests and full reporting of outcomes |
| ☒ | ☐ | Estimates of effect sizes (e.g. Cohen's *d*, Pearson's *r*), indicating how they were calculated |

*Our web collection on statistics for biologists contains articles on many of the points above.*

## Software and code

Policy information about availability of computer code

| | |
|---|---|
| Data collection | Custom code was not used for data collection |
| Data analysis | Analysis of tomographic data: Avizo 2019.1 (Thermo Fisher Scientific, USA); Dragonfly 2022.2 (Comet Technologies Canada).<br><br>Phylogenetic analysis: MrBayes 3.2.7a; R 4.3.0 in RStudio 2023.12.1 (Posit) using phylogenetic workflow adapted from https://github.com/smithlabdurham/phylo-workflow, with packages ape v5.7, Rogue v2.1.6, TreeTools v1.9.0, TreeDist v2.7.0, TreeSearch v1.4.0, MorphoBank v0.0.0.9000 (https://github.com/ms609/MorphoBank). |

For manuscripts utilizing custom algorithms or software that are central to the research but not yet described in published literature, software must be made available to editors and reviewers. We strongly encourage code deposition in a community repository (e.g. GitHub). See the Nature Portfolio guidelines for submitting code & software for further information.

## Data

Policy information about availability of data

All manuscripts must include a data availability statement. This statement should provide the following information, where applicable:

- Accession codes, unique identifiers, or web links for publicly available datasets
- A description of any restrictions on data availability
- For clinical datasets or third party data, please ensure that the statement adheres to our policy

Fossil material is accessioned at the Key Laboratory for Palaeobiology, Yunnan University, Kunming, China (YKLP).

SEMs, virtual dissections and raw sXCT data are available at FigShare: https://doi.org/10.6084/m9.figshare.c.6490717

Phylogenetic data, results, and scripts for analysis are available at MorphoBank: https://morphobank.org/permalink/?P3927

## Research involving human participants, their data, or biological material

Policy information about studies with human participants or human data. See also policy information about sex, gender (identity/presentation), and sexual orientation and race, ethnicity and racism.

| | |
|---|---|
| Reporting on sex and gender | n/a |
| Reporting on race, ethnicity, or other socially relevant groupings | n/a |
| Population characteristics | n/a |
| Recruitment | n/a |
| Ethics oversight | n/a |

Note that full information on the approval of the study protocol must also be provided in the manuscript.

# Field-specific reporting

Please select the one below that is the best fit for your research. If you are not sure, read the appropriate sections before making your selection.

☐ Life sciences ☐ Behavioural & social sciences ☒ Ecological, evolutionary & environmental sciences

For a reference copy of the document with all sections, see nature.com/documents/nr-reporting-summary-flat.pdf

# Ecological, evolutionary & environmental sciences study design

All studies must disclose on these points even when the disclosure is negative.

| | |
|---|---|
| Study description | Observational study of fossil material by synchrotron tomography and electron microscopy |
| Research sample | A single fossil specimen, YKLP12387 |
| Sampling strategy | A single specimen is available for study |
| Data collection | The specimen was recovered by 5% acetic acid digestion of carbonate nodules from black shales of the Yu'anshan formation. Synchrotron X-ray Computed Tomography (XCT) was conducted on the imaging branch of beamline I13, Diamond Light Source under a polychromatic (pink) beam using 3.2 mm aluminimum filters and a 120 ms exposure. |
| Timing and spatial scale | n/a |
| Data exclusions | No data was excluded |
| Reproducibility | Full instructions, files and materials for reproduction of phylogenetic analyses are provided. |
| Randomization | n/a |
| Blinding | n/a |

Did the study involve field work? ☐ Yes ☒ No

# Reporting for specific materials, systems and methods

We require information from authors about some types of materials, experimental systems and methods used in many studies. Here, indicate whether each material, system or method listed is relevant to your study. If you are not sure if a list item applies to your research, read the appropriate section before selecting a response.

## Materials & experimental systems

| n/a | Involved in the study |
|---|---|
| ☒ | ☐ Antibodies |
| ☒ | ☐ Eukaryotic cell lines |
| ☐ | ☒ Palaeontology and archaeology |
| ☒ | ☐ Animals and other organisms |
| ☒ | ☐ Clinical data |
| ☒ | ☐ Dual use research of concern |
| ☒ | ☐ Plants |

## Methods

| n/a | Involved in the study |
|---|---|
| ☒ | ☐ ChIP-seq |
| ☒ | ☐ Flow cytometry |
| ☒ | ☐ MRI-based neuroimaging |

# Palaeontology and Archaeology

Specimen provenance: Black shales of the Yu'anshan Formation. No permits were necessary.

Specimen deposition: Key Laboratory for Palaeobiology, Yunnan University, Kunming, China (YKLP).

Dating methods: n/a

☒ Tick this box to confirm that the raw and calibrated dates are available in the paper or in Supplementary Information.

Ethics oversight: No ethical approval was required for this study.

Note that full information on the approval of the study protocol must also be provided in the manuscript.
